# Analysis of *Theileria orientalis* draft genome sequences reveals potential species-level divergence of the Ikeda, Chitose and Buffeli genotypes

**DOI:** 10.1186/s12864-018-4701-2

**Published:** 2018-04-27

**Authors:** Daniel R. Bogema, Melinda L. Micallef, Michael Liu, Matthew P. Padula, Steven P. Djordjevic, Aaron E. Darling, Cheryl Jenkins

**Affiliations:** 1 NSW Department of Primary Industries, Elizabeth Macarthur Agricultural Institute, Menangle, NSW Australia; 2 The ithree institute, University of Technology Sydney, Ultimo, NSW Australia

**Keywords:** *Theileria orientalis*, Genome, Phylogenomics, Vaccine

## Abstract

**Background:**

*Theileria orientalis* (Apicomplexa: Piroplasmida) has caused clinical disease in cattle of Eastern Asia for many years and its recent rapid spread throughout Australian and New Zealand herds has caused substantial economic losses to production through cattle deaths, late term abortion and morbidity. Disease outbreaks have been linked to the detection of a pathogenic genotype of *T. orientalis*, genotype Ikeda, which is also responsible for disease outbreaks in Asia. Here, we sequenced and compared the draft genomes of one pathogenic (Ikeda) and two apathogenic (Chitose, Buffeli) isolates of *T. orientalis* sourced from Australian herds.

**Results:**

Using de novo assembled sequences and a single nucleotide variant (SNV) analysis pipeline, we found extensive genetic divergence between the *T. orientalis* genotypes. A genome-wide phylogeny reconstructed to address continued confusion over nomenclature of this species displayed concordance with prior phylogenetic studies based on the major piroplasm surface protein (MPSP) gene. However, average nucleotide identity (ANI) values revealed that the divergence between isolates is comparable to that observed between other theilerias which represent distinct species. Analysis of SNVs revealed putative recombination between the Chitose and Buffeli genotypes and also between Australian and Japanese Ikeda isolates. Finally, to inform future vaccine studies, dN/dS ratios and surface location predictions were analysed. Six predicted surface protein targets were confirmed to be expressed during the piroplasm phase of the parasite by mass spectrometry.

**Conclusions:**

We used whole genome sequencing to demonstrate that the *T. orientalis* Ikeda, Chitose and Buffeli variants show substantial genetic divergence. Our data indicates that future researchers could potentially consider disease-associated Ikeda and closely related genotypes as a separate species from non-pathogenic Chitose and Buffeli.

**Electronic supplementary material:**

The online version of this article (10.1186/s12864-018-4701-2) contains supplementary material, which is available to authorized users.

## Background

*Theileria orientalis* is a tick-borne parasite that has caused outbreaks of clinical theileriosis in cattle in Japan, Korea and China [1]. Recently, outbreaks of disease have been reported in Australia and New Zealand where the disease has spread rapidly causing substantial economic losses to cattle production [2]. *Theileria* species display similar morphology [3] and, historically, species delineations have been made based on differences in location, pathogenicity, vector competency and host-pathogen interactions [4–6]. *T. orientalis* was previously split into a three-species complex (*T. sergenti/buffeli/orientalis*), however, more recently the organism is generally classified in the literature as one species (*T. orientalis*) [1, 7, 8]. This recognition has been based on more recent molecular examinations of phylogeny, which have largely focused on individual genes, such as those encoding immunogenic piroplasm surface proteins. Sequence variability in the major piroplasm surface protein (MPSP) has been used to classify the organism into 11 distinct genotypes [1, 7, 8]. Pathogenicity is associated with Type 2 (Ikeda) while other types, such as the widespread Types 1 and 3 (Chitose and Buffeli, respectively), have largely been linked to benign infections [9–11].

The genetic diversity of apicomplexan parasites allows for rapid adaptation to selective pressures, which has significant consequences for vaccine design and the development of drug resistance. Moreover, the highly diverse surfaceomes of these populations allow for the avoidance of specific immune responses thereby limiting pathogen clearance [12]. The development of inexpensive whole genome sequencing technologies that allow for direct sequencing of clinically-derived samples promises to revolutionize the study of parasitic diversity. Additionally, large scale monitoring of genetic variations in field samples can provide critical information for disease surveillance [13]. Furthermore, the study of diverse apicomplexan surfaceomes has the potential to improve the design of subunit vaccines, which currently have limited effectiveness [14].

To date, a single *T. orientalis* genome, that of the Japanese Shintoku strain (genotype Ikeda), has been genome sequenced via the Sanger method. That study revealed a 9 Mb, 4 chromosome nuclear genome structure, as well as mitochondrial and apicoplast genomes [15], a karyotype that appears to be conserved within the *Theileria* genus [16–18]. However, no current studies have focused on whole genome phylogenies of the *T. orientalis* genotypes. In the present study, we used comparative genomics to examine three Australian isolates of *T. orientalis,* namely Robertson (Ikeda), Fish Creek (Chitose) and Goon Nure (Buffeli), using Illumina technology and identified extensive genetic differences between these genotypes.

## Results

### Reference genome read mapping and de novo assembly

For each isolate, reads from three technical replicate sequencing experiments were aligned to the Shintoku reference genome (Assembly ASM74089v1) and merged to generate a single file. The Shintoku reference sequence is of the Ikeda genotype and hence the Robertson strain showed high proportions of reads mapped, reference sequence coverage and depth of sequence coverage (Table 1). Fish Creek [19] and Goon Nure isolates showed reduced percentages of mapped reads and reference coverage, indicating a high amount of sequence divergence from Ikeda. However, alignments of assembled sequences show coverage of a high proportion of the *T. orientalis* Shintoku genome sequence (Additional files 1 & 2). *Bos taurus* host DNA was detected at low levels and represented less than 1.35% of reads for all isolates. Genome assemblies produced varied results with the Robertson assembly producing longer and fewer total contigs. In contrast, assemblies of the Fish Creek and Goon Nure isolates were more fragmented (Table 1). To investigate the reason for this contrast we examined haplotype diversity to indicate if an increased in the number of quasi-species were present in the Goon Nure and Fish Creek isolates. Sequencing reads from each isolate were examined by mapping back to the assemblies and variant calling to identify biallelic SNVs. Goon Nure showed a much higher number of biallelic SNVs (27947) vs Fish Creek (4634) and Robertson (1669) indicating that assembly fragmentation is potentially caused by greater haplotype diversity and quasi-species in the Goon Nure isolate. The assembly pipeline used, A5-miseq, was designed to assemble haploid genomes of clonal, axenically-cultured microbes. When substantial genomic polymorphism is likely to be present in the data, the pipeline produces an assembly that is fragmented, with contig boundaries occurring frequently at polymorphic sites.

**Table 1:** Genome assembly and resequencing statistics

	Robertson	Fish Creek	Goon Nure
Resequencing			
% reads mapped	96.9%	75.2%	78.0%
% host DNA	0.072%	1.34%	0.069%
Genome % ref. coverage (≥ 14×)	93.1%	74.4%	65.0%
CDS % ref. coverage (≥ 14×)	96.5%	85.3%	78.3%
Mean coverage depth	60.5 ×	43.5 ×	47.3 ×
Assembly			
# scaffolds	639	1557	6043
N50	54,695	12,243	2646
% GC	41.7%	39.3%	39.8%
Predicted genes	3677	3604	4905

### SNV validation and analysis

Variation between the three isolates examined in this study was relatively high, reflecting the diversity of this parasite. Moreover, many contigs from the Fish Creek and Goon Nure genome assemblies did not align to the Shintoku reference, or other isolates from this study (Additional file 1). BLAST searches of these contigs using the NCBI non-redundant database produce no significant hits. Novel sequence and differences in gene content are discussed in more detail below.

In this study, in-depth variant analysis of all isolates was limited to SNV mutations due to reported difficulties in the analysis of indel mutations using short read alignment methods [20]. To assess isolate variation, reads were aligned to the Shintoku reference sequence and homozygous SNVs called (Table 2). To examine the effectiveness of this methodology in detecting SNV variants, we examined false discovery using simulated alignments. Simulated alignments with higher divergence to those observed between the Shintoku reference and Fish Creek/Goon Nure reads (~ 900,000 substitution events, ~ 90,000 insertion-deletion events) produced a false discovery rate of 1.8%. Variant detection was also examined with Sanger sequencing of representative sections from each genome (Additional file 3). Very high sensitivity values were observed in the Robertson isolate (Additional file 3). No false positive calls (Illumina positive/Sanger negative) were observed in any isolate, while false negative calls were very low in Robertson, but increased in Fish Creek and Goon Nure strains. When false negative calls were examined in depth it was found that all were closely associated with observed small insertion or deletion events, which have been previously reported to be problematic in SNV calling pipelines [20].

**Table 2:** SNV variance of Robertson, Fish Creek and Goon Nure strains compared with Shintoku reference

	Robertson	Fish Creek	Goon Nure
Total SNVs (Density SNV/kb)	24,132 (2.68)	788,412 (87.8)	676,284 (75.3)
Within CDS SNVs (Density SNV/kb)	16,974 (2.75)	637,070 (103)	565,947 (91.6)
% of genome (coverage adjusted)	0.288%	11.8%	11.6%
% of CDS (coverage adjusted)	0.28%	12.1%	11.7%

When comparing variation between genotypes, the Robertson isolate showed expectedly high similarity to the Shintoku reference sequence, while Fish Creek and Goon Nure strains showed similar levels of divergence (Table 2). Total numbers of variants were similar in the Fish Creek and Goon Nure strains, however, these variants were largely found in differing positions reflecting the divergence between all three isolates. Coding sequences showed a higher variant density than non-coding sequences (Table 2), but this is likely a consequence of greater coding sequence coverage (Table 1). In validation experiments with simulated reads and Sanger sequencing, we noticed that in sequence regions with a high number of short indel mutations read mapping coverage was reduced. These high indel regions seem to be more common in the non-coding genome and result in reduced coverage. Excepting chromosomal ends where sequencing coverage was low, variants were evenly distributed in Fish Creek and Goon Nure sequences (Additional file 4). Some high variation regions were identified in the Robertson sequence and these areas largely correspond to hypothetical genes (Additional files 4 & 5).

### Ortholog clustering and novel gene prescence

To explore differences in gene content between the four genomes, we examined predicted proteins using OrthoFinder [21]. Using an evidence-based annotation methodology for eukaryotic genomes, we identified 3675, 3624 and 4789 genes in the Robertson, Fish Creek and Goon Nure strains, compared with 4002 genes identified in the Shintoku genome sequence. Ortholog clustering identified that the higher gene content detected in the Goon Nure sequence is due to a higher number of genes unassigned to orthologous groups shared by the four genomes (515 vs 59–114) and a higher amount of gene duplication, which is demonstrated by increased number of orthogroups with multiple genes from Goon Nure compared to Robertson/Chitose (Additional file 6). This increase is present when OrthoFinder is run with an increased similarity threshold (e-value = 1e-100), indicating higher gene copy number could be produced by haplotype diversity as outlined above or by gene duplication within the Goon Nure genome. To examine this further we mapped sequencing reads to predicted coding sequences for each isolate with bowtie2, using the -k 1 option so that each read was only aligned once. We then examined if there was a stoichiometric-like relationship between average gene coverage and the per-isolate number of genes in an orthogroup. If duplication of genes was caused by the generation of highly similar quasi-species contigs during the assembly process, then orthogroups with higher numbers of genes per-isolate should show lower coverage in stoichiometric-like ratios. Robertson and Fish Creek showed relatively stable coverage regardless of per-isolate orthogroup gene number (Additional file 7). For Goon Nure, average gene coverage was higher in orthogroups with lower numbers of genes but differences were less than expected for a stoichiometric-like ratio, indicating that increased genome duplication or other reasons may contribute to the fragmentation observed.

To identify unique genes present within Robertson, Fish Creek and Goon Nure strains we first examined the similarity of predicted proteins to those identified from the Shintoku whole genome sequence using blastp, as this method would identify proteins that were both present in each genome and also predicted by each annotation pipeline. A total of 29, 32 and 69 predicted coding sequences from Robertson, Fish Creek and Goon Nure strains respectively did not show similarity (e-value cutoff 1e-5) to any predicted protein sequence from the *T. orientalis* Shintoku genome.

Predicted protein coding sequences that did not match Shintoku predicted proteins were examined further with tblastn to determine if they were present within the Shintoku genome sequence and identify if differences in gene content were due to mutations, annotation pipline differences, pseudogenes or inserted/deleted coding sequences. For strains Robertson, Fish Creek and Goon Nure a respective 28, 19 and 22 zero-hit blastp putative proteins matched regions of the Shintoku genome with tblastn. This resulted in a respective total of 1, 13 and 39 coding sequences from Robertson, Fish Creek and Goon Nure strains did not show similarity (e-value cutoff 1e-5) to the translated Shintoku genome sequence with either blastp or tblastn. Of the zero-hit blastp putative proteins that matched with tblastn 20 (Robertson), 8 (Fish Creek) and 11 (Goon Nure) matched intergenic regions of the Shintoku genome. A further 8, 10 and 10 predicted proteins of the respective Robertson, Fish Creek and Goon Nure genome sequences matched annotations in regions of hypothetical proteins in the Shintoku sequence, but predicted a protein in a different translational frame. Finally, one predicted hypothetical protein from both the Fish Creek and Goon Nure isolates matched the opposite strand of a proteasome component (XP_009692024.1) gene. Further inspection of this proteasome component revealed it is truncated by four C-terminal exons in Fish Creek and Goon Nure when compared to Shintoku and Robertson. However, this truncation is consistant with other piroplasmid species (*T. parva, T. annulate, B. microti* and *B. bigemina*) which are of comparable length to the predicted Fish Creek and Goon Nure proteasome component.

Zero-hit blastp putative proteins were also compared to the non-redundant protein database with BLAST (accessed 29–1-2017) to identify if any were homologous to proteins from other species. A total of 8, 7 and 10 of these putative proteins from the Robertson, Fish Creek and Goon Nure isolates respectively showed similarity to those found in the non-redundant database. These included a conserved apicomplexan specific protein (all isolates), a putative exonuclease (all isolates) and a RNA methyl-transferase (Robertson, Fish Creek). Results of blastp searches of the nr database are shown in Additional file 8.

### Recombination analysis

**Fig. 1:**
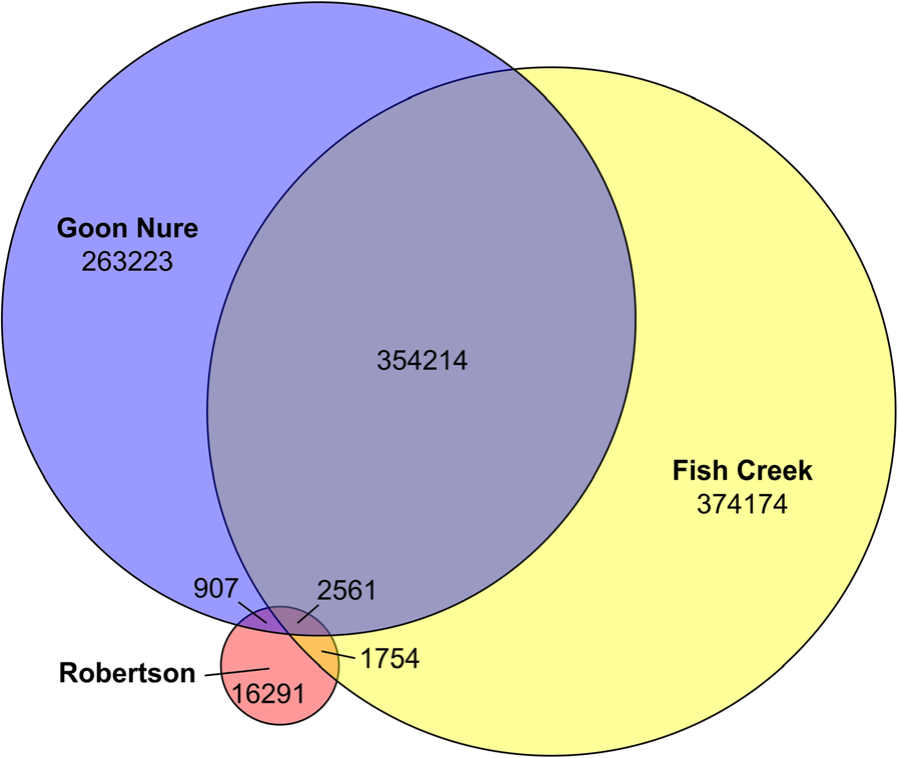
Venn diagram showing number of unique and shared SNVs in the Robertson, Fish Creek and Goon Nure genotypes. Low numbers of Robertson SNVs reflect the use of the Shintoku whole genome sequence as a reference

**Fig. 2:**
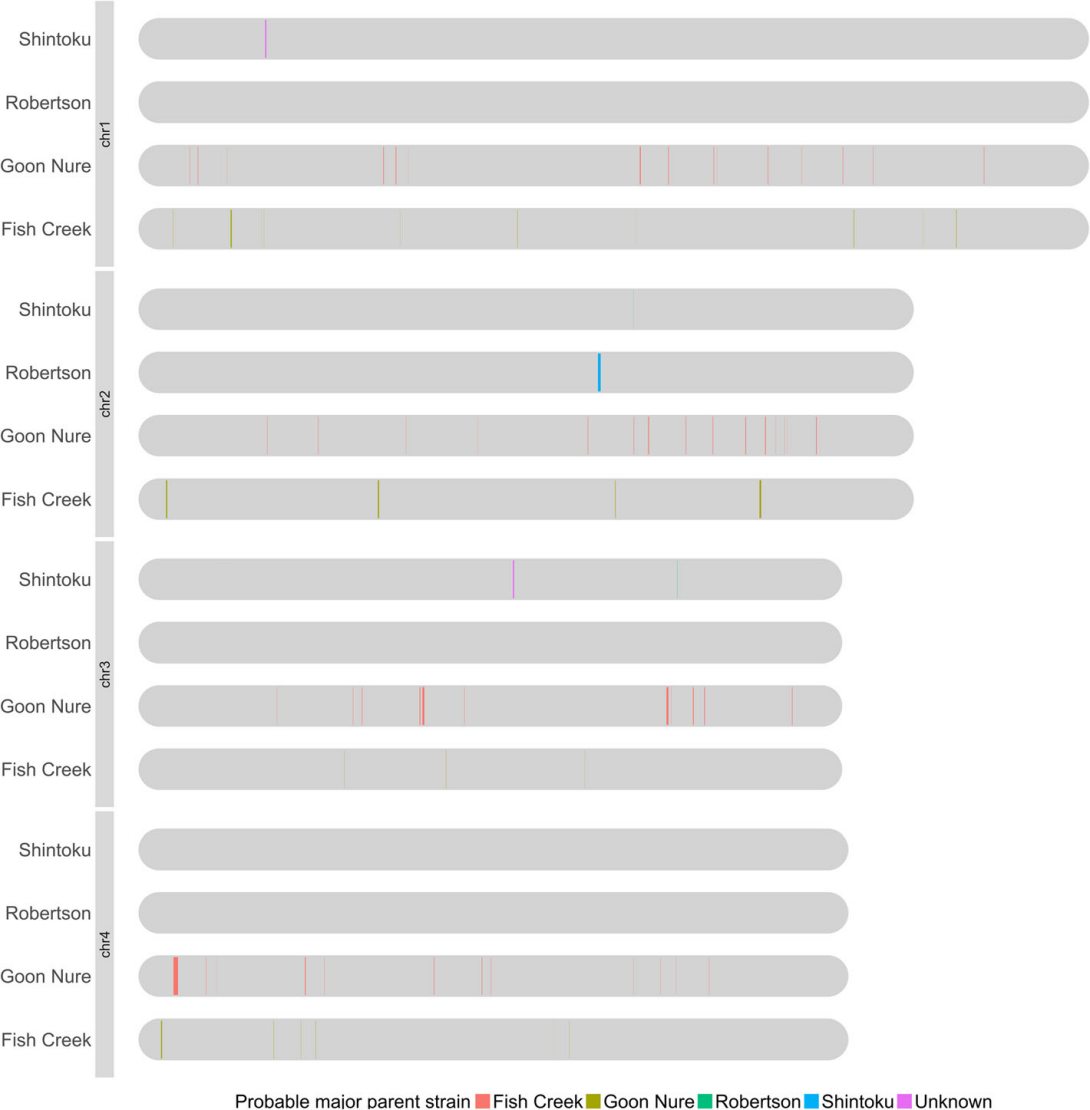
Recombination analysis of *T. orientalis* Shintoku, Ikeda, Chitose and Buffeli genotypes. In total, 83 recombination events were detected using six recombination prediction methods (Geneconv, MaxChi, RDP, SisScan, 3Seq and BootScan). Shintoku, Robertson, Fish Creek and Goon Nure chromosomes (grey) are shown aligned with probable major parent sequences shown as coloured lines. Most likely major parent sources are shown by colour (see legend)

Potential for recombination between *T. orientalis* genotypes has previously been postulated [1, 22]. To explore this, we examined the *T. orientalis* SNV datasets for recombination events as previously done with *Theileria parva* [23]. The frequencies of all 7 possible genotype combinations are shown as a Venn diagram (Fig. 1) and show high values for Fish Creek and Goon Nure containing combinations and low numbers for Robertson combinations, which reflect the genetic distance between these genotypes and the Shintoku reference. When these allele combinations were graphed against their genomic loci, little evidence of SNV clustering associated with recombination events could be observed (data not shown), potentially due to the high number of variant positions present. To further examine potential recombination, six recombination detection tests, namely Geneconv, MaxChi, Recombination Detection Program (RDP), BootScan, 3Seq and SiScan were used to analyse SNV-derived alignments. A total of 83 potential recombination events were detected in the concatenated SNV dataset (Fig. 2; detail in Additional file 9). All detected events were relatively small, with genomic sizes ranging 3–13,821 bp in size and a median of 989 bp. Furthermore, no evidence of recombination was detected between the Ikeda genotype samples (Robertson/Shintoku) and Chitose (Fish Creek) or Buffeli (Goon Nure) genotypes.

### Mitochondrial genomes of Ikeda, Chitose and Buffeli isolates

**Fig. 3:**
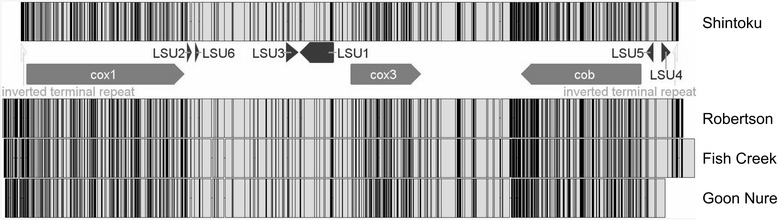
Mitochondrial genomes of *T. orientalis* Ikeda, Chitose and Buffeli. Grey areas represent areas of sequence identity; black lines represent areas of sequence divergence. The cox1, cob and cox3 genes are structurally conserved in all three genomes

*Theileria* mitochondrial genomes have been previously observed to be linear and relatively small at approximately 6 kbp in size [24]. In *T. orientalis* isolates that were examined in this study (Fig. 3), we were able to confirm a linear structure with inverted PCR utilizing outward facing primers at the edge of each mitochondrial genome. Furthermore, some *Babesia* mitochondrial genomes have been described to undergo multiple inversions [25]. To determine if this occurs in *T. orientalis* mitochondrial genomes, we examined sequencing reads mapped to assembled Robertson, Fish Creek and Goon Nure mitochondrial genomes for evidence of split-reads, i.e. reads partially mapped to the contig and soft-clipped. We found no evidence of soft-clipped reads indicative of an inversion event in any of the three *T. orientalis* mitochondrial sequences.

### dN/dS analysis

Analysis of the dN/dS ratio has the potential to inform future vaccine studies due to the link between high dN/dS and antigenic potential [23, 26]. In this study, we examined the dN/dS ratio calculated using reconstructed genomes generated from the Shintoku reference sequence and homozygous SNVs from the Fish Creek and Goon Nure genome sequences. The Robertson isolate was determined to be too closely related to the reference sequence (3377/4002 genes with < 5 mutations) and was excluded from this analysis, however, Robertson coding sequences with high numbers of variants were observed and the number of mutations per coding sequence are listed in Additional file 5. The dN/dS ratios of 3577/4002 and 3230/4002 genes were examined from Fish Creek and Goon Nure sequences (Additional file 10), while the dN/dS of predicted surface proteins (SignalP and TMHMM positive) are shown in Table 3. These dN/dS values compare well with similar studies in other *Theileria* species [23]. To further examine surface proteins of *T. orientalis* we examined extracts of purified piroplasms with mass spectrometry and were able to confirm the expression of 6 of these proteins. These included 2 hypothetical proteins (XP_009690040.1, XP_009690016.1), surface antigens MPSP (XP_009689845.1) and p23 (XP_009690580.1), a bifunctional nuclease (XP_009691620.1) and transmembrane protein 17 (XP_009690633.1).

**Table 3:** Predicted surface proteins with High dN/dS

Protein ID	Functional classification	Homolog in other piroplasmida	Pfam	Mean dN/dS
XP_009690939.1	hypothetical protein	N		0.420013
XP_009691911.1	uncharacterized protein	Y	Pf04385	0.348927
XP_009690040.1	hypothetical protein	Y		0.329911
XP_009690607.1	hypothetical protein	Y		0.326084
XP_009691340.1	hypothetical protein	Y		0.325941
XP_009689372.1	hypothetical protein	Y		0.322271
XP_009689430.1	hypothetical protein	N		0.310138
XP_009690004.1	hypothetical protein	Y		0.307715
XP_009690344.1	ToLocg1 paralog	Y		0.272704
XP_009690269.1	hypothetical protein	Y		0.236663
XP_009691913.1	hypothetical protein	N		0.230252
XP_009689555.1	hypothetical protein	Y		0.228835
XP_009689383.1	hypothetical protein	N		0.225034
XP_009688884.1	CD8+ T cell target antigen Tp2	Y		0.222906
XP_009689185.1	hypothetical protein	Y		0.222693
XP_009692799.1	hypothetical protein	N	Pf04385	0.220597
XP_009692694.1	hypothetical protein	Y		0.217668
XP_009690569.1	brain protein 44-like	Y	Pf03650	0.214623
XP_009689733.1	hypothetical protein	Y		0.211825
XP_009690910.1	hypothetical protein	N		0.210374
XP_009689120.1	hypothetical protein	Y		0.198483
XP_009692522.1	thrombospondin-related anonymous protein	Y	Pf00092	0.196277
XP_009691139.1	putative protease	Y	Pf02517	0.1867
XP_009690803.1	putative apicoplast import protein	Y	Pf16166	0.176391
XP_009692438.1	hypothetical protein	Y		0.17456
XP_009692777.1	uncharacterized protein	Y	Pf04385	0.173405
XP_009692412.1	hypothetical protein	Y		0.16334
XP_009692816.1	surface protein	Y	Pf04145	0.160208
XP_009689868.1	hypothetical protein	Y		0.156548
XP_009692665.1	hypothetical protein	N		0.154705
XP_009689845.1	major piroplasm surface protein	Y	Pf02488	0.146066
XP_009690016.1	hypothetical protein	Y	Pf04385	0.136429
XP_009692660.1	putative lysophospholipase	Y	Pf12146	0.129336
XP_009692759.1	hypothetical protein	Y		0.127301
XP_009691480.1	putative ER oxidoreductin	Y	Pf04137	0.120148
XP_009689697.1	uncharacterized protein	Y		0.116339
XP_009691437.1	hypothetical protein	Y		0.113952
XP_009688850.1	hypothetical protein	Y		0.113259
XP_009690130.1	hypothetical protein	Y	Pf08320	0.1113
XP_009692225.1	uncharacterized protein	Y	Pf07691	0.107945
XP_009690580.1	p23 surface protein	Y		0.104459
XP_009691349.1	zinc transport protein	Y	Pf02535	0.10384
XP_009689509.1	hypothetical protein	Y		0.10243
XP_009689195.1	hypothetical protein	Y		0.102029
XP_009691696.1	hypothetical protein	Y		0.10029
XP_009689770.1	uncharacterized protein	Y	Pf04385	0.099851
XP_009690399.1	50S ribosomal protein L9	Y	Pf01281	0.096548
XP_009689290.1	hypothetical protein	Y	Pf05450	0.095706
XP_009689754.1	uncharacterized protein	Y		0.086991
XP_009688894.1	50S ribosomal protein L33	Y		0.0863757

### MLST phylogeny of Ikeda, Chitose and Buffeli and their place within the Piroplasmida

**Fig. 4:**
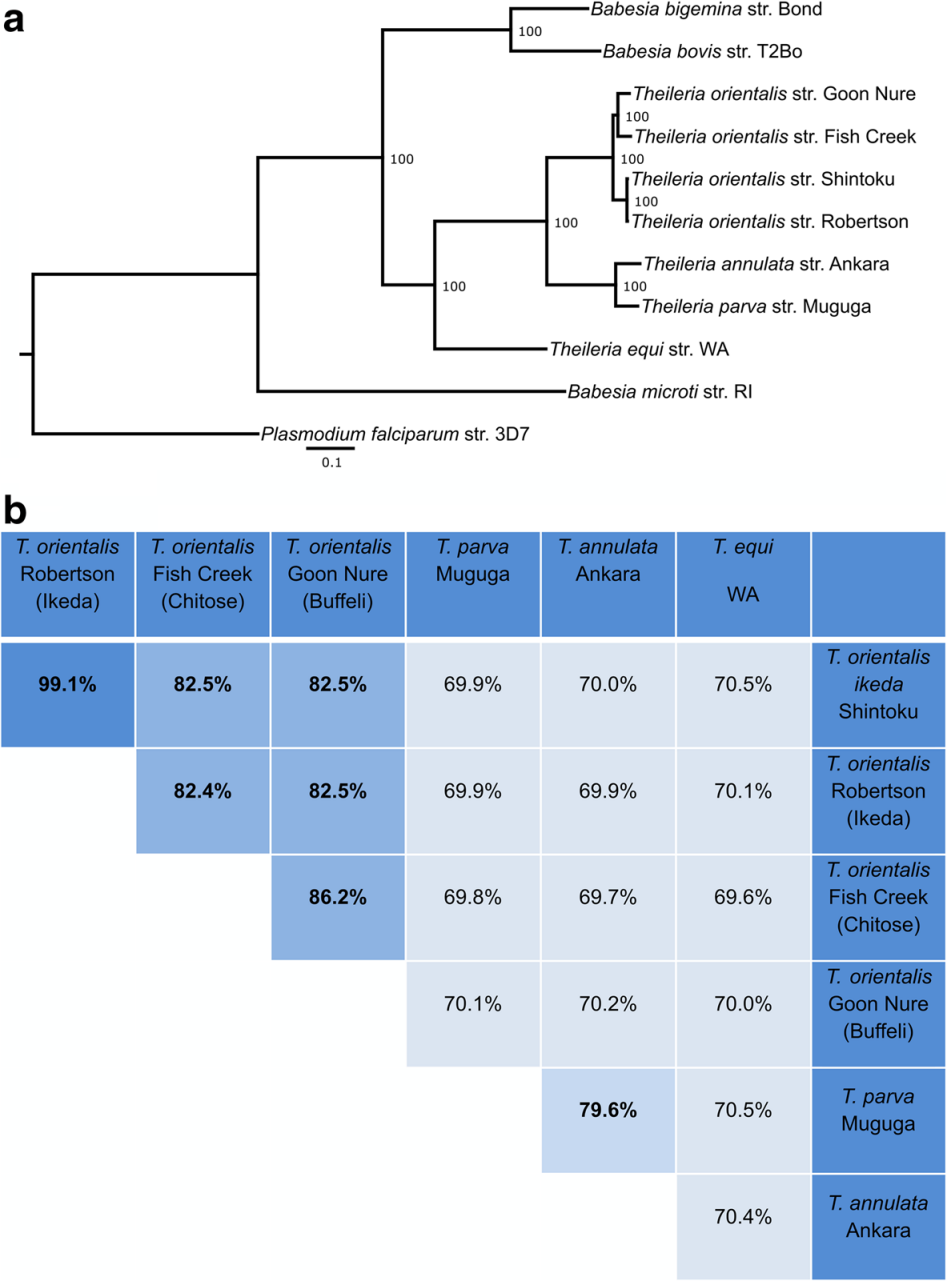
**a**. Multilocus phylogeny of Piroplasmida whole genome sequences. The tree was inferred by maximum-likelihood using an alignment of 654 concatenated protein sequences from single copy genes. *Plasmodium falciparum* str. 3D7 was included as an outgroup. Labels indicated bootstrap support (%). **b**. Average nucleotide identities between genomes of the *Theileria* genus as calculated by the method described in [92]

To assess if whole genome sequences could be used to further refine the taxonomy of *T. orientalis,* we explored two commonly used methods of species definition, phylogeny and average nucleotide identity (ANI), using *T. orientalis* whole genome assemblies and representative whole genome sequences of the Order Piroplasmida (Fig. 4). Support values based on bootstrap analysis show very high support for all branches. Both whole genome phylogeny and ANI reveal a very close relationship between Shintoku and Robertson genomes. In contrast, ANIs between strains Robertson/Shintoku and Fish Creek/Goon Nure are low for organisms considered to be of the same species and compare well with those observed between *T. parva* and *T. annulata* (Fig. 4b). ANI clustering was also used to compare species of the *Plasmodium* genus (Additional file 11). For comparison, murine species *Plasmodium berghei*, *Plasmodium yoelii, Plasmodium vinckei,* and *Plasmodium chabaudi* have pairwise ANI that range from 83.5–90.1%.

## Discussion

This study presents the first genomic analysis of Australian isolates of *T. orientalis* and the first published genomic sequences of Chitose and Buffeli types. Genomic studies allow for multi-locus strategies to define evolutionary relationships with greater confidence than those that rely on single genes. In the Order Piroplasmida, the placement of species such as *Theileria equi* and *Babesia microti,* has been greatly clarified using these techniques [17, 27]. The taxonomy of *T. orientalis* has been controversial [28] and originally *T. orientalis*, *T. sergenti* and *T. buffeli* were all used, in a regionally-specific manner, to describe this organism. Researchers in early molecular studies that originally attempted to identify differences that could separate morphologically indistinguishable *T. orientalis, T. sergenti* and *T. buffeli* instead determined that these “species” consisted of multiple common genotypes that often had little correlation with these definitions [29–31]. The result of these has led to the general use of *T. orientalis* in the literature to describe this group with differences in MPSP used to define 11 recognized genotypes [1, 7, 8]. However, the organism is often referred to as *T. sergenti, T. buffeli*, and the *T. orientalis/buffeli* group and hence its taxonomy requires further clarification [32–34].

Examination of the relationship between Shintoku/Robertson (Ikeda), Fish Creek (Chitose) and Goon Nure (Buffeli) isolates using the multi-locus strategy in this study reveals a similar structure to previous phylogenies [22, 29]. These phylogenetic analyses based on MPSP consistently show a clear separation between Chitose/Buffeli sequences, which cluster with Types 4, 5, 8 and N3, and Ikeda, which clusters with Type 7 [1, 8, 22, 35, 36]. Furthermore, Chitose/Buffeli and Ikeda isolates had comparable ANIs to *T. parva* and *T. annulata* (Fig. 4b). Moreover, prediction of recombination in these isolates identified putative recombinant/major parent pairings within Shintoku/Robertson and Fish Creek/Goon Nure isolates but not between these groups. Alpha-taxonomy continues to be an area of uncertainty in protists as no generally accepted basis for delimiting species exists. Instead, species designations are determined on a case-by-case basis and based on a combination of observed phenotypic and genotypic evidence [37], with pathogenicity considered a key phenotypic delineator [38]. Based on the genotypic differences observed here, previous MPSP-based phylogenetic studies and observations of pathogenicity of the Ikeda genotype [9–11, 22, 39, 40], disease-associated Ikeda and closely related Type 7 could potentially be considered a separate species from non-pathogenic Chitose, Buffeli and related Types 4, 5, 8 and N3. Furthermore, the genomic divergence observed between Chitose and Buffeli isolates (86.5%) compare well to the ANI range of murine *Plasmodium* spp. (83.5–90.1%), indicating that a subspecies classification may also be appropriate for these organisms. Future studies focusing on the genomics of Types 4–8 and N1–3 as well as globally distributed isolates of Ikeda, Chitose and Buffeli would help resolve further confusion surrounding the taxonomy of this organism.

Previous studies have hypothesized that recombination between *T. orientalis* genotypes is unlikely [1]. Other studies have observed mosaic sequences in the *T. orientalis* group are potentially indicative of recombination, however, these sequences could also be products of cloned PCR chimeras [22, 41]. In *Theileria parva* sourced from cattle, recombination has been identified using statistical analysis of concatenated SNV alignments [23]. However, recombination was not detected between cattle- and buffalo-sourced sequences. Furthermore, these statistical techniques are potentially susceptible to false positives. Using these methods, we have detected evidence of recombination in short sequences between Fish Creek and Goon Nure strains and, to a lesser extent, between the Shintoku and Robertson strains. This is particularly notable as the Fish Creek and Goon Nure sequences have a much lower average nucleotide identity than the *T. parva* sequence group. Hayashida et al. postulate that the lack of observed recombination between cattle and buffalo *T. parva* is likely caused by isolation due to host adaptation [23]. Such isolation is not observed in *T. orientalis* isolates studied here, which are all cattle-adapted and prevalent in Eastern Asia and Australia [11, 22]. Identification of recombination limits in *Theileria* will require further comparative genomics of globally distributed genotypes.

Identification of *T. orientalis* Ikeda in Australian herds was confirmed in 2006 and it has since become endemic to the south-east, closely matching the distribution of the recognized vector *Haemaphyalis longicornis* [42]. The original source of *T. orientalis* Ikeda infections in Australia has been difficult to identify. In response to outbreaks of bovine spongiform encephalopathy, the importation of live cattle from Japan was banned in 2001. However, prior to this, Japanese-sourced, live Wagyu cattle were reportedly imported into Australia via the USA [43] to establish the Wagyu genetic line for breeding purposes, and if these imports occurred they could have been a source of introduction. An alternative source of introduction could be migratory birds. Several bird species migrate to Australia from north-eastern Asia during the northern hemisphere winter and *H. longicornis* has been found on migratory birds from these areas [44]. While infection of birds with *T. orientalis* is unlikely, they may play a role in the transport of infected ticks to new areas [45]. Additionally, the potential for transstadial transmission may provide a mechanism for birds to transport tick-borne apicomplexan parasites over long distances [46].

High dN/dS predicted surface proteins that diverged greatly between the pathogenic and apathogenic species included multiple uncharacterized and hypothetical proteins, 7 of which are unique to *T. orientalis*. The Frequently Associated In Theileria (FAINT) domain (Pf04385) shows high representation in these proteins as indicated previously [15]. Other noteworthy proteins include homologs to antigen Tp2, identified in *T. parva* as an immunodominant T-cell antigen [47]; thrombospondin-related anonymous protein, recognized as an adhesin in several *Plasmodium* and *Babesia* species [48–52]; and MPSP and P23 surface proteins, which are highly expressed surface antigens in *T. orientalis* that have been shown to bind heparin and, in the case of the MPSP, also shown to bind bovine erythrocytes [53, 54]. Partial protection and reduced clinical symptoms have been demonstrated using subunit vaccines generated from whole or immunogenic portions of the MPSP sequence [30]. Further vaccine studies using data from this study (Table 3) may inform strategies for future *Theileria* disease outbreaks.

## Methods

### Samples

Samples were sourced from cattle testing PCR positive for a single *T. orientalis* genotype based on the major piroplasm surface protein sequence (ie. Ikeda, Chitose or Buffeli) and were collected by private or district veterinarians as part of routine clinical monitoring. The isolates were sourced from cattle in Robertson, New South Wales (Collected 2009, breed: Angus), Fish Creek in Southern Victoria (Collected 2014, breed: Friesian) and Goon Nure in East Gippsland, Victoria (Collected 2012, breed: Freisian) respectively and named according to their location of isolation. Confirmation that each isolate was of a single genotype was performed with two separate PCR assays [10, 40]. Two to three days following the initial bleed, approximately 80 mL of blood was collected into anticoagulant (heparin or citrate) and shipped to a laboratory cold for propagation (1 passage) and extraction. On arrival samples were mixed with cryopreservative and stored as stabilates at − 80 °C until required.

### Propagation and purification of Theileria piroplasms

This research was carried out in accordance with the Australian Code of Practice for the Care and Use of Animals for Scientific Purposes at the Tick Fever Centre, Wacol, Queensland. To propagate *Theileria* piroplasms, individual splenectomised calves were inoculated with stabilate generated from Robertson, Fish Creek and Goon Nure strains. Blood samples were drawn from each calf at regular intervals to monitor the infection and Giemsa-stained blood smears were used to estimate the level of parasitaemia. The packed cell volume (PCV) was also monitored. When parasitaemia had reached an appropriate level (6–20%), approximately 3.5 L of calf blood was collected into anticoagulant (CPDA1 or heparin). The blood was transferred into 300–500 mL blood bags using a dialysis pump and subsequently passed through Terumo leukocyte filters (300 mL blood per filter) under gravity. Leukocyte-depleted blood was then transferred to centrifuge tubes, centrifuged at 2500×*g* for 20 min and the serum and any remaining buffy coat removed. The remaining erythrocytes were washed 3 × with Dulbecco’s phosphate-buffered saline (D-PBS) followed by centrifugation as described above and diluted 5 × with D-PBS and loaded into a cell disruption vessel (“nitrogen bomb”). The erythrocytes were lysed and the parasite harvested by differential centrifugation as described previously [55]. Briefly, the vessel was infused with nitrogen gas to a pressure of 1000 psi for 1 min and then the pressure was released. The lysed erythrocytes were collected into a clean vessel and then transferred to centrifuge tubes. The cell lysate was centrifuged at 670×*g* for 10 min to pellet the red blood cell debris. The supernatant was then harvested and centrifuged at 2700×*g* for 10 min to pellet the piroplasms. The piroplasms were resuspended in D-PBS and a smear prepared from the suspension, which was stained with Giemsa stain to check purity. The piroplasms were then aliquoted into microfuge tubes and centrifuged at 2700×*g* for 10 min. The supernatant was removed and the piroplasm pellets stored at − 80 °C. Piroplasm preparations were transferred on dry ice and maintained at − 80 °C thereafter.

### Nucleic acid extraction and whole genome sequencing

Piroplasm pellets were resuspended in 200 μL of phosphate-buffered saline and DNA was extracted using the DNeasy Blood and Tissue kit (Qiagen) according to the manufacturer’s instructions. For each sample, tagmentation of genomic DNA, and PCR amplification of tagged DNA were performed in triplicate using the Nextera system (Illumina). Sequencing libraries were pooled and normalized using bead size selection (SPRI beads, Beckman Coulter). The Agilent 2100 Bioanalyzer, with the High Sensitivity DNA kit was used to quantitate the pooled library before loading onto the Illumina platforms (Miseq or HiSeq). Paired-end 250 nt reads were generated using MiSeq V2 chemistry and paired-end 150 nt reads were generated using the HiSeq2500 system.

### Protein extraction, electrophoresis and mass spectrometry

From approximately 0.1 g of *T. orientalis* Ikeda (Robertson strain) pelleted piroplasms, proteins were extracted, enriched, excised from 1-D sodium dodecyl sulfate polyacrylamide gel electrophoresis (SDS-PAGE) gels and prepared for mass spectrometry as previously described [56]. Briefly, proteins were separated by SDS-PAGE, stained, excised from the gel lane and separated into 16 slices. Each gel slice was then diced into ~ 1 mm cubes, stripped of dye, washed and digested in-gel with trypsin for analysis. Protein identification was performed by liquid chromatography with tandem mass spectrometry (LC-MS/MS) using a QSTAR Elite hybrid quadrupole time-of-flight mass spectrometer (AB Sciex). MS/MS data files were analysed using Mascot (provided by the Australian Proteomics Computational Facility, hosted by the Walter and Eliza Hall Institute for Medical Research Systems Biology Mascot Server) against the non-redundant MSPnr100 database compiled from all known reference protein sequences including NCBI, Refseq, UniProt, EuPathDB and Ensembl. Peptides matches at *p* < 0.05 (Ion score > 60) were classified as hits.

### Assembly and annotation

Genome assembly was achieved with raw reads using the A5-miseq (v2015–05-22) pipeline [57]. Levels of remaining host DNA were assessed and filtered using BioBloomTools [58] and the *B. taurus* genome sequence [59]. Whole genome alignments were performed using progressiveMauve from Mauve v2.4.0 and nucmer from MUMmer v3.23 [60, 61]. For analysis of gene presence/absence, genes were annotated using Maker v3.00 beta [62] to combine the annotation tracks derived from expressed sequence tag (EST) and protein alignments and ab initio gene predictors. Firstly, repeat regions of the Robertson, Fish Creek and Goon Nure strain genome assemblies were masked using RepeatMasker v4.0.7. With repeat masked sequences, ab initio annotation tracks were derived from gene predictors Augustus v3.2.3 [63], Snap v2013–11-29 [64], and GeneMark ES Suite v4.32 [65] utilizing both GeneMark ET (Robertson and Fish Creek), and GeneMark ES (Goon Nure). A training set based on EST alignments (RNA data sourced from the Shintoku WGS project [15]) and transcoding regions was generated by PASA v2.1.0 [66] and used to train all ab initio predictors except GeneMark ES. Alignment of EST and protein evidence was performed with Exonerate v2.2.0 [67] using the coding2genome and protein2genome models respectively. Maker was run using the trained gene predictors and performed EST and protein alignments. Following annotation each independent gene track was assessed using Evidence Modeler v1.1.1 [66] to generate the final annotation with the following weights applied (EST: coding2genome = 7, blastn = 2, tblastx = 2. Protein: protein2genome = 10, blastx = 2. Ab initio: augustus = 2, snap = 1, genemark = 1). Additionally, annotations were manually curated by visual inspection and comparison to those transferred by RATT [68] for further quality checking. All parameter files used by Maker and Evidence Modeler to generate each annotation are included as Additional files 12, 13 and 14. Functional annotation was performed with Blast2GO v4.1.9 [69]. Predicted protein sequences were examined with blastp-fast (e-value cutoff 1e-5) against a local version of the non-redundant protein database (downloaded 2017–07-01). From blastp results, a gene ontology (GO) annotation was generated and extended through merging with results from InterProScan [70]. Finally, GO annotations were extended with ANNEX [71].

### Ortholog clustering and gene presence analysis

Ortholog clustering of translated proteins from annotated draft genomes was performed with Orthofinder v2.1.2 [21] using 10 whole genome sequences from species representing the Piroplasmida order and one outgroup species *Plasmodium falciparum* strain 3D7. The 10 genomes include the three *T. orientalis* isolates examined in this study and previously published and annotated whole genome sequences of *T. orientalis¸ Theileria parva*, *Theileria annulata*, *Theileria* (formerly *Babesia*) *equi*, *Babesia bovis*, *Babesia bigemina* and *Babesia microti* [15–18, 27, 72–74]. For characterisation of the presence of *T. orientalis* genes in sequences generated by this study and absence in the Shintoku genome sequence, translated Robertson, Fish Creek and Goon Nure proteins were scanned against translated *T. orientalis* Shintoku proteins using blastp (e-value cutoff 1e-5). Robertson, Fish Creek and Goon Nure proteins that did not match any *T. orientalis* Shintoku proteins were further examined using tblastn (e-value cutoff 1e-5) of the *T. orientalis* Shintoku genome sequence. All proteins that did not match Shintoku sequences were further examined using blastp and the non-redundant protein database (2018–01–30).

### Variant calling and validation

For variant calling, reads were first trimmed with Trimmomatic v0.33 based on leading and trailing base quality (Q < 20) and a 4-base sliding window when average quality scores were less than 20 [75]. Read quality was assessed pre- and post-trimming with FastQC (https://www.bioinformatics.babraham.ac.uk/projects/fastqc/) and sequence files were manipulated using ngs-utils v0.5.7 [76]. For between population variants, trimmed reads were mapped to the *T. orientalis* Shintoku reference sequence [15] with NextGenMap v0.4.12 [77]. Low quality mapped reads (Q < 10) were filtered using samtools v1.2 [78] and alignments were subsequently sorted, and duplicates removed using picard tools v1.138 (http://broadinstitute.github.io/picard/). Variant calling was performed using VarScan v2.3.8 [20] on mpileup files generated by samtools. Variant calling parameters were based on previous studies into apicomplexan SNV detection and variant calling algorithm comparisons [79, 80]. Specifically, variants were called using a minimum coverage of 14 × [79], minimum average quality of 20, minimum variant supporting reads of 4, minimum variant frequency of 0.01 and minimum *p*-value threshold of 0.05. A minimum frequency of 0.9 was used to define homozygous variants. Variant quality was assessed by examining subsets of variant sequences with Sanger sequencing and by using simulated alignments. Primers for Sanger sequencing experiments are shown in Additional file 15. Sanger sequencing was performed on PCR products at the Australian Genome Research Facility. For simulated alignments, the *T. orientalis* Shintoku sequence was modified by the msbar program of EMBOSS [81] to contain approximately 90,000 point, codon and block insertion/deletion events and 900,000 substitution events. Simulated reads were generated from simulated genomes using ART vMountRainier 2016–06-05 [82] and mixed into one fastq file. False discovery rate was calculated by aligning simulated to experimentally determined sequences and comparing true and detected variants.

### Recombination and selection analysis

Recombination analysis was performed on concatenated SNV alignments to allow direct comparison to previous attempts at examining recombination in *Theileria* spp. [23]. These methods are potentially susceptible to error from highly divergent sequences (< 60% identity) and reduced SNV density information. To limit false positive results, we performed recombination analysis using six recombination tests, namely Geneconv, MaxChi, RDP, BootScan, 3Seq and SiScan as previously described with the exception that, due to higher SNV density, SNVs were not discounted if they were within 100 bp of another SNV [23]. For dN/dS analysis, SNV-containing genomes were constructed using homogeneous SNVs from Fish Creek and Goon Nure strain variant analysis with the FastaAlternateReferenceMaker tool from the Genome Analysis Toolkit v3.2.2 [83]. Coding sequence alignments of Reference and SNV-containing genomes were generated for gene regions of 14 × or greater sequencing coverage using customised python scripts and biopython [84]. Coding DNA sequences (CDS) were excluded where sequencing coverage at > 14 × made up less than 50% of the CDS. dN/dS ratios and synonymous/non-synonymous mutations were calculated for coding sequences using KaKs Calculator v2.0 [85]. Predicted surface proteins were identified using SignalP 4.0 [86] and TMHMM [87].

### Piroplasmida phylogenomic analysis

Piroplasmida phylogeny was examined using single copy orthogroups identified during ortholog clustering (outlined above). A total of 654 orthologous groups with single copy genes found in all 11 genomes were identified using Orthofinder v2.1.2 [21], with initial search results filtered to retain only groups with an e-value <1e-30. Sequences from each of the 654 orthologous groups were aligned and trimmed using MAFFT v7.310 and trimAl v1.3 [88, 89] as described previously [27] with the following alterations. Sequence alignments were trimmed using the nogaps automated option and two orthologous groups were removed from the final analysis after trimming due to poor alignment and high trimming. A supermatrix alignment (261,036 residues) was generated by concatenating individual gene alignments using a customised python script. Protein evolution model selection was performed for each gene of the supermatrix alignment using Prottest v3.4 [90]. The supermatrix tree was inferred with RAxML v8.2.4 as previously described [27, 91]. Average nucleotide identity comparisons were calculated using pyani v0.2.3 (https://github.com/widdowquinn/pyani) using the ANIb method described in [92].

## Conclusion

The draft genomes generated in this study from three genotypes of *T. orientalis* allowed us to provide the first phylogenomic evidence for species-level differences between the pathogenic Ikeda and apathogenic Chitose and Buffeli genotypes. Additionally, significant differences between the Chitose and Buffeli genotypes were equivalent to those observed between murine *Plasmodium* spp. indicating that a subspecies classification may be appropriate for those genotypes. Very high average nucleotide identity between an Australian (Robertson) and a Japanese (Shintoku) Ikeda isolate was also observed. Genomewide analysis of variation used in this study should be expanded to include additional strains to elucidate the origin of *T. orientalis* Ikeda infection in Australia and support future vaccine development.

## Additional files

Additional file 1: Mauve alignments with reference genome. Reference alignments between *T. orientalis* Shintoku whole genome sequence and Robertson (top), Fish Creek (middle) and Goon Nure (bottom) strain sequences. Alignments were generated by Mauve. Coloured blocks represent locally collinear blocks (LCBs) which are conserved segments determined to be internally free from genome rearrangements. Lines connecting top sequence to bottom demonstrate aligned LCBs. All alignments are shown with Shintoku as the upper sequence and Australian isolate as the lower. Alignment width is determined by the longest sequence, white sections represent sequence which does not align to Shintoku with Mauve, but may also represent duplicated sequence. (TIF 2219 kb)

Additional file 2: Dot plots of nucmer alignments. Dot plots of Robertson, Fish Creek and Goon Nure sequences against the Shintoku reference sequence (A) and self alignment (B). Alignments were generated using nucmer. Reference alignments represent longest mutually consistent set (delta-filter -g), self alignments include all additional matches > 50 bp in length and 75% identify (delta-filter -i 75 -l 50). Purple lines represent primary or highest scoring matches, blue lines represent additional matches. (TIF 9120 kb)

Additional file 3: Validation of SNV variant calling pipeline. Validation statistics of SNV calling pipeline generated by comparison to short sections of Sanger sequencing. (DOC 32 kb)

Additional file 4: SNV density vs genomic loci histograms. SNV density vs genomic loci histograms of the Robertson (top), Fish Creek (middle) and Goon Nure bottom) strains. Y-axis represents number of SNV per 10 kb axis represents whole genome position, chromosomes are represented by colour. (TIF 846 kb)

Additional file 5: SNVs per open reading frame. Number of SNVs found in each gene of the Robertson isolate when mapped to the reference Shintoku genome. (PDF 203 kb)

Additional file 6: Orthologous genes vs orthologous groups. Number of orthologous genes against orthologous groups containing x number of genes per isolate. (TIF 1293 kb)

Additional file 7: Coding sequence average depth of coverage. Average coverage depth of coding sequences for orthogroups grouped by genes per isolate (Gene numbers containing > 4 orthogroups not shown). (DOC 29 kb)

Additional file 8: Proteins with no blastp matches compared to reference genome. Examination of proteins that produced no blastp match to the Shintoku predicted proteome, by blastp searches of the nr database. (XLSX 13 kb)

Additional file 9: Predicted recombination events. Details of predicted recombination events shown in Fig. 2. (PDF 30 kb)

Additional file 10: dN/dS ratios. Calculated dN/dS (a.k.a. KaKs) ratios for the predicted Shintoku proteome by comparison to mapped Fish Creek (Chitose) and Goon Nure (Buffeli) isolate sequences. (PDF 913 kb)

Additional file 11: Average nucleotide identity (ANI) for members of the *Plasmodium* genus. Pairwise ANI values for species of the *Plasmodium* genus with publically-available genome sequences. (XLSX 11 kb)

Additional file 12: Fish Creek maker parameters. Parameters used for the annotation of the Fish Creek genome sequence using the maker pipeline. (GZ 3 kb)

Additional file 13: Goon Nure maker parameters. Parameters used for the annotation of the Goon Nure genome sequence using the maker pipeline. (GZ 3 kb)

Additional file 14: Robertson maker parameters. Parameters used for the annotation of the Robertson genome sequence using the maker pipeline. (GZ 3 kb)

Additional file 15: Primers used in this study. Primers used for Sanger sequencing validation of the SNV calling pipeline. (DOC 44 kb)

## Abbreviations

ANI: Average nucelotide identity
CDS: Coding DNA sequence
dN/dS: Ratio of non-synonymous/Synonymous mutations per site
D-PBS: Dulbecco’s phosphate-buffered saline
EST: Expressed sequence tag
FAINT: Frequently associated IN Theileria
GO: Gene ontology
LC-MS/MS: Liquid chromatography - tandem mass spectrometry
MPSP: Major piroplasm surface protein
PCV: Packed cell volume
RDP: Recombination detection program
SDS-PAGE: Sodium dodecyl sulfate - polyAcrylamide gel electrophoresis
SNV: Single nucleotide variant
