# Supplementary figures and images for: Analysis of Theileria orientalis draft genome sequences reveals potential species-level divergence of the Ikeda, Chitose and Buffeli genotypes
Source: BMC Genomics. 2018 Apr 27;19:298. doi: 10.1186/s12864-018-4701-2 (PMC5921998; doi:10.1186/s12864-018-4701-2)

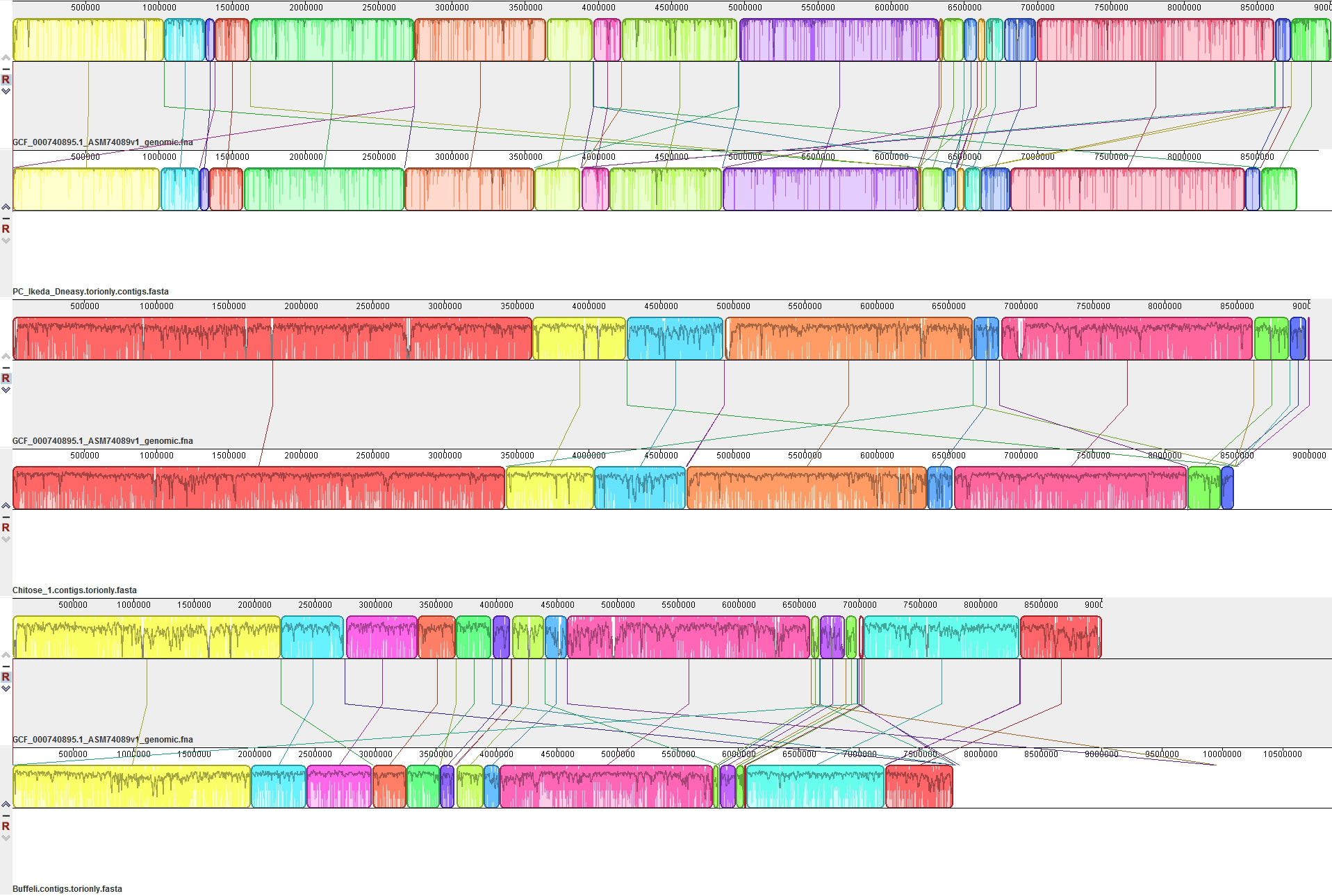

Supplement: Supplementary file 1 — Mauve alignments with reference genome. Reference alignments between T. orientalis Shintoku whole genome sequence and Robertson (top), Fish Creek (middle) and Goon Nure (bottom) strain sequences. Alignments were generated by Mauve. Coloured blocks represent locally collinear blocks (LCBs) which are conserved segments determined to be internally free from genome rearrangements. Lines connecting top sequence to bottom demonstrate aligned LCBs. All alignments are shown with Shintoku as the upper sequence and Australian isolate as the lower. Alignment width is determined by the longest sequence, white sections represent sequence which does not align to Shintoku with Mauve, but may also represent duplicated sequence. (TIF 2219 kb) [file 12864_2018_4701_MOESM1_ESM.tif]

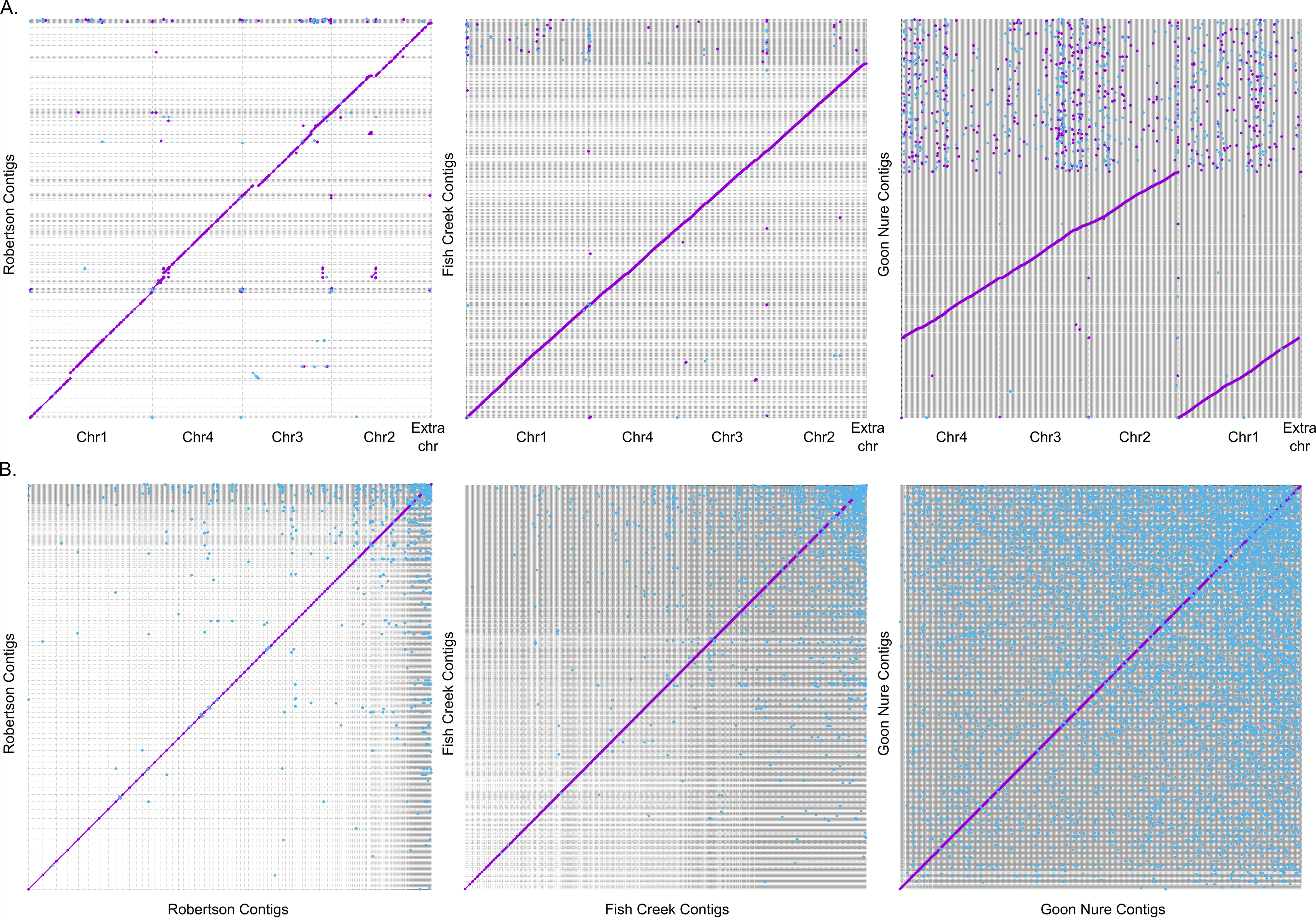

Supplement: Supplementary file 2 — Dot plots of nucmer alignments. Dot plots of Robertson, Fish Creek and Goon Nure sequences against the Shintoku reference sequence (A) and self alignment (B). Alignments were generated using nucmer. Reference alignments represent longest mutually consistent set (delta-filter -g), self alignments include all additional matches > 50 bp in length and 75% identify (delta-filter -i 75 -l 50). Purple lines represent primary or highest scoring matches, blue lines represent additional matches. (TIF 9120 kb) [file 12864_2018_4701_MOESM2_ESM.tif]

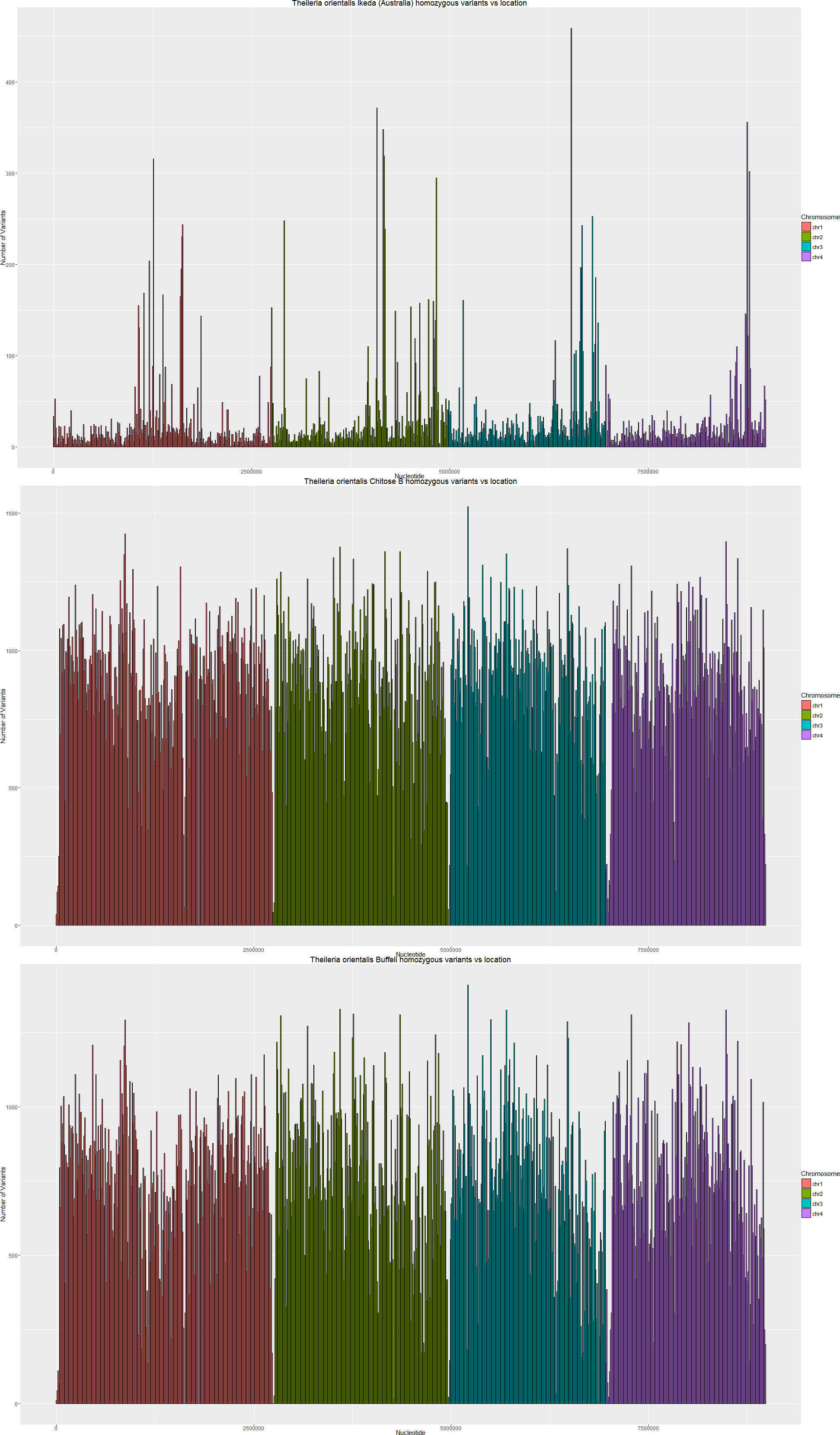

Supplement: Supplementary file 4 — SNV density vs genomic loci histograms. SNV density vs genomic loci histograms of the Robertson (top), Fish Creek (middle) and Goon Nure bottom) strains. Y-axis represents number of SNV per 10 kb axis represents whole genome position, chromosomes are represented by colour. (TIF 846 kb) [file 12864_2018_4701_MOESM4_ESM.tif]

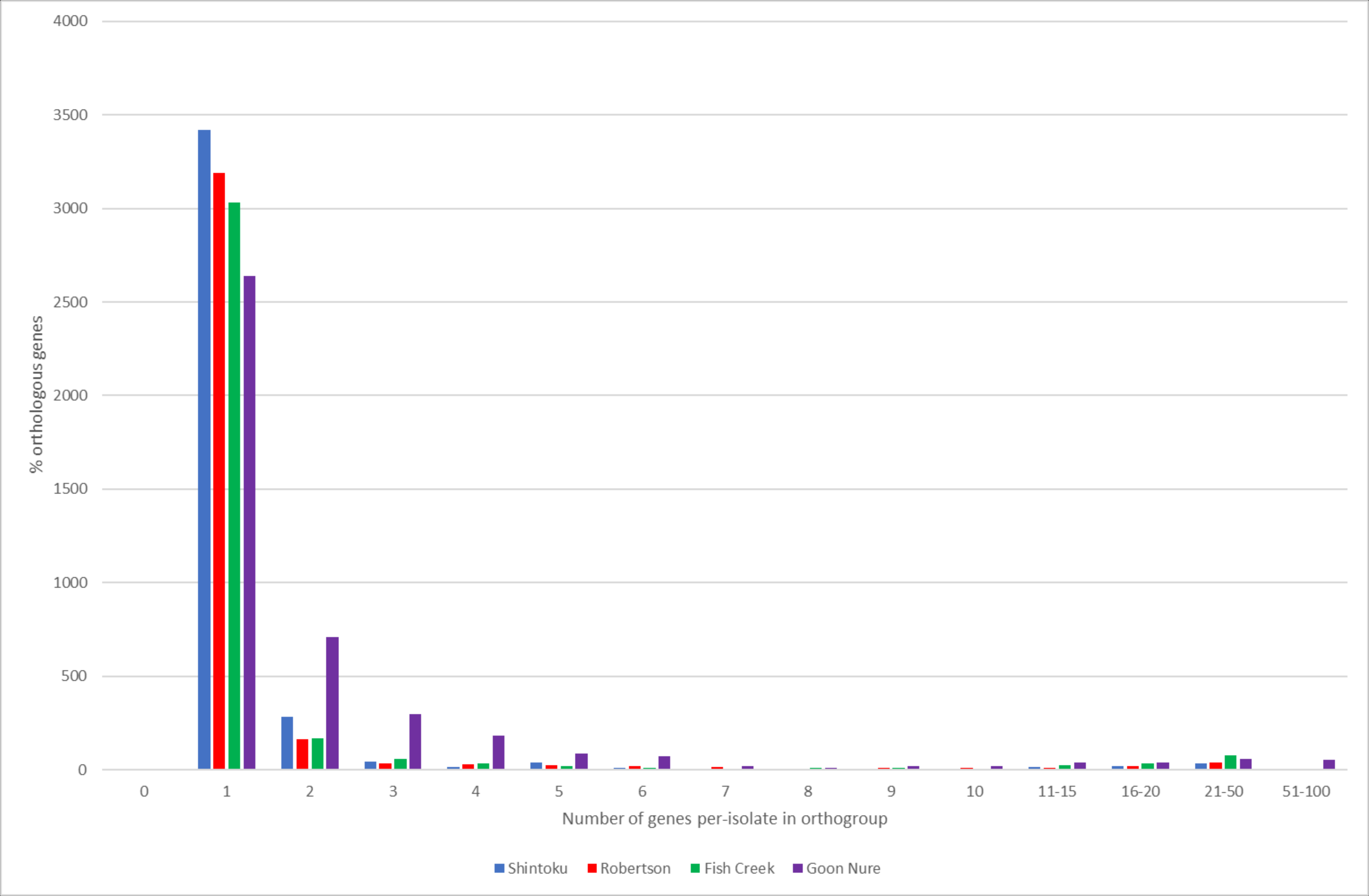

Supplement: Supplementary file 6 — Orthologous genes vs orthologous groups. Number of orthologous genes against orthologous groups containing x number of genes per isolate. (TIF 1293 kb) [file 12864_2018_4701_MOESM6_ESM.tif]
