# Supplementary material for: Analysis of Theileria orientalis draft genome sequences reveals potential species-level divergence of the Ikeda, Chitose and Buffeli genotypes
Source: BMC Genomics. 2018 Apr 27;19:298. doi: 10.1186/s12864-018-4701-2 (PMC5921998; doi:10.1186/s12864-018-4701-2)
Supplement: Supplementary file 3 — Validation of SNV variant calling pipeline. Validation statistics of SNV calling pipeline generated by comparison to short sections of Sanger sequencing. (DOC 32 kb) [file 12864_2018_4701_MOESM3_ESM.doc]

Additional File 3. Validation of SNV variant calling pipeline

|  | Strain | NGS SNP call/Sanger SNV call | | | | Sensitivity |
| --- | --- | --- | --- | --- | --- | --- |
|  |  |  |  |  |  |  |
|  |  | +/+ | +/- | -/+ | -/- |  |
|  |  |  |  |  |  |  |
|  | Robertson | 116 | 0 | 1 | 6953 | 0.991 |
|  | Fish Creek | 515 | 0 | 64 | 3652 | 0.889 |
|  | Goon Nure | 399 | 0 | 72 | 2852 | 0.847 |
|  |  |  |  |  |  |  |
