# Supplementary material for: Analysis of Theileria orientalis draft genome sequences reveals potential species-level divergence of the Ikeda, Chitose and Buffeli genotypes
Source: BMC Genomics. 2018 Apr 27;19:298. doi: 10.1186/s12864-018-4701-2 (PMC5921998; doi:10.1186/s12864-018-4701-2)
Supplement: Supplementary file 5 — SNVs per open reading frame. Number of SNVs found in each gene of the Robertson isolate when mapped to the reference Shintoku genome. (PDF 203 kb) [file 12864_2018_4701_MOESM5_ESM.pdf]

protein_id	function	number_of_SNPs
XP_009690723.1	conserved hypothetical protein	579
XP_009691742.1	uncharacterized protein	439
XP_009690688.1	conserved hypothetical protein	312
XP_009690725.1	conserved hypothetical protein	298
XP_009689372.1	conserved hypothetical protein	252
XP_009690985.1	5'-3' exonuclease	214
XP_009689354.1	uncharacterized protein	194
XP_009691801.1	hypothetical protein	181
XP_009690882.1	uncharacterized protein	173
XP_009691853.1	hypothetical protein	157
XP_009691126.1	conserved hypothetical protein	151
XP_009689498.1	hypothetical protein	141
XP_009692724.1	hypothetical protein	137
XP_009691870.1	conserved hypothetical protein	133
XP_009690948.1	conserved hypothetical protein	129
XP_009690864.1	uncharacterized protein	125
XP_009691879.1	conserved hypothetical protein	122
XP_009689418.1	conserved hypothetical protein	113
XP_009692745.1	uncharacterized protein	110
XP_009692680.1	conserved hypothetical protein	110
XP_009689411.1	hypothetical protein	107
XP_009692729.1	conserved hypothetical protein	107
XP_009689503.1	hypothetical protein	103
XP_009689500.1	conserved hypothetical protein	102
XP_009690903.1	uncharacterized protein	100
XP_009690788.1	conserved hypothetical protein	99
XP_009690848.1	uncharacterized protein	98
XP_009689605.1	conserved hypothetical protein	93
XP_009689292.1	uncharacterized protein	89
XP_009690029.1	conserved hypothetical protein	81
XP_009691790.1	hypothetical protein	81
XP_009691758.1	conserved hypothetical protein	80
XP_009691791.1	conserved hypothetical protein	79
XP_009690634.1	conserved hypothetical protein	72
XP_009689494.1	conserved hypothetical protein	72
XP_009690983.1	uncharacterized protein	70
XP_009690252.1	conserved hypothetical protein	68
XP_009692748.1	conserved hypothetical protein	67

XP_009689502.1	hypothetical protein	66
XP_009690987.1	conserved hypothetical protein	66
XP_009690901.1	uncharacterized protein	66
XP_009689298.1	conserved hypothetical protein	65
XP_009690807.1	hypothetical protein	63
XP_009689321.1	conserved hypothetical protein	63
XP_009689504.1	hypothetical protein	62
XP_009691667.1	hypothetical protein	62
XP_009692733.1	uncharacterized protein	62
XP_009689501.1	conserved hypothetical protein	61
XP_009692730.1	uncharacterized protein	61
XP_009692744.1	uncharacterized protein	59
XP_009690105.1	uncharacterized protein	59
XP_009690969.1	conserved hypothetical protein	57
XP_009692646.1	Conserved hypothetical protein	56
XP_009691922.1	conserved hypothetical protein	56
XP_009689382.1	uncharacterized protein	55
XP_009691106.1	conserved hypothetical protein	52
XP_009692731.1	conserved hypothetical protein	48
XP_009690973.1	conserved hypothetical protein	47
XP_009689457.1	conserved hypothetical protein	46
XP_009689735.1	conserved hypothetical protein	44
XP_009690106.1	hypothetical protein	44
XP_009690635.1	conserved hypothetical protein	43
XP_009689373.1	uncharacterized protein	43
XP_009692796.1	uncharacterized protein	42
XP_009691212.1	conserved hypothetical protein	42
XP_009692822.1	cell division control protein	42
XP_009692622.1	uncharacterized protein	40
XP_009691770.1	conserved hypothetical protein	40
XP_009692529.1	conserved hypothetical protein	39
XP_009691663.1	conserved hypothetical protein	39
XP_009691885.1	uncharacterized protein	39
XP_009689606.1	ubiquitination-mediated degradation component	38
XP_009691032.1	conserved hypothetical protein	35
XP_009691014.1	uncharacterized protein	34
XP_009691940.1	uncharacterized protein	34
XP_009688926.1	fructose-bisphosphate aldolase 2	34
XP_009691769.1	uncharacterized protein	33

XP_009690008.1	conserved hypothetical protein	32
XP_009689956.1	ATP-dependent RNA helicase	31
XP_009691762.1	conserved hypothetical protein	31
XP_009690700.1	ABC transporter	31
XP_009690337.1	glutaminyl-tRNA synthetase	30
XP_009691047.1	conserved hypothetical protein	29
XP_009691277.1	uncharacterized protein	29
XP_009689955.1	conserved hypothetical protein	28
XP_009691016.1	uncharacterized protein	28
XP_009690964.1	conserved hypothetical protein	28
XP_009688836.1	uncharacterized protein	28
XP_009689410.1	conserved hypothetical protein	28
XP_009691866.1	conserved hypothetical protein	27
XP_009690113.1	uncharacterized protein	27
XP_009692741.1	DEAD-box family helicase	27
XP_009692204.1	DNA-directed RNA polymerase III largest subunit RPC1	27
XP_009692735.1	hypothetical protein	27
XP_009689584.1	uncharacterized protein	26
XP_009691242.1	conserved hypothetical protein	26
XP_009692726.1	uncharacterized protein	26
XP_009691802.1	uncharacterized protein	26
XP_009692823.1	conserved hypothetical protein	26
XP_009692043.1	predicted protein	25
XP_009689764.1	conserved hypothetical protein	25
XP_009692649.1	conserved hypothetical protein	25
XP_009690114.1	uncharacterized protein	25
XP_009692698.1	serine/threonine kinase	24
XP_009691937.1	uncharacterized protein	24
XP_009690690.1	transducin-like G-protein beta	24
XP_009689563.1	uncharacterized protein	24
XP_009690898.1	conserved hypothetical protein	24
XP_009691257.1	mRNA transport factor	24
XP_009690689.1	cleavage stimulation factor subunit 1	23
XP_009688835.1	conserved hypothetical protein	23
XP_009690391.1	conserved hypothetical protein	23
XP_009689565.1	chromatin assembly factor subunit	23
XP_009688851.1	cysteine desulfurase	23
XP_009690808.1	conserved hypothetical protein	23
XP_009690494.1	DNA topoisomerase 2	23

XP_009689262.1	uncharacterized protein	23
XP_009690034.1	uncharacterized protein	23
XP_009691031.1	ABC transporter	23
XP_009692769.1	uncharacterized protein	23
XP_009690766.1	uncharacterized protein	22
XP_009690995.1	uncharacterized protein	22
XP_009692743.1	conserved hypothetical protein	22
XP_009691052.1	conserved hypothetical protein	22
XP_009690971.1	conserved hypothetical protein	22
XP_009691840.1	hexose transporter	22
XP_009692826.1	uncharacterized protein	22
XP_009692358.1	uncharacterized protein	22
XP_009689202.1	uncharacterized protein	21
XP_009689133.1	predicted protein	21
XP_009691771.1	uncharacterized protein	21
XP_009692675.1	uncharacterized protein	21
XP_009691050.1	uncharacterized protein	21
XP_009690867.1	uncharacterized protein	21
XP_009690623.1	conserved hypothetical protein	21
XP_009691051.1	uncharacterized protein	21
XP_009689415.1	uncharacterized protein	20
XP_009690025.1	conserved hypothetical protein	20
XP_009690107.1	hypothetical protein	20
XP_009690855.1	conserved hypothetical protein	20
XP_009689244.1	uncharacterized protein	20
XP_009692674.1	uncharacterized protein	20
XP_009690023.1	ABC transporter	20
XP_009691936.1	hypothetical protein	20
XP_009692028.1	asparagine-rich protein	20
XP_009691422.1	conserved hypothetical protein	19
XP_009690697.1	1-hydroxy-2-methyl-2-(e)-butenyl 4-diphosphate synthase	19
XP_009691649.1	uncharacterized protein	19
XP_009691392.1	uncharacterized protein	19
XP_009691054.1	conserved hypothetical protein	18
XP_009691693.1	uncharacterized protein	18
XP_009691597.1	conserved hypothetical protein	18
XP_009690981.1	conserved hypothetical protein	18
XP_009691697.1	ATP-dependent RNA helicase	18
XP_009688917.1	uncharacterized protein	18

XP_009690066.1	uncharacterized protein	18
XP_009692787.1	uncharacterized protein	18
XP_009692276.1	uncharacterized protein	18
XP_009688887.1	uncharacterized protein	18
XP_009689355.1	uncharacterized protein	18
XP_009691442.1	uncharacterized protein	18
XP_009691821.1	conserved hypothetical protein	18
XP_009689585.1	uncharacterized protein	18
XP_009690929.1	conserved hypothetical protein	18
XP_009690947.1	conserved hypothetical protein	18
XP_009689297.1	conserved hypothetical protein	18
XP_009689462.1	flavin-containing amine oxidase	17
XP_009690285.1	conserved hypothetical protein	17
XP_009689785.1	uncharacterized protein	17
XP_009692746.1	uncharacterized protein	17
XP_009689492.1	coatamer subunit beta	17
XP_009689277.1	predicted protein	17
XP_009690863.1	pentatricopeptide repeat containing protein	17
XP_009689318.1	silent information regulator protein Sir2	17
XP_009690831.1	conserved hypothetical protein	16
XP_009689957.1	transcription or splicing factor-like protein	16
XP_009688837.1	conserved hypothetical protein	16
XP_009692732.1	hypothetical protein	16
XP_009691258.1	conserved hypothetical protein	16
XP_009691521.1	conserved hypothetical protein	16
XP_009690884.1	uncharacterized protein	16
XP_009690965.1	conserved hypothetical protein	16
XP_009691485.1	conserved hypothetical protein	16
XP_009691868.1	hypothetical protein	16
XP_009691048.1	conserved hypothetical protein	16
XP_009691316.1	molecular chaperone DnaJ	15
XP_009690341.1	uncharacterized protein	15
XP_009690534.1	conserved hypothetical protein	15
XP_009692645.1	conserved hypothetical protein	15
XP_009692324.1	uncharacterized protein	15
XP_009692263.1	uncharacterized protein	15
XP_009691842.1	uncharacterized protein	15
XP_009691592.1	Prp8 protein	15
XP_009690656.1	conserved hypothetical protein	15

XP_009691764.1	uncharacterized protein	15
XP_009692489.1	exoribonuclease	15
XP_009691914.1	conserved hypothetical protein	14
XP_009692479.1	conserved hypothetical protein	14
XP_009692725.1	conserved hypothetical protein	14
XP_009692139.1	conserved hypothetical protein	14
XP_009689506.1	50S ribosomal protein L17	14
XP_009691246.1	conserved hypothetical protein	14
XP_009689278.1	conserved hypothetical protein	14
XP_009691520.1	uncharacterized protein	14
XP_009691907.1	uncharacterized protein	14
XP_009692195.1	conserved hypothetical protein	14
XP_009692810.1	uncharacterized protein	14
XP_009691746.1	conserved hypothetical protein	14
XP_009689258.1	conserved hypothetical protein	14
XP_009692290.1	uncharacterized protein	14
XP_009691917.1	conserved hypothetical protein	14
XP_009692112.1	uncharacterized protein	14
XP_009691844.1	predicted protein	14
XP_009692137.1	conserved hypothetical protein	14
XP_009691055.1	uncharacterized protein	14
XP_009691878.1	conserved hypothetical protein	14
XP_009689774.1	DNA-directed RNA polymerase precursor	14
XP_009689105.1	60S acidic ribosomal protein P0	13
XP_009690869.1	uncharacterized protein	13
XP_009689361.1	uncharacterized protein	13
XP_009689443.1	uncharacterized protein	13
XP_009691867.1	hypothetical protein	13
XP_009690953.1	guanylyl cyclase	13
XP_009690990.1	uncharacterized protein	13
XP_009690787.1	hypothetical protein	13
XP_009692547.1	uncharacterized protein	13
XP_009689047.1	conserved hypothetical protein	13
XP_009690571.1	NADH-cytochrome b5 reductase	13
XP_009689145.1	conserved hypothetical protein	13
XP_009690610.1	conserved hypothetical protein	13
XP_009691708.1	conserved hypothetical protein	13
XP_009689607.1	DNA-directed RNA polymerase	13
XP_009692382.1	uncharacterized protein	13

XP_009692656.1	conserved hypothetical protein	13
XP_009692191.1	DEAD-box family helicase	13
XP_009691026.1	conserved hypothetical protein	13
XP_009688963.1	conserved hypothetical protein	12
XP_009691942.1	uncharacterized protein	12
XP_009692521.1	conserved hypothetical protein	12
XP_009689507.1	predicted protein	12
XP_009691818.1	uncharacterized protein	12
XP_009689495.1	conserved hypothetical protein	12
XP_009689328.1	conserved hypothetical protein	12
XP_009691645.1	uncharacterized protein	12
XP_009691587.1	conserved hypothetical protein	12
XP_009692491.1	condensin subunit	12
XP_009688838.1	uncharacterized protein	12
XP_009692653.1	Ran binding protein 1	12
XP_009689856.1	conserved hypothetical protein	12
XP_009692475.1	conserved hypothetical protein	12
XP_009690032.1	uncharacterized protein	12
XP_009691795.1	uncharacterized protein	12
XP_009691588.1	uncharacterized protein	12
XP_009689294.1	conserved hypothetical protein	12
XP_009691676.1	splicing factor subunit	12
XP_009690828.1	uncharacterized protein	12
XP_009692258.1	conserved hypothetical protein	11
XP_009692678.1	conserved hypothetical protein	11
XP_009691056.1	uncharacterized protein	11
XP_009690625.1	conserved hypothetical protein	11
XP_009689549.1	heat-shock protein	11
XP_009690299.1	RNA helicase	11
XP_009689312.1	conserved hypothetical protein	11
XP_009691849.1	conserved hypothetical protein	11
XP_009690297.1	conserved hypothetical protein	11
XP_009691888.1	conserved hypothetical protein	11
XP_009692454.1	conserved hypothetical protein	11
XP_009691665.1	hypothetical protein	11
XP_009690464.1	predicted protein	11
XP_009691785.1	hypothetical protein	11
XP_009691803.1	uncharacterized protein	11
XP_009689270.1	uncharacterized protein	11

XP_009691203.1	hypothetical protein	11
XP_009690934.1	conserved hypothetical protein	11
XP_009691053.1	uncharacterized protein	11
XP_009691683.1	uncharacterized protein	11
XP_009690693.1	serine/threonine protein kinase	11
XP_009690902.1	uncharacterized protein	11
XP_009689493.1	hypothetical protein	11
XP_009689076.1	uncharacterized protein	11
XP_009690650.1	thrombospondin%2C type I repeat containing protein	11
XP_009692790.1	conserved hypothetical protein	11
XP_009692035.1	DNA polymerase epsilon catalytic subunit	10
XP_009689952.1	uncharacterized protein	10
XP_009689381.1	uncharacterized protein	10
XP_009691852.1	conserved hypothetical protein	10
XP_009689393.1	DEAD-box helicase	10
XP_009691151.1	conserved hypothetical protein	10
XP_009689709.1	uncharacterized protein	10
XP_009689526.1	elongation factor 1-alpha	10
XP_009691916.1	uncharacterized protein	10
XP_009691857.1	carbamoyl phosphate synthase II	10
XP_009690890.1	conserved hypothetical protein	10
XP_009691908.1	uncharacterized protein	10
XP_009690972.1	conserved hypothetical protein	10
XP_009692017.1	conserved hypothetical protein	10
XP_009691159.1	U5 small nuclear ribonucleoprotein-specific helicase	10
XP_009690860.1	eukaryotic peptide chain release factor	10
XP_009691243.1	DEAD/DEAH-like helicase	10
XP_009689324.1	long-chain-fatty-acid--CoA ligase 5	10
XP_009689940.1	uncharacterized protein	10
XP_009691232.1	conserved hypothetical protein	10
XP_009691074.1	conserved hypothetical protein	10
XP_009692750.1	uncharacterized protein	10
XP_009689537.1	conserved hypothetical protein	10
XP_009691679.1	conserved hypothetical protein	10
XP_009692345.1	uncharacterized protein	10
XP_009692722.1	uncharacterized protein	10
XP_009690538.1	chromatin-binding protein	10
XP_009692483.1	Ran-binding protein	10
XP_009689480.1	uncharacterized protein	10

XP_009692589.1	uncharacterized protein	10
XP_009690026.1	hypothetical protein	10
XP_009691692.1	DNA-directed RNA polymerase D subunit	10
XP_009691441.1	homeodomain-like containing protein	10
XP_009690957.1	predicted protein	10
XP_009690477.1	conserved hypothetical protein	9
XP_009689922.1	DEAD-box family helicase	9
XP_009691122.1	uncharacterized protein	9
XP_009689370.1	conserved hypothetical protein	9
XP_009692761.1	conserved hypothetical protein	9
XP_009690355.1	uncharacterized protein	9
XP_009689066.1	ABC transporter	9
XP_009692523.1	conserved hypothetical protein	9
XP_009691393.1	hypothetical protein	9
XP_009691698.1	translation initiation factor	9
XP_009691743.1	conserved hypothetical protein	9
XP_009690128.1	replication licensing factor	9
XP_009692092.1	conserved hypothetical protein	9
XP_009690551.1	uncharacterized protein	9
XP_009689483.1	DEAD-box family helicase	9
XP_009691839.1	conserved hypothetical protein	9
XP_009690450.1	conserved hypothetical protein	9
XP_009689305.1	uncharacterized protein	9
XP_009690011.1	uncharacterized protein	9
XP_009690352.1	conserved hypothetical protein	9
XP_009691254.1	regulator of nonsense transcripts-related protein	9
XP_009689465.1	cation ATPase	9
XP_009689505.1	cytochrome c oxidase subunit 2	9
XP_009690245.1	uncharacterized protein	9
XP_009689970.1	uncharacterized protein	9
XP_009690090.1	conserved hypothetical protein	9
XP_009689750.1	threonyl-tRNA synthetase	9
XP_009689445.1	conserved hypothetical protein	9
XP_009689730.1	conserved hypothetical protein	9
XP_009691367.1	chromosome condensation protein	9
XP_009690811.1	conserved hypothetical protein	9
XP_009690938.1	uncharacterized protein	9
XP_009689829.1	conserved hypothetical protein	9
XP_009692164.1	uncharacterized protein	9

XP_009691819.1	conserved hypothetical protein	8
XP_009690936.1	conserved hypothetical protein	8
XP_009689251.1	conserved hypothetical protein	8
XP_009691171.1	uncharacterized protein	8
XP_009692501.1	uncharacterized protein	8
XP_009691823.1	conserved hypothetical protein	8
XP_009689201.1	HEAT repeat containing protein	8
XP_009691621.1	conserved hypothetical protein	8
XP_009690036.1	conserved hypothetical protein	8
XP_009692597.1	conserved hypothetical protein	8
XP_009692306.1	importin-alpha	8
XP_009691773.1	conserved hypothetical protein	8
XP_009689061.1	uncharacterized protein	8
XP_009689190.1	aminopeptidase N	8
XP_009690797.1	uncharacterized protein	8
XP_009689752.1	uncharacterized protein	8
XP_009690195.1	uncharacterized protein	8
XP_009690439.1	uncharacterized protein	8
XP_009690548.1	conserved hypothetical protein	8
XP_009690104.1	transcription factor	8
XP_009691101.1	uncharacterized protein	8
XP_009690701.1	uncharacterized protein	8
XP_009690443.1	uncharacterized protein	8
XP_009691038.1	hypothetical protein	8
XP_009692108.1	eukaryotic translation initiation factor 3 subunit 8	8
XP_009690866.1	conserved hypothetical protein	8
XP_009690366.1	conserved hypothetical protein	8
XP_009692639.1	uncharacterized protein	8
XP_009690028.1	hypothetical protein	8
XP_009691792.1	uncharacterized protein	8
XP_009690336.1	conserved hypothetical protein	8
XP_009691526.1	conserved hypothetical protein	8
XP_009691395.1	nucleolar protein	8
XP_009691410.1	P-type ATPase 2	8
XP_009691385.1	conserved hypothetical protein	8
XP_009692497.1	uncharacterized protein	8
XP_009691397.1	uncharacterized protein	8
XP_009690531.1	uncharacterized protein	8
XP_009690127.1	conserved hypothetical protein	8

XP_009691087.1	integral membrane protein	8
XP_009692277.1	hypothetical protein	8
XP_009688906.1	uncharacterized protein	8
XP_009692121.1	sodium transporter	8
XP_009691572.1	uncharacterized protein	8
XP_009690051.1	conserved hypothetical protein	8
XP_009690380.1	uncharacterized protein	8
XP_009691848.1	conserved hypothetical protein	8
XP_009689660.1	conserved hypothetical protein	8
XP_009692734.1	hypothetical protein	8
XP_009691411.1	DEAD-box family RNA helicase	8
XP_009689256.1	uncharacterized protein	8
XP_009691854.1	hypothetical protein	8
XP_009691213.1	40S ribosomal protein S3a	8
XP_009692116.1	vacuolar H+ATPase subunit	7
XP_009692321.1	conserved hypothetical protein	7
XP_009689765.1	7%2C8 dihydro-8-oxoguanine DNA glycosylase	7
XP_009688937.1	uncharacterized protein	7
XP_009690539.1	uncharacterized protein	7
XP_009689280.1	conserved hypothetical protein	7
XP_009689939.1	uncharacterized protein	7
XP_009692487.1	tyrosyl-tRNA synthetase	7
XP_009691670.1	conserved hypothetical protein	7
XP_009692074.1	conserved hypothetical protein	7
XP_009691571.1	conserved hypothetical protein	7
XP_009692785.1	hypothetical protein	7
XP_009690083.1	1-deoxy-D-xylulose 5-phosphate reducto-isomerase	7
XP_009689746.1	conserved hypothetical protein	7
XP_009690108.1	predicted protein	7
XP_009691089.1	conserved hypothetical protein	7
XP_009692457.1	uncharacterized protein	7
XP_009691174.1	uncharacterized protein	7
XP_009689388.1	conserved hypothetical protein	7
XP_009692736.1	tubulin subunit alpha	7
XP_009691881.1	conserved hypothetical protein	7
XP_009691564.1	conserved hypothetical protein	7
XP_009689210.1	conserved hypothetical protein	7
XP_009689837.1	patatin-family phospholipase	7
XP_009691202.1	conserved hypothetical protein	7

XP_009691691.1	phospholipase	7
XP_009690974.1	uncharacterized protein	7
XP_009692476.1	conserved hypothetical protein	7
XP_009691509.1	conserved hypothetical protein	7
XP_009690049.1	conserved hypothetical protein	7
XP_009691334.1	uncharacterized protein	7
XP_009691569.1	uncharacterized protein	7
XP_009691983.1	likely GTP/GDP exchange factor for ARF	7
XP_009690101.1	uncharacterized protein	7
XP_009689799.1	alanyl-tRNA synthetase	7
XP_009689338.1	conserved hypothetical protein	7
XP_009689540.1	uncharacterized protein	7
XP_009692459.1	MAC/perforin	7
XP_009689095.1	importin beta	7
XP_009692148.1	conserved hypothetical protein	7
XP_009692516.1	transcription modulator	7
XP_009689548.1	histone acetyltransferase	7
XP_009691248.1	conserved hypothetical protein	7
XP_009691015.1	conserved hypothetical protein	7
XP_009690454.1	conserved hypothetical protein	7
XP_009690544.1	uncharacterized protein	7
XP_009689475.1	uncharacterized protein	7
XP_009688840.1	ABC transporter	7
XP_009691880.1	conserved hypothetical protein	7
XP_009689159.1	uncharacterized protein	7
XP_009691604.1	hypothetical protein	7
XP_009690740.1	ubiquitin-activating enzyme E1	7
XP_009689822.1	uncharacterized protein	6
XP_009692686.1	conserved hypothetical protein	6
XP_009689658.1	uncharacterized protein	6
XP_009691341.1	uncharacterized protein	6
XP_009689046.1	E3 ubiquitin-protein ligase HUWE1	6
XP_009691359.1	conserved hypothetical protein	6
XP_009690238.1	conserved hypothetical protein	6
XP_009689131.1	conserved hypothetical protein	6
XP_009690926.1	conserved hypothetical protein	6
XP_009692768.1	uncharacterized protein	6
XP_009692153.1	Pbj2	6
XP_009691637.1	conserved hypothetical protein	6

XP_009690563.1	5'-3' exonuclease	6
XP_009689646.1	conserved hypothetical protein	6
XP_009692477.1	predicted protein	6
XP_009690198.1	conserved hypothetical protein	6
XP_009691864.1	uncharacterized protein	6
XP_009691593.1	conserved hypothetical protein	6
XP_009692553.1	uncharacterized protein	6
XP_009689449.1	conserved hypothetical protein	6
XP_009691057.1	conserved hypothetical protein	6
XP_009691776.1	conserved hypothetical protein	6
XP_009688978.1	membrance occupation and recognition nexus protein 1	6
XP_009690360.1	uncharacterized protein	6
XP_009692107.1	valyl-tRNA synthetase	6
XP_009692705.1	structure-specific recognition protein 1	6
XP_009690086.1	homeodomain-like containing protein	6
XP_009692710.1	conserved hypothetical protein	6
XP_009692052.1	uncharacterized protein	6
XP_009691423.1	uncharacterized protein	6
XP_009690213.1	1-phosphatidylinositol-4-phosphate 5-kinase	6
XP_009691824.1	uncharacterized protein	6
XP_009689073.1	peptidyl-prolyl cis-trans isomerase	6
XP_009689219.1	conserved hypothetical protein	6
XP_009692146.1	uncharacterized protein	6
XP_009689496.1	ribosomal protein L15	6
XP_009688869.1	hexokinase 1	6
XP_009688985.1	conserved hypothetical protein	6
XP_009689089.1	conserved hypothetical protein	6
XP_009692259.1	uncharacterized protein	6
XP_009692467.1	uncharacterized protein	6
XP_009692158.1	uncharacterized protein	6
XP_009692621.1	gamma adaptin	6
XP_009689616.1	phosphatidylinositol 4-kinase	6
XP_009688892.1	protein phosphatase 2c	6
XP_009689588.1	conserved hypothetical protein	6
XP_009690292.1	conserved hypothetical protein	6
XP_009690975.1	FKBP-type peptidyl-prolyl cis-trans isomerase	6
XP_009691298.1	ATP synthase subunit alpha	6
XP_009691863.1	conserved hypothetical protein	6
XP_009692351.1	conserved hypothetical protein	6

XP_009692129.1	tRNA-splicing endonuclease	6
XP_009692485.1	uncharacterized protein	6
XP_009689352.1	uncharacterized protein	6
XP_009691820.1	conserved hypothetical protein	6
XP_009689491.1	conserved hypothetical protein	6
XP_009690680.1	adenosylhomocysteinase	6
XP_009690132.1	uncharacterized protein	6
XP_009689895.1	conserved hypothetical protein	6
XP_009692753.1	conserved hypothetical protein	6
XP_009689843.1	conserved hypothetical protein	6
XP_009690993.1	hypothetical protein	6
XP_009691648.1	uncharacterized protein	6
XP_009690568.1	isoleucyl-tRNA synthetase	6
XP_009690702.1	uncharacterized protein	6
XP_009692554.1	uncharacterized protein	6
XP_009691930.1	conserved hypothetical protein	6
XP_009690699.1	uncharacterized protein	6
XP_009692435.1	DNA-binding chaperone	6
XP_009689390.1	uncharacterized protein	6
XP_009689079.1	conserved hypothetical protein	6
XP_009689299.1	uncharacterized protein	5
XP_009692618.1	uncharacterized protein	5
XP_009691514.1	ion-translocating ATPase	5
XP_009688971.1	mitochondrial processing peptidase	5
XP_009692014.1	uncharacterized protein	5
XP_009690527.1	uncharacterized protein	5
XP_009690885.1	uncharacterized protein	5
XP_009689033.1	uncharacterized protein	5
XP_009691946.1	uncharacterized protein	5
XP_009690185.1	uncharacterized protein	5
XP_009691754.1	hypothetical protein	5
XP_009692480.1	microfibrillar-associated protein	5
XP_009690564.1	uncharacterized protein	5
XP_009691574.1	chromosome segregation protein	5
XP_009692550.1	uncharacterized protein	5
XP_009690744.1	kinesin-like protein	5
XP_009691421.1	uncharacterized protein	5
XP_009689453.1	conserved hypothetical protein	5
XP_009689227.1	HEAT repeat containing protein	5

XP_009689510.1	protein kinase	5
XP_009689835.1	porin	5
XP_009691718.1	conserved hypothetical protein	5
XP_009691215.1	elongation factor tu	5
XP_009691977.1	glutamate dehydrogenase	5
XP_009692046.1	pentatricopeptide repeat containing protein	5
XP_009690727.1	uncharacterized protein	5
XP_009690997.1	uncharacterized protein	5
XP_009692755.1	Requim%2C req/dpf2	5
XP_009690753.1	uncharacterized protein	5
XP_009690976.1	conserved hypothetical protein	5
XP_009689067.1	uncharacterized protein	5
XP_009690887.1	uncharacterized protein	5
XP_009690836.1	conserved hypothetical protein	5
XP_009689524.1	conserved hypothetical protein	5
XP_009689434.1	uncharacterized protein	5
XP_009690056.1	uncharacterized protein	5
XP_009690331.1	helicase	5
XP_009690284.1	uncharacterized protein	5
XP_009691605.1	cysteine protease precursor TacP	5
XP_009690077.1	uncharacterized protein	5
XP_009690641.1	conserved hypothetical protein	5
XP_009690377.1	uncharacterized protein	5
XP_009689470.1	conserved hypothetical protein	5
XP_009690657.1	uncharacterized protein	5
XP_009692712.1	uncharacterized protein	5
XP_009689369.1	uncharacterized protein	5
XP_009692778.1	superoxide dismutase	5
XP_009691810.1	conserved hypothetical protein	5
XP_009691876.1	uncharacterized protein	5
XP_009689821.1	uncharacterized protein	5
XP_009691045.1	hypothetical protein	5
XP_009689265.1	conserved hypothetical protein	5
XP_009689098.1	structural maintenance of chromosome protein%2C type 1	5
XP_009690126.1	ubiquitin carboxyl-terminal hydrolase	5
XP_009691550.1	major facilitator superfamily MFS-1 protein	5
XP_009690729.1	protein transport protein	5
XP_009691973.1	uncharacterized protein	5
XP_009692315.1	uncharacterized protein	5

XP_009692500.1	replication factor C subunit	5
XP_009691805.1	4-methyl-5(b-hydroxyethyl)-thiazol monophosphate biosynthesis enzyme	5
XP_009690191.1	protein kinase	5
XP_009692727.1	uncharacterized protein	5
XP_009690622.1	conserved hypothetical protein	5
XP_009691808.1	uncharacterized protein	5
XP_009692772.1	chaperonin HSP60	5
XP_009688879.1	conserved hypothetical protein	5
XP_009691263.1	conserved hypothetical protein	5
XP_009692406.1	conserved hypothetical protein	5
XP_009689320.1	SprA protein	5
XP_009691640.1	uncharacterized protein	5
XP_009692431.1	chromosome segregation protein	5
XP_009689184.1	uncharacterized protein	5
XP_009689452.1	ABC transporter	5
XP_009691612.1	conserved hypothetical protein	5
XP_009689531.1	uncharacterized protein	5
XP_009689242.1	histone acetyltransferase gcn5-related	5
XP_009690642.1	uncharacterized protein	5
XP_009689755.1	uncharacterized protein	5
XP_009689301.1	ABC transporter	5
XP_009692749.1	conserved hypothetical protein	5
XP_009691875.1	U1 snRNP protein	5
XP_009690459.1	ATP-dependent RNA helicase	5
XP_009692742.1	uncharacterized protein	5
XP_009691286.1	conserved hypothetical protein	5
XP_009689090.1	translation elongation factor G	5
XP_009690231.1	conserved hypothetical protein	5
XP_009691981.1	uncharacterized protein	5
XP_009691616.1	uncharacterized protein	5
XP_009688938.1	conserved hypothetical protein	5
XP_009691836.1	uncharacterized protein	5
XP_009692347.1	metallopeptidase	5
XP_009691784.1	conserved hypothetical protein	5
XP_009689329.1	conserved hypothetical protein	5
XP_009690883.1	uncharacterized protein	5
XP_009690980.1	mono-oxygenase	5
XP_009691075.1	uncharacterized protein	5
XP_009689542.1	conserved hypothetical protein	5

XP_009690722.1	uncharacterized protein	5
XP_009692482.1	uncharacterized protein	5
XP_009692079.1	uncharacterized protein	5
XP_009691992.1	conserved hypothetical protein	4
XP_009690914.1	conserved hypothetical protein	4
XP_009689456.1	conserved hypothetical protein	4
XP_009689804.1	uncharacterized protein	4
XP_009690217.1	transcription initiation factor TFIIb	4
XP_009691196.1	RabGDI protein	4
XP_009689112.1	serine/threonine protein kinase	4
XP_009692517.1	conserved hypothetical protein	4
XP_009690843.1	26S proteasome non-ATPase regulatory subunit 12	4
XP_009692426.1	conserved hypothetical protein	4
XP_009689947.1	uncharacterized protein	4
XP_009690294.1	hypothetical protein	4
XP_009690602.1	60S ribosomal protein L4/L1	4
XP_009690616.1	casein kinase II subunit beta	4
XP_009691673.1	uncharacterized protein	4
XP_009689316.1	elongation factor 2	4
XP_009690300.1	uncharacterized protein	4
XP_009691722.1	uncharacterized protein	4
XP_009691157.1	uncharacterized protein	4
XP_009691293.1	uncharacterized protein	4
XP_009691453.1	conserved hypothetical protein	4
XP_009689072.1	mRNA capping enzyme subunit	4
XP_009689554.1	conserved hypothetical protein	4
XP_009690369.1	uncharacterized protein	4
XP_009690553.1	N2%2CN2-dimethylguanosine tRNA methyltransferase	4
XP_009689059.1	uncharacterized protein	4
XP_009692825.1	hypothetical protein	4
XP_009692262.1	conserved hypothetical protein	4
XP_009691210.1	conserved hypothetical protein	4
XP_009691312.1	SOH1-like protein	4
XP_009691860.1	glycogen synthase kinase	4
XP_009689208.1	uncharacterized protein	4
XP_009691779.1	conserved hypothetical protein	4
XP_009689474.1	26S proteasome regulatory subunit	4
XP_009691237.1	ubiquitin carboxyl-terminal hydrolase	4
XP_009692562.1	conserved hypothetical protein	4

XP_009689245.1	conserved hypothetical protein	4
XP_009689802.1	conserved hypothetical protein	4
XP_009690552.1	calcium-transporting ATPase	4
XP_009690436.1	uncharacterized protein	4
XP_009690613.1	conserved hypothetical protein	4
XP_009689509.1	conserved hypothetical protein	4
XP_009689115.1	conserved hypothetical protein	4
XP_009690647.1	uncharacterized protein	4
XP_009690624.1	conserved hypothetical protein	4
XP_009690949.1	serine/threonine protein phosphatase pp2a catalytic subunit	4
XP_009689174.1	conserved hypothetical protein	4
XP_009689864.1	queuine tRNA-ribosyltransferase	4
XP_009691856.1	uncharacterized protein	4
XP_009689106.1	uncharacterized protein	4
XP_009690716.1	uncharacterized protein	4
XP_009691431.1	uncharacterized protein	4
XP_009689122.1	conserved hypothetical protein	4
XP_009690629.1	conserved hypothetical protein	4
XP_009689715.1	uncharacterized protein	4
XP_009689518.1	conserved hypothetical protein	4
XP_009691512.1	conserved hypothetical protein	4
XP_009691716.1	uncharacterized protein	4
XP_009691877.1	vacuolar ATP synthase subunit H	4
XP_009692310.1	uncharacterized protein	4
XP_009690924.1	hypothetical protein	4
XP_009691744.1	alpha adaptin	4
XP_009690031.1	hypothetical protein	4
XP_009688951.1	protein disulfide isomerase precursor	4
XP_009689408.1	NEDD8-activating enzyme E1 catalytic subunit	4
XP_009692427.1	conserved hypothetical protein	4
XP_009688948.1	DNA-directed RNA polymerase subunit alpha	4
XP_009692609.1	conserved hypothetical protein	4
XP_009691689.1	DNA polymerase	4
XP_009692409.1	uncharacterized protein	4
XP_009692559.1	conserved hypothetical protein	4
XP_009691394.1	conserved hypothetical protein	4
XP_009690333.1	predicted protein	4
XP_009691972.1	uncharacterized protein	4
XP_009692403.1	uncharacterized protein	4

XP_009689663.1	uncharacterized protein	4
XP_009692717.1	uncharacterized protein	4
XP_009690211.1	DNA topoisomerase I	4
XP_009689342.1	uncharacterized protein	4
XP_009691939.1	hypothetical protein	4
XP_009690911.1	cathepsin-like cysteine protease	4
XP_009691786.1	2-oxoglutarate dehydrogenase E1 component	4
XP_009691681.1	uncharacterized protein	4
XP_009689077.1	glutathione synthetase	4
XP_009691451.1	conserved hypothetical protein	4
XP_009692502.1	glyceraldehyde-3-phosphate dehydrogenase	4
XP_009691747.1	uncharacterized protein	4
XP_009691855.1	60S ribosomal protein L24	4
XP_009690536.1	DNA helicase	4
XP_009690353.1	conserved hypothetical protein	4
XP_009689581.1	uncharacterized protein	4
XP_009691443.1	conserved hypothetical protein	4
XP_009691704.1	DNA-directed RNA polymerase	4
XP_009691653.1	RNA 3'-terminal phosphate cyclase	4
XP_009692183.1	conserved hypothetical protein	4
XP_009691209.1	uncharacterized protein	4
XP_009691013.1	molecular chaperone protein	4
XP_009691227.1	uncharacterized protein	4
XP_009690609.1	pyrophosphate--fructose 6-phosphate 1-phosphotransferase subunit alpha	4
XP_009689036.1	uncharacterized protein	4
XP_009689078.1	glutathione synthetase	4
XP_009691711.1	ankyrin repeat containing protein	4
XP_009689455.1	adaptor protein	4
XP_009690970.1	hypothetical protein	4
XP_009690827.1	conserved hypothetical protein	4
XP_009690177.1	coatomer complex subunit alpha	4
XP_009690791.1	uncharacterized protein	4
XP_009689466.1	conserved hypothetical protein	4
XP_009689116.1	3'-5' exoribonuclease	4
XP_009690946.1	conserved hypothetical protein	4
XP_009689950.1	uncharacterized protein	4
XP_009690620.1	conserved hypothetical protein	4
XP_009689590.1	conserved hypothetical protein	4
XP_009690881.1	uncharacterized protein	4

XP_009691869.1	conserved hypothetical protein	4
XP_009689523.1	uncharacterized protein	4
XP_009691383.1	conserved hypothetical protein	4
XP_009691380.1	DNA ligase 1 precursor	4
XP_009692642.1	uncharacterized protein	4
XP_009692545.1	uncharacterized protein	4
XP_009689004.1	uncharacterized protein	4
XP_009691409.1	clathrin heavy chain	4
XP_009690234.1	molecular chaperone DnaJ	4
XP_009690317.1	conserved hypothetical protein	4
XP_009691800.1	hypothetical protein	4
XP_009692261.1	leucyl-tRNA synthetase	4
XP_009688972.1	uncharacterized protein	4
XP_009692354.1	uncharacterized protein	4
XP_009689826.1	uncharacterized protein	4
XP_009690348.1	uncharacterized protein	4
XP_009691581.1	conserved hypothetical protein	4
XP_009689775.1	conserved hypothetical protein	4
XP_009692064.1	conserved hypothetical protein	4
XP_009691529.1	conserved hypothetical protein	4
XP_009690891.1	hypothetical protein	4
XP_009692094.1	nucleoside diphosphate hydrolase	4
XP_009691474.1	uncharacterized protein	4
XP_009689334.1	uncharacterized protein	4
XP_009690813.1	conserved hypothetical protein	4
XP_009691012.1	uncharacterized protein	4
XP_009689180.1	DNA polymerase I	4
XP_009691837.1	cysteinyl-tRNA synthetase	4
XP_009689350.1	conserved hypothetical protein	4
XP_009689322.1	uncharacterized protein	4
XP_009691752.1	methionyl-tRNA synthetase	4
XP_009691898.1	hypothetical protein	4
XP_009689726.1	molecular chaperone protein	4
XP_009691496.1	conserved hypothetical protein	4
XP_009692178.1	40S ribosomal protein S4	4
XP_009690977.1	actin 1	4
XP_009692494.1	uncharacterized protein	4
XP_009691259.1	conserved hypothetical protein	4
XP_009691794.1	uncharacterized protein	4

XP_009690927.1	hypothetical protein	4
XP_009692576.1	uncharacterized protein	4
XP_009689427.1	DEAD-box family helicase	4
XP_009689341.1	ATP-dependent helicase	4
XP_009690041.1	uncharacterized protein	4
XP_009692185.1	conserved hypothetical protein	4
XP_009691622.1	conserved hypothetical protein	4
XP_009690502.1	diacylglycerol kinase	4
XP_009690668.1	clathrin-coat assembly protein	4
XP_009691772.1	thioredoxin reductase	4
XP_009691656.1	dihydrolipoyl dehydrogenase	4
XP_009690212.1	uncharacterized protein	4
XP_009692387.1	uncharacterized protein	4
XP_009690345.1	Bromodomain-containing protein 1	3
XP_009690637.1	mitochondrial carrier protein	3
XP_009691883.1	conserved hypothetical protein	3
XP_009691328.1	conserved hypothetical protein	3
XP_009690372.1	uncharacterized protein	3
XP_009689818.1	conserved hypothetical protein	3
XP_009692188.1	conserved hypothetical protein	3
XP_009690047.1	ubiquitin-transferase	3
XP_009691757.1	uncharacterized protein	3
XP_009691602.1	cysteine protease precursor TacP	3
XP_009692813.1	phosphatidate cytidylyltransferase	3
XP_009691282.1	queuine tRNA-ribosyltransferase-like protein	3
XP_009690618.1	conserved hypothetical protein	3
XP_009691935.1	conserved hypothetical protein	3
XP_009690769.1	conserved hypothetical protein	3
XP_009691240.1	ferrodoxin reductase-like protein	3
XP_009691440.1	uncharacterized protein	3
XP_009691720.1	conserved hypothetical protein	3
XP_009692298.1	uncharacterized protein	3
XP_009690595.1	conserved hypothetical protein	3
XP_009690209.1	3-demethylubiquinone-9 3-methyltransferase-like protein	3
XP_009689031.1	autoaggregation-mediating protein	3
XP_009692624.1	uncharacterized protein	3
XP_009691482.1	uncharacterized protein	3
XP_009689288.1	DNA replication licensing factor Mcm2	3
XP_009688859.1	conserved hypothetical protein	3

XP_009691705.1	uncharacterized protein	3
XP_009690567.1	conserved hypothetical protein	3
XP_009689943.1	predicted protein	3
XP_009689056.1	conserved hypothetical protein	3
XP_009690357.1	uncharacterized protein	3
XP_009692439.1	conserved hypothetical protein	3
XP_009690632.1	conserved hypothetical protein	3
XP_009691954.1	uncharacterized protein	3
XP_009692070.1	protein kinase	3
XP_009691400.1	aspartyl-tRNA synthetase	3
XP_009691002.1	conserved hypothetical protein	3
XP_009689144.1	uncharacterized protein	3
XP_009690155.1	conserved hypothetical protein	3
XP_009692309.1	uncharacterized protein	3
XP_009689128.1	conserved hypothetical protein	3
XP_009690557.1	DNA replication licensing factor	3
XP_009691320.1	conserved hypothetical protein	3
XP_009692025.1	conserved hypothetical protein	3
XP_009691488.1	pentatricopeptide repeat containing protein	3
XP_009690886.1	conserved hypothetical protein	3
XP_009691641.1	conserved hypothetical protein	3
XP_009691146.1	zinc transport protein	3
XP_009689068.1	uncharacterized protein	3
XP_009689378.1	conserved hypothetical protein	3
XP_009690734.1	conserved hypothetical protein	3
XP_009689569.1	conserved hypothetical protein	3
XP_009691279.1	conserved hypothetical protein	3
XP_009690741.1	HEAT repeat containing protein	3
XP_009691672.1	uncharacterized protein	3
XP_009692453.1	uncharacterized protein	3
XP_009689603.1	recombinational repair protein	3
XP_009691582.1	uncharacterized protein	3
XP_009690720.1	Ngp	3
XP_009690120.1	conserved hypothetical protein	3
XP_009689904.1	predicted protein	3
XP_009689008.1	prohibitin	3
XP_009690199.1	conserved hypothetical protein	3
XP_009689942.1	uncharacterized protein	3
XP_009690381.1	uncharacterized protein	3

XP_009692316.1	RNA polymerase small subunit	3
XP_009690935.1	uncharacterized protein	3
XP_009690173.1	DNA repair protein Rad50	3
XP_009692779.1	tRNA modification GTPase	3
XP_009691446.1	proteasome subunit	3
XP_009692157.1	heat-shock protein HSP70	3
XP_009689530.1	uncharacterized protein	3
XP_009689263.1	uncharacterized protein	3
XP_009689152.1	uncharacterized protein	3
XP_009692586.1	conserved hypothetical protein	3
XP_009691630.1	uncharacterized protein	3
XP_009689806.1	uncharacterized protein	3
XP_009690532.1	uncharacterized protein	3
XP_009691315.1	conserved hypothetical protein	3
XP_009692180.1	conserved hypothetical protein	3
XP_009689276.1	myosin a	3
XP_009691685.1	chaperonin CPN60	3
XP_012965617.1	cytochrome c oxidase subunit 1	3
XP_009692682.1	hypothetical protein	3
XP_009692096.1	uncharacterized protein	3
XP_009692456.1	hypothetical protein	3
XP_009691373.1	major facilitator superfamily MFS-1 protein	3
XP_009692418.1	serine/threonine protein phosphatase	3
XP_009689096.1	uncharacterized protein	3
XP_009691538.1	conserved hypothetical protein	3
XP_009690100.1	uncharacterized protein	3
XP_009690265.1	uncharacterized protein	3
XP_009692176.1	protein kinase	3
XP_009688834.1	conserved hypothetical protein	3
XP_009690585.1	sulfate transporter	3
XP_009692584.1	conserved hypothetical protein	3
XP_009691372.1	ubiquitin carrier protein	3
XP_009692783.1	conserved hypothetical protein	3
XP_009689347.1	conserved hypothetical protein	3
XP_009689398.1	splicing factor	3
XP_009689478.1	uncharacterized protein	3
XP_009691635.1	Conserved hypothetical protein	3
XP_009689691.1	DNA polymerase delta catalytic subunit	3
XP_009689544.1	uncharacterized protein	3

XP_009691961.1	phosphoinositide binding protein	3
XP_009692507.1	uncharacterized protein	3
XP_009690434.1	conserved hypothetical protein	3
XP_009690438.1	uncharacterized protein	3
XP_009691696.1	conserved hypothetical protein	3
XP_009689974.1	conserved hypothetical protein	3
XP_009692357.1	conserved hypothetical protein	3
XP_009689224.1	predicted protein	3
XP_009691426.1	adenosine monophosphate deaminase 2 (isoform L) isoform 3	3
XP_009692331.1	uncharacterized protein	3
XP_009689780.1	conserved hypothetical protein	3
XP_009690141.1	eukaryotic translation initiation factor 4a	3
XP_009690471.1	uncharacterized protein	3
XP_009692428.1	conserved hypothetical protein	3
XP_009691647.1	DNA replication licensing factor	3
XP_009690714.1	uncharacterized protein	3
XP_009690181.1	uncharacterized protein	3
XP_009692065.1	Hbeta58/Vps26 protein	3
XP_009690302.1	T-complex protein 1 epsilon subunit	3
XP_009692637.1	conserved hypothetical protein	3
XP_009690506.1	conserved hypothetical protein	3
XP_009690524.1	conserved hypothetical protein	3
XP_009689543.1	conserved hypothetical protein	3
XP_009692464.1	conserved hypothetical protein	3
XP_009690923.1	conserved hypothetical protein	3
XP_009691201.1	protein farnesyltransferase subunit alpha	3
XP_009689949.1	conserved hypothetical protein	3
XP_009692193.1	conserved hypothetical protein	3
XP_009691425.1	conserved hypothetical protein	3
XP_009689052.1	eukaryotic translation initiation factor 3 subunit 10	3
XP_009692264.1	uncharacterized protein	3
XP_009690535.1	centromere/microtubule binding protein	3
XP_009691271.1	conserved hypothetical protein	3
XP_009692339.1	conserved hypothetical protein	3
XP_009691027.1	uncharacterized protein	3
XP_009692342.1	chromosome maintenance protein	3
XP_009691290.1	uncharacterized protein	3
XP_009690329.1	conserved hypothetical protein	3
XP_009689487.1	uncharacterized protein	3

XP_009688952.1	conserved hypothetical protein	3
XP_009692462.1	conserved hypothetical protein	3
XP_009692759.1	conserved hypothetical protein	3
XP_009691682.1	conserved hypothetical protein	3
XP_009691465.1	phosphatidylinositol-4-phosphate 5-kinase	3
XP_009691458.1	uncharacterized protein	3
XP_009688852.1	uncharacterized protein	3
XP_009692466.1	uncharacterized protein	3
XP_009688920.1	conserved hypothetical protein	3
XP_009689268.1	exosome complex exonuclease rrp44	3
XP_009689166.1	uncharacterized protein	3
XP_009690449.1	uncharacterized protein	3
XP_009690194.1	translation initiation factor IF-2	3
XP_009691780.1	conserved hypothetical protein	3
XP_009689915.1	DNA topoisomerase 2	3
XP_009691847.1	aspartyl protease precursor	3
XP_009691540.1	peptidyl-prolyl cis-trans isomerase	3
XP_009691205.1	uncharacterized protein	3
XP_009691525.1	2-C-methyl-D-erythritol 2%2C4-cyclodiphosphate synthase	3
XP_009691434.1	conserved hypothetical protein	3
XP_009690851.1	conserved hypothetical protein	3
XP_009691707.1	conserved hypothetical protein	3
XP_009692207.1	serine/threonine protein kinase	3
XP_009691093.1	DEAD-box RNA helicase	3
XP_009692292.1	uncharacterized protein	3
XP_009692640.1	nucleosome/chromatin assembly factor	3
XP_009691241.1	conserved hypothetical protein	3
XP_009690433.1	uncharacterized protein	3
XP_009691390.1	cleavage and polyadenylation specificty factor subunit	3
XP_009692367.1	uncharacterized protein	3
XP_009692760.1	uncharacterized protein	3
XP_009692509.1	conserved hypothetical protein	3
XP_009691816.1	uncharacterized protein	3
XP_009689711.1	P-type ATPase	3
XP_009691399.1	cell division cycle CDC48 homologue/transitional endoplasmic reticulum ATPase	3
XP_009692751.1	uncharacterized protein	3
XP_009690335.1	cyclin-dependent protein kinase PHO85 homologue	3
XP_009689442.1	uncharacterized protein	3
XP_009689100.1	conserved hypothetical protein	3

XP_009689555.1	conserved hypothetical protein	3
XP_009691601.1	cysteine proteinase precursor	3
XP_009691483.1	prohibitin	3
XP_009691545.1	glucose-6-phosphate isomerase	3
XP_009690793.1	farnesyl-protein transferase beta subunit	3
XP_009690604.1	conserved hypothetical protein	3
XP_009692349.1	elongation factor 1-gamma	3
XP_009690768.1	conserved hypothetical protein	3
XP_009688839.1	hypothetical protein	3
XP_009689497.1	Plasmodium falciparum CPW-WPC repeat containing protein	3
XP_009689971.1	ribonuclease P subunit	3
XP_009692275.1	uncharacterized protein	3
XP_009688992.1	uncharacterized protein	3
XP_009690069.1	uncharacterized protein	3
XP_009689511.1	succinyl-Coa ligase%2C subunit	3
XP_009689467.1	uncharacterized protein	3
XP_009691974.1	uncharacterized protein	3
XP_009691783.1	conserved hypothetical protein	3
XP_009690781.1	uncharacterized protein	3
XP_009692234.1	conserved hypothetical protein	3
XP_009691166.1	conserved hypothetical protein	3
XP_009689101.1	26S proteasome regulatory particle	3
XP_009692781.1	uncharacterized protein	3
XP_009690482.1	conserved hypothetical protein	3
XP_009691596.1	uncharacterized protein	3
XP_009689463.1	DNA replication licensing factor	3
XP_009690508.1	conserved hypothetical protein	3
XP_009692363.1	uncharacterized protein	3
XP_009689232.1	conserved hypothetical protein	3
XP_009689284.1	uncharacterized protein	3
XP_009689724.1	conserved hypothetical protein	3
XP_009691123.1	uncharacterized protein	3
XP_009690281.1	uncharacterized protein	3
XP_009691454.1	conserved hypothetical protein	3
XP_009692058.1	conserved hypothetical protein	3
XP_009689973.1	ethanolamine phosphotransferase	3
XP_009692557.1	conserved hypothetical protein	3
XP_009691893.1	conserved hypothetical protein	3
XP_009690809.1	uncharacterized protein	3

XP_009690665.1	GDP-mannose pyrophosphorylase	3
XP_009690703.1	CAMP-dependent protein kinase%2C beta-catalytic subunit	3
XP_009691034.1	conserved hypothetical protein	3
XP_009688850.1	conserved hypothetical protein	3
XP_009690686.1	choline/ethanolamine kinase	3
XP_009689439.1	DNA-directed RNA polymerase III	3
XP_009692243.1	ankyrin repeat containing protein	3
XP_009692136.1	conserved hypothetical protein	3
XP_009692392.1	conserved hypothetical protein	3
XP_009689866.1	hydrolase	3
XP_009689060.1	uncharacterized protein	3
XP_009692037.1	uncharacterized protein	3
XP_009689417.1	conserved hypothetical protein	3
XP_009691620.1	bifunctional nuclease	3
XP_009692365.1	dimethyladenosine transferase	3
XP_009691460.1	replication factor	3
XP_009690342.1	conserved hypothetical protein	3
XP_009692047.1	endopeptidase ATP-binding subunit	3
XP_009691662.1	uncharacterized protein	3
XP_009692031.1	conserved hypothetical protein	3
XP_009691817.1	uncharacterized protein	3
XP_009692414.1	uncharacterized protein	3
XP_009689639.1	conserved hypothetical protein	3
XP_009689293.1	conserved hypothetical protein	3
XP_009690775.1	uncharacterized protein	3
XP_009689710.1	uncharacterized protein	3
XP_009692105.1	conserved hypothetical protein	3
XP_009692688.1	uncharacterized protein	3
XP_009690365.1	uncharacterized protein	3
XP_009689064.1	conserved hypothetical protein	3
XP_009692287.1	cytosol aminopeptidase	3
XP_009689164.1	proliferation-associated protein 2g4	3
XP_009692569.1	uncharacterized protein	2
XP_009692269.1	conserved hypothetical protein	2
XP_009692072.1	conserved hypothetical protein	2
XP_009690085.1	uncharacterized protein	2
XP_009691524.1	uncharacterized protein	2
XP_009689850.1	conserved hypothetical protein	2
XP_009692583.1	uncharacterized protein	2

XP_009692458.1	DNA-directed RNA polymerase	2
XP_009690687.1	choline/ethanolamine kinase	2
XP_009690619.1	conserved hypothetical protein	2
XP_009692144.1	uncharacterized protein	2
XP_009692192.1	hypothetical protein	2
XP_009690576.1	nucleolar protein Nop5	2
XP_009691573.1	casein kinase	2
XP_009691234.1	conserved hypothetical protein	2
XP_009689772.1	conserved hypothetical protein	2
XP_009691182.1	conserved hypothetical protein	2
XP_009690237.1	conserved hypothetical protein	2
XP_009690505.1	conserved hypothetical protein	2
XP_009692252.1	conserved hypothetical protein	2
XP_009691361.1	conserved hypothetical protein	2
XP_009692739.1	conserved hypothetical protein	2
XP_009692651.1	splicing factor 3b subunit	2
XP_009692383.1	branched-chain alpha-keto acid dihydrolipoyl acyltransferase precursor	2
XP_009689168.1	conserved hypothetical protein	2
XP_009689675.1	conserved hypothetical protein	2
XP_009689446.1	hypothetical protein	2
XP_009689421.1	adenylate kinase	2
XP_009690932.1	conserved hypothetical protein	2
XP_009690837.1	conserved hypothetical protein	2
XP_009689148.1	uncharacterized protein	2
XP_009690546.1	uncharacterized protein	2
XP_009691247.1	uncharacterized protein	2
XP_009688949.1	ubiquitin-activating enzyme E1	2
XP_009691982.1	ubiquitin-like protease	2
XP_009691459.1	DEAD-box family RNA helicase	2
XP_009691349.1	zinc transport protein	2
XP_009690016.1	conserved hypothetical protein	2
XP_009692548.1	uncharacterized protein	2
XP_009689153.1	conserved hypothetical protein	2
XP_009692124.1	conserved hypothetical protein	2
XP_009691470.1	protein disulphide isomerase	2
XP_009689621.1	conserved hypothetical protein	2
XP_009691536.1	conserved hypothetical protein	2
XP_009689696.1	uncharacterized protein	2
XP_009691941.1	conserved hypothetical protein	2

XP_009692150.1	hypothetical protein	2
XP_009691976.1	uncharacterized protein	2
XP_009689440.1	hypothetical protein	2
XP_009689853.1	conserved hypothetical protein	2
XP_009689708.1	conserved hypothetical protein	2
XP_009689884.1	kinesin	2
XP_009691732.1	conserved hypothetical protein	2
XP_009691417.1	origin recognition complex protein 1	2
XP_009692463.1	DNA-directed RNA polymerase	2
XP_009690547.1	ATP-dependent RNA helicase	2
XP_009692225.1	uncharacterized protein	2
XP_009689814.1	conserved hypothetical protein	2
XP_009689220.1	conserved hypothetical protein	2
XP_009690839.1	conserved hypothetical protein	2
XP_009692291.1	conserved hypothetical protein	2
XP_009692109.1	uncharacterized protein	2
XP_009691631.1	uncharacterized protein	2
XP_009690200.1	uncharacterized protein	2
XP_009691017.1	aldo-keto reductase	2
XP_009690733.1	calcium-dependent protein kinase	2
XP_009690219.1	uncharacterized protein	2
XP_009692080.1	ubiquitin carboxyl-terminal hydrolase	2
XP_009691105.1	hypothetical protein	2
XP_009692160.1	uncharacterized protein	2
XP_009690516.1	uncharacterized protein	2
XP_009690667.1	uncharacterized protein	2
XP_009692239.1	SWI/SNF-related chromatin remodelling factor	2
XP_009689407.1	uncharacterized protein	2
XP_009689836.1	uncharacterized protein	2
XP_009691338.1	uncharacterized protein	2
XP_009689123.1	predicted protein	2
XP_009692067.1	conserved hypothetical protein	2
XP_009689771.1	conserved hypothetical protein	2
XP_009690803.1	conserved hypothetical protein	2
XP_009691125.1	eukaryotic translation initiation factor 3 subunit 7	2
XP_009688924.1	protein transport protein sec24-like	2
XP_009692218.1	conserved hypothetical protein	2
XP_009689433.1	uncharacterized protein	2
XP_009692578.1	conserved hypothetical protein	2

XP_009692059.1	conserved hypothetical protein	2
XP_009690596.1	uncharacterized protein	2
XP_009691715.1	conserved hypothetical protein	2
XP_009691562.1	conserved hypothetical protein	2
XP_009692540.1	conserved hypothetical protein	2
XP_009691567.1	uncharacterized protein	2
XP_009692103.1	conserved hypothetical protein	2
XP_009690343.1	conserved hypothetical protein	2
XP_009692700.1	Pumilio RNA-binding region repeat containing protein	2
XP_009691250.1	conserved hypothetical protein	2
XP_009689508.1	conserved hypothetical protein	2
XP_009691351.1	DAZ-associated protein 1	2
XP_009690184.1	phenylalanyl-tRNA synthetase	2
XP_009690346.1	uncharacterized protein	2
XP_009692203.1	conserved hypothetical protein	2
XP_009690076.1	hypothetical protein	2
XP_009689001.1	uncharacterized protein	2
XP_009690493.1	uncharacterized protein	2
XP_009689758.1	uncharacterized protein	2
XP_009689053.1	uncharacterized protein	2
XP_009692343.1	conserved hypothetical protein	2
XP_009691322.1	nicotinate-nucleotide adenylyltransferase-like protein	2
XP_009691750.1	protein kinase	2
XP_009689644.1	eukaryotic translation initiation factor 2 subunit alpha	2
XP_009691600.1	uncharacterized protein	2
XP_009691255.1	conserved hypothetical protein	2
XP_009689629.1	uncharacterized protein	2
XP_009692283.1	uncharacterized protein	2
XP_009691477.1	uncharacterized protein	2
XP_009691332.1	conserved hypothetical protein	2
XP_009690420.1	ABC transporter	2
XP_009690118.1	uncharacterized protein	2
XP_009692182.1	conserved hypothetical protein	2
XP_009689731.1	transitional endoplasmic reticulum ATPase	2
XP_009690630.1	proliferating cell nuclear antigen 2	2
XP_009689154.1	uncharacterized protein	2
XP_009689593.1	conserved hypothetical protein	2
XP_009689169.1	protein transport protein sec24-like	2
XP_009692368.1	RNA polymerases I and III subunit	2

XP_009692619.1	conserved hypothetical protein	2
XP_009692301.1	prohibitin-like protein	2
XP_009689182.1	ATP synthase subunit beta	2
XP_009692005.1	uncharacterized protein	2
XP_009689189.1	uncharacterized protein	2
XP_009691611.1	conserved hypothetical protein	2
XP_009690920.1	CTP synthase	2
XP_009691185.1	peptide chain release factor 2	2
XP_009689275.1	hypothetical protein	2
XP_009691343.1	tRNA nucleotidyltransferase	2
XP_009690123.1	long-chain acyl-CoA synthetase	2
XP_009689359.1	conserved hypothetical protein	2
XP_009689599.1	conserved hypothetical protein	2
XP_009689657.1	splicing factor	2
XP_009691594.1	conserved hypothetical protein	2
XP_009689213.1	serine-threonine protein kinase	2
XP_009692667.1	predicted protein	2
XP_009690959.1	uncharacterized protein	2
XP_009691490.1	RNA processing factor	2
XP_009692806.1	conserved hypothetical protein	2
XP_009691103.1	elongation factor g 1%2C mitochondrial	2
XP_009691806.1	4-methyl-5(b-hydroxyethyl)-thiazol monophosphate biosynthesis enzyme	2
XP_009692715.1	conserved hypothetical protein	2
XP_009689266.1	conserved hypothetical protein	2
XP_009690166.1	heat shock protein	2
XP_009692784.1	conserved hypothetical protein	2
XP_009690057.1	conserved hypothetical protein	2
XP_009689732.1	uncharacterized protein	2
XP_009689011.1	peptidyl-prolyl cis-trans isomerase	2
XP_009691253.1	hypothetical protein	2
XP_009691200.1	conserved hypothetical protein	2
XP_009691283.1	conserved hypothetical protein	2
XP_009690485.1	ABC transporter	2
XP_009692443.1	26S proteasome regulatory subunit	2
XP_009689149.1	conserved hypothetical protein	2
XP_009690631.1	hypothetical protein	2
XP_009692145.1	conserved hypothetical protein	2
XP_009689539.1	disrupter of silencing protein	2
XP_009690183.1	uncharacterized protein	2

XP_009689601.1	aspartyl protease	2
XP_009692029.1	DEAD-box family ATP-dependent helicase	2
XP_009691481.1	neutral sphingomyelinase	2
XP_009690410.1	U1/2 small nuclear ribonucleoprotein	2
XP_009689386.1	uncharacterized protein	2
XP_009689230.1	tRNA delta (2)-isopentenylpyrophosphate transferase	2
XP_009690520.1	uncharacterized protein	2
XP_009692512.1	uncharacterized protein	2
XP_009689978.1	uncharacterized protein	2
XP_009689450.1	ABC transporter	2
XP_009690206.1	erythrocyte membrane-associated malaria antigen-like	2
XP_009689954.1	major facilitator superfamily MFS-1 protein	2
XP_009691433.1	conserved hypothetical protein	2
XP_009692446.1	uncharacterized protein	2
XP_009689683.1	uncharacterized protein	2
XP_009690306.1	major facilitator superfamily MFS-1 protein	2
XP_009689515.1	uncharacterized protein	2
XP_009690709.1	conserved hypothetical protein	2
XP_009688984.1	uncharacterized protein	2
XP_009692777.1	uncharacterized protein	2
XP_009689712.1	DEAD-box family RNA helicase	2
XP_009690444.1	uncharacterized protein	2
XP_009692605.1	hypothetical protein	2
XP_009690904.1	conserved hypothetical protein	2
XP_009692450.1	transcription factor	2
XP_009692417.1	26S proteasome regulatory subunit	2
XP_009691155.1	conserved hypothetical protein	2
XP_009689951.1	conserved hypothetical protein	2
XP_009689729.1	SWI/SNF family transcriptional activator protein	2
XP_009690894.1	uncharacterized protein	2
XP_009692219.1	replication factor C	2
XP_009689330.1	tryptophanyl-tRNA synthetase	2
XP_009690236.1	transporter protein cg10	2
XP_009689142.1	Conserved hypothetical protein	2
XP_009689773.1	conserved hypothetical protein	2
XP_009690050.1	conserved hypothetical protein	2
XP_009691381.1	conserved hypothetical protein	2
XP_009689728.1	conserved hypothetical protein	2
XP_009691152.1	uncharacterized protein	2

XP_009689906.1	conserved hypothetical protein	2
XP_009688945.1	uncharacterized protein	2
XP_009690820.1	uncharacterized protein	2
XP_009688936.1	uncharacterized protein	2
XP_009691559.1	uncharacterized protein	2
XP_009691789.1	hypothetical protein	2
XP_009689700.1	uncharacterized protein	2
XP_009691736.1	conserved hypothetical protein	2
XP_009689727.1	uncharacterized protein	2
XP_009689357.1	ribosomal protein L3	2
XP_009689769.1	conserved hypothetical protein	2
XP_009690594.1	proteasome subunit y	2
XP_009690523.1	conserved hypothetical protein	2
XP_009689917.1	conserved hypothetical protein	2
XP_009692163.1	uncharacterized protein	2
XP_009689080.1	prefoldin subunit	2
XP_009691788.1	uncharacterized protein	2
XP_009689471.1	clathrin-adaptor chain	2
XP_009689913.1	metalloprotease/cell division cycle protein	2
XP_009690755.1	major facilitator superfamily MFS-1 protein	2
XP_009692030.1	serine/threonine kinase	2
XP_009690590.1	uncharacterized protein	2
XP_009692016.1	uncharacterized protein	2
XP_009690164.1	histone acethyltransferase-like protein	2
XP_009692120.1	conserved hypothetical protein	2
XP_009691717.1	uncharacterized protein	2
XP_009692249.1	conserved hypothetical protein	2
XP_009692599.1	conserved hypothetical protein	2
XP_009688909.1	conserved hypothetical protein	2
XP_009689744.1	mitochondrial inner membrane subunit	2
XP_009690196.1	uncharacterized protein	2
XP_009692004.1	conserved hypothetical protein	2
XP_009690958.1	conserved hypothetical protein	2
XP_009691669.1	inosine-5'-monophosphate dehydrogenase	2
XP_009690512.1	conserved hypothetical protein	2
XP_009692684.1	U5 snRNP-specific subunit	2
XP_009691303.1	uncharacterized protein	2
XP_009691059.1	uncharacterized protein	2
XP_009691568.1	uncharacterized protein	2

XP_009690261.1	conserved hypothetical protein	2
XP_009690389.1	uncharacterized protein	2
XP_009691231.1	conserved hypothetical protein	2
XP_009691903.1	uncharacterized protein	2
XP_009689968.1	uncharacterized protein	2
XP_009688916.1	uncharacterized protein	2
XP_009692581.1	vesicle transport protein	2
XP_009689638.1	conserved hypothetical protein	2
XP_009691606.1	uncharacterized protein	2
XP_009690035.1	conserved hypothetical protein	2
XP_009690311.1	conserved hypothetical protein	2
XP_009690072.1	inorganic pyrophosphatase	2
XP_009692683.1	26S proteasome ATPase subunit	2
XP_009690943.1	protein phosphatase 2C homolog 2	2
XP_009689058.1	T-complex protein 1 delta subunit	2
XP_009689461.1	minichromosome maintenance protein	2
XP_009689770.1	uncharacterized protein	2
XP_009690445.1	uncharacterized protein	2
XP_009690431.1	calcium-dependent protein kinase	2
XP_009691193.1	hypothetical protein	2
XP_009689412.1	uncharacterized protein	2
XP_009690880.1	conserved hypothetical protein	2
XP_009689849.1	uncharacterized protein	2
XP_009691728.1	conserved hypothetical protein	2
XP_009692574.1	conserved hypothetical protein	2
XP_009689422.1	diphthine synthase	2
XP_009690467.1	uncharacterized protein	2
XP_009690939.1	hypothetical protein	2
XP_009690871.1	hypothetical protein	2
XP_009689525.1	ribonucleotide reductase R2 subunit/ribonucleoside-diphosphate reductase small chain	2
XP_009692057.1	cell-cycle-related serine/threonine protein kinase	2
XP_009691072.1	conserved hypothetical protein	2
XP_009691484.1	uncharacterized protein	2
XP_009689615.1	uncharacterized protein	2
XP_009691874.1	hypothetical protein	2
XP_009692386.1	uncharacterized protein	2
XP_009692612.1	lipid-binding/transfer protein	2
XP_009692380.1	uncharacterized protein	2
XP_009690142.1	methionine-tRNA ligase	2

XP_009691221.1	conserved hypothetical protein	2
XP_009689521.1	conserved hypothetical protein	2
XP_009692703.1	leucine carboxyl methyltransferase	2
XP_009689221.1	conserved hypothetical protein	2
XP_009691061.1	ADP-ribosylation factor	2
XP_009689012.1	NifU protein	2
XP_009692104.1	16S rRNA processing protein	2
XP_009689654.1	GMP synthase	2
XP_009689756.1	uncharacterized protein	2
XP_009692452.1	splicing factor	2
XP_009690125.1	conserved hypothetical protein	2
XP_009691624.1	uncharacterized protein	2
XP_009692587.1	peptide chain release factor	2
XP_009692233.1	isocitrate dehydrogenase	2
XP_009689857.1	HR1 rho-binding repeat containing protein	2
XP_009690315.1	hypothetical protein	2
XP_009691148.1	conserved hypothetical protein	2
XP_009691494.1	uncharacterized protein	2
XP_009691688.1	Fasciclin-2-like protein	2
XP_009690416.1	uncharacterized protein	2
XP_009692660.1	conserved hypothetical protein	2
XP_009692814.1	ferredoxin	2
XP_009691811.1	cytochrome reductase	2
XP_009692293.1	predicted protein	2
XP_009692305.1	small GTPase	2
XP_009690816.1	conserved hypothetical protein	2
XP_009691368.1	glucose-6-phosphate/phosphate translocator	2
XP_009692534.1	coat protein%2C gamma subunit	2
XP_009689489.1	hypothetical protein	2
XP_009689713.1	DEAD-box family RNA helicase	2
XP_009690621.1	uncharacterized protein	2
XP_009689870.1	HAD-superfamily hydrolase%2C subfamily IIB protein	2
XP_009691076.1	uncharacterized protein	2
XP_009690713.1	calmodulin	2
XP_009690351.1	apurinic/apyrimidinic endonuclease	2
XP_009692716.1	hypothetical protein	2
XP_009691311.1	conserved hypothetical protein	2
XP_009690731.1	conserved hypothetical protein	2
XP_009690669.1	conserved hypothetical protein	2

XP_009690658.1	serine/threonine protein kinase	2
XP_009691236.1	aspartyl(acid) protease	2
XP_009690084.1	conserved hypothetical protein	2
XP_009689003.1	uncharacterized protein	2
XP_009690158.1	hypothetical protein	2
XP_009691557.1	peptidyl-prolyl cis-trans isomerase	2
XP_009688897.1	hypothetical protein	2
XP_009690515.1	vacuolar ATP synthase subunit D	2
XP_009692093.1	conserved hypothetical protein	2
XP_009692101.1	ribosomal protein S2	2
XP_009692580.1	uncharacterized protein	2
XP_009689482.1	conserved hypothetical protein	2
XP_009690135.1	uncharacterized protein	2
XP_009692658.1	uncharacterized protein	2
XP_009691044.1	hypothetical protein	2
XP_009690889.1	asparaginyl-tRNA synthetase	2
XP_009691650.1	conserved hypothetical protein	2
XP_009689358.1	hypothetical protein	2
XP_009692186.1	conserved hypothetical protein	2
XP_009688993.1	conserved hypothetical protein	2
XP_009688857.1	alkylated DNA repair protein	2
XP_009689279.1	conserved hypothetical protein	2
XP_009690282.1	50S ribosomal protein L9	2
XP_009690161.1	uncharacterized protein	2
XP_009691398.1	DNA mismatch repair protein	2
XP_009690537.1	uncharacterized protein	2
XP_009692174.1	histone H4	2
XP_009690841.1	uncharacterized protein	2
XP_009691330.1	uncharacterized protein	2
XP_009690472.1	conserved hypothetical protein	2
XP_009691224.1	RNA splicing factor	2
XP_009692141.1	enolase	2
XP_009690928.1	uncharacterized protein	2
XP_009689290.1	conserved hypothetical protein	2
XP_009691990.1	beta-tubulin cofactor D	2
XP_009689642.1	uncharacterized protein	2
XP_009692492.1	conserved hypothetical protein	2
XP_009690773.1	conserved hypothetical protein	2
XP_009690328.1	conserved hypothetical protein	2

XP_009691049.1	hypothetical protein	2
XP_009690892.1	ankyrin repeat containing protein	2
XP_009691299.1	conserved hypothetical protein	2
XP_009689391.1	conserved hypothetical protein	2
XP_009691861.1	26S proteasome regulatory subunit	2
XP_009690323.1	conserved hypothetical protein	2
XP_009692196.1	uncharacterized protein	2
XP_009691671.1	hypothetical protein	2
XP_009691086.1	conserved hypothetical protein	2
XP_009690628.1	peptidyl-prolyl cis-trans isomerase	2
XP_009692519.1	40S ribosomal protein S9	2
XP_009690912.1	uncharacterized protein	2
XP_009689310.1	conserved hypothetical protein	2
XP_009690151.1	conserved hypothetical protein	2
XP_009692570.1	RNA-binding protein Puf1	2
XP_009691832.1	conserved hypothetical protein	2
XP_009690648.1	uncharacterized protein	2
XP_009691690.1	snRNP splicing factor U2AF	2
XP_009692588.1	peptide chain release factor	2
XP_009691702.1	conserved hypothetical protein	2
XP_009692524.1	cell surface/extracellular protein	2
XP_009690204.1	serine/threonine protein kinase	2
XP_009691342.1	tRNA nucleotidyltransferase	2
XP_009690314.1	conserved hypothetical protein	2
XP_009690805.1	uncharacterized protein	2
XP_009692255.1	papain-family cysteine protease	2
XP_009692359.1	conserved hypothetical protein	1
XP_009691387.1	glucose-6-phosphate-1-dehydrogenase	1
XP_009692282.1	uncharacterized protein	1
XP_009690814.1	conserved hypothetical protein	1
XP_009690777.1	serine/threonine-protein kinase ripk4	1
XP_009689000.1	30S ribosomal protein S11	1
XP_009692762.1	predicted protein	1
XP_009692254.1	conserved hypothetical protein	1
XP_009691585.1	uncharacterized protein	1
XP_009692424.1	conserved hypothetical protein	1
XP_009691815.1	uncharacterized protein	1
XP_009689054.1	pre-rRNA processing protein	1
XP_009692010.1	conserved hypothetical protein	1

XP_009689253.1	aspartate transcarbamoylase	1
XP_009690165.1	conserved hypothetical protein	1
XP_009690798.1	conserved hypothetical protein	1
XP_009691576.1	hypothetical protein	1
XP_009690442.1	conserved hypothetical protein	1
XP_009692499.1	uncharacterized protein	1
XP_009690254.1	conserved hypothetical protein	1
XP_009689930.1	conserved hypothetical protein	1
XP_009689437.1	hypothetical protein	1
XP_009691897.1	uncharacterized protein	1
XP_009691555.1	uncharacterized protein	1
XP_009691295.1	hypothetical protein	1
XP_009692661.1	conserved hypothetical protein	1
XP_009691614.1	uncharacterized protein	1
XP_009691831.1	conserved hypothetical protein	1
XP_009690110.1	replication factor-A protein 1	1
XP_009692212.1	vacuolar protein sorting/secretion protein	1
XP_009690262.1	uncharacterized protein	1
XP_009692001.1	uncharacterized protein	1
XP_009691294.1	hypothetical protein	1
XP_009688928.1	uncharacterized protein	1
XP_009691288.1	GTP-binding protein	1
XP_009692539.1	26S proteasome subunit	1
XP_009690145.1	conserved hypothetical protein	1
XP_009690094.1	conserved hypothetical protein	1
XP_009691129.1	conserved hypothetical protein	1
XP_009691993.1	uncharacterized protein	1
XP_009689402.1	conserved hypothetical protein	1
XP_009691925.1	conserved hypothetical protein	1
XP_009692436.1	hypothetical protein	1
XP_009690367.1	uncharacterized protein	1
XP_009689514.1	conserved hypothetical protein	1
XP_009688873.1	hexokinase 1	1
XP_009689891.1	uncharacterized protein	1
XP_009692422.1	conserved hypothetical protein	1
XP_009688941.1	uncharacterized protein	1
XP_009692707.1	structure-specific recognition protein 1	1
XP_009691901.1	conserved hypothetical protein	1
XP_009689568.1	1-phosphatidylinositol-4%2C5-bisphosphate phosphodiesterase	1

XP_009691659.1	conserved hypothetical protein	1
XP_009690612.1	peptidyl-prolyl cis-trans isomerase	1
XP_009689948.1	ATPase	1
XP_009691684.1	conserved hypothetical protein	1
XP_009690269.1	hypothetical protein	1
XP_009690274.1	falcilysin	1
XP_009690208.1	glutaredoxin-like protein grla	1
XP_009691371.1	NADH dehydrogenase	1
XP_009689326.1	conserved hypothetical protein	1
XP_009692215.1	uncharacterized protein	1
XP_009690396.1	uncharacterized protein	1
XP_009691495.1	DEAD-box family helicase	1
XP_009692490.1	conserved hypothetical protein	1
XP_009690225.1	uncharacterized protein	1
XP_009690462.1	uncharacterized protein	1
XP_009689371.1	conserved hypothetical protein	1
XP_009692071.1	tryptophanyl-tRNA synthetase	1
XP_009692582.1	uncharacterized protein	1
XP_009692159.1	succinyl-CoA ligase [GDP-forming] alpha-chain%2C mitochondrial	1
XP_009689776.1	uncharacterized protein	1
XP_009689476.1	uncharacterized protein	1
XP_009692095.1	hypothetical protein	1
XP_009690055.1	histone deacetylase	1
XP_009689610.1	complexed with cef1p	1
XP_009692318.1	conserved hypothetical protein	1
XP_009692627.1	conserved hypothetical protein	1
XP_009690140.1	uncharacterized protein	1
XP_009689935.1	inositol phosphatase	1
XP_009690330.1	uncharacterized protein	1
XP_009690424.1	ribosomal protein L20	1
XP_009689666.1	hypothetical protein	1
XP_009690321.1	conserved hypothetical protein	1
XP_009692638.1	conserved hypothetical protein	1
XP_009689420.1	uncharacterized protein	1
XP_009692360.1	uncharacterized protein	1
XP_009688855.1	uncharacterized protein	1
XP_009689762.1	conserved hypothetical protein	1
XP_009690705.1	glycerol kinase	1
XP_009689459.1	uncharacterized protein	1

XP_009690999.1	metalloprotease	1
XP_009690277.1	conserved hypothetical protein	1
XP_009689094.1	hypothetical protein	1
XP_009690379.1	uncharacterized protein	1
XP_009691915.1	conserved hypothetical protein	1
XP_009688965.1	ATP-dependent RNA helicase	1
XP_009691329.1	conserved hypothetical protein	1
XP_009691355.1	uncharacterized protein	1
XP_009689195.1	conserved hypothetical protein	1
XP_009691905.1	ribosomal protein L15	1
XP_009689283.1	ribosomal protein S15	1
XP_009691651.1	uncharacterized protein	1
XP_009692694.1	hypothetical protein	1
XP_009689454.1	glycerophosphoryl diester phosphodiesterase	1
XP_009691124.1	uncharacterized protein	1
XP_009692390.1	uncharacterized protein	1
XP_009692170.1	RING-box protein 1a	1
XP_009692532.1	hypothetical protein	1
XP_009689238.1	conserved hypothetical protein	1
XP_009690253.1	conserved hypothetical protein	1
XP_009689579.1	uncharacterized protein	1
XP_009690162.1	DNA repair helicase	1
XP_009690945.1	conserved hypothetical protein	1
XP_009689469.1	conserved hypothetical protein	1
XP_009692113.1	conserved hypothetical protein	1
XP_009689919.1	conserved hypothetical protein	1
XP_009690718.1	uncharacterized protein	1
XP_009692217.1	conserved hypothetical protein	1
XP_009689828.1	conserved hypothetical protein	1
XP_009689924.1	conserved hypothetical protein	1
XP_009692460.1	MAC/perforin	1
XP_009692341.1	DNA helicase	1
XP_009690207.1	conserved hypothetical protein	1
XP_009691077.1	GPI anchor transamidase	1
XP_009689612.1	cyclophilin peptidyl-prolyl cis-trans isomerase protein	1
XP_009691430.1	methionine aminopeptidase	1
XP_009692317.1	uncharacterized protein	1
XP_009691346.1	palmitoyltransferase	1
XP_009688922.1	conserved hypothetical protein	1

XP_009689958.1	eukaryotic initiation factor-2 alpha kinase-A	1
XP_009689013.1	6-phosphogluconate dehydrogenase	1
XP_009691362.1	hypothetical protein	1
XP_009689541.1	conserved hypothetical protein	1
XP_009691138.1	conserved hypothetical protein	1
XP_009691480.1	uncharacterized protein	1
XP_009691119.1	uncharacterized protein	1
XP_009691161.1	DNA-directed RNA polymerase III	1
XP_009690996.1	uncharacterized protein	1
XP_009691969.1	S-adenosylmethionine synthetase	1
XP_009690728.1	hypothetical protein	1
XP_009692481.1	conserved hypothetical protein	1
XP_009691270.1	uncharacterized protein	1
XP_009692411.1	60S ribosomal subunit biogenesis protein	1
XP_009690487.1	uncharacterized protein	1
XP_009692723.1	signal peptidase	1
XP_009690096.1	60S ribosomal protein L5	1
XP_009692123.1	conserved hypothetical protein	1
XP_009692520.1	conserved hypothetical protein	1
XP_009689368.1	DHHC-containing protein 20	1
XP_009691629.1	conserved hypothetical protein	1
XP_009690058.1	serine/threonine protein phosphatase pp-x isozyme 1	1
XP_009692013.1	uncharacterized protein	1
XP_009692078.1	methionine aminopeptidase	1
XP_009689653.1	uncharacterized protein	1
XP_009691813.1	uncharacterized protein	1
XP_009691950.1	conserved hypothetical protein	1
XP_009689325.1	conserved hypothetical protein	1
XP_009692173.1	uncharacterized protein	1
XP_009689617.1	conserved hypothetical protein	1
XP_009690510.1	conserved hypothetical protein	1
XP_009692775.1	conserved hypothetical protein	1
XP_009692140.1	conserved hypothetical protein	1
XP_009692625.1	uncharacterized protein	1
XP_009691491.1	RNA processing factor	1
XP_009690466.1	purine nucleoside phosphorylase	1
XP_009692245.1	pyruvate kinase	1
XP_009692641.1	conserved hypothetical protein	1
XP_009690362.1	uncharacterized protein	1

XP_009691350.1	50S ribosomal protein L3	1
XP_009689062.1	conserved hypothetical protein	1
XP_009691099.1	conserved hypothetical protein	1
XP_009692379.1	RNA polymerase common subunit	1
XP_009691862.1	hypothetical protein	1
XP_009691486.1	hypothetical protein	1
XP_009692128.1	uncharacterized protein	1
XP_009692405.1	vacuolar ATP synthase	1
XP_009692811.1	50S ribosomal protein L16	1
XP_009692615.1	acetyl-coenzyme A synthetase	1
XP_009690698.1	conserved hypothetical protein	1
XP_009690224.1	conserved hypothetical protein	1
XP_009692240.1	translation elongation factor 1-alpha	1
XP_009690696.1	citrate synthase%2C mitochondrial precursor	1
XP_009689428.1	Dim1 protein	1
XP_009689183.1	serine hydroxymethyltransferase	1
XP_009688944.1	long-chain-fatty-acid--CoA ligase	1
XP_009691655.1	50S ribosomal protein L27	1
XP_009692393.1	uncharacterized protein	1
XP_009689630.1	uncharacterized protein	1
XP_009691527.1	conserved hypothetical protein	1
XP_009691548.1	ER lumen protein retaining receptor 1	1
XP_009690303.1	vesicle trafficking protein-like 1	1
XP_009690518.1	uncharacterized protein	1
XP_009689343.1	choline kinase	1
XP_009692590.1	hypothetical protein	1
XP_009690441.1	uncharacterized protein	1
XP_009691957.1	hypothetical protein	1
XP_009690917.1	ADP-ribosylation factor	1
XP_009689833.1	uncharacterized protein	1
XP_009689559.1	clathrin assembly protein	1
XP_009691613.1	uncharacterized protein	1
XP_009690764.1	conserved hypothetical protein	1
XP_009690771.1	ATP-dependent RNA helicase	1
XP_009691445.1	nucleosome assembly protein	1
XP_009692169.1	uncharacterized protein	1
XP_009689809.1	conserved hypothetical protein	1
XP_009689534.1	uncharacterized protein	1
XP_009691190.1	exon junction complex protein	1

XP_009691583.1	uncharacterized protein	1
XP_009690513.1	uncharacterized protein	1
XP_009689550.1	clathrin assembly protein	1
XP_009689173.1	tRNA (guanine-n-(7))-methyltransferase	1
XP_009690073.1	conserved hypothetical protein	1
XP_009692626.1	conserved hypothetical protein	1
XP_009690592.1	nucleolar protein	1
XP_009689255.1	hypothetical protein	1
XP_009689345.1	conserved hypothetical protein	1
XP_009689636.1	26S proteasome subunit 4	1
XP_009691652.1	seryl-tRNA synthetase	1
XP_009692718.1	conserved hypothetical protein	1
XP_009691272.1	conserved hypothetical protein	1
XP_009690278.1	conserved hypothetical protein	1
XP_009692415.1	uncharacterized protein	1
XP_009692776.1	conserved hypothetical protein	1
XP_009689889.1	peptidyl-prolyl cis-trans isomerase	1
XP_009689429.1	conserved hypothetical protein	1
XP_009692374.1	triosephosphate isomerase	1
XP_009691183.1	conserved hypothetical protein	1
XP_009689910.1	chloroplast ribosomal protein L1/50S ribosomal protein L10	1
XP_009689558.1	conserved hypothetical protein	1
XP_009691251.1	synaptic glycoprotein sc2	1
XP_009692672.1	GNAT-family N-acetyltransferase	1
XP_009692564.1	eukaryotic translation initiation factor	1
XP_009691275.1	methionine aminopeptidase	1
XP_009689994.1	uncharacterized protein	1
XP_009691851.1	40S ribosomal protein S27	1
XP_009692295.1	mitochondrial carrier protein	1
XP_009691865.1	hypothetical protein	1
XP_009688991.1	conserved hypothetical protein	1
XP_009692560.1	conserved hypothetical protein	1
XP_009692474.1	methylase	1
XP_009692038.1	microtubule-associated protein	1
XP_009688918.1	cytochrome C oxidase subunit 2a	1
XP_009692304.1	uncharacterized protein	1
XP_009690401.1	dual-specificity phosphatase	1
XP_009690754.1	uncharacterized protein	1
XP_009690838.1	hypothetical protein	1

XP_009690910.1	conserved hypothetical protein	1
XP_009689204.1	conserved hypothetical protein	1
XP_009689185.1	conserved hypothetical protein	1
XP_009691674.1	conserved hypothetical protein	1
XP_009688853.1	conserved hypothetical protein	1
XP_009689045.1	hypothetical protein	1
XP_009690821.1	chromatin assembly protein	1
XP_009691345.1	conserved hypothetical protein	1
XP_009689920.1	phosphatidylglycerophosphate synthase	1
XP_009689384.1	uncharacterized protein	1
XP_009689834.1	uncharacterized protein	1
XP_009692666.1	uncharacterized protein	1
XP_009691798.1	uncharacterized protein	1
XP_009689375.1	secretory protein	1
XP_009691435.1	conserved hypothetical protein	1
XP_009692774.1	uncharacterized protein	1
XP_009689757.1	conserved hypothetical protein	1
XP_009691308.1	hypothetical protein	1
XP_009692601.1	DNA repair protein Rad51	1
XP_009690059.1	histone	1
XP_009692395.1	uncharacterized protein	1
XP_009690350.1	predicted protein	1
XP_009689005.1	uncharacterized protein	1
XP_009689652.1	ubiquinol-cytochrome C reductase complex subunit-like protein	1
XP_009689205.1	conserved hypothetical protein	1
XP_009692510.1	50S ribosomal protein L14	1
XP_009688927.1	uncharacterized protein	1
XP_009690054.1	protein yippee-like 1	1
XP_009692419.1	conserved hypothetical protein	1
XP_009691198.1	uncharacterized protein	1
XP_009690448.1	conserved hypothetical protein	1
XP_009691310.1	ribosome biogenesis protein	1
XP_009689234.1	hypothetical protein	1
XP_009690283.1	conserved hypothetical protein	1
XP_009690215.1	proteasome (prosome%2C macropain) 26S subunit%2C non-ATPase%2C 14	1
XP_009689595.1	pre-mRNA splicing factor	1
XP_009692764.1	uncharacterized protein	1
XP_009689460.1	molecular chaperone DnaJ	1
XP_009689967.1	conserved hypothetical protein	1

XP_009691094.1	transcription factor	1
XP_009690789.1	conserved hypothetical protein	1
XP_009689890.1	ADP-ribosylation factor GTPase activating protein	1
XP_009690257.1	heat shock protein	1
XP_009689879.1	conserved hypothetical protein	1
XP_009689356.1	conserved hypothetical protein	1
XP_009692577.1	uncharacterized protein	1
XP_009691173.1	light induced protein like protein	1
XP_009689397.1	conserved hypothetical protein	1
XP_009691177.1	conserved hypothetical protein	1
XP_009692565.1	prenyltransferase	1
XP_009688976.1	DNA excision-repair helicase	1
XP_009689451.1	uncharacterized protein	1
XP_009692338.1	conserved hypothetical protein	1
XP_009691895.1	methyltransferase	1
XP_009689250.1	conserved hypothetical protein	1
XP_009690171.1	conserved hypothetical protein	1
XP_009689817.1	arginyl-tRNA synthetase	1
XP_009688847.1	uncharacterized protein	1
XP_009692432.1	conserved hypothetical protein	1
XP_009692592.1	uncharacterized protein	1
XP_009689671.1	uncharacterized protein	1
XP_009691406.1	conserved hypothetical protein	1
XP_009691787.1	hypothetical protein	1
XP_009689403.1	trehalose-6-phosphate synthase	1
XP_009691128.1	conserved hypothetical protein	1
XP_009691729.1	hypothetical protein	1
XP_009691216.1	protein kinase	1
XP_009689716.1	actin depolymerizing factor	1
XP_009690785.1	uncharacterized protein	1
XP_009689051.1	dynein light chain 1	1
XP_009689738.1	conserved hypothetical protein	1
XP_009692391.1	conserved hypothetical protein	1
XP_009692142.1	predicted protein	1
XP_009689339.1	conserved hypothetical protein	1
XP_009689839.1	60S ribosomal protein L2/L8	1
XP_009692687.1	hypothetical protein	1
XP_009689608.1	ADP-ribosylation factor GTPase activating protein	1
XP_009688931.1	conserved hypothetical protein	1

XP_009690169.1	conserved hypothetical protein	1
XP_009690842.1	conserved hypothetical protein	1
XP_009689878.1	eukaryotic translation initiation factor 3 subunit	1
XP_009690230.1	mitochondrial processing peptidase subunit alpha	1
XP_009689686.1	conserved hypothetical protein	1
XP_009688923.1	heat shock protein 70	1
XP_009690021.1	hypothetical protein	1
XP_009688950.1	uncharacterized protein	1
XP_009688966.1	conserved hypothetical protein	1
XP_009689874.1	elongation factor 2	1
XP_009690349.1	ATP-dependent RNA helicase	1
XP_009690577.1	hypothetical protein	1
XP_009690258.1	heat shock protein 90	1
XP_009688902.1	conserved hypothetical protein	1
XP_009691083.1	uncharacterized protein	1
XP_009690326.1	topoisomerase-related nucleotidyltransferase	1
XP_009690758.1	uncharacterized protein	1
XP_009691284.1	conserved hypothetical protein	1
XP_009689241.1	cytokine induced apoptosis inhibitor 1	1
XP_009690061.1	aspartate aminotransferase%2C cytoplasmic	1
XP_009692051.1	hypothetical protein	1
XP_009690672.1	uncharacterized protein	1
XP_009689360.1	conserved hypothetical protein	1
XP_009691033.1	hypothetical protein	1
XP_009689304.1	uncharacterized protein	1
XP_009689048.1	uncharacterized protein	1
XP_009690419.1	conserved hypothetical protein	1
XP_009690879.1	ubiquitin carboxyl-terminal hydrolase	1
XP_009692669.1	hypothetical protein	1
XP_009692740.1	T-complex protein 1 chaperonin	1
XP_009691703.1	Band 7-related protein	1
XP_009691987.1	phenylalanyl-tRNA synthetase	1
XP_009691302.1	conserved hypothetical protein	1
XP_009689611.1	uncharacterized protein	1
XP_009691158.1	conserved hypothetical protein	1
XP_009690763.1	uncharacterized protein	1
XP_009688874.1	uncharacterized protein	1
XP_009689819.1	uncharacterized protein	1
XP_009689432.1	conserved hypothetical protein	1

XP_009689609.1	uncharacterized protein	1
XP_009688925.1	uncharacterized protein	1
XP_009690812.1	uncharacterized protein	1
XP_009692407.1	uncharacterized protein	1
XP_009691025.1	40S ribosomal protein S8	1
XP_009690543.1	predicted protein	1
XP_009689217.1	conserved hypothetical protein	1
XP_009689267.1	phosphoenolpyruvate carboxykinase	1
XP_009690038.1	uncharacterized protein	1
XP_009692236.1	conserved hypothetical protein	1
XP_009691384.1	conserved hypothetical protein	1
XP_009691115.1	uncharacterized protein	1
XP_009689160.1	Map2 kinase	1
XP_009688981.1	uncharacterized protein	1
XP_009691104.1	conserved hypothetical protein	1
XP_009689486.1	phosphotyrosyl phosphatase activator protein	1
XP_009690082.1	conserved hypothetical protein	1
XP_009690850.1	hypothetical protein	1
XP_009691358.1	uncharacterized protein	1
XP_009689838.1	aconitate hydratase 3%2C mitochondrial precursor	1
XP_009692330.1	uncharacterized protein	1
XP_009689269.1	ribonucleoprotein	1
XP_009692002.1	RNase L inhibitor protein	1
XP_009688932.1	uncharacterized protein	1
XP_009692313.1	conserved hypothetical protein	1
XP_009690955.1	uncharacterized protein	1
XP_009690244.1	conserved hypothetical protein	1
XP_009689271.1	conserved hypothetical protein	1
XP_009689739.1	polyadenylate-binding protein	1
XP_009691999.1	uncharacterized protein	1
XP_009689017.1	hypothetical protein	1
XP_009689093.1	uncharacterized protein	1
XP_009689430.1	hypothetical protein	1
XP_009691114.1	4-hydroxybenzoate octaprenyltransferase	1
XP_009690393.1	conserved hypothetical protein	1
XP_009690078.1	uncharacterized protein	1
XP_009690617.1	conserved hypothetical protein	1
XP_009691469.1	dehydrodolichyl diphosphate synthase	1
XP_009691462.1	uncharacterized protein	1

XP_009691450.1	conserved hypothetical protein	1
XP_009691846.1	2-C-methyl-D-erythritol 4-phosphate cytidylyltransferase	1
XP_009692221.1	uncharacterized protein	1
XP_009690382.1	hydrolase	1
XP_009689528.1	cell division control protein 2 homolog	1
XP_009690757.1	major facilitator superfamily MFS-1 protein	1
XP_009689925.1	conserved hypothetical protein	1
XP_009691178.1	conserved hypothetical protein	1
XP_009690607.1	conserved hypothetical protein	1
XP_009691407.1	conserved hypothetical protein	1
XP_009689490.1	uncharacterized protein	1
XP_009689896.1	uncharacterized protein	1
XP_009689117.1	conserved hypothetical protein	1
XP_009690919.1	DNA primase small subunit	1
XP_009692022.1	uncharacterized protein	1
XP_009690124.1	conserved hypothetical protein	1
XP_009692719.1	uncharacterized protein	1
XP_009689587.1	actin	1
XP_009689464.1	ubiquitin	1
XP_009691775.1	conserved hypothetical protein	1
XP_009688961.1	chromatin assembly factor 1 protein	1
XP_009692302.1	uncharacterized protein	1
XP_009691475.1	conserved hypothetical protein	1
XP_009690925.1	hypothetical protein	1
XP_009689259.1	uncharacterized protein	1
XP_009690533.1	autophagy-related peptidase	1
XP_009692585.1	uncharacterized protein	1
XP_009692495.1	GTPase-activating protein	1
XP_009692106.1	conserved hypothetical protein	1
XP_009691634.1	Conserved hypothetical protein	1
XP_009692397.1	conserved hypothetical protein	1
XP_009691376.1	major facilitator superfamily MFS-1 protein	1
XP_009690549.1	conserved hypothetical protein	1
XP_009689416.1	transcription initiation factor	1
XP_009689999.1	uncharacterized protein	1
XP_009691561.1	uncharacterized protein	1
XP_009689193.1	uncharacterized protein	1
XP_009691962.1	uncharacterized protein	1
XP_009691170.1	hypothetical protein	1

XP_009691598.1	conserved hypothetical protein	1
XP_009692400.1	uncharacterized protein	1
XP_009688915.1	conserved hypothetical protein	1
XP_009690033.1	hypothetical protein	1
XP_009692138.1	uncharacterized protein	1
XP_009692041.1	uncharacterized protein	1
XP_009691766.1	uncharacterized protein	1
XP_009690852.1	conserved hypothetical protein	1
XP_009690309.1	uncharacterized protein	1
XP_009688975.1	hypothetical protein	1
XP_009690427.1	uncharacterized protein	1
XP_009690633.1	transmembrane protein 17	1
XP_009689385.1	conserved hypothetical protein	1
XP_009690591.1	conserved hypothetical protein	1
XP_009691826.1	conserved hypothetical protein	1
XP_009691127.1	conserved hypothetical protein	1
XP_009689868.1	conserved hypothetical protein	1
XP_009689041.1	uncharacterized protein	1
XP_009689694.1	conserved hypothetical protein	1
XP_009692055.1	splicing component	1
XP_009692036.1	conserved hypothetical protein	1
XP_009691515.1	membrane transporter	1
XP_009692604.1	uncharacterized protein	1
XP_009692546.1	conserved hypothetical protein	1
XP_009691827.1	conserved hypothetical protein	1
XP_009692171.1	uncharacterized protein	1
XP_009690778.1	ABC transporter	1
XP_009691024.1	conserved hypothetical protein	1
XP_009690481.1	uncharacterized protein	1
XP_009692441.1	uncharacterized protein	1
XP_009692110.1	mitochondrial large subunit ribosomal protein	1
XP_009689069.1	uncharacterized protein	1
XP_009692278.1	uncharacterized protein	1
XP_009692032.1	Plasmodium falciparum CPW-WPC repeat containing protein	1
XP_009691352.1	U6 snRNA-associated sm-like protein lsm4	1
XP_009692260.1	mitochondrial carrier protein	1
XP_009692616.1	importin beta/transportin	1
XP_009688861.1	molecular chaperone DnaJ	1
XP_009690182.1	5'-3' exoribonuclease 2	1

XP_009691118.1	conserved hypothetical protein	1
XP_009690188.1	conserved hypothetical protein	1
XP_009692344.1	conserved hypothetical protein	1
XP_009689803.1	RNA pseudouridylate synthase	1
XP_009691088.1	cytochrome c oxidase copper chaperone%2C Dopuin	1
XP_009691896.1	endonuclease	1
XP_009691304.1	conserved hypothetical protein	1
XP_009689614.1	uncharacterized protein	1
XP_009690484.1	uncharacterized protein	1
XP_009690706.1	membrane skeletal protein IMC1	1
XP_009691187.1	T-complex protein subunit beta	1
XP_009689551.1	conserved hypothetical protein	1
XP_009692280.1	conserved hypothetical protein	1
XP_009691774.1	hypothetical protein	1
XP_009691337.1	conserved hypothetical protein	1
XP_009691508.1	conserved hypothetical protein	1
XP_009691274.1	uncharacterized protein	1
XP_009689989.1	RNA-binding protein	1
XP_009692425.1	tRNA 3' processing endoribonuclease	1
XP_009692657.1	molecular chaperone ClpB	1
XP_009688900.1	importin subunit beta-1	1
XP_009689865.1	calmodulin-like domain protein kinase	1
XP_009691884.1	conserved hypothetical protein	1
XP_009690418.1	uncharacterized protein	1
XP_009689333.1	uncharacterized protein	1
XP_009688912.1	vesicle transport protein	1
XP_009690743.1	TatD-like deoxyribonuclease	1
XP_009689216.1	uncharacterized protein	1
XP_009690900.1	dolichol phosphate mannose synthase	1
XP_009689869.1	uncharacterized protein	1
XP_009690423.1	uncharacterized protein	1
XP_009692222.1	uncharacterized protein	1
XP_009689673.1	casein kinase II subunit beta	1
XP_009689150.1	uncharacterized protein	1
XP_009690150.1	conserved hypothetical protein	1
XP_009689404.1	conserved hypothetical protein	1
XP_009689086.1	MORN motif repeat containing protein	1
XP_009692648.1	conserved hypothetical protein	1
XP_009690358.1	uncharacterized protein	1

XP_009689512.1	uncharacterized protein	1
XP_009691071.1	uncharacterized protein	1
XP_009690205.1	uncharacterized protein	1
XP_009689783.1	thrombospondin%2C type I repeat containing protein	1
XP_009689049.1	conserved hypothetical protein	1
XP_009690307.1	conserved hypothetical protein	1
XP_009691825.1	uncharacterized protein	1
XP_009689979.1	lipoate-protein ligase	1
XP_009692396.1	conserved hypothetical protein	1
XP_009691997.1	signal recognition particle subunit	1
XP_009692709.1	hypothetical protein	1
XP_009688914.1	RNA-processing protein	1
XP_009691960.1	conserved hypothetical protein	1
XP_009692818.1	conserved hypothetical protein	1
XP_009691314.1	uncharacterized protein	1
XP_009691911.1	uncharacterized protein	1
XP_009690575.1	conserved hypothetical protein	1
XP_009691822.1	fumarate hydratase class I	1
XP_009691723.1	hypothetical protein	1
XP_009689165.1	conserved hypothetical protein	1
XP_009689812.1	DEAD-box family helicase	1
XP_009690167.1	hypothetical protein	1
XP_009689186.1	uncharacterized protein	1
XP_009690044.1	conserved hypothetical protein	1
XP_009690027.1	conserved hypothetical protein	1
XP_009689988.1	uncharacterized protein	1
XP_009692375.1	conserved hypothetical protein	1
XP_009690589.1	conserved hypothetical protein	1
XP_009690468.1	uncharacterized protein	1
XP_009692352.1	conserved hypothetical protein	1
XP_009692685.1	conserved hypothetical protein	1
XP_009689982.1	vesicle transport protein	1
XP_009689656.1	conserved hypothetical protein	1
XP_009689118.1	molecular chaperone	1
XP_009692267.1	mRNA turnover/deadenylation component	1
XP_009691169.1	uncharacterized protein	1
XP_009692361.1	putative 60S Ribosomal protein L44	1
XP_009691809.1	uncharacterized protein	1
XP_009689447.1	uncharacterized protein	1

XP_009692693.1	conserved hypothetical protein	1
XP_009690354.1	conserved hypothetical protein	1
XP_009692060.1	GTPase	1
XP_009689081.1	conserved hypothetical protein	1
XP_009692809.1	conserved hypothetical protein	1
XP_009689307.1	uncharacterized protein	1
XP_009688935.1	conserved hypothetical protein	1
XP_009692504.1	uncharacterized protein	1
XP_009689176.1	deoxyuridine 5'-triphosphate nucleotidohydrolase	1
XP_009690853.1	conserved hypothetical protein	1
XP_009692369.1	lysyl-tRNA synthetase	1
XP_009691080.1	hypothetical protein	1
XP_009688940.1	peptidylprolyl isomerase	1
XP_009691533.1	cysteine proteinase	1
XP_009692294.1	mitochondrial carrier protein	1
XP_009690679.1	uncharacterized protein	1
XP_009691712.1	uncharacterized protein	1
XP_009691252.1	uncharacterized protein	1
XP_009689034.1	conserved hypothetical protein	1
XP_009690495.1	conserved hypothetical protein	1
XP_009690463.1	uncharacterized protein	1
XP_009692381.1	uncharacterized protein	1
XP_009691097.1	conserved hypothetical protein	1
XP_009691154.1	60S ribosomal protein L15	1
XP_009691082.1	uncharacterized protein	1
XP_009689444.1	conserved hypothetical protein	1
XP_009689014.1	actin	1
XP_009688883.1	DEAD-box family RNA helicase	1
XP_009689600.1	conserved hypothetical protein	1
XP_009689723.1	conserved hypothetical protein	1
XP_009690760.1	vacuolar-protein sorting-associated protein	1
XP_009691064.1	farnesyl pyrophosphate synthetase	1
XP_009689887.1	XPA binding protein 1	1
XP_009689672.1	protein kinase	1
XP_009691184.1	conserved hypothetical protein	1
XP_009692488.1	conserved hypothetical protein	1
XP_009690856.1	conserved hypothetical protein	1
XP_009689161.1	uncharacterized protein	1
XP_009689536.1	conserved hypothetical protein	1

XP_009692320.1	mannosyltransferase	1
XP_009688871.1	glucokinase	1
XP_009689240.1	glycyl-tRNA synthetase	1
XP_009690937.1	uncharacterized protein	1
XP_009691899.1	conserved hypothetical protein	1
XP_009692536.1	dynamin	1
XP_009690796.1	uncharacterized protein	1
XP_009692766.1	uncharacterized protein	1
XP_009690001.1	uncharacterized protein	1
XP_009690695.1	uncharacterized protein	1
XP_009692697.1	glucose-inhibited division protein a-like protein	1
XP_009689932.1	acylphosphatase	1
XP_009690967.1	26S proteasome ATPase subunit	1
XP_009691405.1	hypothetical protein	1
XP_009691269.1	cobalamin synthesis protein	1
XP_009689655.1	protein disulfide isomerase	1
XP_009689801.1	uncharacterized protein	1
XP_009689126.1	serine protease	1
XP_009692024.1	proteasome component	1
XP_009688947.1	tRNA wybutosine-synthesizing protein 1 homolog	1
XP_009690070.1	glycylpeptide N-tetradecanoyltransferase 1	1
XP_009690525.1	ATP-dependent RNA helicase	1
XP_009688964.1	histidyl-tRNA synthetase	1
XP_009688982.1	seryl-tRNA synthetase	1
XP_009692421.1	conserved hypothetical protein	1
XP_009689346.1	transcription elongation factor	1
XP_009690776.1	initiation factor 4E	1
XP_009689848.1	CDC5-like	1
XP_009692086.1	uncharacterized protein	1
XP_009691267.1	60S ribosomal protein L35a	1
XP_009691760.1	conserved hypothetical protein	1
XP_009691317.1	conserved hypothetical protein	1
XP_009690319.1	beta adaptin	1
XP_009689620.1	uncharacterized protein	1
XP_009691694.1	hypothetical protein	1
XP_009692378.1	vesicle-associated membrane protein	1
XP_009690400.1	Mg protoporphyrin IX chelatase	1
XP_009691408.1	uncharacterized protein	1
XP_009689141.1	uncharacterized protein	1

XP_009689963.1	pre-mRNA splicing protein	1
XP_009690373.1	ribosomal protein S9/S16	1
XP_009690359.1	conserved hypothetical protein	1
XP_009688911.1	protein phosphatase 2A regulatory subunit B	1
XP_009691389.1	uncharacterized protein	1
XP_009690390.1	hypothetical protein	1
XP_009691609.1	eukaryotic initiation factor	1
XP_009691244.1	Ras-related GTPase	1
XP_009692061.1	uncharacterized protein	1
XP_009689387.1	hypothetical protein	1
XP_009689841.1	hypothetical protein	1
XP_009688903.1	uncharacterized protein	1
XP_009692329.1	elongation factor subunit	1
XP_009690042.1	conserved hypothetical protein	1
XP_009692366.1	phosphorylase phosphatase	1
XP_009689020.1	60S ribosomal protein L13	1
XP_009691020.1	serine/threonine protein phosphatase	1
XP_009691006.1	conserved hypothetical protein	1
XP_009690989.1	formate/nitrate transporter	1
XP_009690998.1	phosphoglucosamine mutase	1
XP_009692355.1	hypothetical protein	1
XP_009689647.1	uncharacterized protein	1
XP_009690780.1	hypothetical protein	1
XP_009692165.1	PRL1 protein	1
XP_009690412.1	uncharacterized protein	1
XP_009691778.1	ADP-ribosylation factor	1
XP_009690905.1	CTP:phosphorylcholine cytidylyltransferase	1
XP_009692713.1	uncharacterized protein	1
XP_009689194.1	conserved hypothetical protein	1
XP_009692084.1	conserved hypothetical protein	1
XP_009688933.1	uncharacterized protein	1
XP_009690043.1	conserved hypothetical protein	1
XP_009689309.1	conserved transmembrane protein	1
XP_009689902.1	conserved hypothetical protein	1
XP_009690071.1	uncharacterized protein	1
XP_009690870.1	hypothetical protein	1
XP_009690742.1	conserved hypothetical protein	1
XP_009689075.1	dihydrolipoamide succinyltransferase component of 2-oxoglutarate dehydrogenase complex%2C mitochondrial precursor	1
XP_009690233.1	hypothetical protein	1

XP_009690873.1	conserved hypothetical protein	1
XP_009692522.1	uncharacterized protein	1
XP_009691639.1	Box C/D snoRNA protein	1
XP_009690663.1	splicing factor 3a subunit 2	1
XP_009689472.1	26S proteasome subunit	1
XP_009689026.1	uncharacterized protein	1
XP_009689282.1	uncharacterized protein	1
XP_009691706.1	T-complex protein 1 alpha subunit	1
XP_009691498.1	conserved hypothetical protein	1
XP_009689233.1	conserved hypothetical protein	1
XP_009691513.1	transcription factor IIIb subunit	1
XP_009692617.1	thioredoxin	1
XP_009691416.1	hypothetical protein	1
XP_009689091.1	developmentally regulated GTP-binding protein 1	1
XP_009691519.1	protein translocation complex subunit gamma chain	1
XP_009689823.1	nucleolar phosphoprotein	1
XP_009690138.1	uncharacterized protein	1
XP_009690388.1	conserved hypothetical protein	1
XP_009690578.1	hypothetical protein	1
XP_009689908.1	uncharacterized protein	1
XP_009691900.1	putative RPC10 subunit of RNA polymerases I%2C II%2C and III	1
XP_009691457.1	spliceosome-associated protein	1
XP_009690288.1	uncharacterized protein	1
XP_009690407.1	splicing factor 3b subunit 4	1
XP_009690223.1	conserved hypothetical protein	1
XP_009691333.1	uncharacterized protein	1
XP_009692326.1	conserved hypothetical protein	1
XP_009688954.1	conserved hypothetical protein	1
XP_009692323.1	conserved hypothetical protein	1
XP_009690332.1	60S ribosomal protein L27a	1
XP_009692285.1	DEAD-box family RNA-dependent helicase	1
XP_009690394.1	Diphthamide synthesis protein	1
XP_009689124.1	conserved hypothetical protein	1
XP_009691147.1	conserved hypothetical protein	1
XP_009690187.1	N-ethylmaleimide-sensitive factor	1
XP_009689538.1	conserved hypothetical protein	1
XP_009691658.1	sulfur metabolism negative regulator	1
XP_009690795.1	conserved hypothetical protein	1
XP_009692437.1	uncharacterized protein	1

XP_009692608.1	uncharacterized protein	1
XP_009691709.1	conserved hypothetical protein	1
XP_009689914.1	conserved hypothetical protein	1
