# Supplementary material for: Analysis of Theileria orientalis draft genome sequences reveals potential species-level divergence of the Ikeda, Chitose and Buffeli genotypes
Source: BMC Genomics. 2018 Apr 27;19:298. doi: 10.1186/s12864-018-4701-2 (PMC5921998; doi:10.1186/s12864-018-4701-2)
Supplement: Supplementary file 7 — Coding sequence average depth of coverage. Average coverage depth of coding sequences for orthogroups grouped by genes per isolate (Gene numbers containing > 4 orthogroups not shown). (DOC 29 kb) [file 12864_2018_4701_MOESM7_ESM.doc]

Additional File 7. Average coverage depth of coding sequences for orthogroups grouped by genes per isolate (Gene numbers containing >4 orthogroups not shown).

|  | Number of genes per- |  | Average coding sequence coverage |  |
| --- | --- | --- | --- | --- |
|  | isolate in orthogroup | Robertson | Fish Creek | Goon Nure |
|  | 1 | 54.9x | 63.6x | 75.9x |
|  | 2 | 55.0x | 59.0x | 51.9x |
|  | 3 | 65.2x | 65.9x | 43.0x |
|  | 4 | 54.4x | 75.6x | 37.4x |
