# Supplementary material for: Analysis of Theileria orientalis draft genome sequences reveals potential species-level divergence of the Ikeda, Chitose and Buffeli genotypes
Source: BMC Genomics. 2018 Apr 27;19:298. doi: 10.1186/s12864-018-4701-2 (PMC5921998; doi:10.1186/s12864-018-4701-2)
Supplement: Supplementary file 9 — Predicted recombination events. Details of predicted recombination events shown in Fig. 2. (PDF 30 kb) [file 12864_2018_4701_MOESM9_ESM.pdf]

Sequence	Chromosome	Start	End	Size	Major.parent	Minor.parent
Goon Nure	chr1	1451809	1454775	2966	Fish Creek	Shintoku
Fish Creek	chr1	2098416	2099863	1447	Goon Nure	Unknown
Fish Creek	chr1	1082071	1083058	987	Goon Nure	Shintoku
Fish Creek	chr1	41550	42295	745	Goon Nure	Unknown
Goon Nure	chr1	91823	92763	940	Fish Creek	Robertson
Goon Nure	chr1	2156430	2157315	885	Fish Creek	Robertson
Goon Nure	chr1	1838526	1840037	1511	Fish Creek	Robertson
Goon Nure	chr1	714190	716230	2040	Fish Creek	Shintoku
Fish Creek	chr1	316373	316771	398	Goon Nure	Unknown
Goon Nure	chr1	1674917	1676204	1287	Fish Creek	Shintoku
Goon Nure	chr1	116061	117503	1442	Fish Creek	Robertson
Fish Creek	chr1	2308036	2308378	342	Goon Nure	Robertson
Goon Nure	chr1	1941231	1941890	659	Fish Creek	Robertson
Goon Nure	chr1	205145	205574	429	Fish Creek	Shintoku
Fish Creek	chr1	727474	727849	375	Goon Nure	Robertson
Fish Creek	chr1	1440991	1441148	157	Goon Nure	Robertson
Fish Creek	chr1	2407669	2408989	1320	Goon Nure	Robertson
Fish Creek	chr1	215317	218922	3605	Goon Nure	Robertson
Shintoku	chr1	319556	323352	3796	Unknown	Fish Creek
Robertson	chr1	1856634	1856688	54	Shintoku	Goon Nure
Goon Nure	chr1	676806	678640	1834	Fish Creek	Robertson
Goon Nure	chr1	1537796	1539566	1770	Fish Creek	Robertson
Goon Nure	chr1	1686212	1686626	414	Fish Creek	Robertson
Goon Nure	chr1	751621	751921	300	Fish Creek	Robertson
Goon Nure	chr1	2065106	2066293	1187	Fish Creek	Shintoku
Fish Creek	chr1	307696	307965	269	Goon Nure	Shintoku
Goon Nure	chr1	2491831	2493103	1272	Fish Creek	Shintoku
Robertson	chr2	1325451	1333601	8150	Shintoku	Goon Nure
Goon Nure	chr2	1672054	1673509	1455	Fish Creek	Robertson
Goon Nure	chr2	479350	480339	989	Fish Creek	Unknown
Goon Nure	chr2	745340	745996	656	Fish Creek	Robertson
Fish Creek	chr2	20354	23856	3502	Goon Nure	Unknown
Goon Nure	chr2	1771012	1772990	1978	Fish Creek	Robertson
Shintoku	chr2	1433021	1433361	340	Robertson	Goon Nure
Goon Nure	chr2	1888440	1889263	823	Fish Creek	Robertson
Goon Nure	chr2	1861634	1862192	558	Fish Creek	Robertson
Goon Nure	chr2	1984472	1986490	2018	Fish Creek	Shintoku
Goon Nure	chr2	1477327	1479866	2539	Fish Creek	Robertson
Goon Nure	chr2	962335	962791	456	Fish Creek	Robertson
Goon Nure	chr2	326111	327144	1033	Fish Creek	Robertson
Goon Nure	chr2	1590677	1591962	1285	Fish Creek	Robertson
Goon Nure	chr2	1897168	1897490	322	Fish Creek	Robertson
Fish Creek	chr2	1813568	1818799	5231	Goon Nure	Shintoku
Goon Nure	chr2	1294598	1296044	1446	Fish Creek	Shintoku
Fish Creek	chr2	1377299	1378742	1443	Goon Nure	Unknown
Goon Nure	chr2	1830804	1833029	2225	Fish Creek	Shintoku
Fish Creek	chr2	660356	664101	3745	Goon Nure	Shintoku
Goon Nure	chr2	1433402	1434655	1253	Fish Creek	Robertson
Shintoku	chr3	1564972	1565528	556	Robertson	Unknown

Fish Creek	chr3	1286048	1286546	498	Goon Nure	Unknown
Goon Nure	chr3	1612476	1614894	2418	Fish Creek	Robertson
Goon Nure	chr3	584745	585818	1073	Fish Creek	Shintoku
Fish Creek	chr3	558983	559465	482	Goon Nure	Unknown
Goon Nure	chr3	786421	788910	2489	Fish Creek	Shintoku
Goon Nure	chr3	1546309	1547010	701	Fish Creek	Robertson
Goon Nure	chr3	1646511	1648568	2057	Fish Creek	Shintoku
Fish Creek	chr3	866236	867012	776	Goon Nure	Robertson
Goon Nure	chr3	355452	356016	564	Fish Creek	Robertson
Goon Nure	chr3	921574	922558	984	Fish Creek	Robertson
Goon Nure	chr3	612092	613576	1484	Fish Creek	Shintoku
Goon Nure	chr3	794854	800825	5971	Fish Creek	Shintoku
Goon Nure	chr3	1532485	1538326	5841	Fish Creek	Robertson
Goon Nure	chr3	1911780	1913432	1652	Fish Creek	Robertson
Shintoku	chr3	204954	204984	30	Robertson	Unknown
Shintoku	chr3	1068475	1072242	3767	Unknown	Fish Creek
Goon Nure	chr4	173747	174012	265	Fish Creek	Unknown
Goon Nure	chr4	1514482	1515110	628	Fish Creek	Shintoku
Goon Nure	chr4	498676	499378	702	Fish Creek	Robertson
Goon Nure	chr4	1661061	1661948	887	Fish Creek	Shintoku
Goon Nure	chr4	1001978	1002892	914	Fish Creek	Shintoku
Goon Nure	chr4	42779	56600	13821	Fish Creek	Robertson
Fish Creek	chr4	427485	428125	640	Goon Nure	Unknown
Goon Nure	chr4	829434	830967	1533	Fish Creek	Robertson
Fish Creek	chr4	1238780	1239234	454	Goon Nure	Robertson
Fish Creek	chr4	345420	346221	801	Goon Nure	Robertson
Goon Nure	chr4	974201	975790	1589	Fish Creek	Robertson
Goon Nure	chr4	1561764	1562180	416	Fish Creek	Shintoku
Goon Nure	chr4	141542	142525	983	Fish Creek	Shintoku
Goon Nure	chr4	439828	442363	2535	Fish Creek	Shintoku
Fish Creek	chr4	5245	8483	3238	Goon Nure	Robertson
Goon Nure	chr4	1432823	1433429	606	Fish Creek	Shintoku
Fish Creek	chr4	1193984	1194108	124	Goon Nure	Robertson
Fish Creek	chr4	471996	472894	898	Goon Nure	Shintoku
