# Supplementary material for: Analysis of Theileria orientalis draft genome sequences reveals potential species-level divergence of the Ikeda, Chitose and Buffeli genotypes
Source: BMC Genomics. 2018 Apr 27;19:298. doi: 10.1186/s12864-018-4701-2 (PMC5921998; doi:10.1186/s12864-018-4701-2)
Supplement: Supplementary file 10 — dN/dS ratios. Calculated dN/dS (a.k.a. KaKs) ratios for the predicted Shintoku proteome by comparison to mapped Fish Creek (Chitose) and Goon Nure (Buffeli) isolate sequences. (PDF 913 kb) [file 12864_2018_4701_MOESM10_ESM.pdf]

protein_id	Chitose_ka	Chitose_ks	Chitose_kaks	Buffeli_ka	Buffeli_ks	Buffeli_kaks	mean_kaks	function
XP_009688834.1				NA	NA	NA	NA	conserved hypothetical protein
XP_009688837.1	0.0310876	0.278964	0.11144	0.0457958	0.458896	0.0997956	0.105618	conserved hypothetical protein
XP_009688840.1	0.0340762	0.304461	0.111923				0.111923	ABC transporter
XP_009688841.1	0.106194	0.401843	0.264267	0.0722702	0.290093	0.249128	0.256697	conserved hypothetical protein
XP_009688842.1	0.056748	0.314502	0.180438	0.0246136	0.288844	0.0852143	0.132826	protein kinase C inhibitor
XP_009688843.1	0.0136872	0.47378	0.0288894	0.00725252	0.363064	0.0199759	0.024433	exosome complex exonuclease
XP_009688844.1	0.0217719	0.349641	0.0622694	0.0222697	0.427824	0.0520533	0.057161	uncharacterized protein
XP_009688845.1	0.0387495	0.530712	0.0730142	0.0416433	0.576754	0.0722029	0.072609	uncharacterized protein
XP_009688846.1	0.0101654	0.6159	0.0165049	0.0131432	0.601815	0.0218393	0.019172	sortilin
XP_009688847.1	0.0329515	0.352508	0.0934773	0.0193105	0.367486	0.0525476	0.073012	uncharacterized protein
XP_009688848.1	0.0189937	0.239981	0.0791467	0.0177178	0.139542	0.126972	0.103059	uncharacterized protein
XP_009688849.1	0.0230915	0.48847	0.0472731	0.0127578	0.451549	0.0282535	0.037763	conserved hypothetical protein
XP_009688850.1	0.0783345	0.687547	0.113933	0.0684085	0.607615	0.112585	0.113259	conserved hypothetical protein
XP_009688851.1	0.0494115	0.837348	0.0590096	0.0541606	0.960162	0.0564077	0.057709	cysteine desulfurase
XP_009688852.1	0.0626902	0.477327	0.131336	0.0583182	0.506189	0.11521	0.123273	uncharacterized protein
XP_009688853.1	0.129551	0.467954	0.276846	0.111737	0.393349	0.284065	0.280455	conserved hypothetical protein
XP_009688854.1	0.0406743	0.790417	0.0514593	0.0574665	1.67464	0.0343157	0.042887	conserved hypothetical protein
XP_009688855.1	0.0259317	0.445566	0.0581994	0.0329958	0.403561	0.0817615	0.06998	uncharacterized protein
XP_009688856.1	0.0112306	0.479721	0.0234108	0.0119812	0.456429	0.0262499	0.02483	DNA-directed RNA polymerase II
XP_009688857.1	0.0536089	0.44808	0.119641	0.0271315	0.513331	0.0528538	0.086247	alkylated DNA repair protein
XP_009688858.1	0.0351281	0.874634	0.0401632	0.0357038	1.10487	0.032315	0.036239	aspartyl/glutamyl-tRNA amido transferase
XP_009688859.1	0.153251	0.516643	0.296629	0.189632	0.679226	0.279188	0.287909	conserved hypothetical protein
XP_009688860.1	0.114608	0.623239	0.183891	0.116518	0.570758	0.204147	0.194019	conserved hypothetical protein
XP_009688861.1	0.0361204	0.470733	0.0767322	0.0412804	0.564063	0.073184	0.074958	molecular chaperone DnaJ
XP_009688862.1	0.0690884	0.437872	0.157782	0.0540272	0.352899	0.153095	0.155439	conserved hypothetical protein
XP_009688863.1	0.0566892	0.340405	0.166534	0.045067	0.328192	0.137319	0.151926	polyadenylate polymerase
XP_009688864.1	0.141896	0.4667	0.304041	0.134513	0.45738	0.294094	0.299068	conserved hypothetical protein
XP_009688865.1	0.00594987	0.55747	0.010673	0.00556416	0.445562	0.012488	0.011581	U3 small nucleolar ribonucleoprotein
XP_009688866.1	0.0410959	0.290059	0.141681	0.0287822	0.316556	0.0909227	0.116302	conserved hypothetical protein
XP_009688867.1	0.0273192	0.447454	0.0610546	0.0323484	0.576018	0.0561586	0.058607	ribonuclease
XP_009688868.1	0.0744829	0.613432	0.12142	0.0597005	0.580673	0.102813	0.112117	conserved hypothetical protein
XP_009688873.1	0.0275627	0.425916	0.064714	0.0170015	0.552083	0.0307952	0.047755	hexokinase 1
XP_009688874.1	0.084219	0.283504	0.297064	0.0239186	0.298059	0.0802478	0.188656	uncharacterized protein
XP_009688875.1	0.113167	0.322941	0.350426	0.122565	0.306177	0.400308	0.375367	conserved hypothetical protein
XP_009688876.1	0.0648791	0.374056	0.173447	0.0561653	0.434244	0.129341	0.151394	conserved hypothetical protein
XP_009688877.1	0.0268998	0.546322	0.0492379	0.0311625	0.470243	0.0662688	0.057753	uncharacterized protein
XP_009688878.1	0.0366649	0.509908	0.0719049	0.0289544	0.50246	0.0576252	0.064765	conserved hypothetical protein
XP_009688879.1	0.0410386	0.395236	0.103833	0.0567628	0.435385	0.130374	0.117103	conserved hypothetical protein
XP_009688880.1	0.0697737	0.577296	0.120863	0.0661339	0.629316	0.105089	0.112976	uncharacterized protein
XP_009688881.1	0.0277501	0.515803	0.0537998	0.0266165	0.628464	0.0423516	0.048076	uncharacterized protein
XP_009688882.1	0.0402059	0.403552	0.09963	0.0461565	0.321265	0.143671	0.12165	GTPase
XP_009688883.1	0.0370876	0.527477	0.0703112	0.0266167	0.485817	0.0547875	0.062549	DEAD-box family RNA helicase
XP_009688884.1	0.113087	0.474629	0.238264	0.0839756	0.40461	0.207547	0.222906	conserved hypothetical protein
XP_009688885.1	0.0518566	0.304487	0.170308	0.0565249	0.204619	0.276245	0.223276	uncharacterized protein
XP_009688886.1	0.0472784	0.440489	0.107332	0.0404908	0.741575	0.0546011	0.080967	conserved hypothetical protein
XP_009688887.1	0.0442921	0.610213	0.0725847	0.0427738	0.669482	0.0638908	0.068238	uncharacterized protein
XP_009688888.1	0.0209458	0.508824	0.0411652	0.0144522	0.334127	0.0432535	0.042209	conserved hypothetical protein
XP_009688889.1	0.068367	0.42069	0.162512	0.0480705	0.328275	0.146434	0.154473	NifU-like protein 2%2C chloroplast precursor
XP_009688890.1	0.0314178	0.231512	0.135707				0.135707	conserved hypothetical protein
XP_009688891.1	0.0712804	0.376426	0.189361	0.0506788	0.396883	0.127692	0.158527	conserved hypothetical protein
XP_009688892.1	0.0288397	0.879943	0.0327745	0.0174233	0.880304	0.0197923	0.026283	protein phosphatase 2c
XP_009688893.1	0.0695883	1.47706	0.0471128	0.0605767	1.72847	0.0350464	0.04108	conserved hypothetical protein
XP_009688894.1	0.0230148	0.26645	0.0863757				0.0863757	50S ribosomal protein L33

protein_id	Chitose_ka	Chitose_ks	Chitose_kaks	Buffeli_ka	Buffeli_ks	Buffeli_kaks	mean_kaks	function
XP_009688895.1	0.0126359	0.354604	0.035634	NA	NA	NA	NA	acyl-CoA-binding protein
XP_009688896.1	0.196793	0.392211	0.501754	0.204284	0.358095	0.570475	0.536115	conserved hypothetical protein
XP_009688898.1	0.0472803	0.479451	0.0986136	0.0550233	0.476125	0.115565	0.107089	uncharacterized protein
XP_009688899.1	0.0290378	0.494859	0.058679	3.18349E-05	0.0318349	0.001	0.02984	conserved hypothetical protein
XP_009688900.1	0.0956622	0.634886	0.150676	0.137649	0.801586	0.171721	0.161199	importin subunit beta-1
XP_009688901.1	0.114928	0.407837	0.281799	0.142311	0.433158	0.328543	0.305171	orotate phosphoribosyltransferase
XP_009688902.1	0.0851099	0.379505	0.224265	0.0735462	0.342894	0.214486	0.219376	conserved hypothetical protein
XP_009688903.1	0.0113234	0.316106	0.0358215	0.00433321	0.296594	0.0146099	0.025216	uncharacterized protein
XP_009688904.1	0.0533125	0.537823	0.0991266	0.0549394	0.528106	0.104031	0.101579	methyltransferase
XP_009688905.1	0.0742231	0.523154	0.141876	0.0638463	0.53151	0.120123	0.130999	conserved hypothetical protein
XP_009688906.1	0.0832151	0.696526	0.119472	0.0817298	0.687032	0.118961	0.119217	uncharacterized protein
XP_009688908.1	0.0151971	0.422149	0.0359995				0.0359995	conserved hypothetical protein
XP_009688909.1	0.0214164	0.314941	0.0680015	0.0296288	0.417116	0.0710324	0.069517	conserved hypothetical protein
XP_009688910.1	0.0827771	0.301892	0.274194	0.106805	0.270274	0.395172	0.334683	conserved hypothetical protein
XP_009688911.1	0.0435849	0.692865	0.0629054	0.0389611	0.677491	0.0575079	0.060207	protein phosphatase 2A regulatory subunit B
XP_009688912.1	0.0266546	0.417184	0.0638917	0.0305279	0.56966	0.0535898	0.058741	vesicle transport protein
XP_009688913.1	0.0105457	0.31339	0.0336505	0.0121481	0.383526	0.0316748	0.032663	CUG-BP- and ETR-3-like factor 1
XP_009688914.1	0.0214717	0.644547	0.0333128	0.0199651	0.586797	0.0340239	0.033668	RNA-processing protein
XP_009688915.1	0.0362578	0.59384	0.0610565	0.0319886	0.565353	0.0565817	0.058819	conserved hypothetical protein
XP_009688916.1	0.0639763	0.446978	0.143131	0.0657793	0.445802	0.147553	0.145342	uncharacterized protein
XP_009688917.1	0.0458228	0.555627	0.0824704	0.0321421	0.563687	0.0570211	0.069746	uncharacterized protein
XP_009688918.1	0.0624131	0.348987	0.178841	0.0512211	0.419451	0.122115	0.150478	cytochrome C oxidase subunit 2a
XP_009688919.1	0.0438817	0.257442	0.170453	0.140463	0.413753	0.339485	0.254969	uncharacterized protein
XP_009688920.1	0.107194	0.40804	0.262705	0.133568	0.43227	0.308991	0.285848	conserved hypothetical protein
XP_009688921.1	0.0560547	0.352065	0.159217	0.0426713	0.338159	0.126187	0.142702	conserved hypothetical protein
XP_009688922.1	0.0106779	0.264633	0.0403499	0.00470766	0.127146	0.0370256	0.038688	conserved hypothetical protein
XP_009688923.1	0.0856073	0.605429	0.1414	0.0987574	0.638845	0.154587	0.147994	heat shock protein 70
XP_009688924.1	0.0213606	0.547652	0.0390039	0.0251303	0.424131	0.0592513	0.049128	protein transport protein sec24-like
XP_009688925.1	0.0961394	0.672414	0.142976	0.080878	0.657372	0.123032	0.133004	uncharacterized protein
XP_009688926.1	0.0277664	0.419274	0.066225	0.0500091	0.634196	0.0788543	0.07254	fructose-bisphosphate aldolase 2
XP_009688927.1	0.0231769	0.179149	0.129372	0.00836062	0.254813	0.0328108	0.081091	uncharacterized protein
XP_009688928.1	0.0420025	0.679247	0.0618368	0.0306744	0.599962	0.0511272	0.056482	uncharacterized protein
XP_009688929.1	0.0440342	0.348093	0.126502	0.0431246	0.122145	0.353062	0.239782	cytochrome C oxidase assembly protein-like
XP_009688930.1	0.0189746	0.476534	0.0398179				0.0398179	conserved hypothetical protein
XP_009688931.1	0.114954	0.352092	0.326488	0.0401173	0.11882	0.337632	0.33206	conserved hypothetical protein
XP_009688932.1	0.0309905	0.522326	0.0593317	0.0450225	0.666172	0.0675839	0.063458	uncharacterized protein
XP_009688933.1	0.0290366	0.273753	0.106068				0.106068	uncharacterized protein
XP_009688934.1	0.00617033	0.316731	0.0194813	0.000343409	0.343409	0.001	0.010241	conserved hypothetical protein
XP_009688935.1	0.0483023	0.253006	0.190914				0.190914	conserved hypothetical protein
XP_009688936.1	0.0552948	0.880304	0.0628133	0.0486127	0.972722	0.0499759	0.056395	uncharacterized protein
XP_009688937.1	0.0676085	0.471815	0.143295	0.0607869	0.331095	0.183593	0.163444	uncharacterized protein
XP_009688938.1	0.11332	0.596553	0.189958	0.102628	0.805166	0.127461	0.15871	conserved hypothetical protein
XP_009688939.1	0.0345633	0.310853	0.111189	0.0298282	0.258303	0.115478	0.113334	S-adenosyl-methyltransferase
XP_009688940.1	0.0380194	0.695314	0.0546795	0.0344703	0.444544	0.0775407	0.06611	peptidylprolyl isomerase
XP_009688941.1	0.0658719	0.449693	0.146482	0.0449977	0.371292	0.121192	0.133837	uncharacterized protein
XP_009688942.1	0.0745967	0.517619	0.144115	0.0628827	0.46732	0.13456	0.139338	predicted protein
XP_009688943.1	0.0840344	0.240139	0.34994	0.10032	0.252895	0.396686	0.373313	glycosyl transferase%2C family 4 protein
XP_009688944.1	0.0335752	0.565967	0.0593236	0.042425	0.535699	0.0791957	0.06926	long-chain-fatty-acid--CoA ligase
XP_009688945.1	0.0548353	0.464948	0.117939	0.0590861	0.476567	0.123983	0.120961	uncharacterized protein
XP_009688946.1	0.049029	0.426594	0.114931	0.0347631	0.317268	0.10957	0.112251	conserved hypothetical protein
XP_009688947.1	0.0487605	0.345253	0.141231	0.0270402	0.29699	0.0910476	0.116139	tRNA wybutosine-synthesizing protein 1 homolog
XP_009688948.1	0.0258218	0.463856	0.0556676	0.0267864	0.430963	0.0621549	0.058911	DNA-directed RNA polymerase subunit alpha
XP_009688949.1	0.0573921	0.802319	0.0715327	0.0574469	0.847815	0.0677587	0.069646	ubiquitin-activating enzyme E1

protein_id	Chitose_ka	Chitose_ks	Chitose_kaks	Buffeli_ka	Buffeli_ks	Buffeli_kaks	mean_kaks	function
XP_009688950.1	0.051287	0.797159	0.0643372	0.0422283	0.809149	0.0521886	0.058263	uncharacterized protein
XP_009688951.1	0.0276319	0.415314	0.0665324	0.0308714	0.544373	0.0567101	0.061621	protein disulfide isomerase precursor
XP_009688952.1	0.0493287	0.611907	0.0806147	0.0486554	0.645435	0.0753839	0.077999	conserved hypothetical protein
XP_009688953.1	0.0391247	0.494609	0.0791022	0.0395147	0.50041	0.0789646	0.079033	conserved hypothetical protein
XP_009688954.1	0.0603731	0.423295	0.142627	0.0551882	0.328505	0.167998	0.155313	conserved hypothetical protein
XP_009688955.1	0.0237153	0.379024	0.0625693	0.0360968	0.427926	0.084353	0.073461	conserved hypothetical protein
XP_009688956.1	0.0292279	0.43929	0.0665343	0.0267236	0.535825	0.0498738	0.058204	GTPase-activating protein
XP_009688957.1	0.0305079	0.629351	0.0484751	0.0256677	0.532204	0.048229	0.048352	conserved hypothetical protein
XP_009688958.1	0.00996587	0.30027	0.0331897	0.000309369	0.309369	0.001	0.017095	uncharacterized protein
XP_009688959.1	0.0774	0.410266	0.188658	0.0536789	0.442047	0.121433	0.155046	uncharacterized protein
XP_009688960.1	0.0816765	0.674609	0.121072	0.0867836	0.646321	0.134273	0.127672	uncharacterized protein
XP_009688961.1	0.0279632	0.412924	0.0677198	0.0228616	0.361589	0.0632252	0.065472	chromatin assembly factor 1 protein
XP_009688962.1	0.104608	0.381706	0.274053	0.107268	0.349168	0.30721	0.290631	conserved hypothetical protein
XP_009688963.1	0.0675509	0.361537	0.186844	0.0829344	0.429952	0.192892	0.189868	conserved hypothetical protein
XP_009688964.1	0.0489806	0.38807	0.126216	0.0500802	0.436502	0.114731	0.120473	histidyl-tRNA synthetase
XP_009688965.1	0.0408446	0.445999	0.0915801	0.0252951	0.410495	0.061621	0.076601	ATP-dependent RNA helicase
XP_009688966.1	0.0600244	0.372163	0.161285	0.066877	0.319699	0.209188	0.185237	conserved hypothetical protein
XP_009688967.1	0.044053	0.491647	0.0896029	0.0320252	0.400285	0.080006	0.084804	serine-threonine protein kinase
XP_009688968.1	0.0574619	0.385029	0.14924	0.0314873	0.260244	0.120992	0.135116	conserved hypothetical protein
XP_009688969.1	0.113108	0.438972	0.257665	0.103426	0.558164	0.185296	0.22148	conserved hypothetical protein
XP_009688970.1	0.11248	0.308252	0.364895				0.364895	conserved hypothetical protein
XP_009688971.1	0.0208119	0.431661	0.0482136	0.00914296	0.311212	0.0293786	0.038796	mitochondrial processing peptidase
XP_009688972.1	0.130331	0.491347	0.265253	0.143314	0.625564	0.229096	0.247175	uncharacterized protein
XP_009688973.1	0.059714	0.476925	0.125206	0.0319131	0.343289	0.0929628	0.109084	uncharacterized protein
XP_009688974.1	0.0374996	0.497501	0.075376	0.0407013	0.50161	0.0811413	0.078259	conserved hypothetical protein
XP_009688975.1				0.128422	0.428047	0.300019	0.300019	hypothetical protein
XP_009688976.1	0.0185598	0.514068	0.0361038	0.0123851	0.391674	0.0316209	0.033862	DNA excision-repair helicase
XP_009688977.1	0.0380485	0.354775	0.107247	0.00917775	0.174107	0.0527134	0.07998	ubiquitin regulatory protein
XP_009688978.1	0.146661	0.608053	0.241198	0.122713	0.651022	0.188493	0.214845	membrance occupation and recognition nexus protein 1
XP_009688979.1	0.0110478	0.381972	0.028923	0.0141838	0.44249	0.0320545	0.030489	membrance occupation and recognition nexus protein 1
XP_009688980.1	0.11689	0.294964	0.396285	0.157538	0.220898	0.713171	0.554728	conserved hypothetical protein
XP_009688981.1	0.0707088	0.434235	0.162835	0.0741712	0.3317	0.223609	0.193222	uncharacterized protein
XP_009688982.1	0.0595279	0.491201	0.121188	0.0483936	0.384936	0.125719	0.123453	seryl-tRNA synthetase
XP_009688983.1	0.0528808	0.436506	0.121146	0.037975	0.204105	0.186056	0.153601	signal recognition particle
XP_009688984.1	0.0447116	0.383244	0.116666	0.0605293	0.343101	0.176418	0.146542	uncharacterized protein
XP_009688985.1	0.0788188	0.441223	0.178637	0.069077	0.347731	0.198651	0.188644	conserved hypothetical protein
XP_009688986.1	0.0247724	0.587002	0.0422016	0.0304125	0.38263	0.0794828	0.060842	prolyl-tRNA synthetase
XP_009688987.1	0.0620367	0.409288	0.151572	0.0633311	0.255671	0.247705	0.199638	uncharacterized protein
XP_009688988.1	0.018982	0.560174	0.0338859	0.00744189	0.228182	0.0326138	0.03325	conserved hypothetical protein
XP_009688989.1	0.0723969	0.577838	0.125289	0.0719225	0.586131	0.122707	0.123998	conserved hypothetical protein
XP_009688990.1	0.124159	0.00248318	50	0.154	0.00308	50	50	hypothetical protein
XP_009688992.1	0.0638266	0.321647	0.198437	0.0680067	0.298512	0.227819	0.213128	uncharacterized protein
XP_009688993.1	0.0449818	0.30642	0.146798	0.0376889	0.254377	0.148162	0.14748	conserved hypothetical protein
XP_009688994.1	0.00357121	0.368321	0.00969592	0.0132619	0.296387	0.0447451	0.027221	adrenodoxin-like ferredoxin
XP_009688995.1	0.0219719	0.301745	0.0728161	6.00347E-05	0.0600347	0.001	0.036908	conserved hypothetical protein
XP_009688996.1	0.00420804	0.168432	0.0249836	0.000139467	0.139467	0.001	0.012992	conserved hypothetical protein
XP_009688997.1	0.0323796	0.507955	0.063745	0.0326364	0.523268	0.0623704	0.063058	2-cys peroxiredoxin
XP_009688998.1	0.219296	0.139851	1.56806	0.200353	0.12366	1.6202	1.59413	hypothetical protein
XP_009688999.1	0.0849472	0.762677	0.11138	0.106636	0.713287	0.149499	0.130439	conserved hypothetical protein
XP_009689000.1	0.0548396	0.876789	0.062546	0.040395	0.876516	0.0460858	0.054316	30S ribosomal protein S11
XP_009689001.1	0.0648269	0.38103	0.170136	0.0712043	0.289623	0.245851	0.207993	uncharacterized protein
XP_009689002.1	0.0101846	0.28892	0.0352507	0.00853342	0.355472	0.0240059	0.029628	uncharacterized protein
XP_009689003.1	0.0671358	0.686754	0.0977582	0.0682823	0.597645	0.114252	0.106005	uncharacterized protein

protein_id	Chitose_ka	Chitose_ks	Chitose_kaks	Buffeli_ka	Buffeli_ks	Buffeli_kaks	mean_kaks	function
XP_009689004.1	0.0550057	0.719267	0.0764746	0.0468883	0.510854	0.0917842	0.084129	uncharacterized protein
XP_009689005.1	0.0220722	0.279083	0.0790883	0.0342702	0.174171	0.196761	0.137925	uncharacterized protein
XP_009689006.1	0.0294917	0.36288	0.0812714	0.0429909	0.431767	0.0995696	0.09042	conserved hypothetical protein
XP_009689007.1	0.0187961	0.568895	0.0330397	0.00802337	0.331762	0.0241841	0.028612	proteasome subunit beta type 2
XP_009689008.1	0.00673665	0.475096	0.0141795	0.00501167	0.563526	0.00889341	0.011536	prohibitin
XP_009689009.1	0.0319272	0.310512	0.102821	0.0210172	0.166172	0.126479	0.11465	conserved hypothetical protein
XP_009689010.1	0.000339298	0.339298	0.001	0.00435066	0.481547	0.00903474	0.005017	chaperonin
XP_009689011.1	0.0160802	0.608148	0.0264413	0.00829735	0.523727	0.0158429	0.021142	peptidyl-prolyl cis-trans isomerase
XP_009689012.1	0.0386387	0.31139	0.124085	0.0264906	0.341689	0.0775283	0.100807	NifU protein
XP_009689013.1	0.0516585	0.375154	0.137699	0.0459044	0.267012	0.171919	0.154809	6-phosphogluconate dehydrogenase
XP_009689014.1	0.0359098	0.509164	0.0705269	0.0330169	0.551456	0.0598722	0.0652	actin
XP_009689015.1	0.0279206	0.382662	0.0729642	0.0135906	0.406669	0.0334193	0.053192	conserved hypothetical protein
XP_009689016.1	0.128547	0.27786	0.462632	0.158278	0.288877	0.54791	0.505271	conserved hypothetical protein
XP_009689017.1	0.117258	0.210683	0.556564	0.115252	0.251556	0.458154	0.507359	hypothetical protein
XP_009689018.1	0.0544065	0.264987	0.205318	0.0385375	0.295742	0.130308	0.167813	uncharacterized protein
XP_009689019.1	0.031733	0.310957	0.102049				0.102049	conserved hypothetical protein
XP_009689020.1	0.0113404	0.528185	0.0214704	0.013797	0.513739	0.0268562	0.024163	60S ribosomal protein L13
XP_009689021.1	0.0031522	0.295909	0.0106526	0.00328933	0.366655	0.0089712	0.009812	40S ribosomal protein S16
XP_009689022.1	0.0195697	0.44966	0.0435212	0.0104559	0.421669	0.0247964	0.034159	ATP synthase subunit gamma%2C mitochondrial precursor
XP_009689023.1	0.0209922	0.373008	0.0562781	0.0090058	0.39487	0.022807	0.039543	uncharacterized protein
XP_009689024.1	0.0303943	0.472263	0.0643588	0.019382	0.385408	0.0502897	0.057324	conserved hypothetical protein
XP_009689025.1	0.057119	0.584862	0.0976624	0.0257882	0.391401	0.0658869	0.081775	conserved hypothetical protein
XP_009689026.1	0.0299857	0.439512	0.0682249	0.0328352	0.545773	0.0601627	0.064194	uncharacterized protein
XP_009689027.1	0.0571533	0.388972	0.146934	0.0510041	0.457016	0.111602	0.129268	conserved hypothetical protein
XP_009689028.1	0.0256721	1.24204	0.0206694	0.0304994	1.37909	0.0221155	0.021392	Bromodomain protein
XP_009689029.1	0.0132917	0.351812	0.0377807	0.0149379	0.592146	0.0252268	0.031504	succinate dehydrogenase
XP_009689030.1	0.0842304	0.453006	0.185937	0.0447583	0.344358	0.129976	0.157956	conserved hypothetical protein
XP_009689031.1	0.0681689	0.339384	0.200861	0.0695129	0.307498	0.22606	0.21346	autoaggregation-mediating protein
XP_009689032.1	0.0158267	0.340601	0.0464671	0.0172065	0.234576	0.0733518	0.059909	conserved hypothetical protein
XP_009689033.1	0.0441472	0.656416	0.0672549	0.0347204	0.638625	0.0543675	0.060811	uncharacterized protein
XP_009689034.1	0.0401883	0.642312	0.0625683	0.0355592	0.585498	0.0607333	0.061651	conserved hypothetical protein
XP_009689035.1	0.0218988	0.612271	0.0357665	0.00918988	0.454337	0.020227	0.027997	uncharacterized protein
XP_009689036.1	0.0781489	0.394212	0.198241	0.0589707	0.41114	0.143432	0.170837	uncharacterized protein
XP_009689037.1	0.0287691	0.296722	0.0969562	0.0311875	0.287695	0.108405	0.102681	glycerol-3-phosphate acyltransferase
XP_009689038.1	0.0377014	0.309145	0.121954	0.0420188	0.292614	0.143598	0.132776	conserved hypothetical protein
XP_009689039.1	0.118824	0.0642894	1.84827	0.0977158	0.0400812	2.43795	2.14311	conserved hypothetical protein
XP_009689040.1	0.0817609	0.413227	0.19786	0.0541641	0.415643	0.130314	0.164087	conserved hypothetical protein
XP_009689041.1	0.0566057	0.536918	0.105427	0.0499893	0.356662	0.140159	0.122793	uncharacterized protein
XP_009689042.1	0.0115048	0.285896	0.0402411	0.00298697	0.0892805	0.033456	0.036849	DNA-directed RNA polymerase II
XP_009689043.1				NA	NA	NA	NA	conserved hypothetical protein
XP_009689044.1	0.0114864	0.378716	0.0303298	0.000318431	0.318431	0.001	0.015665	centrin 3
XP_009689045.1	0.127934	0.234432	0.545719				0.545719	hypothetical protein
XP_009689046.1	0.0599355	0.834644	0.0718097	0.0628567	0.801091	0.0784638	0.075137	E3 ubiquitin-protein ligase HUWE1
XP_009689047.1	0.0759473	0.542643	0.139958	0.0905836	0.564351	0.160509	0.150234	conserved hypothetical protein
XP_009689048.1	0.0144819	0.44864	0.0322795	0.0163851	0.482468	0.033961	0.03312	uncharacterized protein
XP_009689049.1	0.024184	0.304391	0.0794505	0.0224381	0.30158	0.074402	0.076926	conserved hypothetical protein
XP_009689050.1	0.00325102	0.431051	0.00754206	0.000612727	0.612727	0.001	0.004271	DNA-directed RNA polymerases I%2C II%2C and III subunit
XP_009689051.1	0.0276257	0.78468	0.0352063	0.0448325	1.9301	0.023228	0.029217	dynein light chain 1
XP_009689052.1	0.0412902	0.789159	0.0523218	0.0391412	0.681141	0.0574641	0.054893	eukaryotic translation initiation factor 3 subunit 10
XP_009689053.1	0.0645111	0.406134	0.158842	0.0596927	0.442595	0.13487	0.146856	uncharacterized protein
XP_009689054.1	0.0345501	0.465475	0.0742255	0.0164314	0.352096	0.0466673	0.060446	pre-rRNA processing protein
XP_009689055.1	0.0466657	0.444493	0.104986	0.0520554	0.459398	0.113312	0.109149	RNA-binding motif protein%2C X-linked 2
XP_009689056.1	0.0516404	0.486669	0.10611	0.0448046	0.399161	0.112247	0.109178	conserved hypothetical protein

protein_id	Chitose_ka	Chitose_ks	Chitose_kaks	Buffeli_ka	Buffeli_ks	Buffeli_kaks	mean_kaks	function
XP_009689057.1	0.0229385	0.508709	0.0450917	0.0213314	0.53342	0.0399899	0.042541	uncharacterized protein
XP_009689058.1	0.00765157	0.518649	0.0147529	0.00805831	0.665729	0.0121045	0.013429	T-complex protein 1 delta subunit
XP_009689059.1	0.0538625	0.476695	0.112992	0.0470719	0.395048	0.119155	0.116073	uncharacterized protein
XP_009689060.1	0.100018	0.588734	0.169886	0.0897294	0.510443	0.175787	0.172837	uncharacterized protein
XP_009689061.1	0.0568277	0.714882	0.0794925	0.0652337	0.714791	0.0912626	0.085378	uncharacterized protein
XP_009689062.1	0.0976159	0.453024	0.215476	0.0980929	0.49346	0.198786	0.207131	conserved hypothetical protein
XP_009689063.1	0.092122	0.344162	0.26767				0.26767	conserved hypothetical protein
XP_009689064.1	0.0305781	0.319581	0.0956818	0.0222179	0.255796	0.0868579	0.09127	conserved hypothetical protein
XP_009689066.1	0.0544832	0.407618	0.133662	0.0441823	0.344987	0.12807	0.130866	ABC transporter
XP_009689067.1	0.0703973	0.456453	0.154227	0.0668402	0.372077	0.179641	0.166934	uncharacterized protein
XP_009689068.1	0.0415318	0.55608	0.0746868	0.0386788	0.398966	0.0969476	0.085817	uncharacterized protein
XP_009689069.1	0.0391878	0.316332	0.123882	0.0379648	0.273837	0.13864	0.131261	uncharacterized protein
XP_009689070.1	0.0210521	0.507473	0.0414843	0.0167922	0.443064	0.0379001	0.039692	acyl transferase
XP_009689071.1	0.0668961	0.537941	0.124356	0.0517966	0.554432	0.0934227	0.108889	uncharacterized protein
XP_009689072.1	0.0603462	0.678098	0.0889933	0.0581879	0.575441	0.101119	0.095056	mRNA capping enzyme subunit
XP_009689073.1	0.0282671	0.432894	0.065298	0.0312988	0.456706	0.0685317	0.066915	peptidyl-prolyl cis-trans isomerase
XP_009689074.1	0.0500136	0.497875	0.100454	0.0383113	0.42413	0.0903292	0.095392	conserved hypothetical protein
XP_009689075.1	0.0388739	0.463379	0.0838923	0.0353958	0.365946	0.096724	0.090308	dihydrolipoamide succinyltransferase component of 2-oxoglutarate dehydrogenase complex%2C mitochondrial precursor
XP_009689076.1	0.0477956	1.15394	0.0414194	0.0457079	1.17827	0.0387924	0.040106	uncharacterized protein
XP_009689077.1	0.0309828	0.423897	0.0730903	0.0294474	0.412973	0.0713057	0.072198	glutathione synthetase
XP_009689078.1	0.0265989	0.501335	0.0530561	0.0217049	0.345503	0.062821	0.057939	glutathione synthetase
XP_009689079.1	0.0883979	0.337662	0.261794	0.0780549	0.345787	0.225731	0.243762	conserved hypothetical protein
XP_009689080.1	0.0390243	0.491974	0.0793217	5.69974E-05	0.0569974	0.001	0.040161	prefoldin subunit
XP_009689081.1	0.0832355	0.407931	0.204043				0.204043	conserved hypothetical protein
XP_009689082.1	0.0350847	0.45502	0.0771059	0.0418461	0.35799	0.116892	0.096999	uncharacterized protein
XP_009689083.1	0.0472933	0.353597	0.133749				0.133749	conserved hypothetical protein
XP_009689084.1	0.0717051	0.449613	0.159482	0.0788511	0.507551	0.155356	0.157419	conserved hypothetical protein
XP_009689085.1	0.0422085	0.620505	0.0680229	0.0345915	0.666508	0.0518996	0.059961	uncharacterized protein
XP_009689086.1	0.0404419	0.692018	0.0584405	0.0408928	0.733555	0.0557461	0.057093	MORN motif repeat containing protein
XP_009689087.1	0.0784134	0.454365	0.172578	0.0746306	0.43679	0.170861	0.17172	conserved hypothetical protein
XP_009689088.1	0.0262976	0.25785	0.101988	0.0244385	0.265583	0.0920181	0.097003	translationally-controlled tumor protein homolog TCTP
XP_009689089.1	0.0505418	0.377478	0.133893	0.0484938	0.412779	0.117481	0.125687	conserved hypothetical protein
XP_009689090.1	0.0221273	0.478209	0.0462712	0.019065	0.495747	0.0384571	0.042364	translation elongation factor G
XP_009689091.1	0.00148537	0.470033	0.00316014	0.0015334	0.395221	0.00387985	0.00352	developmentally regulated GTP-binding protein 1
XP_009689092.1	0.0367078	0.357206	0.102764	0.0305262	0.35957	0.0848964	0.09383	uncharacterized protein
XP_009689093.1	0.045815	0.712761	0.0642783	0.0437174	0.629514	0.0694462	0.066862	uncharacterized protein
XP_009689094.1	0.127107	0.268356	0.473653	0.0656803	0.220016	0.298525	0.386089	hypothetical protein
XP_009689095.1	0.0492406	0.64061	0.0768651	0.0817193	1.1319	0.0721968	0.074531	importin beta
XP_009689096.1	0.123069	0.482178	0.255236	0.129079	0.580127	0.222501	0.238869	uncharacterized protein
XP_009689097.1	0.030562	0.549818	0.0555856	0.0202446	0.544831	0.0371576	0.046372	uncharacterized protein
XP_009689098.1	0.0751773	0.63757	0.117912	0.0641959	0.634628	0.101155	0.109534	structural maintenance of chromosome protein%2C type 1
XP_009689099.1	0.041496	0.455111	0.0911778	0.000302464	0.302464	0.001	0.046089	ubiquitin-fold modifier 1 precursor
XP_009689100.1	0.0508333	0.675664	0.0752346	0.0531882	0.738111	0.0720599	0.073647	conserved hypothetical protein
XP_009689101.1	0.000479651	0.479651	0.001	0.00163394	0.299745	0.00545111	0.003226	26S proteasome regulatory particle
XP_009689102.1	0.0493199	0.502357	0.098177	0.0447904	0.403475	0.111012	0.104595	conserved hypothetical protein
XP_009689103.1	0.0241574	0.323439	0.0746891	0.0361582	0.330665	0.10935	0.09202	hydroxyacylglutathione hydrolase
XP_009689104.1	0.0155815	0.345268	0.0451288				0.0451288	60S ribosomal protein L38
XP_009689105.1	0.0168643	0.682063	0.0247255	0.0222891	0.512573	0.0434848	0.034105	60S acidic ribosomal protein P0
XP_009689106.1	0.0441864	0.388337	0.113784	0.0486567	0.603792	0.0805852	0.097185	uncharacterized protein
XP_009689107.1	0.121821	0.514277	0.236878	0.0189218	0.429634	0.0440416	0.14046	conserved hypothetical protein
XP_009689108.1	0.0534359	0.515793	0.1036	0.0449792	0.434084	0.103619	0.103609	conserved hypothetical protein
XP_009689109.1	0.0641691	0.44126	0.145422	0.0602115	0.347761	0.17314	0.159281	uncharacterized protein
XP_009689110.1	0.00840888	0.139665	0.0602076				0.0602076	uncharacterized protein

protein_id	Chitose_ka	Chitose_ks	Chitose_kaks	Buffeli_ka	Buffeli_ks	Buffeli_kaks	mean_kaks	function
XP_009689111.1	0.0135228	0.499544	0.0270703	0.00992007	0.356893	0.0277957	0.027433	glycerol-3-phosphate dehydrogenase
XP_009689112.1	0.0661833	0.396856	0.166769	0.058529	0.314412	0.186154	0.176461	serine/threonine protein kinase
XP_009689113.1	0.0520455	0.539595	0.0964529	0.0499119	0.634351	0.0786818	0.087567	conserved hypothetical protein
XP_009689114.1	0.0111475	0.108095	0.103127				0.103127	conserved hypothetical protein
XP_009689115.1	0.098714	0.561727	0.175733	0.0935259	0.564301	0.165738	0.170735	conserved hypothetical protein
XP_009689116.1				0.0153211	0.167802	0.0913046	0.0913046	3'-5' exoribonuclease
XP_009689117.1	0.142195	0.469929	0.302588	0.115111	0.490825	0.234525	0.268557	conserved hypothetical protein
XP_009689118.1	0.0640521	0.355494	0.180178	0.0307543	0.438976	0.0700592	0.125119	molecular chaperone
XP_009689119.1	0.0965884	0.737547	0.130959	0.0871219	0.749514	0.116238	0.123599	uncharacterized protein
XP_009689120.1	0.0519304	0.287254	0.180782	0.0372497	0.172306	0.216184	0.198483	conserved hypothetical protein
XP_009689121.1	0.0131379	0.272113	0.0482812	0.00834237	0.190956	0.0436874	0.045984	eukaryotic translation initiation factor
XP_009689122.1	0.0761444	0.292082	0.260696				0.260696	conserved hypothetical protein
XP_009689123.1	0.0360304	0.393218	0.0916296				0.0916296	predicted protein
XP_009689124.1	0.0406338	0.410866	0.098898	0.0341662	0.546033	0.0625717	0.080735	conserved hypothetical protein
XP_009689125.1	0.0795035	0.211909	0.375177	0.0508349	0.336533	0.151055	0.263116	hypothetical protein
XP_009689126.1	0.0306963	0.30839	0.0995371	0.0266201	0.297449	0.0894947	0.094516	serine protease
XP_009689127.1	0.0844868	0.400041	0.211195	0.040155	0.106786	0.376033	0.293614	potential glutaredoxin
XP_009689128.1	0.0982273	0.616741	0.159268	0.0935125	0.675986	0.138335	0.148802	conserved hypothetical protein
XP_009689129.1	0.0186621	0.513202	0.036364	0.0124874	0.123938	0.100755	0.068559	uncharacterized protein
XP_009689130.1	0.0188975	0.417816	0.0452292	0.0248778	0.564102	0.0441017	0.044665	tubulin subunit alpha
XP_009689131.1	0.0506661	0.872166	0.0580923	0.05489	0.986628	0.0556339	0.056863	conserved hypothetical protein
XP_009689132.1	0.0285963	0.559522	0.0511084	0.0480449	0.635662	0.0755825	0.063345	conserved hypothetical protein
XP_009689133.1	0.080528	0.896438	0.0898311	0.0779422	0.833346	0.0935292	0.09168	predicted protein
XP_009689134.1	0.0504886	0.16819	0.300187				0.300187	uncharacterized protein
XP_009689135.1	0.0123109	0.279643	0.0440236				0.0440236	conserved hypothetical protein
XP_009689136.1	0.0261196	0.263046	0.0992968	0.031988	0.568429	0.0562743	0.077786	uncharacterized protein
XP_009689137.1	0.0320646	0.30606	0.104766				0.104766	Bet3 transport protein
XP_009689139.1	0.186694	0.354418	0.526763	0.157906	0.458454	0.344432	0.435597	conserved hypothetical protein
XP_009689140.1	0.00319062	0.410408	0.00777425	0.00695508	0.543638	0.0127936	0.010284	eukaryotic translation initiation factor 2 subunit gamma
XP_009689141.1	0.0363228	0.534054	0.0680133	0.0525459	0.564098	0.0931503	0.080582	uncharacterized protein
XP_009689142.1	0.0335792	0.401427	0.0836497	0.0106705	0.178271	0.0598555	0.071753	Conserved hypothetical protein
XP_009689144.1	0.0505995	0.361364	0.140023	0.0250368	0.0735967	0.34019	0.240107	uncharacterized protein
XP_009689145.1	0.131276	0.516283	0.254272	0.102507	0.454375	0.2256	0.239936	conserved hypothetical protein
XP_009689146.1	0.127969	0.656484	0.194931	0.126262	0.579213	0.217988	0.206459	hypothetical protein
XP_009689148.1	0.0398715	0.411386	0.0969198	0.0323006	0.415383	0.0777611	0.08734	uncharacterized protein
XP_009689149.1	0.126137	0.429681	0.293559	0.122372	0.450694	0.271518	0.282539	conserved hypothetical protein
XP_009689150.1	0.0402982	0.525747	0.0766495	0.044407	0.371743	0.119456	0.098053	uncharacterized protein
XP_009689151.1	0.0390862	0.896672	0.0435903	0.0418121	0.934908	0.0447233	0.044157	uncharacterized protein
XP_009689152.1	0.0393821	0.710004	0.0554675	0.0419925	0.7916	0.0530477	0.054258	uncharacterized protein
XP_009689153.1	0.0534664	0.521561	0.102512	0.0371825	0.668269	0.0556401	0.079076	conserved hypothetical protein
XP_009689154.1	0.0828086	0.195359	0.423879	0.0781774	0.2324	0.336391	0.380135	uncharacterized protein
XP_009689155.1	0.052168	0.432215	0.120699	0.0320814	0.386973	0.0829035	0.101801	conserved hypothetical protein
XP_009689156.1	0.00305171	0.306948	0.00994209	0.0555451	0.296624	0.187258	0.0986	ribosomal protein S18
XP_009689157.1	0.000371489	0.371489	0.001	0.000309385	0.309385	0.001	0.001	60S ribosomal protein L11a
XP_009689158.1	0.0328665	0.35449	0.0927149	0.0191732	0.467492	0.0410129	0.066864	conserved hypothetical protein
XP_009689159.1	0.0819916	0.401267	0.204332	0.0520441	0.28741	0.18108	0.192706	uncharacterized protein
XP_009689160.1	0.033333	0.763	0.0436868	0.0440131	0.721311	0.0610181	0.052352	Map2 kinase
XP_009689161.1	0.00364162	0.208814	0.0174395	0.00784914	0.200229	0.0392007	0.02832	uncharacterized protein
XP_009689162.1	0.0432364	0.39168	0.110387	0.0352117	0.184733	0.190609	0.150498	conserved hypothetical protein
XP_009689163.1	0.0529988	0.740836	0.0715392	0.0568256	0.765103	0.0742718	0.072905	Gmp reductase
XP_009689164.1	0.0308788	0.348306	0.0886543	0.0366893	0.46104	0.0795794	0.084117	proliferation-associated protein 2g4
XP_009689165.1	0.0742676	0.494165	0.150289	0.076575	0.63468	0.120651	0.13547	conserved hypothetical protein
XP_009689166.1	0.084028	0.393648	0.21346	0.0486707	0.312237	0.155878	0.184669	uncharacterized protein

protein_id	Chitose_ka	Chitose_ks	Chitose_kaks	Buffeli_ka	Buffeli_ks	Buffeli_kaks	mean_kaks	function
XP_009689167.1	0.0223568	0.269488	0.08296				0.08296	ribosomal protein L13
XP_009689168.1	0.0283324	0.556673	0.050896	0.0274106	0.675886	0.040555	0.045726	conserved hypothetical protein
XP_009689169.1	0.0301454	0.771783	0.0390594	0.0380473	0.74248	0.0512435	0.045151	protein transport protein sec24-like
XP_009689170.1	0.0610878	0.339784	0.179784	0.0598278	0.362806	0.164903	0.172343	ATP-dependent RNA helicase
XP_009689171.1	0.0169602	0.38737	0.0437831	0.0169288	0.351468	0.048166	0.045975	conserved hypothetical protein
XP_009689172.1	0.0062525	0.469182	0.0133264	0.000307766	0.307766	0.001	0.007163	small nuclear ribonucleoprotein f
XP_009689173.1	0.0527481	0.645777	0.0816816	0.0331262	0.729438	0.0454133	0.063547	tRNA (guanine-n-(7))-methyltransferase
XP_009689174.1	0.140356	0.53907	0.260368				0.260368	conserved hypothetical protein
XP_009689175.1	0.155262	0.45093	0.344316	0.0989681	0.268165	0.369056	0.356686	conserved hypothetical protein
XP_009689176.1	0.0441758	0.971276	0.0454822	0.0244085	0.790224	0.030888	0.038185	deoxyuridine 5'-triphosphate nucleotidohydrolase
XP_009689177.1	0.0690926	0.592995	0.116515	0.0619142	0.58559	0.10573	0.111122	conserved hypothetical protein
XP_009689178.1	0.0254047	0.551144	0.0460945	0.0246623	0.433482	0.0568934	0.051494	uncharacterized protein
XP_009689179.1	0.132696	0.313171	0.423719	0.157907	0.397859	0.396891	0.410305	conserved hypothetical protein
XP_009689180.1	0.0341786	0.692132	0.0493816	0.0351396	0.563275	0.0623844	0.055883	DNA polymerase I
XP_009689181.1	0.039016	0.211702	0.184297				0.184297	uncharacterized protein
XP_009689182.1	0.0368225	0.702022	0.052452	0.0174704	0.618718	0.0282365	0.040344	ATP synthase subunit beta
XP_009689183.1	0.0648736	0.88162	0.0735846	0.0480761	0.956128	0.050282	0.061933	serine hydroxymethyltransferase
XP_009689184.1	0.0794626	0.729265	0.108963	0.101244	0.536857	0.188586	0.148775	uncharacterized protein
XP_009689185.1	0.113636	0.489222	0.232279	0.0970291	0.455307	0.213107	0.222693	conserved hypothetical protein
XP_009689186.1	0.0178204	0.533775	0.0333856	0.0617894	0.513009	0.120445	0.076915	uncharacterized protein
XP_009689187.1	0.0373581	0.526696	0.0709291	0.0314978	0.486621	0.0647276	0.067828	coatomer complex subunit beta
XP_009689188.1	0.0262334	0.0339466	0.772784	0.0498009	0.48867	0.101911	0.437348	uncharacterized protein
XP_009689189.1	0.0683238	0.734367	0.0930377	0.0653473	0.676403	0.09661	0.094824	uncharacterized protein
XP_009689190.1	0.0235719	1.10877	0.0212594	0.0217898	1.09138	0.0199654	0.020612	aminopeptidase N
XP_009689191.1	0.0510096	2.8788	0.017719	0.00580984	4.29114	0.00135392	0.009536	hypothetical protein
XP_009689192.1	0.02904	0.61859	0.0469454	0.026699	0.65011	0.0410683	0.044007	exosome complex exonuclease rrp41
XP_009689193.1	0.00583123	0.348641	0.0167256	0.00204584	0.32523	0.00629044	0.011508	uncharacterized protein
XP_009689194.1	0.0558651	0.519715	0.107492	0.0388858	0.424038	0.0917035	0.099598	conserved hypothetical protein
XP_009689195.1	0.0547164	0.527951	0.103639	0.048345	0.481428	0.10042	0.102029	conserved hypothetical protein
XP_009689197.1	0.0644056	0.456163	0.14119	0.0642501	0.378135	0.169913	0.155552	transporter
XP_009689198.1	0.135478	0.537439	0.252081	0.108247	0.660221	0.163956	0.208018	conserved hypothetical protein
XP_009689199.1	0.0695216	0.449921	0.15452	0.0504568	0.390671	0.129154	0.141837	uncharacterized protein
XP_009689200.1	0.118589	0.059117	2.00601				2.00601	hypothetical protein
XP_009689201.1	0.126924	0.752967	0.168565	0.105107	0.660937	0.159028	0.163797	HEAT repeat containing protein
XP_009689202.1	0.119623	0.778248	0.153708				0.153708	uncharacterized protein
XP_009689203.1	0.0268216	0.748941	0.0358126	0.0272004	0.813915	0.0334192	0.034616	conserved hypothetical protein
XP_009689204.1	0.00746251	0.220593	0.0338293	0.00385304	0.203615	0.0189232	0.026376	conserved hypothetical protein
XP_009689205.1	0.0226875	0.344025	0.0659472				0.0659472	conserved hypothetical protein
XP_009689206.1	0.0142982	0.703678	0.0203193	0.00412993	1.67805	0.00246115	0.01139	ribosomal protein l6
XP_009689207.1	0.0140813	0.295123	0.0477135	0.0149628	0.332639	0.0449822	0.046348	ribosomal protein S15%2C mitochondrial precursor
XP_009689208.1	0.0512951	0.604939	0.0847939	0.0442688	0.55754	0.0794002	0.082097	uncharacterized protein
XP_009689209.1	0.0382217	0.954713	0.0400347	0.0702516	0.467739	0.150194	0.095114	uncharacterized protein
XP_009689210.1	0.0720565	0.698406	0.103173	0.0652512	0.57707	0.113073	0.108123	conserved hypothetical protein
XP_009689211.1	0.101545	0.505729	0.200791	0.122845	0.474304	0.259	0.229896	uncharacterized protein
XP_009689212.1	0.130941	0.394381	0.332018	0.111647	0.387048	0.288458	0.310238	conserved hypothetical protein
XP_009689213.1	0.044803	0.897216	0.0499356	0.0346214	0.789729	0.0438395	0.046888	serine-threonine protein kinase
XP_009689214.1	0.041357	0.43046	0.0960763	0.0438728	0.391746	0.111993	0.104035	peptide chain release factor 1%2C mitochondrial precursor
XP_009689215.1	0.0635962	0.385555	0.164947	0.0585714	0.407568	0.143709	0.154328	uncharacterized protein
XP_009689216.1	0.00638339	0.45689	0.0139714	0.00744574	0.65475	0.0113719	0.012672	uncharacterized protein
XP_009689217.1	0.0386647	0.668955	0.0577987	0.0305962	0.602021	0.0508224	0.054311	conserved hypothetical protein
XP_009689218.1	0.0291704	0.465501	0.0626644	0.0381947	0.49544	0.0770925	0.069878	conserved hypothetical protein
XP_009689219.1	0.069077	0.98076	0.0704322	0.0681405	0.950871	0.0716611	0.071047	conserved hypothetical protein
XP_009689220.1	0.122141	0.410652	0.297432	0.12196	0.428645	0.284523	0.290978	conserved hypothetical protein

protein_id	Chitose_ka	Chitose_ks	Chitose_kaks	Buffeli_ka	Buffeli_ks	Buffeli_kaks	mean_kaks	function
XP_009689221.1	0.0549276	0.976089	0.0562731	0.0419776	0.937851	0.0447594	0.050516	conserved hypothetical protein
XP_009689222.1	0.0611892	0.668349	0.0915528	0.0707611	0.790163	0.0895525	0.090553	conserved hypothetical protein
XP_009689223.1	0.023753	0.33468	0.0709722	0.0146495	0.261679	0.0559826	0.063477	leucine-rich repeat%2C typical subtype containing protein
XP_009689224.1	0.0847295	0.526294	0.160993	0.082971	0.462022	0.179583	0.170288	predicted protein
XP_009689225.1	0.0549839	0.391562	0.140422	0.0507712	0.310511	0.163509	0.151965	conserved hypothetical protein
XP_009689226.1	0.0661257	0.46604	0.141889	0.0753846	0.534863	0.140942	0.141415	dihydrofolate synthase/folylpolyglutamate synthase
XP_009689227.1	0.0801077	0.738983	0.108403	0.0787368	0.634048	0.124181	0.116292	HEAT repeat containing protein
XP_009689228.1	0.0210998	0.371106	0.0568566	NA	NA	NA	NA	uncharacterized protein
XP_009689229.1	0.126118	0.417124	0.302352				0.302352	Conserved hypothetical protein
XP_009689230.1	0.045508	0.336573	0.13521	0.0468493	0.275579	0.170003	0.152606	tRNA delta (2)-isopentenylpyrophosphate transferase
XP_009689231.1	0.0409117	0.588466	0.0695226	0.0492241	0.394779	0.124688	0.097105	uncharacterized protein
XP_009689232.1	0.0862758	0.566575	0.152276	0.0833916	0.452508	0.184287	0.168282	conserved hypothetical protein
XP_009689233.1	0.0555188	0.00111038	50	0.0363371	0.000726814	49.995	49.9975	conserved hypothetical protein
XP_009689237.1	0.0436099	0.719446	0.060616	0.0268842	0.613528	0.043819	0.052218	DNA mismatch repair protein Msh2
XP_009689238.1	0.052746	0.742169	0.0710701	0.0500119	0.718287	0.0696266	0.070348	conserved hypothetical protein
XP_009689239.1	0.0363345	0.262143	0.138606	0.0561225	0.375874	0.149312	0.143959	uncharacterized protein
XP_009689240.1	0.0308713	0.692518	0.0445783	0.0227774	0.841279	0.0270748	0.035827	glycyl-tRNA synthetase
XP_009689241.1	0.0413312	0.433437	0.0953568	0.0350649	0.434879	0.0806315	0.087994	cytokine induced apoptosis inhibitor 1
XP_009689242.1	0.016548	0.692599	0.0238926	0.014135	0.705518	0.020035	0.021964	histone acetyltransferase gcn5-related
XP_009689243.1	0.203638	0.0725212	2.80798	0.322144	0.163585	1.96928	2.38863	hypothetical protein
XP_009689244.1	0.0784259	0.985159	0.0796073	0.0649961	0.955871	0.0679967	0.073802	uncharacterized protein
XP_009689245.1	0.0847994	0.514927	0.164682	0.0679596	0.510996	0.132994	0.148838	conserved hypothetical protein
XP_009689246.1	0.0312704	0.487542	0.0641388	0.0261289	0.425043	0.0614735	0.062806	conserved hypothetical protein
XP_009689247.1	0.0112352	0.283301	0.0396582				0.0396582	conserved hypothetical protein
XP_009689248.1	0.0560057	0.515033	0.108742	0.0473768	0.474647	0.0998148	0.104278	integral membrane protein
XP_009689249.1	0.0639701	0.305682	0.20927	0.0548547	0.266792	0.205609	0.207439	uncharacterized protein
XP_009689250.1	0.0376831	0.58219	0.0647265	0.0349242	0.508091	0.068736	0.066731	conserved hypothetical protein
XP_009689251.1	0.0735621	0.477437	0.154077	0.0543728	0.462641	0.117527	0.135802	conserved hypothetical protein
XP_009689252.1	0.0159814	0.356007	0.0448908				0.0448908	conserved thioredoxin-like protein
XP_009689253.1	0.0246127	0.528066	0.0466092	0.0213761	0.682372	0.0313262	0.038968	aspartate transcarbamoylase
XP_009689254.1	0.00854971	0.000172316	49.6164				49.6164	hypothetical protein
XP_009689255.1	0.123382	0.107988	1.14255	0.14377	0.108326	1.32721	1.23488	hypothetical protein
XP_009689256.1	0.050788	0.98022	0.0518128	0.0552134	0.647851	0.0852255	0.068519	uncharacterized protein
XP_009689257.1	0.036686	0.589645	0.0622171	0.0464163	0.537949	0.0862838	0.07425	HSP70 chaperone
XP_009689258.1	0.0973442	0.614607	0.158385	0.123523	0.73084	0.169015	0.1637	conserved hypothetical protein
XP_009689259.1	0.110298	0.451665	0.244202	0.0996211	0.445013	0.223861	0.234032	uncharacterized protein
XP_009689260.1	0.0864693	0.92109	0.0938771	0.0689561	0.746894	0.0923238	0.0931	conserved hypothetical protein
XP_009689261.1	0.0242496	0.933401	0.0259798	0.0191568	0.877762	0.0218246	0.023902	U4/U6 small nuclear ribonucleoprotein
XP_009689262.1	0.0615283	0.813839	0.0756026	0.0531283	0.676812	0.0784979	0.07705	uncharacterized protein
XP_009689263.1	0.0108324	0.462393	0.0234268	0.00717339	0.588477	0.0121897	0.017808	uncharacterized protein
XP_009689264.1	0.0519628	0.507343	0.102422	0.0536885	0.4936	0.108769	0.105596	UDP-N-acetylglucosamine pyrophosphorylase
XP_009689265.1	0.041948	0.504137	0.0832075	0.0428401	0.525604	0.0815065	0.082357	conserved hypothetical protein
XP_009689266.1	0.0666429	0.841163	0.0792271	0.0657893	0.828153	0.079441	0.079334	conserved hypothetical protein
XP_009689267.1	0.0197178	0.639014	0.0308566	0.0274969	0.587454	0.0468069	0.038832	phosphoenolpyruvate carboxykinase
XP_009689268.1	0.0485863	0.58618	0.0828864	0.0473038	0.626342	0.0755238	0.079205	exosome complex exonuclease rrp44
XP_009689269.1	0.0412697	0.993956	0.0415206	0.0616345	0.603911	0.102059	0.07179	ribonucleoprotein
XP_009689270.1	0.0419638	0.589926	0.071134	0.0253354	0.512014	0.0494818	0.060308	uncharacterized protein
XP_009689271.1	0.101278	0.621082	0.163068	0.129372	0.694843	0.186189	0.174628	conserved hypothetical protein
XP_009689272.1	0.0333602	0.422884	0.0788873				0.0788873	conserved hypothetical protein
XP_009689274.1	0.0896511	0.419559	0.21368	0.0293857	0.35411	0.0829846	0.148332	conserved hypothetical protein
XP_009689276.1	0.0252904	0.769958	0.0328465	0.0193343	0.93499	0.0206786	0.026763	myosin a
XP_009689278.1				0.0891823	0.40558	0.219888	0.219888	conserved hypothetical protein
XP_009689279.1	0.127471	0.259632	0.49097	0.111018	0.243952	0.455082	0.473026	conserved hypothetical protein

protein_id	Chitose_ka	Chitose_ks	Chitose_kaks	Buffeli_ka	Buffeli_ks	Buffeli_kaks	mean_kaks	function
XP_009689280.1	0.102569	0.359183	0.285563	0.119787	0.313267	0.38238	0.333971	conserved hypothetical protein
XP_009689282.1	0.0644058	0.490996	0.131174				0.131174	uncharacterized protein
XP_009689283.1	0.000497503	0.497503	0.001	0.000460579	0.460579	0.001	0.001	ribosomal protein S15
XP_009689284.1	0.0668005	0.456379	0.146371				0.146371	uncharacterized protein
XP_009689285.1	0.0590209	0.529702	0.111423	0.0608299	0.515344	0.118037	0.11473	uncharacterized protein
XP_009689286.1	0.0858528	0.525392	0.163407	0.0770596	0.635504	0.121257	0.142332	ribosomal protein S12
XP_009689287.1	0.0156049	0.299252	0.0521461				0.0521461	uncharacterized protein
XP_009689288.1	0.0228299	0.927376	0.0246177	0.0180206	0.697289	0.0258438	0.025231	DNA replication licensing factor Mcm2
XP_009689289.1	0.0101957	0.396042	0.025744	0.00757094	0.276218	0.0274093	0.026577	ubiquitin carrier protein
XP_009689290.1	0.0457773	0.423238	0.10816	0.0382302	0.459212	0.0832516	0.095706	conserved hypothetical protein
XP_009689291.1	0.0568199	0.384165	0.147905				0.147905	conserved hypothetical protein
XP_009689292.1	0.0536567	0.918002	0.0584495	0.0547575	0.878179	0.0623534	0.060401	uncharacterized protein
XP_009689296.1	0.280416	0.0648331	4.32521				4.32521	hypothetical protein
XP_009689297.1	0.0347593	0.338464	0.102697				0.102697	conserved hypothetical protein
XP_009689298.1	0.112288	0.437287	0.256782	0.0801075	0.656655	0.121993	0.189388	conserved hypothetical protein
XP_009689299.1	0.111019	0.478467	0.23203	0.114777	0.448079	0.256154	0.244092	uncharacterized protein
XP_009689300.1	0.182547	0.268684	0.67941				0.67941	hypothetical protein
XP_009689301.1	0.0885254	0.628271	0.140903				0.140903	ABC transporter
XP_009689302.1	0.00292378	0.140947	0.0207437				0.0207437	transcription factor
XP_009689303.1	0.10439	0.510181	0.204614	0.0930466	0.690181	0.134815	0.169714	conserved hypothetical protein
XP_009689304.1	0.128509	1.09171	0.117713				0.117713	uncharacterized protein
XP_009689305.1	0.114359	0.510299	0.224102	0.117079	0.555794	0.210652	0.217377	uncharacterized protein
XP_009689307.1	0.102162	0.357905	0.285445	0.0905559	0.280898	0.32238	0.303913	uncharacterized protein
XP_009689308.1	0.126519	0.651205	0.194285	0.105226	0.452254	0.232671	0.213478	uncharacterized protein
XP_009689309.1	0.0251286	0.500686	0.0501882	0.0248255	0.305699	0.0812089	0.065699	conserved transmembrane protein
XP_009689310.1	0.190792	0.386409	0.493757				0.493757	conserved hypothetical protein
XP_009689311.1	0.0964097	0.445258	0.216525	0.13449	0.800628	0.167981	0.192253	uncharacterized protein
XP_009689312.1	0.0844993	0.846932	0.099771	0.0745399	0.823033	0.0905673	0.095169	conserved hypothetical protein
XP_009689313.1	0.070879	0.333867	0.212297	0.0420963	0.279083	0.150838	0.181567	conserved hypothetical protein
XP_009689314.1	0.0874288	0.544345	0.160613	0.113332	0.529372	0.214088	0.187351	uncharacterized protein
XP_009689315.1	0.048649	0.04455	1.09201				1.09201	hypothetical protein
XP_009689316.1	0.0054828	0.763122	0.0071847	0.00556763	0.917661	0.0060672	0.006626	elongation factor 2
XP_009689317.1	0.0342715	0.329738	0.103935				0.103935	conserved hypothetical protein
XP_009689318.1	0.0675397	0.756322	0.0893002	0.062725	0.849516	0.0738361	0.081568	silent information regulator protein Sir2
XP_009689319.1	0.0202754	0.91	0.0222807	0.010186	0.279867	0.0363959	0.029338	uncharacterized protein
XP_009689320.1	0.0106487	0.171345	0.0621478				0.0621478	SprA protein
XP_009689321.1	0.110322	0.405643	0.271969				0.271969	conserved hypothetical protein
XP_009689322.1	0.0640496	0.363431	0.176236	0.0640485	0.39144	0.163623	0.16993	uncharacterized protein
XP_009689323.1	0.003746	0.0370452	0.10112	0.0102311	0.156059	0.0655588	0.083339	uncharacterized protein
XP_009689324.1	0.0486835	0.419592	0.116026	0.0403959	0.388183	0.104064	0.110045	long-chain-fatty-acid--CoA ligase 5
XP_009689325.1	0.082834	0.613739	0.134966				0.134966	conserved hypothetical protein
XP_009689326.1	0.0518286	0.409656	0.126517	0.0368475	0.400292	0.0920515	0.109284	conserved hypothetical protein
XP_009689327.1	0.0725545	0.482536	0.150361				0.150361	conserved hypothetical protein
XP_009689330.1	0.0302974	0.44589	0.0679482	0.0320992	0.4503	0.0712841	0.069616	tryptophanyl-tRNA synthetase
XP_009689331.1	0.03046	0.246011	0.123816	0.0379222	0.249517	0.151982	0.137899	uncharacterized protein
XP_009689332.1	0.0763685	0.394259	0.193701	0.0880793	0.484166	0.18192	0.18781	1-deoxy-D-xylulose-5-phosphate synthase
XP_009689333.1	0.0327878	0.219278	0.149526				0.149526	uncharacterized protein
XP_009689334.1	0.0788064	0.430086	0.183234	0.079331	0.320855	0.247248	0.215241	uncharacterized protein
XP_009689335.1	0.0389006	0.304718	0.127661	0.0231767	0.182768	0.126809	0.127235	myosin a tail domain interacting protein mtip
XP_009689336.1	0.00485733	0.110131	0.0441052	7.46306E-05	0.0746306	0.001	0.022553	conserved hypothetical protein
XP_009689338.1	0.114971	0.414443	0.27741				0.27741	conserved hypothetical protein
XP_009689339.1	0.0720161	0.992678	0.0725473	0.0697168	1.12529	0.0619545	0.067251	conserved hypothetical protein
XP_009689341.1	0.0244678	0.568706	0.0430237	0.0254979	0.449383	0.0567399	0.049882	ATP-dependent helicase

protein_id	Chitose_ka	Chitose_ks	Chitose_kaks	Buffeli_ka	Buffeli_ks	Buffeli_kaks	mean_kaks	function
XP_009689342.1	0.0828914	0.603178	0.137424	0.0840627	0.57846	0.145322	0.141373	uncharacterized protein
XP_009689343.1	0.0229555	0.355901	0.0644997	0.0134397	0.463846	0.0289745	0.046737	choline kinase
XP_009689344.1	0.0733711	0.355113	0.206613	0.0619164	0.362028	0.171027	0.18882	choline kinase
XP_009689345.1	0.0790308	0.842766	0.0937754	0.0684196	0.916959	0.0746157	0.084196	conserved hypothetical protein
XP_009689346.1	0.057606	0.559529	0.102954	0.0490294	0.516974	0.0948391	0.098897	transcription elongation factor
XP_009689347.1	0.0537271	0.470364	0.114224	0.0629889	0.426785	0.147589	0.130907	conserved hypothetical protein
XP_009689348.1	0.0878437	0.568322	0.154567	0.0684418	0.680069	0.100639	0.127603	hypothetical protein
XP_009689349.1	0.0121938	0.279501	0.043627	0.0216333	0.315618	0.0685427	0.056085	uncharacterized protein
XP_009689350.1	0.0763797	0.434679	0.175715	0.0658323	0.377671	0.174311	0.175013	conserved hypothetical protein
XP_009689352.1	0.122105	0.485792	0.251353	0.145461	0.643664	0.225989	0.238671	uncharacterized protein
XP_009689353.1	0.112766	0.189295	0.595718	0.0638901	0.103174	0.619247	0.607482	hypothetical protein
XP_009689355.1	0.0545147	1.11377	0.0489459	0.0560361	1.17154	0.0478312	0.048389	uncharacterized protein
XP_009689356.1	0.146307	0.37872	0.38632	0.132613	0.409456	0.323877	0.355098	conserved hypothetical protein
XP_009689357.1	0.00927044	0.848111	0.0109307	0.0099304	1.16701	0.00850926	0.00972	ribosomal protein L3
XP_009689358.1	0.132168	1.79401	0.0736714				0.0736714	hypothetical protein
XP_009689359.1	0.138722	0.721835	0.19218	0.127762	0.648883	0.196895	0.194537	conserved hypothetical protein
XP_009689361.1	0.14862	0.44004	0.337743	0.175649	0.471441	0.372579	0.355161	uncharacterized protein
XP_009689362.1	0.100642	0.859273	0.117124	0.070748	0.910372	0.0777132	0.097419	uncharacterized protein
XP_009689363.1	0.0485344	0.46964	0.103344	0.0439766	0.315906	0.139208	0.121276	uncharacterized protein
XP_009689364.1	0.0908307	0.358935	0.253056	0.0458288	0.1993	0.229949	0.241503	uncharacterized protein
XP_009689366.1				0.0607109	0.769697	0.0788764	0.0788764	uncharacterized protein
XP_009689367.1	0.0927474	0.0731567	1.26779	NA	NA	NA	NA	hypothetical protein
XP_009689368.1	0.0808966	0.290557	0.278419	0.0752901	0.240971	0.312445	0.295432	DHHC-containing protein 20
XP_009689369.1	0.0852717	0.32281	0.264154	0.0869226	0.40373	0.215299	0.239727	uncharacterized protein
XP_009689370.1	0.0677167	0.664395	0.101922	0.0874117	0.73512	0.118908	0.110415	conserved hypothetical protein
XP_009689371.1	0.0266218	0.446615	0.0596079	0.064311	0.564103	0.114006	0.086807	conserved hypothetical protein
XP_009689372.1	0.0984888	0.276123	0.356685	0.112637	0.391293	0.287858	0.322271	conserved hypothetical protein
XP_009689373.1	0.13402	0.398851	0.336015	0.146842	0.562389	0.261103	0.298559	uncharacterized protein
XP_009689374.1	0.0722847	0.42858	0.168661				0.168661	hypothetical protein
XP_009689375.1	0.0225167	0.439625	0.0512181	0.00134098	0.232637	0.00576428	0.028491	secretory protein
XP_009689376.1	0.0152783	0.252926	0.0604062	0.00546665	0.304286	0.0179655	0.039186	proteasome subunit alpha type 5-2
XP_009689377.1	0.152489	0.478448	0.318715	0.153605	0.525395	0.29236	0.305538	conserved hypothetical protein
XP_009689378.1	0.149569	0.491366	0.304393	0.0913911	0.208913	0.437459	0.370926	conserved hypothetical protein
XP_009689379.1	0.138789	0.430877	0.322109	0.116937	0.518456	0.225549	0.273829	conserved hypothetical protein
XP_009689380.1	0.0248828	0.470299	0.0529086	0.0363479	0.48851	0.0744056	0.063657	uncharacterized protein
XP_009689381.1	0.103649	0.558389	0.185621	0.0819782	0.51965	0.157757	0.171689	uncharacterized protein
XP_009689383.1	0.122573	0.548456	0.223488	0.112327	0.495749	0.22658	0.225034	conserved hypothetical protein
XP_009689384.1	0.0952482	0.613645	0.155217	0.0745952	0.423826	0.176004	0.16561	uncharacterized protein
XP_009689385.1	0.0670751	0.639932	0.104816	0.0797492	0.699138	0.114068	0.109442	conserved hypothetical protein
XP_009689386.1	0.0909141	0.382734	0.237538				0.237538	uncharacterized protein
XP_009689387.1				NA	NA	NA	NA	hypothetical protein
XP_009689388.1	0.146724	0.584364	0.251083				0.251083	conserved hypothetical protein
XP_009689390.1	0.110531	0.829546	0.133243	0.103704	0.74336	0.139507	0.136375	uncharacterized protein
XP_009689391.1	0.00944265	0.597899	0.0157931	0.00928851	0.616744	0.0150606	0.015427	conserved hypothetical protein
XP_009689393.1	0.0336881	0.800186	0.0421004	0.0304317	0.880536	0.0345604	0.03833	DEAD-box helicase
XP_009689395.1	0.252031	0.205609	1.22578	0.211869	0.183629	1.15379	1.189785	hypothetical protein
XP_009689396.1	0.206303	0.296661	0.695417				0.695417	hypothetical protein
XP_009689397.1	0.088411	0.346517	0.255142	0.0860647	0.536518	0.160413	0.207778	conserved hypothetical protein
XP_009689398.1	0.0239361	0.770432	0.0310684	0.0293981	0.907054	0.0324105	0.031739	splicing factor
XP_009689399.1	0.0747237	0.253505	0.294762	0.0730016	0.412246	0.177083	0.235923	uncharacterized protein
XP_009689400.1	0.0817045	0.382712	0.213488	0.0723728	0.450113	0.160788	0.187138	uncharacterized protein
XP_009689403.1	0.0213301	1.05945	0.0201331	0.0242122	0.850822	0.0284574	0.024295	trehalose-6-phosphate synthase
XP_009689404.1	0.0250175	0.169296	0.147774	0.0118419	0.139721	0.0847542	0.116264	conserved hypothetical protein

protein_id	Chitose_ka	Chitose_ks	Chitose_kaks	Buffeli_ka	Buffeli_ks	Buffeli_kaks	mean_kaks	function
XP_009689405.1	0.10326	0.323231	0.319462	0.115552	0.296492	0.38973	0.354596	uncharacterized protein
XP_009689406.1	0.0184812	0.427652	0.0432155	0.012519	0.490326	0.025532	0.034374	uncharacterized protein
XP_009689407.1	0.0912293	0.472387	0.193124	0.0623456	0.518181	0.120316	0.15672	uncharacterized protein
XP_009689408.1	0.120606	0.420816	0.2866	0.129252	0.472767	0.273394	0.279997	NEDD8-activating enzyme E1 catalytic subunit
XP_009689409.1	0.210249	0.144473	1.45528	0.174504	0.12987	1.34368	1.39948	hypothetical protein
XP_009689410.1	0.116746	0.614121	0.190102	0.0895513	0.435976	0.205404	0.197753	conserved hypothetical protein
XP_009689411.1	0.0965327	0.209613	0.460529				0.460529	hypothetical protein
XP_009689412.1	0.051456	0.317886	0.16187	0.0457614	0.314396	0.145554	0.153712	uncharacterized protein
XP_009689413.1	0.0388446	0.250842	0.154857				0.154857	conserved hypothetical protein
XP_009689414.1	0.0430723	0.30536	0.141054	0.107983	0.382158	0.282562	0.211808	conserved hypothetical protein
XP_009689415.1	0.108234	0.70398	0.153746	0.088367	0.723489	0.12214	0.137943	uncharacterized protein
XP_009689416.1	0.0050441	0.407262	0.0123854				0.0123854	transcription initiation factor
XP_009689417.1	0.0924429	0.506022	0.182686	0.11207	0.616972	0.181645	0.182165	conserved hypothetical protein
XP_009689419.1	0.0191402	0.0726012	0.263634				0.263634	hypothetical protein
XP_009689420.1	0.0549334	0.469647	0.116967	0.0563604	0.479728	0.117484	0.117226	uncharacterized protein
XP_009689421.1	0.0434002	0.69327	0.0626022	0.0587743	0.657286	0.0894196	0.076011	adenylate kinase
XP_009689422.1	0.0258623	0.381583	0.0677763				0.0677763	diphthine synthase
XP_009689423.1	0.0536913	0.443702	0.121008	0.0169463	0.211002	0.0803136	0.100661	uncharacterized protein
XP_009689424.1	0.0609721	0.370979	0.164355	0.0628057	0.229873	0.273219	0.218787	uncharacterized protein
XP_009689425.1	0.0155321	0.38175	0.0406866	0.0492553	0.324565	0.151758	0.096222	elongation factor
XP_009689426.1	0.0372335	0.172473	0.21588	0.0191404	0.0833091	0.229752	0.222816	Rab5 GTPase
XP_009689427.1	0.0289648	0.714304	0.0405496	0.0259208	0.637063	0.040688	0.040619	DEAD-box family helicase
XP_009689428.1	0.000331663	0.331663	0.001	0.00030942	0.30942	0.001	0.001	Dim1 protein
XP_009689429.1	0.0696543	0.674435	0.103278	0.0713415	1.0416	0.0684921	0.085885	conserved hypothetical protein
XP_009689430.1	0.155553	0.574846	0.2706	0.0900976	0.25766	0.349676	0.310138	hypothetical protein
XP_009689431.1	0.0282932	0.327536	0.0863818	0.0465993	0.433642	0.10746	0.096921	conserved hypothetical protein
XP_009689432.1	0.0522896	0.691957	0.0755677	0.0486969	0.596475	0.0816412	0.078604	conserved hypothetical protein
XP_009689433.1	0.058564	0.444091	0.131874	0.0650251	0.492734	0.131968	0.131921	uncharacterized protein
XP_009689434.1	0.0646259	0.508474	0.127098	0.0678817	0.39081	0.173695	0.150396	uncharacterized protein
XP_009689435.1	0.148185	0.319203	0.464235				0.464235	conserved hypothetical protein
XP_009689436.1	0.0327384	0.118114	0.277176				0.277176	conserved hypothetical protein
XP_009689437.1	0.0453446	0.224986	0.201544				0.201544	hypothetical protein
XP_009689438.1	0.0607093	0.129232	0.469771	0.0602512	0.310459	0.194071	0.331921	conserved hypothetical protein
XP_009689439.1	0.00373558	0.24036	0.0155416				0.0155416	DNA-directed RNA polymerase III
XP_009689440.1	0.10313	0.40798	0.252781	0.0867777	0.417601	0.2078	0.230291	hypothetical protein
XP_009689441.1	0.000414046	0.414046	0.001	0.000457395	0.457395	0.001	0.001	40S ribosomal protein S25
XP_009689442.1	0.0758416	0.584179	0.129826	0.0102253	0.0583603	0.175211	0.152519	uncharacterized protein
XP_009689443.1	0.0683658	0.435538	0.156969	0.0664312	0.265901	0.249834	0.203402	uncharacterized protein
XP_009689444.1	0.10855	0.369493	0.29378	0.0903115	0.389749	0.231717	0.262748	conserved hypothetical protein
XP_009689445.1	0.0926305	0.371411	0.249401				0.249401	conserved hypothetical protein
XP_009689446.1	0.092899	0.769242	0.120767				0.120767	hypothetical protein
XP_009689447.1	0.0641554	0.342706	0.187202	0.0811001	0.288641	0.280972	0.234087	uncharacterized protein
XP_009689448.1	0.0339622	0.0860936	0.394481	0.00801968	0.0493382	0.162545	0.278513	hypothetical protein
XP_009689449.1	0.00846943	0.972102	0.00871249	0.00777732	1.76723	0.00440085	0.006557	conserved hypothetical protein
XP_009689450.1				0.173667	1.13403	0.153142	0.153142	ABC transporter
XP_009689453.1	0.125043	0.311057	0.401994	0.0836965	0.201255	0.415873	0.408934	conserved hypothetical protein
XP_009689454.1	0.0672387	0.569347	0.118098	0.0618341	0.510346	0.121161	0.119629	glycerophosphoryl diester phosphodiesterase
XP_009689455.1	0.0233059	0.7106	0.0327975	0.0244274	0.658093	0.0371185	0.034958	adaptor protein
XP_009689456.1	0.116129	0.511774	0.226914	0.137329	0.666506	0.206044	0.216479	conserved hypothetical protein
XP_009689457.1	0.0935513	0.482493	0.193891	0.081219	0.409568	0.198304	0.196098	conserved hypothetical protein
XP_009689458.1	0.0456993	0.366395	0.124727	0.0313288	0.162125	0.193239	0.158983	ribosomal protein S18
XP_009689459.1	0.061686	0.723472	0.0852639	0.0594793	0.721558	0.0824317	0.083848	uncharacterized protein
XP_009689460.1	0.0592191	0.298669	0.198277	0.0318047	0.127234	0.249971	0.224124	molecular chaperone DnaJ

protein_id	Chitose_ka	Chitose_ks	Chitose_kaks	Buffeli_ka	Buffeli_ks	Buffeli_kaks	mean_kaks	function
XP_009689461.1	0.0805836	0.366917	0.219623	0.0418011	0.336612	0.124182	0.171903	minichromosome maintenance protein
XP_009689462.1	0.0859435	0.483239	0.177849	0.081352	0.505049	0.161077	0.169463	flavin-containing amine oxidase
XP_009689463.1	0.0124958	0.41434	0.0301584	0.013656	0.484444	0.0281891	0.029174	DNA replication licensing factor
XP_009689464.1	0.0060093	0.517066	0.0116219	0.000808496	0.808496	0.001	0.006311	ubiquitin
XP_009689465.1	0.0368735	0.812792	0.0453664	0.0364337	0.918407	0.0396706	0.042519	cation ATPase
XP_009689466.1	0.0705269	0.415597	0.1697	0.0468372	0.302278	0.154948	0.162324	conserved hypothetical protein
XP_009689467.1	0.0826847	0.480329	0.172142				0.172142	uncharacterized protein
XP_009689468.1	0.0340792	0.67639	0.0503839	0.0140117	0.979072	0.0143112	0.032348	30S ribosomal protein S16
XP_009689469.1	0.152663	0.552526	0.276299	0.12662	0.517256	0.244792	0.260545	conserved hypothetical protein
XP_009689470.1	0.0910468	0.356182	0.255619	0.0494121	0.227034	0.217642	0.23663	conserved hypothetical protein
XP_009689471.1	0.0040243	0.910436	0.00442019	0.00505822	0.734275	0.00688872	0.005654	clathrin-adaptor chain
XP_009689472.1	0.000458576	0.458576	0.001	0.000195793	0.195793	0.001	0.001	26S proteasome subunit
XP_009689473.1	0.0981901	0.623671	0.157439	0.104107	0.572728	0.181774	0.169606	conserved hypothetical protein
XP_009689474.1	0.0323741	0.826567	0.039167	0.0246673	0.842677	0.0292726	0.03422	26S proteasome regulatory subunit
XP_009689475.1	0.110363	0.665348	0.165873	0.0987434	0.733038	0.134704	0.150288	uncharacterized protein
XP_009689476.1	0.0362395	0.364147	0.099519	0.0328741	0.192355	0.170904	0.135211	uncharacterized protein
XP_009689477.1	0.0495047	0.5774	0.0857373	0.0493554	0.514824	0.0958686	0.090803	molecular chaperone protein
XP_009689478.1	0.0555797	0.337465	0.164698	0.0470518	0.321577	0.146316	0.155507	uncharacterized protein
XP_009689479.1	0.0557967	1.07315	0.0519934	0.0493999	0.71061	0.0695176	0.060756	uncharacterized protein
XP_009689480.1	0.0597434	0.508882	0.117401	0.0495874	0.34232	0.144857	0.131129	uncharacterized protein
XP_009689481.1	0.101679	1.2028	0.0845353	0.0896458	1.12233	0.0798748	0.082205	conserved hypothetical protein
XP_009689482.1	0.0354905	0.498579	0.0711834				0.0711834	conserved hypothetical protein
XP_009689483.1	0.0789724	0.613765	0.128669	0.0677864	0.455165	0.148927	0.138798	DEAD-box family helicase
XP_009689484.1	0.0627695	0.467315	0.134319	0.0457228	0.46818	0.0976605	0.11599	uncharacterized protein
XP_009689485.1	0.0868694	0.458819	0.189333	0.0627631	0.382957	0.163891	0.176612	conserved hypothetical protein
XP_009689486.1	0.0172459	0.153902	0.112058	0.0479041	0.191813	0.249744	0.180901	phosphotyrosyl phosphatase activator protein
XP_009689487.1	0.0614181	0.732699	0.0838244	0.0617651	0.69245	0.0891979	0.086511	uncharacterized protein
XP_009689490.1	0.0320866	0.395571	0.0811146	0.0253512	0.236347	0.107263	0.094189	uncharacterized protein
XP_009689491.1	0.0821794	0.430392	0.190941	0.0816985	0.36758	0.22226	0.206601	conserved hypothetical protein
XP_009689492.1	0.0493603	0.445418	0.110818	0.0525877	0.433465	0.12132	0.116069	coatamer subunit beta
XP_009689493.1	0.0530527	0.638577	0.0830796	0.0469204	0.503553	0.0931788	0.088129	hypothetical protein
XP_009689496.1	0.0425049	0.370728	0.114652	0.0678166	0.355268	0.190888	0.15277	ribosomal protein L15
XP_009689497.1	0.0654033	0.332913	0.196458	0.0325307	0.205377	0.158395	0.177426	Plasmodium falciparum CPW-WPC repeat containing protein
XP_009689500.1	0.0184575	0.0850391	0.217047				0.217047	conserved hypothetical protein
XP_009689503.1	0.0618498	0.125358	0.493387				0.493387	hypothetical protein
XP_009689505.1	0.0135912	0.324259	0.0419145	0.00835946	0.325007	0.0257209	0.033818	cytochrome c oxidase subunit 2
XP_009689506.1	0.0331563	0.110532	0.299969	0.0305463	0.0840064	0.363619	0.331794	50S ribosomal protein L17
XP_009689507.1	0.0580183	0.48696	0.119144	0.0493312	0.498672	0.0989252	0.109035	predicted protein
XP_009689509.1	0.0725788	0.620266	0.117012	0.0590149	0.671784	0.087848	0.10243	conserved hypothetical protein
XP_009689510.1	0.0546135	0.552823	0.0987902	0.0458047	0.504214	0.0908437	0.094817	protein kinase
XP_009689511.1	0.0473728	0.88866	0.0533081	0.0470254	0.725408	0.0648262	0.059067	succinyl-Coa ligase%2C subunit
XP_009689512.1	0.0193611	0.210539	0.0919599				0.0919599	uncharacterized protein
XP_009689513.1	0.0117513	0.308162	0.0381336	0.0147178	0.307546	0.0478556	0.042995	60S ribosomal protein L14
XP_009689514.1	0.0766522	0.578307	0.132546	0.0992634	0.749722	0.1324	0.132473	conserved hypothetical protein
XP_009689515.1	0.0754952	0.547688	0.137843	0.0837309	0.640656	0.130696	0.134269	uncharacterized protein
XP_009689517.1	0.0214344	0.377495	0.0567806				0.0567806	uncharacterized protein
XP_009689518.1	0.0866454	0.430001	0.201501				0.201501	conserved hypothetical protein
XP_009689519.1	0.068916	0.573817	0.120101	0.0484102	0.497153	0.0973749	0.108738	conserved hypothetical protein
XP_009689520.1	0.032404	0.635173	0.0510161				0.0510161	hypothetical protein
XP_009689521.1	0.0875759	0.469188	0.186654	0.0667114	0.440167	0.151559	0.169106	conserved hypothetical protein
XP_009689522.1	0.0832524	0.623099	0.13361	0.0732224	0.523642	0.139833	0.136721	conserved hypothetical protein
XP_009689523.1	0.049254	0.560206	0.0879212	0.0543517	0.627931	0.0865567	0.087239	uncharacterized protein
XP_009689524.1	0.0521743	0.34019	0.153368	0.067263	0.347888	0.193347	0.173357	conserved hypothetical protein

protein_id	Chitose_ka	Chitose_ks	Chitose_kaks	Buffeli_ka	Buffeli_ks	Buffeli_kaks	mean_kaks	function
XP_009689525.1	0.0041386	0.438387	0.0094405	0.00923735	0.20151	0.0458407	0.027641	ribonucleotide reductase R2 subunit/ribonucleoside-diphosphate reductase small chain
XP_009689526.1	0.00100834	0.655034	0.00153938	0.00197605	0.498128	0.00396696	0.002753	elongation factor 1-alpha
XP_009689527.1	0.0406957	0.133009	0.305963				0.305963	uncharacterized protein
XP_009689528.1	0.00152382	0.386829	0.00393925	0.00036624	0.36624	0.001	0.00247	cell division control protein 2 homolog
XP_009689529.1	0.0226857	0.267182	0.0849074	0.0370166	0.221091	0.167427	0.126167	molecular chaperone DnaJ
XP_009689530.1	0.0376215	0.404627	0.092978	0.0312701	0.369205	0.0846958	0.088837	uncharacterized protein
XP_009689531.1	0.0889988	0.788825	0.112825	0.0900788	0.836905	0.107633	0.110229	uncharacterized protein
XP_009689532.1	0.0176962	0.282414	0.0626605	0.0145723	0.295312	0.0493455	0.056003	ubiquitin carrier protein
XP_009689533.1	0.0117033	0.55217	0.0211951	0.018556	0.525393	0.0353184	0.028257	mitochondrial inner membrane protein
XP_009689534.1	0.0796295	0.491341	0.162066	0.0480377	0.418327	0.114833	0.13845	uncharacterized protein
XP_009689535.1	0.044347	0.175374	0.252871				0.252871	uncharacterized protein
XP_009689536.1	0.130498	0.320298	0.407426	0.118946	0.253662	0.468917	0.438171	conserved hypothetical protein
XP_009689537.1	0.107548	0.641369	0.167684	0.119532	0.418631	0.285531	0.226607	conserved hypothetical protein
XP_009689538.1	0.111728	0.456802	0.244588	0.0989874	0.474219	0.208738	0.226663	conserved hypothetical protein
XP_009689539.1	0.0188872	0.358911	0.0526236				0.0526236	disrupter of silencing protein
XP_009689540.1	0.0411774	1.00382	0.0410207	0.0377514	0.997376	0.0378507	0.039436	uncharacterized protein
XP_009689541.1	0.0488171	0.0800396	0.609912				0.609912	conserved hypothetical protein
XP_009689542.1	0.0675852	0.421952	0.160173	0.0588839	0.489268	0.120351	0.140262	conserved hypothetical protein
XP_009689543.1	0.075235	0.448956	0.167578	0.0567838	0.458946	0.123726	0.145652	conserved hypothetical protein
XP_009689544.1	0.059638	0.579261	0.102955	0.0468551	0.563505	0.0831495	0.093052	uncharacterized protein
XP_009689545.1	0.0380341	0.441191	0.0862078	0.0394493	0.383748	0.1028	0.094504	uncharacterized protein
XP_009689546.1	0.0424072	0.700055	0.0605769	0.0327265	0.611405	0.0535268	0.057052	ribonucleolar protein
XP_009689547.1	0.0827399	0.496201	0.166747	0.0702314	0.590678	0.1189	0.142823	conserved hypothetical protein
XP_009689548.1	0.0491991	0.524459	0.0938093	0.0452673	0.457134	0.099024	0.096417	histone acetyltransferase
XP_009689549.1	0.0330733	0.638849	0.0517702	0.0266495	0.559663	0.0476171	0.049694	heat-shock protein
XP_009689550.1	0.0153797	1.2405	0.012398	0.012192	1.25835	0.00968893	0.011043	clathrin assembly protein
XP_009689551.1	0.0730691	0.609365	0.11991	0.0557253	0.497982	0.111902	0.115906	conserved hypothetical protein
XP_009689552.1	0.0959115	0.586801	0.163448	0.0870797	0.575799	0.151233	0.15734	uncharacterized protein
XP_009689553.1	0.00214626	0.244204	0.00878882	0.00270226	0.333196	0.00811013	0.008449	uncharacterized protein
XP_009689554.1	0.0542725	0.415061	0.130758	0.0438757	0.448318	0.0978673	0.114313	conserved hypothetical protein
XP_009689555.1	0.0751248	0.328292	0.228835				0.228835	conserved hypothetical protein
XP_009689556.1	0.0512009	0.390775	0.131024	0.0525025	0.358877	0.146297	0.138661	tyrosyl-tRNA synthetase
XP_009689557.1	0.0290082	0.428253	0.0677362	0.0202086	0.47721	0.0423473	0.055042	conserved hypothetical protein
XP_009689558.1	0.084604	0.630758	0.134131	0.0721009	0.617421	0.116778	0.125454	conserved hypothetical protein
XP_009689559.1	0.046928	0.501239	0.093624	0.0231062	0.320043	0.0721971	0.082911	clathrin assembly protein
XP_009689560.1	0.00338415	0.500232	0.00676516	0.00347451	0.349235	0.00994891	0.008357	eukaryotic translation initiation factor
XP_009689561.1	0.0793105	0.434883	0.182372	0.0565188	0.297931	0.189704	0.186038	conserved hypothetical protein
XP_009689562.1	0.074806	0.376776	0.198542	0.0356037	0.206975	0.172019	0.185281	conserved hypothetical protein
XP_009689563.1	0.0750694	1.02613	0.0731577	0.0856546	0.840614	0.101895	0.087526	uncharacterized protein
XP_009689564.1	0.0560284	0.19822	0.282657	0.054334	0.259284	0.209554	0.246105	conserved hypothetical protein
XP_009689565.1	0.0385554	0.492437	0.0782952	0.0302107	0.65121	0.0463916	0.062343	chromatin assembly factor subunit
XP_009689566.1	0.0323686	0.752209	0.0430314	0.0284104	0.67191	0.042283	0.042657	myosin
XP_009689568.1	0.0478812	0.691903	0.0692022	0.040771	0.65262	0.0624728	0.065838	1-phosphatidylinositol-4%2C5-bisphosphate phosphodiesterase
XP_009689569.1	0.0859329	0.648253	0.132561	0.0755881	0.504297	0.149888	0.141225	conserved hypothetical protein
XP_009689570.1	0.0392915	0.101743	0.386184				0.386184	conserved hypothetical protein
XP_009689571.1	0.0361744	0.524859	0.0689222	0.0528881	0.537783	0.0983448	0.083633	conserved hypothetical protein
XP_009689572.1	0.0282604	0.462839	0.0610588	0.0127043	0.455836	0.0278704	0.044465	transcriptional adaptor
XP_009689573.1	0.109003	0.316906	0.343962	0.0792955	0.340663	0.232768	0.288365	conserved hypothetical protein
XP_009689577.1	0.0231085	0.399292	0.0578736	0.0310548	0.498181	0.0623363	0.060105	cyclin-dependent kinase CRK3
XP_009689578.1	0.112585	0.186902	0.602374	0.0623029	0.187347	0.332553	0.467463	hypothetical protein
XP_009689579.1	0.0243626	0.469474	0.0518933	0.0162765	0.377585	0.0431069	0.0475	uncharacterized protein
XP_009689581.1	0.0461842	0.615508	0.0750342	0.0424738	0.561695	0.0756172	0.075326	uncharacterized protein
XP_009689582.1	0.0404252	0.561793	0.0719575	0.029667	0.604839	0.0490494	0.060503	phosphomannomutase

protein_id	Chitose_ka	Chitose_ks	Chitose_kaks	Buffeli_ka	Buffeli_ks	Buffeli_kaks	mean_kaks	function
XP_009689583.1	0.065839	0.276614	0.238017	0.0609976	0.254298	0.239867	0.238942	hypothetical protein
XP_009689584.1	0.0756238	0.582243	0.129884	0.0833258	0.627143	0.132866	0.131375	uncharacterized protein
XP_009689585.1	0.0556239	0.529663	0.105018	0.0600836	0.585387	0.102639	0.103828	uncharacterized protein
XP_009689586.1	0.0053262	0.240771	0.0221214	0.000243567	0.243567	0.001	0.011561	ribosomal protein L11
XP_009689587.1	0.0247306	0.778185	0.0317798	0.0168906	0.746052	0.02264	0.02721	actin
XP_009689588.1	0.0667163	0.495786	0.134567	0.043981	0.687492	0.0639731	0.09927	conserved hypothetical protein
XP_009689589.1	0.0313162	0.351491	0.0890955	0.0260882	0.304332	0.0857227	0.087409	uncharacterized protein
XP_009689590.1	0.0457061	0.517815	0.0882672	0.0368418	0.486192	0.0757763	0.082022	conserved hypothetical protein
XP_009689591.1	0.0245423	0.198493	0.123643	0.0473042	0.300666	0.157331	0.140487	conserved hypothetical protein
XP_009689592.1	0.0404698	0.453706	0.0891982	0.0322148	0.40639	0.0792706	0.084234	uncharacterized protein
XP_009689593.1	0.0622947	0.447715	0.139139	0.0576715	0.415917	0.138661	0.1389	conserved hypothetical protein
XP_009689594.1	0.0127379	0.607276	0.0209755	0.000904404	0.904404	0.001	0.010988	conserved hypothetical protein
XP_009689595.1	0.0140046	0.616487	0.0227168	0.0070013	0.654374	0.0106992	0.016708	pre-mRNA splicing factor
XP_009689596.1	0.082695	0.664108	0.12452				0.12452	uncharacterized protein
XP_009689597.1	0.0228409	0.342481	0.0666924	0.0211146	0.324582	0.0650516	0.065872	conserved hypothetical protein
XP_009689598.1	0.00450324	0.575211	0.00782885	0.000683928	0.683928	0.001	0.004414	mitochondrial import inner membrane translocase subunit
XP_009689599.1	0.0818362	0.590414	0.138608	0.0778047	0.524191	0.148428	0.143518	conserved hypothetical protein
XP_009689600.1	0.0526246	0.475528	0.110666	0.0297671	0.322806	0.0922135	0.10144	conserved hypothetical protein
XP_009689601.1	0.0320312	0.303462	0.105553	0.0351265	0.290208	0.121039	0.113296	aspartyl protease
XP_009689602.1	0.00613538	0.237897	0.02579	0.00283329	0.236855	0.0119621	0.018876	ubiquitin carrier protein
XP_009689603.1	0.0156501	0.65228	0.0239929	0.015538	0.407682	0.0381131	0.031053	recombinational repair protein
XP_009689604.1	0.00542716	0.452986	0.0119809	0.0131834	0.612475	0.0215247	0.016753	uncharacterized protein
XP_009689605.1	0.0733446	0.873132	0.0840018	0.0597845	0.600436	0.0995684	0.091785	conserved hypothetical protein
XP_009689606.1	0.0412361	0.590671	0.0698123	0.0359671	0.809914	0.0444086	0.05711	ubiquitination-mediated degradation component
XP_009689607.1	0.0373817	0.830189	0.045028	0.0294322	0.81362	0.0361744	0.040601	DNA-directed RNA polymerase
XP_009689608.1	0.0218775	0.532234	0.041105	0.0194937	0.522596	0.0373016	0.039203	ADP-ribosylation factor GTPase activating protein
XP_009689609.1	0.0551967	0.368726	0.149696	0.0358167	0.413202	0.0866808	0.118188	uncharacterized protein
XP_009689610.1	0.0291051	0.777967	0.0374118	0.0286267	0.990711	0.0288951	0.033153	complexed with cef1p
XP_009689611.1	0.0543355	0.710966	0.0764249	0.0673483	0.780319	0.0863086	0.081367	uncharacterized protein
XP_009689612.1	0.0205937	0.525737	0.0391711	0.0162051	0.446759	0.0362727	0.037722	cyclophilin peptidyl-prolyl cis-trans isomerase protein
XP_009689613.1	0.0379556	0.230554	0.164627	0.0560241	0.379454	0.147644	0.156135	conserved hypothetical protein
XP_009689614.1	0.0766221	0.369862	0.207164	0.100942	0.405801	0.248747	0.227955	uncharacterized protein
XP_009689615.1	0.0703224	0.543984	0.129273	0.0496136	0.353289	0.140434	0.134854	uncharacterized protein
XP_009689616.1	0.0307474	1.06873	0.0287701	0.0362605	1.03555	0.0350157	0.031893	phosphatidylinositol 4-kinase
XP_009689617.1	0.0602769	0.69275	0.0870111	0.0525617	0.822645	0.0638935	0.075452	conserved hypothetical protein
XP_009689618.1	0.0427297	0.475398	0.0898819	0.0413905	0.511425	0.0809317	0.085407	DEAD-box family helicase
XP_009689619.1	0.0590731	0.698033	0.084628	0.061451	0.624943	0.0983307	0.091479	conserved hypothetical protein
XP_009689620.1	0.0862431	0.347834	0.247943	0.0535068	0.293278	0.182444	0.215193	uncharacterized protein
XP_009689621.1	0.0977063	0.452804	0.21578	0.106161	0.455073	0.233283	0.224531	conserved hypothetical protein
XP_009689622.1	0.0187172	0.551611	0.0339318	0.0129467	0.45287	0.0285882	0.03126	uncharacterized protein
XP_009689623.1	0.0170264	0.0886397	0.192086	0.00552237	0.0432905	0.127565	0.159826	uncharacterized protein
XP_009689624.1	0.0110106	0.542036	0.0203134	0.0124229	0.520271	0.0238777	0.022096	nonsense-mediated mRNA decay protein
XP_009689625.1	0.0267942	0.57925	0.0462566	0.0220599	0.592321	0.0372432	0.04175	uncharacterized protein
XP_009689626.1	0.11879	0.867882	0.136874	0.103467	0.853379	0.121244	0.129059	tRNA-2'-O-ribose methyltransferase
XP_009689627.1	0.0278544	0.541963	0.0513954	0.0233269	0.557708	0.0418264	0.046611	uncharacterized protein
XP_009689628.1	0.0180417	0.227979	0.0791377	NA	NA	NA	NA	conserved hypothetical protein
XP_009689629.1	0.0412068	0.625819	0.0658445	0.0389955	0.728598	0.0535213	0.059683	uncharacterized protein
XP_009689630.1	0.0389007	0.911856	0.042661	0.0325368	0.723857	0.0449492	0.043805	uncharacterized protein
XP_009689631.1	0.0665769	0.532335	0.125066	0.0581614	0.55665	0.104485	0.114776	uncharacterized protein
XP_009689632.1	0.0252736	0.52577	0.0480698	0.0278088	0.397497	0.0699597	0.059015	conserved hypothetical protein
XP_009689633.1	0.0288055	0.396157	0.0727124	0.0286245	0.421067	0.067981	0.070347	conserved hypothetical protein
XP_009689634.1	0.0253043	0.38854	0.0651267	0.0210396	0.298223	0.0705499	0.067838	5'-3' exoribonuclease 1
XP_009689635.1	0.0743015	0.505629	0.146949	0.0552935	0.473055	0.116886	0.131917	uncharacterized protein

protein_id	Chitose_ka	Chitose_ks	Chitose_kaks	Buffeli_ka	Buffeli_ks	Buffeli_kaks	mean_kaks	function
XP_009689636.1	0.00996776	0.572477	0.0174116	0.00208994	0.711939	0.00293557	0.010174	26S proteasome subunit 4
XP_009689637.1	0.0189199	0.494591	0.0382537	0.00489424	0.256295	0.0190961	0.028675	uncharacterized protein
XP_009689638.1	0.10167	0.698435	0.145568	0.0772742	0.562459	0.137386	0.141477	conserved hypothetical protein
XP_009689639.1	0.0929974	0.378449	0.245733	0.0454162	0.213569	0.212653	0.229193	conserved hypothetical protein
XP_009689640.1	0.0469825	0.637592	0.0736874	0.0316611	0.592998	0.0533915	0.063539	uncharacterized protein
XP_009689641.1	0.0409013	0.395644	0.103379	0.0544567	0.342474	0.15901	0.131194	DNA repair protein
XP_009689642.1	0.0190302	0.592537	0.0321165	0.0151854	0.56066	0.0270848	0.029601	uncharacterized protein
XP_009689643.1	0.004228	0.469985	0.00899602	0.00650586	0.315904	0.0205944	0.014795	signal recognition particle protein 2
XP_009689644.1	0.01043	0.30845	0.0338143	0.0118885	0.240141	0.0495063	0.04166	eukaryotic translation initiation factor 2 subunit alpha
XP_009689645.1	0.0441674	0.376787	0.117221	0.00936729	0.362054	0.0258726	0.071547	uncharacterized protein
XP_009689646.1	0.104136	0.604799	0.172182	0.0763381	0.698378	0.109308	0.140745	conserved hypothetical protein
XP_009689647.1	0.0738527	0.284701	0.259405	0.0591391	0.33787	0.175035	0.21722	uncharacterized protein
XP_009689648.1	0.0317582	0.303461	0.104653	0.0353742	0.320739	0.11029	0.107471	uncharacterized protein
XP_009689649.1	0.0304049	0.471054	0.0645466	0.0647489	0.264563	0.244739	0.154643	hypothetical protein
XP_009689650.1	0.0495962	0.358925	0.13818	0.0380156	0.445533	0.085326	0.111753	uncharacterized protein
XP_009689651.1	0.0152827	0.187299	0.0815953				0.0815953	predicted protein
XP_009689652.1	0.00474841	0.390641	0.0121554	0.00904404	0.394371	0.0229328	0.017544	ubiquinol-cytochrome C reductase complex subunit-like protein
XP_009689653.1	0.0477448	0.600883	0.0794578	0.0590583	0.568526	0.10388	0.091669	uncharacterized protein
XP_009689654.1	0.0205632	0.909296	0.0226145	0.0197957	1.08887	0.0181801	0.020397	GMP synthase
XP_009689655.1	0.0682952	0.385048	0.177368	0.044746	0.492391	0.0908749	0.134121	protein disulfide isomerase
XP_009689656.1	0.0598284	0.582161	0.10277	0.0588452	0.626323	0.0939535	0.098362	conserved hypothetical protein
XP_009689657.1	0.0232292	0.482878	0.0481056	0.0155199	0.494823	0.0313645	0.039735	splicing factor
XP_009689658.1	0.0255023	1.57739	0.0161674	0.0302692	1.94077	0.0155965	0.015882	uncharacterized protein
XP_009689659.1	0.069761	0.372273	0.187392	0.067573	0.354436	0.190649	0.189021	conserved hypothetical protein
XP_009689660.1	0.188709	0.448696	0.420571	0.152393	0.220343	0.691616	0.556094	conserved hypothetical protein
XP_009689661.1	0.0185427	0.379332	0.0488826				0.0488826	conserved hypothetical protein
XP_009689662.1	0.0544275	0.329991	0.164936	0.0425604	0.259475	0.164025	0.164481	DNA repair protein
XP_009689663.1	0.0511091	0.757767	0.067447	0.0465531	0.736038	0.0632482	0.065348	uncharacterized protein
XP_009689664.1	0.17074	0.327733	0.520974				0.520974	conserved hypothetical protein
XP_009689665.1	0.051884	0.0316184	1.64094				1.64094	hypothetical protein
XP_009689666.1	0.0912484	0.307023	0.297204	0.159326	0.350532	0.454526	0.375865	hypothetical protein
XP_009689667.1	0.03326	0.519892	0.0639749	0.0333686	0.483965	0.0689483	0.066462	conserved hypothetical protein
XP_009689668.1	0.0214496	0.483963	0.0443207	0.0138614	0.449149	0.0308615	0.037591	proteasome subunit beta type
XP_009689669.1	0.0455094	0.297694	0.152873	0.0530409	0.380096	0.139546	0.146209	conserved hypothetical protein
XP_009689670.1	0.0028429	0.293852	0.00967461				0.00967461	Rab GTPase
XP_009689671.1	0.0627036	0.59249	0.105831	0.0727536	0.845069	0.086092	0.095962	uncharacterized protein
XP_009689672.1	0.0690147	0.580047	0.118981	0.0342349	0.405382	0.0844508	0.101716	protein kinase
XP_009689673.1	0.0202803	0.519341	0.03905	0.0102632	0.590513	0.0173801	0.028215	casein kinase II subunit beta
XP_009689674.1	0.0879047	0.346288	0.253849	0.0788972	0.239728	0.329111	0.29148	conserved hypothetical protein
XP_009689675.1	0.0905041	0.437777	0.206735	0.0710991	0.455428	0.156115	0.181425	conserved hypothetical protein
XP_009689676.1	0.0100438	0.203696	0.0493077	0.00602921	0.191547	0.0314764	0.040392	eukaryotic translation initiation factor
XP_009689677.1	0.000390125	0.390125	0.001	0.000346425	0.346425	0.001	0.001	Tim10
XP_009689678.1	0.0384628	0.52007	0.073957	0.0361028	0.437343	0.0825501	0.078254	cytochrome C1 heme lyase
XP_009689679.1	0.0335806	0.328388	0.102259	0.0127408	0.411687	0.0309478	0.066603	Pre-rRNA-processing protein PNO1
XP_009689680.1	0.0779266	0.335516	0.232259	0.0620194	0.422596	0.146758	0.189508	Conserved hypothetical protein
XP_009689681.1	0.00522388	0.45032	0.0116004	0.000665772	0.665772	0.001	0.0063	uncharacterized protein
XP_009689682.1	0.099414	0.371186	0.267828	0.0632394	0.315188	0.20064	0.234234	conserved hypothetical protein
XP_009689683.1	0.0407448	0.36742	0.110894	0.0432215	0.41926	0.10309	0.106992	uncharacterized protein
XP_009689685.1	0.0224796	0.593734	0.0378614	0.0175404	0.549594	0.0319153	0.034888	conserved hypothetical protein
XP_009689686.1	0.0265826	0.28982	0.0917212	0.0273396	0.293908	0.0930207	0.092371	conserved hypothetical protein
XP_009689687.1	0.0470621	0.247355	0.190261				0.190261	conserved hypothetical protein
XP_009689689.1	0.0876427	0.581934	0.150606	0.092857	0.75672	0.12271	0.136658	conserved hypothetical protein
XP_009689690.1	0.100102	0.603736	0.165803	0.0603591	0.492502	0.122556	0.14418	exosome ribonuclease PH

protein_id	Chitose_ka	Chitose_ks	Chitose_kaks	Buffeli_ka	Buffeli_ks	Buffeli_kaks	mean_kaks	function
XP_009689691.1	0.0229765	0.576952	0.0398239	0.0206963	0.467561	0.0442643	0.042044	DNA polymerase delta catalytic subunit
XP_009689692.1	0.0716989	0.843034	0.0850487	0.0544716	0.985975	0.0552464	0.070148	uncharacterized protein
XP_009689693.1	0.0550783	0.52723	0.104467	0.0437836	0.475582	0.0920631	0.098265	uncharacterized protein
XP_009689694.1	0.0780807	0.522478	0.149443	0.0688537	0.566217	0.121603	0.135523	conserved hypothetical protein
XP_009689695.1	2.29097E-05	0.0229097	0.001				0.001	conserved hypothetical protein
XP_009689696.1	0.0744462	0.574327	0.129623	0.0936577	0.641139	0.14608	0.137851	uncharacterized protein
XP_009689697.1	0.0606694	0.544708	0.11138	0.061821	0.509663	0.121298	0.116339	uncharacterized protein
XP_009689698.1	0.0559507	0.746166	0.0749842	0.0379403	0.599439	0.063293	0.069139	exosome complex exonuclease
XP_009689699.1	0.0213276	0.356269	0.0598639	0.0119257	0.348204	0.0342491	0.047057	proteasome subunit beta type
XP_009689700.1	0.0255308	0.151375	0.16866	0.0396229	0.200099	0.198017	0.183339	uncharacterized protein
XP_009689701.1	0.0930816	0.641698	0.145055	0.0743044	0.664582	0.111806	0.128431	conserved hypothetical protein
XP_009689702.1	0.0531075	0.401605	0.132238	0.0396757	0.4038	0.0982559	0.115247	conserved hypothetical protein
XP_009689703.1	0.0502893	0.500005	0.100578	0.0555359	0.587285	0.0945637	0.097571	conserved hypothetical protein
XP_009689704.1	0.0672611	0.35225	0.190947	0.0670996	0.364723	0.183974	0.187461	conserved hypothetical protein
XP_009689705.1	0.0995769	0.539174	0.184684	0.0446743	0.482571	0.0925756	0.13863	conserved hypothetical protein
XP_009689706.1	0.070681	0.281418	0.25116	0.0595987	0.354116	0.168303	0.209732	conserved hypothetical protein
XP_009689707.1	0.056442	0.446234	0.126485	0.0534881	0.333678	0.160299	0.143392	uncharacterized protein
XP_009689708.1	0.123819	0.560998	0.220713				0.220713	conserved hypothetical protein
XP_009689709.1	0.0546794	0.854718	0.0639736	0.0583938	0.778571	0.0750012	0.069487	uncharacterized protein
XP_009689710.1	0.0727685	0.464311	0.156723	0.072818	0.481242	0.151313	0.154018	uncharacterized protein
XP_009689711.1	0.0454925	0.514517	0.0884179	0.0423548	0.376858	0.112389	0.100403	P-type ATPase
XP_009689712.1	0.0724157	0.450484	0.160751	0.0686489	0.324829	0.211339	0.186045	DEAD-box family RNA helicase
XP_009689713.1	0.0633775	0.0599466	1.05723	0.043378	0.125758	0.344933	0.701081	DEAD-box family RNA helicase
XP_009689714.1	0.00495796	0.703661	0.00704596	0.00319184	1.22002	0.00261622	0.004831	60S ribosomal protein L31
XP_009689715.1	0.0306488	0.561954	0.0545398	0.0178433	0.617671	0.028888	0.041714	uncharacterized protein
XP_009689716.1	0.0523091	0.758564	0.0689581	0.0342524	0.604209	0.0566896	0.062824	actin depolymerizing factor
XP_009689718.1	0.0821012	0.00164202	50				50	hypothetical protein
XP_009689719.1	0.0273414	0.239318	0.114247	0.0143993	0.0924665	0.155724	0.134986	prefoldin subunit 3
XP_009689720.1	0.045848	0.454331	0.100913				0.100913	vesicular cargo protein
XP_009689721.1	0.0445708	0.673451	0.0661827	0.0295627	0.632117	0.0467677	0.056475	ATP synthase subunit delta
XP_009689722.1	0.132961	0.587458	0.226333	0.124271	0.25486	0.487605	0.356969	metastasis-associated protein MTA2
XP_009689723.1	0.0883938	0.59622	0.148257	0.0734579	0.611451	0.120137	0.134197	conserved hypothetical protein
XP_009689724.1	0.0899924	0.701803	0.12823	0.0854032	0.667859	0.127876	0.128053	conserved hypothetical protein
XP_009689726.1	0.0290109	1.19438	0.0242896	0.0298326	1.36703	0.0218229	0.023056	molecular chaperone protein
XP_009689727.1	0.0923062	0.414288	0.222807	0.0600481	0.315407	0.190383	0.206595	uncharacterized protein
XP_009689728.1	0.0532989	0.350526	0.152054	0.112463	0.32752	0.343378	0.247716	conserved hypothetical protein
XP_009689729.1	0.018762	0.850149	0.0220691	0.0207359	0.71915	0.0288339	0.025452	SWI/SNF family transcriptional activator protein
XP_009689730.1	0.152116	0.73434	0.207147	0.0962534	0.684555	0.140607	0.173877	conserved hypothetical protein
XP_009689731.1	0.00565917	0.79437	0.00712411	0.00579199	0.647398	0.00894657	0.008035	transitional endoplasmic reticulum ATPase
XP_009689732.1	0.0343242	0.697253	0.0492278	0.0333794	0.556444	0.059987	0.054607	uncharacterized protein
XP_009689733.1	0.0953606	0.518412	0.183948	0.0698248	0.291299	0.239702	0.211825	conserved hypothetical protein
XP_009689734.1	0.011845	0.392696	0.0301632	0.0172504	0.370441	0.0465674	0.038365	uncharacterized protein
XP_009689735.1	0.129601	0.404436	0.320448	0.0895953	0.37214	0.240757	0.280602	conserved hypothetical protein
XP_009689736.1	0.0533246	0.631865	0.0843923	0.0373474	0.510183	0.0732039	0.078798	choline/ethanolamine kinase
XP_009689737.1				0.0158726	0.16735	0.0948467	0.0948467	protein kinase
XP_009689738.1	0.106594	0.517227	0.206088				0.206088	conserved hypothetical protein
XP_009689739.1	0.0162363	0.959235	0.0169263	0.0136752	1.04128	0.0131331	0.01503	polyadenylate-binding protein
XP_009689740.1	0.0190823	0.44296	0.0430791				0.0430791	ribosome biogenesis regulatory protein
XP_009689741.1	0.0442558	0.47832	0.0925233	0.0238775	0.486901	0.0490397	0.070781	co-chaperone protein
XP_009689742.1	0.0236948	0.3265	0.0725723	0.0279476	0.332292	0.0841057	0.078339	conserved hypothetical protein
XP_009689743.1	0.015387	0.285363	0.0539209	0.00856884	0.232331	0.036882	0.045401	small ribonucleoprotein D1
XP_009689744.1	0.0279215	0.360629	0.0774243	0.0250161	0.257586	0.0971175	0.087271	mitochondrial inner membrane subunit
XP_009689745.1	0.000358311	0.358311	0.001	0.00529045	0.32447	0.0163049	0.008652	mitochondrial import inner membrane translocase Tim17 subunit

protein_id	Chitose_ka	Chitose_ks	Chitose_kaks	Buffeli_ka	Buffeli_ks	Buffeli_kaks	mean_kaks	function
XP_009689746.1	0.0874512	0.661835	0.132134	0.101312	0.979204	0.103463	0.117799	conserved hypothetical protein
XP_009689747.1	0.0523637	0.464048	0.112841	0.0535287	0.56312	0.0950573	0.103949	conserved hypothetical protein
XP_009689748.1	0.0707211	0.422514	0.167382	0.0469181	0.252539	0.185786	0.176584	conserved hypothetical protein
XP_009689749.1	0.00921551	0.402131	0.0229167	0.0116855	0.456838	0.0255791	0.024248	transketolase subunit
XP_009689750.1	0.0233024	0.607901	0.0383325	0.0187662	0.602751	0.0311343	0.034733	threonyl-tRNA synthetase
XP_009689751.1	0.0438415	0.559968	0.0782928	0.0356998	0.527915	0.0676242	0.072958	autophagy protein
XP_009689752.1	0.119189	0.535115	0.222736	0.0955483	0.53107	0.179917	0.201326	uncharacterized protein
XP_009689753.1	0.0487558	0.236942	0.205771	0.0858935	0.35717	0.240484	0.223128	uncharacterized protein
XP_009689754.1	0.052016	0.496913	0.104678	0.0330411	0.476762	0.0693031	0.086991	uncharacterized protein
XP_009689755.1	0.0589922	0.408789	0.14431	0.0587498	0.471278	0.124661	0.134485	uncharacterized protein
XP_009689756.1	0.0479067	0.319407	0.149987	0.0504794	0.381759	0.132228	0.141107	uncharacterized protein
XP_009689757.1	0.0134337	0.509369	0.0263732	0.0188204	0.385318	0.0488437	0.037608	conserved hypothetical protein
XP_009689758.1	0.0170417	0.409999	0.0415653	0.00317447	0.334569	0.00948824	0.025527	uncharacterized protein
XP_009689759.1	0.00176868	1.76868	0.001	0.0003411	0.3411	0.001	0.001	60S ribosomal protein L35
XP_009689760.1	0.0437653	0.917142	0.0477192	0.0404871	0.987501	0.0409995	0.044359	phosphoglycerate kinase
XP_009689761.1	0.208632	0.159149	1.31092	0.183337	0.133738	1.37087	1.340895	conserved hypothetical protein
XP_009689762.1	0.0642367	0.919163	0.069886	0.0845441	1.00506	0.0841185	0.077002	conserved hypothetical protein
XP_009689763.1	0.161269	0.0283883	5.68083	0.178701	0.0750278	2.3818	4.031315	hypothetical protein
XP_009689765.1	0.0236066	0.524517	0.0450063	0.035825	0.518993	0.069028	0.057017	7%2C8 dihydro-8-oxoguanine DNA glycosylase
XP_009689766.1	0.0201106	0.440408	0.0456635	0.0201038	0.578897	0.0347278	0.040196	conserved hypothetical protein
XP_009689767.1	0.0319715	0.524186	0.0609927	0.0353443	0.373251	0.0946931	0.077843	uncharacterized protein
XP_009689768.1	0.0243646	0.277784	0.0877104	0.0219854	0.414251	0.0530726	0.070391	mitochondrial membrane protease%2C subunit 2
XP_009689769.1	0.0750876	0.521216	0.144062	0.0736498	0.437444	0.168364	0.156213	conserved hypothetical protein
XP_009689770.1	0.075686	0.662734	0.114203	0.0559137	0.65397	0.0854989	0.099851	uncharacterized protein
XP_009689771.1	0.124141	0.589871	0.210454	0.148051	0.610232	0.242615	0.226534	conserved hypothetical protein
XP_009689772.1	1.38327E-05	0.0138327	0.001	0.00039469	0.39469	0.001	0.001	conserved hypothetical protein
XP_009689773.1	0.109668	0.457804	0.239551	0.0681498	0.49011	0.13905	0.1893	conserved hypothetical protein
XP_009689774.1	0.0551549	0.765157	0.072083	0.0517968	0.780075	0.0663998	0.069241	DNA-directed RNA polymerase precursor
XP_009689775.1	0.0283738	0.487406	0.0582138	0.0238518	0.398816	0.0598065	0.05901	conserved hypothetical protein
XP_009689776.1	0.0234337	0.277631	0.0844057	0.00523894	0.263092	0.0199129	0.052159	uncharacterized protein
XP_009689777.1	0.0197257	0.224653	0.0878053	0.00957486	0.369915	0.0258839	0.056845	replication factor C subunit
XP_009689778.1	0.0208795	0.548076	0.038096	0.0355499	0.328219	0.108312	0.073204	conserved hypothetical protein
XP_009689779.1	0.000377484	0.377484	0.001	0.000273524	0.273524	0.001	0.001	ribosomal protein S26e
XP_009689780.1	0.0639323	0.598875	0.106754	0.0768603	0.128306	0.599038	0.352896	conserved hypothetical protein
XP_009689781.1	0.0829486	0.701841	0.118187	0.0732814	0.732515	0.100041	0.109114	conserved hypothetical protein
XP_009689782.1	0.0367674	0.594349	0.0618617	0.0445791	0.702369	0.0634697	0.062666	calmodulin-domain protein kinase
XP_009689783.1	0.0656235	0.409005	0.160447	0.0520769	0.251937	0.206706	0.183577	thrombospondin%2C type I repeat containing protein
XP_009689784.1	0.0353695	0.43017	0.082222	0.0140004	0.410281	0.034124	0.058173	uncharacterized protein
XP_009689785.1	0.158625	0.367401	0.431748	0.14784	0.338688	0.436508	0.434128	uncharacterized protein
XP_009689786.1	0.0215182	0.626242	0.0343608	0.0218968	0.577924	0.0378888	0.036125	transcription factor IIH subunit
XP_009689787.1	0.0185347	0.491582	0.0377041	0.0128576	0.63666	0.0201954	0.02895	conserved hypothetical protein
XP_009689788.1	0.00822768	0.234431	0.0350964	0.0188616	0.250477	0.0753027	0.0552	uncharacterized protein
XP_009689789.1	0.025925	0.238805	0.108561				0.108561	uncharacterized protein
XP_009689790.1	0.0279306	0.525813	0.0531189	0.0359754	0.512965	0.0701323	0.061626	phosphoribosyl pyrophosphate synthetase
XP_009689791.1	0.0829467	0.562094	0.147567	0.0769272	0.51987	0.147974	0.14777	conserved hypothetical protein
XP_009689793.1	0.0212788	0.52217	0.0407507				0.0407507	DNA repair protein
XP_009689794.1	0.0526903	0.189995	0.277324	0.0325337	0.0941277	0.345634	0.311479	conserved hypothetical protein
XP_009689795.1	0.0583529	0.565175	0.103248	0.062369	0.563992	0.110585	0.106916	conserved hypothetical protein
XP_009689796.1	0.0933955	0.405515	0.230313	0.0728389	0.495793	0.146914	0.188613	conserved hypothetical protein
XP_009689797.1	0.0840228	0.619283	0.135678	0.0683364	0.647577	0.105526	0.120602	uncharacterized protein
XP_009689798.1	0.0395383	0.331998	0.119092	0.0205228	0.241006	0.0851548	0.102123	uncharacterized protein
XP_009689799.1	0.027676	0.761239	0.0363565	0.0215899	0.572337	0.0377224	0.037039	alanyl-tRNA synthetase
XP_009689800.1	0.0268059	0.578885	0.0463061	0.0213127	0.42181	0.0505268	0.048416	uncharacterized protein

protein_id	Chitose_ka	Chitose_ks	Chitose_kaks	Buffeli_ka	Buffeli_ks	Buffeli_kaks	mean_kaks	function
XP_009689801.1	0.0818251	0.629729	0.129937	0.0542809	0.68447	0.0793036	0.10462	uncharacterized protein
XP_009689802.1	0.0233074	1.03553	0.0225076	0.0299165	0.601361	0.049748	0.036128	conserved hypothetical protein
XP_009689803.1	0.0336109	0.415003	0.0809894	0.0256085	0.378046	0.0677392	0.074364	RNA pseudouridylate synthase
XP_009689804.1	0.0609743	0.704184	0.0865886	0.0505413	0.526922	0.095918	0.091253	uncharacterized protein
XP_009689805.1	0.0267506	0.623294	0.042918	0.0252537	0.613415	0.0411691	0.042044	actin
XP_009689806.1	0.0292747	0.493571	0.0593121	0.0348164	0.419326	0.0830295	0.071171	uncharacterized protein
XP_009689807.1	0.0390083	0.447873	0.0870968				0.0870968	conserved hypothetical protein
XP_009689808.1	0.0216412	0.446487	0.0484699	0.0174686	0.425395	0.0410644	0.044767	uncharacterized protein
XP_009689809.1	0.0485675	0.34444	0.141004	0.03036	0.255394	0.118875	0.129939	conserved hypothetical protein
XP_009689810.1	0.0358032	0.528212	0.0677819	0.0356973	0.462328	0.0772122	0.072497	uncharacterized protein
XP_009689811.1	0.0580844	0.798832	0.0727116	0.0517722	0.569371	0.0909286	0.08182	uncharacterized protein
XP_009689812.1	0.0406107	0.788338	0.0515143	0.0266903	0.909572	0.0293438	0.040429	DEAD-box family helicase
XP_009689813.1	0.0182944	0.702672	0.0260354	0.0206445	0.445223	0.0463689	0.036202	conserved hypothetical protein
XP_009689814.1	0.121735	0.645432	0.18861	0.113591	0.730071	0.155589	0.1721	conserved hypothetical protein
XP_009689815.1	0.0397428	0.723938	0.0548981	0.0453564	0.522832	0.0867513	0.070825	binding protein
XP_009689817.1	0.0781862	0.538392	0.145222	0.0758763	0.523874	0.144837	0.145029	arginyl-tRNA synthetase
XP_009689818.1	0.0543824	0.727868	0.0747146	0.0352983	0.646732	0.0545794	0.064647	conserved hypothetical protein
XP_009689819.1	0.0422782	0.413411	0.102267	0.0304245	0.435603	0.0698446	0.086056	uncharacterized protein
XP_009689820.1	0.00653175	0.323823	0.0201707	0.0250294	0.447388	0.0559457	0.038058	protein kinase
XP_009689821.1	0.0630197	0.828875	0.0760303	0.0713361	0.6172	0.11558	0.095805	uncharacterized protein
XP_009689822.1	0.0605735	0.569886	0.106291	0.0523134	0.608914	0.0859126	0.096102	uncharacterized protein
XP_009689823.1	0.0425882	0.691049	0.0616283				0.0616283	nucleolar phosphoprotein
XP_009689824.1	0.0959756	0.572416	0.167667	0.08424	0.467449	0.180212	0.17394	uncharacterized protein
XP_009689825.1	0.0487288	1.02922	0.0473454	0.0392404	0.997875	0.039324	0.043335	uncharacterized protein
XP_009689826.1	0.090029	0.332507	0.270758	0.0866201	0.412567	0.209954	0.240356	uncharacterized protein
XP_009689827.1	0.0632305	0.695747	0.0908815	0.0584171	0.582579	0.100273	0.095577	conserved hypothetical protein
XP_009689828.1	0.0509429	0.309456	0.164621	0.0573766	0.265566	0.216054	0.190337	conserved hypothetical protein
XP_009689829.1	0.0991523	0.331664	0.298954	0.102799	0.300807	0.341744	0.320349	conserved hypothetical protein
XP_009689830.1	0.0108313	0.298445	0.0362924				0.0362924	ubiquitin-like protein
XP_009689831.1	0.00793191	0.421886	0.0188011	0.00455954	0.538098	0.00847343	0.013637	ATP-dependent Clp protease proteolytic subunit
XP_009689832.1	0.0744546	0.428749	0.173656	0.0641707	0.336748	0.19056	0.182108	uncharacterized protein
XP_009689833.1	0.070152	0.422432	0.166067	0.0580796	0.382165	0.151975	0.159021	uncharacterized protein
XP_009689834.1	0.0249187	0.476824	0.0522596	0.0217308	0.469915	0.0462441	0.049252	uncharacterized protein
XP_009689835.1	0.0298138	0.514528	0.0579439	0.028914	0.483023	0.0598606	0.058902	porin
XP_009689836.1	0.0825149	0.728086	0.113331	0.0584938	0.611039	0.0957285	0.10453	uncharacterized protein
XP_009689837.1	0.070317	0.828918	0.0848299	0.0757756	0.770554	0.0983392	0.091585	patatin-family phospholipase
XP_009689838.1	0.0095577	0.555059	0.0172193	0.00952447	0.663682	0.0143509	0.015785	aconitate hydratase 3%2C mitochondrial precursor
XP_009689839.1	0.00734668	0.543925	0.0135068	0.0145888	0.596388	0.0244619	0.018984	60S ribosomal protein L2/L8
XP_009689840.1	0.0400862	0.515575	0.0777504	0.0528706	0.3446	0.153426	0.115588	3' exonuclease%2C exosome component
XP_009689841.1	0.0915211	0.494396	0.185117	0.0934743	0.454904	0.205481	0.195299	hypothetical protein
XP_009689842.1	0.0294776	0.685987	0.0429711	0.0267603	0.512069	0.052259	0.047615	glutamyl-tRNA synthetase
XP_009689843.1	0.103875	0.825772	0.125791	0.0833758	0.669044	0.124619	0.125205	conserved hypothetical protein
XP_009689844.1	0.0750175	0.418392	0.1793	0.0825012	0.518684	0.159059	0.169179	conserved hypothetical protein
XP_009689845.1	0.0703296	0.429366	0.163799	0.0796767	0.620865	0.128332	0.146066	major piroplasm surface protein
XP_009689846.1	0.108592	0.719566	0.150914	0.158395	0.831783	0.190428	0.170671	conserved hypothetical protein
XP_009689847.1	0.0561586	0.437905	0.128244	0.0504342	0.503089	0.100249	0.114247	conserved hypothetical protein
XP_009689848.1	0.0251151	0.474227	0.05296	0.023682	0.537459	0.044063	0.048511	CDC5-like
XP_009689849.1	0.0415036	0.313584	0.132352	0.0352914	0.298925	0.118061	0.125206	uncharacterized protein
XP_009689850.1	0.0405808	0.434439	0.0934098	0.0303518	0.402085	0.075486	0.084448	conserved hypothetical protein
XP_009689851.1	0.0196409	0.504176	0.0389565	0.00548837	0.495913	0.0110672	0.025012	replication factor C subunit
XP_009689852.1	0.0759309	0.48901	0.155275	0.0698355	0.541288	0.129017	0.142146	uncharacterized protein
XP_009689853.1	0.0539904	0.968133	0.0557675	0.050306	0.890004	0.0565234	0.056145	conserved hypothetical protein
XP_009689854.1	0.0335749	0.994823	0.0337496	0.0301612	1.00848	0.0299077	0.031829	uncharacterized protein

protein_id	Chitose_ka	Chitose_ks	Chitose_kaks	Buffeli_ka	Buffeli_ks	Buffeli_kaks	mean_kaks	function
XP_009689855.1	0.0672259	0.334697	0.200856	0.0583203	0.314666	0.18534	0.193098	predicted protein
XP_009689856.1	0.0768384	0.629919	0.121981	0.0737342	0.793765	0.0928917	0.107436	conserved hypothetical protein
XP_009689857.1	0.0684135	0.546134	0.125269	0.0688677	0.600337	0.114715	0.119992	HR1 rho-binding repeat containing protein
XP_009689858.1	0.0653626	0.300008	0.217869	0.0430242	0.253425	0.169771	0.19382	DnaJ-like molecular chaperone
XP_009689859.1	0.0590149	0.342208	0.172453	0.0425104	0.40007	0.106258	0.139355	uncharacterized protein
XP_009689860.1	0.023864	0.689301	0.0346206	0.0382123	0.752198	0.0508008	0.042711	hexose transporter
XP_009689861.1	0.250042	0.397828	0.628517				0.628517	hypothetical protein
XP_009689862.1	0.000265789	0.265789	0.001	0.00325701	0.306467	0.0106276	0.005814	Ubc protein
XP_009689863.1	0.0481674	0.526733	0.0914456	0.0330789	0.526042	0.0628827	0.077164	transcription factor TFIIE
XP_009689864.1	0.0423388	0.427686	0.0989948	0.0353536	0.404251	0.0874547	0.093225	queuine tRNA-ribosyltransferase
XP_009689865.1	0.0146838	0.451968	0.0324884	0.016371	0.549876	0.0297721	0.03113	calmodulin-like domain protein kinase
XP_009689866.1	0.153478	0.387817	0.39575				0.39575	hydrolase
XP_009689867.1	0.0683283	0.513565	0.133047	0.0419772	0.29165	0.14393	0.138489	conserved hypothetical protein
XP_009689868.1	0.068044	0.439883	0.154686	0.0634824	0.400744	0.158411	0.156548	conserved hypothetical protein
XP_009689869.1	0.0408871	0.198796	0.205674	0.0495018	0.213171	0.232216	0.218945	uncharacterized protein
XP_009689870.1	0.0298404	0.217765	0.13703	0.134285	0.692137	0.194015	0.165523	HAD-superfamily hydrolase%2C subfamily IIB protein
XP_009689871.1	0.0567369	0.300144	0.189032	0.0413116	0.222216	0.185908	0.18747	haloacid dehalogenase-like family hydrolase
XP_009689872.1	0.0583455	0.473567	0.123204	0.0185245	0.241435	0.0767264	0.099965	nucleosome assembly protein
XP_009689873.1	0.0297682	0.314565	0.094633	0.0182817	0.326713	0.0559564	0.075295	conserved hypothetical protein
XP_009689874.1	0.0301672	0.539208	0.0559473	0.0248515	0.468776	0.0530136	0.05448	elongation factor 2
XP_009689875.1	0.0297353	0.624403	0.047622	0.0308725	0.849902	0.0363248	0.041973	step II splicing factor
XP_009689876.1	0.0442492	0.490116	0.0902833	0.0412231	0.384009	0.107349	0.098816	apoptosis regulatory protein
XP_009689877.1	0.0807938	0.642653	0.125719	0.0652862	0.565001	0.115551	0.120635	conserved hypothetical protein
XP_009689878.1	0.0144785	0.497265	0.0291162	0.0309025	0.644205	0.04797	0.038543	eukaryotic translation initiation factor 3 subunit
XP_009689879.1	0.102746	0.536616	0.191471	0.085095	0.584728	0.145529	0.1685	conserved hypothetical protein
XP_009689880.1	0.0473047	0.516554	0.0915775	0.0405554	0.558114	0.0726651	0.082121	cysteine desulfurase
XP_009689881.1	0.0527646	0.528032	0.0999269	0.048691	0.462836	0.105201	0.102564	uncharacterized protein
XP_009689882.1	0.122178	0.123482	0.989437	0.0854302	0.0761983	1.12116	1.055298	hypothetical protein
XP_009689883.1	0.0287591	0.51415	0.0559353	0.0392747	0.519981	0.075531	0.065733	translation elongation factor E1-F beta
XP_009689884.1	0.0643417	0.373038	0.17248	0.061465	0.373258	0.164672	0.168576	kinesin
XP_009689886.1	0.0136364	0.385202	0.0354007	0.0178403	0.406933	0.043841	0.039621	mitochondrial phosphate carrier protein
XP_009689887.1	0.008612	0.320394	0.0268794	0.0317567	0.559519	0.0567571	0.041818	XPA binding protein 1
XP_009689888.1	0.0929484	0.68634	0.135426	0.0663894	0.529788	0.125313	0.130369	pseudouridylate synthase
XP_009689889.1	0.0144518	0.604248	0.023917	0.0088038	0.574211	0.015332	0.019624	peptidyl-prolyl cis-trans isomerase
XP_009689890.1	0.128536	0.645865	0.199014	0.13313	0.721867	0.184424	0.191719	ADP-ribosylation factor GTPase activating protein
XP_009689891.1	0.0576077	0.855731	0.0673199	0.0572167	1.07223	0.0533621	0.060341	uncharacterized protein
XP_009689892.1	0.116416	0.380661	0.305825	0.0672455	0.398211	0.168869	0.237347	conserved hypothetical protein
XP_009689893.1	0.0413059	0.45247	0.0912898	0.030768	0.431067	0.0713764	0.081333	myosin
XP_009689894.1	0.0462916	0.813753	0.0568865	0.0401131	0.710715	0.0564404	0.056663	conserved hypothetical protein
XP_009689895.1	0.123961	0.499204	0.248317	0.121984	0.440165	0.277132	0.262725	conserved hypothetical protein
XP_009689896.1	0.0590698	0.545698	0.108246	0.0596464	0.369838	0.161277	0.134762	uncharacterized protein
XP_009689897.1	0.0957519	0.620371	0.154346	0.0869534	0.58241	0.149299	0.151822	conserved hypothetical protein
XP_009689898.1	0.110934	0.464101	0.23903	0.112523	0.458007	0.24568	0.242355	conserved hypothetical protein
XP_009689899.1	0.135294	0.417577	0.323998	0.115622	0.343491	0.33661	0.330304	hypothetical protein
XP_009689900.1	0.0306192	0.581088	0.0526928	0.0192331	0.661102	0.0290925	0.040893	chaperonin
XP_009689901.1	0.108947	0.404948	0.269039	0.105584	0.378487	0.278964	0.274002	conserved hypothetical protein
XP_009689902.1	0.156468	0.412611	0.379215	0.107639	0.343514	0.313347	0.346281	conserved hypothetical protein
XP_009689906.1	0.13624	0.488211	0.27906	0.0937758	0.414268	0.226365	0.252713	conserved hypothetical protein
XP_009689907.1	0.0795119	0.427457	0.186011	0.060992	0.336531	0.181237	0.183624	conserved hypothetical protein
XP_009689908.1	0.0468367	0.53599	0.0873835	0.0452551	0.516085	0.0876893	0.087536	uncharacterized protein
XP_009689909.1	0.0883812	0.368376	0.239921	0.064595	0.418585	0.154317	0.197119	conserved hypothetical protein
XP_009689910.1	0.0199224	0.442936	0.0449782	0.00440596	0.391439	0.0112558	0.028117	chloroplast ribosomal protein L1/50S ribosomal protein L10
XP_009689911.1	0.0205683	0.545424	0.0377106	0.0233011	0.429201	0.0542895	0.046	uncharacterized protein

protein_id	Chitose_ka	Chitose_ks	Chitose_kaks	Buffeli_ka	Buffeli_ks	Buffeli_kaks	mean_kaks	function
XP_009689912.1	0.0657503	0.809342	0.0812392	0.0558981	0.624464	0.0895137	0.085376	vacuolar sorting protein
XP_009689913.1	0.0337591	0.44524	0.0758222	0.0215884	0.474298	0.0455165	0.060669	metalloprotease/cell division cycle protein
XP_009689914.1	0.0335981	0.531203	0.0632491	0.0273618	0.4994	0.0547893	0.059019	conserved hypothetical protein
XP_009689915.1	0.0388905	0.577934	0.0672922	0.037163	0.516108	0.0720062	0.069649	DNA topoisomerase 2
XP_009689916.1	0.0646107	0.309207	0.208956	0.0587272	0.284172	0.206661	0.207809	hypothetical protein
XP_009689917.1	0.0935417	0.59853	0.156286	0.0713827	0.511045	0.13968	0.147983	conserved hypothetical protein
XP_009689918.1	0.0207703	0.417477	0.0497521	0.0205066	0.548879	0.037361	0.043557	putative ribosomal protein L23
XP_009689919.1	0.0821033	0.31961	0.256886	0.107947	0.424151	0.254501	0.255694	conserved hypothetical protein
XP_009689920.1	0.0527669	0.61172	0.0862599	0.0566984	0.701258	0.0808525	0.083556	phosphatidylglycerophosphate synthase
XP_009689922.1	0.0263781	0.476817	0.0553213	0.0251768	0.50889	0.049474	0.052398	DEAD-box family helicase
XP_009689923.1	0.0493244	0.602388	0.0818815	0.0657686	0.538511	0.12213	0.102006	orotidine 5'-phosphate decarboxylase
XP_009689924.1	0.0938891	0.446621	0.210221	0.0875127	0.387353	0.225925	0.218073	conserved hypothetical protein
XP_009689925.1	0.124577	0.591037	0.210776	0.115322	0.633428	0.18206	0.196418	conserved hypothetical protein
XP_009689926.1	0.0303138	0.452954	0.0669247	0.0341662	0.385725	0.0885766	0.077751	ubiquitin-fusion degradation pathway component
XP_009689927.1	0.0598841	0.700596	0.0854759	0.0476559	0.555398	0.0858049	0.08564	uncharacterized protein
XP_009689928.1	0.0626737	0.435102	0.144044	0.0836288	0.388997	0.214985	0.179515	ubiquitin-related chaperonin
XP_009689929.1	0.0265997	0.282255	0.09424	0.040462	0.195697	0.206759	0.1505	uncharacterized protein
XP_009689930.1	0.0953613	0.391689	0.243462	0.0863447	0.342845	0.251848	0.247655	conserved hypothetical protein
XP_009689931.1	0.00271125	0.605856	0.00447507	0.00551769	0.482098	0.0114452	0.00796	protein tyrosine phosphatase
XP_009689932.1	0.0568635	0.536988	0.105893	0.0508692	0.455177	0.111757	0.108825	acylphosphatase
XP_009689933.1	0.0583179	0.463596	0.125794	0.0508725	0.534408	0.0951942	0.110494	conserved hypothetical protein
XP_009689935.1	0.0840448	0.601404	0.139748	0.084497	0.553172	0.15275	0.146249	inositol phosphatase
XP_009689936.1	0.0582102	0.536667	0.108466	0.0842068	0.502902	0.167442	0.137954	conserved hypothetical protein
XP_009689937.1	0.0552393	0.349952	0.157848	0.0659394	0.280289	0.235255	0.196551	conserved hypothetical protein
XP_009689938.1	0.034182	0.196106	0.174304				0.174304	conserved hypothetical protein
XP_009689939.1	0.0458402	0.446539	0.102657	0.0522837	0.351417	0.14878	0.125719	uncharacterized protein
XP_009689940.1	0.0547669	0.724553	0.0755872	0.057212	0.693946	0.0824445	0.079016	uncharacterized protein
XP_009689941.1	0.0355076	0.540606	0.0656811	0.0189734	0.465314	0.0407755	0.053228	aspartyl aminopeptidase
XP_009689942.1	0.0825363	0.436275	0.189184	0.0986897	0.47298	0.208655	0.198919	uncharacterized protein
XP_009689943.1	0.0375535	0.384063	0.0977795	0.0373271	0.4526	0.0824725	0.090126	predicted protein
XP_009689944.1	0.0681135	0.442916	0.153784	0.00854416	0.383381	0.0222864	0.088035	conserved hypothetical protein
XP_009689945.1	0.0347237	0.330107	0.105189	0.0233238	0.238561	0.0977687	0.101479	uncharacterized protein
XP_009689946.1	0.00910119	0.176053	0.0516957				0.0516957	Fe/Mn superoxide dismutase
XP_009689947.1	0.0160909	0.612953	0.0262514	0.0257216	0.475977	0.0540396	0.040146	uncharacterized protein
XP_009689948.1	0.030258	0.644788	0.0469271	0.0145306	0.509784	0.0285034	0.037715	ATPase
XP_009689949.1	0.124101	0.429705	0.288804	0.0924641	0.406847	0.22727	0.258037	conserved hypothetical protein
XP_009689950.1	0.0702105	0.458343	0.153183	0.0555083	0.486976	0.113986	0.133584	uncharacterized protein
XP_009689951.1	0.0645662	0.407716	0.158361	0.0483955	0.427391	0.113235	0.135798	conserved hypothetical protein
XP_009689952.1	0.0879849	0.434989	0.202269	0.0869284	0.381091	0.228104	0.215187	uncharacterized protein
XP_009689953.1	0.0362256	0.677504	0.0534692	0.0373166	0.800744	0.0466024	0.050036	major facilitator superfamily MFS-1 protein
XP_009689954.1	0.1312	0.567666	0.231121	0.0736319	0.639725	0.115099	0.17311	major facilitator superfamily MFS-1 protein
XP_009689955.1	0.059042	0.40413	0.146097	0.0475731	0.323397	0.147104	0.146601	conserved hypothetical protein
XP_009689956.1	0.0566005	0.466228	0.121401	0.0586879	0.419191	0.140003	0.130702	ATP-dependent RNA helicase
XP_009689957.1	0.00437232	0.572463	0.00763773	0.011979	0.563146	0.0212717	0.014455	transcription or splicing factor-like protein
XP_009689958.1	0.0525783	0.49301	0.106648	0.0626362	0.486202	0.128828	0.117738	eukaryotic initiation factor-2 alpha kinase-A
XP_009689959.1	0.0479537	0.347206	0.138113	0.0688828	0.348139	0.19786	0.167987	U1 snRNP-specific protein C
XP_009689960.1	0.000029819	0.029819	0.001	0.00634346	0.013222	0.479767	0.240383	conserved hypothetical protein
XP_009689961.1	0.0486012	0.251784	0.193027				0.193027	conserved hypothetical protein
XP_009689962.1	0.0898877	0.274196	0.327823				0.327823	conserved hypothetical protein
XP_009689963.1	0.0435837	0.526178	0.0828306	0.0519353	0.703776	0.0737953	0.078313	pre-mRNA splicing protein
XP_009689964.1	0.0196879	0.48632	0.0404836	0.00630234	0.36329	0.017348	0.028916	L-lactate dehydrogenase
XP_009689965.1	0.0976311	0.521026	0.187382	0.0701016	0.602166	0.116416	0.151899	uncharacterized protein
XP_009689966.1	0.0562155	0.521207	0.107856	0.0553509	0.412773	0.134095	0.120975	uncharacterized protein

protein_id	Chitose_ka	Chitose_ks	Chitose_kaks	Buffeli_ka	Buffeli_ks	Buffeli_kaks	mean_kaks	function
XP_009689967.1	0.187764	0.424843	0.441961	0.165018	0.394225	0.418587	0.430274	conserved hypothetical protein
XP_009689968.1	0.0423385	0.411723	0.102833	0.0353021	0.398457	0.0885971	0.095715	uncharacterized protein
XP_009689969.1	0.0540315	0.38583	0.140039	0.0486532	0.269724	0.180381	0.16021	uncharacterized protein
XP_009689970.1	0.0453298	1.00153	0.0452604	0.0424886	0.932067	0.0455853	0.045423	uncharacterized protein
XP_009689971.1	0.0444655	0.563558	0.0789014	0.0371955	0.454803	0.0817836	0.080342	ribonuclease P subunit
XP_009689972.1	0.0250731	0.755577	0.0331841	0.0208999	0.659867	0.0316729	0.032428	homeodomain-like containing protein
XP_009689973.1	0.0723636	0.889469	0.0813559	0.0698586	0.749801	0.0931695	0.087263	ethanolamine phosphotransferase
XP_009689974.1	0.0545138	0.33945	0.160594	0.0673824	0.317877	0.211977	0.186285	conserved hypothetical protein
XP_009689975.1	0.0271713	0.498892	0.0544632	0.0257714	0.48292	0.0533659	0.053915	uncharacterized protein
XP_009689976.1	0.0587919	0.405167	0.145105	0.0480542	0.364682	0.13177	0.138437	uncharacterized protein
XP_009689977.1	0.0669413	0.47012	0.142392	0.0496448	0.509835	0.0973742	0.119883	conserved hypothetical protein
XP_009689978.1	0.0422732	0.389933	0.108411	0.0237481	0.316174	0.0751107	0.091761	uncharacterized protein
XP_009689979.1	0.0288779	0.441531	0.065404	0.0293976	0.470774	0.0624453	0.063925	lipoate-protein ligase
XP_009689980.1	0.0172171	0.423002	0.0407021	0.0155247	0.391346	0.0396701	0.040186	uncharacterized protein
XP_009689981.1				0.0274922	0.180914	0.151963	0.151963	uncharacterized protein
XP_009689982.1	0.0453058	0.448928	0.10092	0.0163893	0.338859	0.0483661	0.074643	vesicle transport protein
XP_009689983.1	0.0505515	0.374027	0.135155	0.0689737	0.350261	0.196921	0.166038	ribonucleoside reductase
XP_009689984.1	0.0945115	0.784748	0.120436	0.0918609	0.455868	0.201508	0.160972	hypothetical protein
XP_009689985.1	0.0847672	0.344351	0.246165	0.107281	0.545637	0.196616	0.22139	conserved hypothetical protein
XP_009689986.1	0.111592	0.424979	0.262583	0.100495	0.25844	0.388854	0.325719	conserved hypothetical protein
XP_009689987.1	0.0761837	0.730523	0.104287	0.0571054	0.901137	0.0633704	0.083829	uncharacterized protein
XP_009689988.1	0.0608845	0.410933	0.148162	0.0428816	0.523704	0.0818815	0.115022	uncharacterized protein
XP_009689989.1	0.0180324	0.434827	0.0414704	0.0141365	0.36298	0.0389456	0.040208	RNA-binding protein
XP_009689990.1	0.0176188	0.73652	0.0239217	0.0202749	0.859302	0.0235947	0.023758	cell-cycle-control protein
XP_009689991.1	0.00413023	0.295446	0.0139797	0.00380028	0.261597	0.0145272	0.014253	30S ribosomal protein S8
XP_009689992.1	0.00378546	0.475118	0.00796741	0.000426645	0.426645	0.001	0.004484	small GTPase
XP_009689993.1	0.0271998	0.7356	0.0369764	0.0315716	0.687569	0.0459177	0.041447	uncharacterized protein
XP_009689994.1	0.0543925	0.601857	0.0903745	0.0531998	0.693404	0.0767226	0.083549	uncharacterized protein
XP_009689995.1	0.0276079	0.236131	0.116918	0.0197885	0.149602	0.132274	0.124596	conserved hypothetical protein
XP_009689996.1	0.0480842	0.28915	0.166295	0.030903	0.306947	0.100679	0.133487	conserved hypothetical protein
XP_009689997.1	0.0441027	0.271944	0.162176	0.01802	0.0703694	0.256077	0.209126	uncharacterized protein
XP_009689998.1	0.036615	0.175668	0.208434	0.081427	0.165372	0.492386	0.35041	conserved hypothetical protein
XP_009689999.1	0.0798201	0.473358	0.168625	0.0488425	0.439488	0.111135	0.13988	uncharacterized protein
XP_009690000.1	0.0508223	0.472239	0.10762	0.0548901	0.537932	0.102039	0.104829	uncharacterized protein
XP_009690001.1	0.0529201	0.327571	0.161553	0.0866689	0.221176	0.391855	0.276704	uncharacterized protein
XP_009690002.1	0.0534516	0.214084	0.249677				0.249677	conserved hypothetical protein
XP_009690003.1	0.0155137	0.158791	0.0976986	0.0208906	0.214747	0.0972803	0.097489	cyclin
XP_009690004.1	0.160489	0.474358	0.33833	0.133701	0.482499	0.2771	0.307715	conserved hypothetical protein
XP_009690005.1	0.0755209	0.529736	0.142563	0.0651565	0.35564	0.183209	0.162886	conserved hypothetical protein
XP_009690006.1	0.00432907	0.34422	0.0125764	0.00223794	0.377951	0.00592124	0.009249	phosphatidylserine decarboxylase
XP_009690010.1	0.0512524	0.0932756	0.549473				0.549473	hypothetical protein
XP_009690011.1	0.0996441	0.37253	0.26748				0.26748	uncharacterized protein
XP_009690012.1	0.162073	0.713199	0.227248	0.144068	0.505266	0.285133	0.25619	conserved hypothetical protein
XP_009690013.1	0.126308	0.428777	0.294577	0.0880972	0.546652	0.161158	0.227868	hypothetical protein
XP_009690014.1				0.0719506	0.215101	0.334497	0.334497	conserved hypothetical protein
XP_009690015.1				0.00701388	0.000140278	49.9999	49.9999	hypothetical protein
XP_009690016.1	0.109385	0.875729	0.124907	0.10392	0.702399	0.14795	0.136429	conserved hypothetical protein
XP_009690017.1	NA	NA	NA				NA	hypothetical protein
XP_009690018.1	0.0888881	0.161533	0.550279				0.550279	uncharacterized protein
XP_009690034.1	0.0482727	1.22988	0.0392498				0.0392498	uncharacterized protein
XP_009690035.1	0.0634935	1.01658	0.0624576	0.0679762	0.845832	0.0803662	0.071412	conserved hypothetical protein
XP_009690036.1	0.0965506	0.582998	0.165611				0.165611	conserved hypothetical protein
XP_009690037.1	0.0850913	0.444497	0.191433	0.0804234	0.489803	0.164196	0.177814	conserved hypothetical protein

protein_id	Chitose_ka	Chitose_ks	Chitose_kaks	Buffeli_ka	Buffeli_ks	Buffeli_kaks	mean_kaks	function
XP_009690038.1	0.0897916	0.400526	0.224184	0.101253	0.317593	0.318815	0.2715	uncharacterized protein
XP_009690040.1				0.134247	0.406921	0.329911	0.329911	conserved hypothetical protein
XP_009690041.1	0.0809992	0.736565	0.109969	0.0576036	0.72334	0.0796356	0.094802	uncharacterized protein
XP_009690042.1	0.134603	0.488578	0.2755	0.169237	0.531018	0.318702	0.297101	conserved hypothetical protein
XP_009690043.1	0.109632	0.504421	0.217342	0.116943	0.489229	0.239034	0.228188	conserved hypothetical protein
XP_009690044.1	0.0628845	0.4616	0.136232	0.0568874	0.419717	0.135537	0.135884	conserved hypothetical protein
XP_009690045.1	0.0267851	0.46928	0.0570771	0.0295686	0.428735	0.0689671	0.063022	uncharacterized protein
XP_009690046.1	0.0861106	0.518823	0.165973	0.0669047	0.58875	0.113639	0.139806	conserved hypothetical protein
XP_009690047.1	0.0295338	0.679124	0.043488	0.0287451	0.669785	0.0429169	0.043202	ubiquitin-transferase
XP_009690048.1	0.0838267	0.371174	0.225842	0.0696445	0.386867	0.180022	0.202932	conserved hypothetical protein
XP_009690049.1	0.0300552	0.343387	0.0875256	0.0351299	0.334428	0.105045	0.096285	conserved hypothetical protein
XP_009690050.1	0.0772528	0.514921	0.150028	0.0715759	0.470302	0.152191	0.15111	conserved hypothetical protein
XP_009690051.1	0.048239	0.608391	0.0792895	0.0440445	0.602417	0.073113	0.076201	conserved hypothetical protein
XP_009690052.1	0.00865039	0.425597	0.0203253	0.00975735	0.544989	0.0179037	0.019114	vacuolar ATP synthase
XP_009690053.1	0.0285976	0.447319	0.0639311	0.0318086	0.292209	0.108856	0.086394	thioredoxin
XP_009690054.1	0.000255716	0.255716	0.001	6.98577E-05	0.0698577	0.001	0.001	protein yippee-like 1
XP_009690055.1	0.0042328	0.553425	0.00764838	0.00530114	0.453391	0.0116922	0.00967	histone deacetylase
XP_009690056.1	0.0409916	0.230438	0.177885	0.0344782	0.216275	0.159418	0.168652	uncharacterized protein
XP_009690057.1	0.0216624	0.36803	0.0588604	0.0270476	0.503172	0.0537542	0.056307	conserved hypothetical protein
XP_009690058.1	0.00614225	0.470946	0.0130424	0.00173194	0.518926	0.00333755	0.00819	serine/threonine protein phosphatase pp-x isozyme 1
XP_009690059.1	0.0645259	0.688387	0.0937348	0.0558008	0.539585	0.103414	0.098574	histone
XP_009690060.1	0.0020794	0.457536	0.00454478	0.000315409	0.315409	0.001	0.002772	cytochrome Coxidase subunit
XP_009690061.1	0.0416559	0.603201	0.069058	0.0197766	0.472555	0.0418505	0.055454	aspartate aminotransferase%2C cytoplasmic
XP_009690062.1	0.0309541	0.408817	0.0757163	0.0232383	0.44339	0.0524105	0.064063	uncharacterized protein
XP_009690063.1	0.0354813	0.413187	0.0858721	0.0338619	0.4051	0.0835889	0.084731	putative peptidyl-prolyl cis-trans isomerase taCyP
XP_009690065.1	0.0625335	0.688814	0.0907843	0.0604065	0.650604	0.0928467	0.091815	conserved hypothetical protein
XP_009690066.1	0.0940897	0.717074	0.131213	0.0987847	0.751186	0.131505	0.131359	uncharacterized protein
XP_009690067.1	0.0103749	0.740972	0.0140017	0.00684652	0.829782	0.00825098	0.011126	actin II
XP_009690068.1	0.0393837	0.561435	0.0701483	0.0283394	0.566625	0.0500144	0.060081	deoxyhypusine synthase
XP_009690069.1	0.121421	0.492367	0.246607	0.107489	0.537496	0.19998	0.223293	uncharacterized protein
XP_009690070.1	0.0253893	0.567273	0.0447567	0.0286128	0.608345	0.0470338	0.045895	glycylpeptide N-tetradecanoyltransferase 1
XP_009690071.1	0.067995	0.459859	0.147861	0.0746307	0.498316	0.149766	0.148813	uncharacterized protein
XP_009690072.1	0.0381523	0.53371	0.071485	0.0376878	0.557271	0.0676292	0.069557	inorganic pyrophosphatase
XP_009690073.1	0.0405128	0.963623	0.0420422	0.0502132	0.945421	0.053112	0.047577	conserved hypothetical protein
XP_009690074.1	0.0279725	0.60661	0.0461128	0.0205973	0.6543	0.0314799	0.038796	uncharacterized protein
XP_009690075.1	0.00383409	0.549928	0.00697199	0.000536467	0.536467	0.001	0.003986	40S ribosomal protein S15A
XP_009690076.1	0.12991	0.392558	0.330933	0.0386979	0.119602	0.323557	0.327245	hypothetical protein
XP_009690077.1	0.0475791	0.707562	0.0672438	0.0490467	0.707114	0.0693618	0.068303	uncharacterized protein
XP_009690078.1	0.0344904	0.484207	0.0712307	0.0340033	0.495068	0.068684	0.069957	uncharacterized protein
XP_009690079.1	0.0490037	0.719204	0.068136	0.0392624	0.524599	0.0748428	0.071489	uncharacterized protein
XP_009690080.1	0.0539782	0.622893	0.0866573	0.0504954	0.581165	0.0868865	0.086772	uncharacterized protein
XP_009690081.1	0.0562487	0.179927	0.31262	0.0582562	0.263293	0.22126	0.26694	conserved hypothetical protein
XP_009690082.1	0.0467436	0.345255	0.135389	0.0575444	0.353954	0.162576	0.148983	conserved hypothetical protein
XP_009690083.1	0.0505356	0.812348	0.0622093	0.0427692	0.70308	0.0608311	0.06152	1-deoxy-D-xylulose 5-phosphate reducto-isomerase
XP_009690084.1	0.0397568	0.353023	0.112618	0.043446	0.511296	0.0849724	0.098795	conserved hypothetical protein
XP_009690085.1	0.0154355	0.853558	0.0180837	0.0112242	0.825276	0.0136006	0.015842	uncharacterized protein
XP_009690086.1	0.035117	0.557288	0.0630141	0.0307079	0.532206	0.0576994	0.060357	homeodomain-like containing protein
XP_009690087.1	0.0658982	0.785583	0.0838844	0.0623897	0.805436	0.0774608	0.080673	uncharacterized protein
XP_009690088.1	0.235795	0.262985	0.896608				0.896608	hypothetical protein
XP_009690089.1	0.00944359	0.428596	0.0220338	0.00946178	0.565714	0.0167254	0.01938	60S ribosomal protein L27
XP_009690090.1	0.0742759	0.550697	0.134876	0.0685405	0.600116	0.114212	0.124544	conserved hypothetical protein
XP_009690091.1	0.170407	0.447944	0.380421	0.131045	0.367489	0.356595	0.368508	conserved hypothetical protein
XP_009690092.1	NA	NA	NA				NA	ribosomal protein L29

protein_id	Chitose_ka	Chitose_ks	Chitose_kaks	Buffeli_ka	Buffeli_ks	Buffeli_kaks	mean_kaks	function
XP_009690093.1	NA	NA	NA				NA	hypothetical protein
XP_009690094.1	0.0366922	0.458186	0.0800814	0.0279294	0.628335	0.0444498	0.062266	conserved hypothetical protein
XP_009690095.1	0.165643	0.0378918	4.37147	0.192424	0.00384849	50	27.185735	hypothetical protein
XP_009690096.1	0.0070464	0.641218	0.0109891	0.00761922	0.645558	0.0118025	0.011396	60S ribosomal protein L5
XP_009690097.1	0.00731988	0.597616	0.0122485	0.00554227	0.616914	0.00898386	0.010616	60S ribosomal protein L7a
XP_009690098.1	0.125806	0.0623998	2.01613				2.01613	hypothetical protein
XP_009690099.1	0.0732602	0.398842	0.183682	0.0756671	0.41315	0.183147	0.183415	conserved hypothetical protein
XP_009690100.1	0.0759423	0.391377	0.194039	0.0816151	0.50965	0.16014	0.17709	uncharacterized protein
XP_009690101.1	0.109087	0.475547	0.229393	0.105119	0.609257	0.172536	0.200964	uncharacterized protein
XP_009690102.1	0.051707	0.771672	0.0670064	0.023879	0.851729	0.0280359	0.047521	arginine N-methyltransferase
XP_009690103.1	0.00569728	0.505032	0.011281	0.00304059	0.394228	0.00771277	0.009497	60S ribosomal protein L21
XP_009690104.1	0.0348382	0.263716	0.132105	0.0299455	0.385268	0.0777263	0.104916	transcription factor
XP_009690105.1	0.0708115	0.293239	0.241481	0.0728268	0.289212	0.251811	0.246646	uncharacterized protein
XP_009690106.1	0.0647289	0.224728	0.288033	0.118055	0.482984	0.244428	0.26623	hypothetical protein
XP_009690107.1	0.0734382	0.273319	0.268691				0.268691	hypothetical protein
XP_009690108.1	0.00230225	0.225551	0.0102072				0.0102072	predicted protein
XP_009690109.1	0.0132889	0.373417	0.0355874				0.0355874	50S ribosomal protein L24
XP_009690110.1	0.0313399	0.547357	0.0572568	0.0223146	0.483532	0.0461491	0.051703	replication factor-A protein 1
XP_009690111.1	0.00837322	0.41014	0.0204155	0.0111325	0.414588	0.0268519	0.023634	molecular chaperone DnaJ
XP_009690112.1	0.0776658	0.397062	0.195601	0.0865961	0.426226	0.203169	0.199385	uncharacterized protein
XP_009690113.1	0.123172	0.369507	0.333342	0.069275	0.475168	0.14579	0.239566	uncharacterized protein
XP_009690114.1	0.0717822	0.443518	0.161847	0.0545793	0.321993	0.169505	0.165676	uncharacterized protein
XP_009690115.1	0.096702	0.430944	0.224396	0.0893262	0.531345	0.168113	0.196254	uncharacterized protein
XP_009690116.1	0.0575623	0.78231	0.0735799				0.0735799	hypothetical protein
XP_009690117.1	0.0571328	0.0173344	3.29592				3.29592	conserved hypothetical protein
XP_009690118.1	0.0538452	0.480425	0.112078	0.0432181	0.532042	0.0812307	0.096654	uncharacterized protein
XP_009690119.1	0.0406853	0.404108	0.100679	0.0114439	0.138159	0.0828315	0.091755	uncharacterized protein
XP_009690120.1	0.0357447	0.27322	0.130828	0.0768954	0.33739	0.227913	0.17937	conserved hypothetical protein
XP_009690121.1	0.0427043	0.400909	0.106519	0.044233	0.376954	0.117343	0.111931	integral membrane protein
XP_009690122.1	0.0455564	0.434422	0.104867	0.0217535	0.34703	0.0626848	0.083776	U6 snRNA-associated sm-like protein Lsm3
XP_009690123.1	0.0387065	0.705409	0.054871	0.030993	0.586587	0.0528362	0.053854	long-chain acyl-CoA synthetase
XP_009690124.1	0.12349	0.4835	0.255408	0.157358	0.713268	0.220615	0.238012	conserved hypothetical protein
XP_009690125.1	0.0836353	0.625017	0.133813	0.0735515	0.514579	0.142935	0.138374	conserved hypothetical protein
XP_009690126.1	0.0416928	0.425434	0.0980005	0.0322553	0.336754	0.095783	0.096892	ubiquitin carboxyl-terminal hydrolase
XP_009690127.1	0.104656	0.401562	0.260622	0.100227	0.385784	0.259802	0.260212	conserved hypothetical protein
XP_009690128.1	0.0252188	0.866055	0.0291192	0.0216242	0.891588	0.0242536	0.026686	replication licensing factor
XP_009690129.1	0.0191792	0.300369	0.0638524	0.0120062	0.28746	0.0417666	0.05281	conserved hypothetical protein
XP_009690130.1	0.071655	0.624657	0.114711	0.0663297	0.614788	0.10789	0.1113	conserved hypothetical protein
XP_009690131.1	0.0783622	0.698715	0.112152	0.0414984	0.821836	0.0504948	0.081323	hypothetical protein
XP_009690132.1	0.0396923	0.584105	0.0679541	0.0339952	0.530397	0.0640938	0.066024	uncharacterized protein
XP_009690133.1	0.0402193	0.490946	0.081922	0.018408	0.392135	0.046943	0.064433	conserved hypothetical protein
XP_009690134.1	NA	NA	NA				NA	hypothetical protein
XP_009690135.1	0.0391468	0.869039	0.045046	0.0369683	0.790729	0.0467522	0.045899	uncharacterized protein
XP_009690137.1	NA	NA	NA				NA	hypothetical protein
XP_009690138.1	0.0241738	0.950964	0.0254203	0.0240915	0.7633	0.0315623	0.028491	uncharacterized protein
XP_009690139.1	0.00023778	0.23778	0.001	6.64969E-05	0.0664969	0.001	0.001	uncharacterized protein
XP_009690140.1	0.137987	0.576664	0.239284	0.107942	0.616027	0.175223	0.207253	uncharacterized protein
XP_009690141.1	0.000364734	0.364734	0.001	0.000587428	0.587428	0.001	0.001	eukaryotic translation initiation factor 4a
XP_009690142.1	0.0306045	0.368869	0.0829685	0.0276641	0.30629	0.0903199	0.086644	methionine-tRNA ligase
XP_009690143.1	0.0599476	0.527213	0.113706	0.0676887	0.614917	0.110078	0.111892	conserved hypothetical protein
XP_009690144.1	0.00484328	0.626539	0.00773021	0.010323	0.808553	0.0127672	0.010249	uncharacterized protein
XP_009690145.1	0.0847888	0.482735	0.175643	0.0634047	0.381342	0.166267	0.170955	conserved hypothetical protein
XP_009690146.1	0.00497566	0.208703	0.0238409	0.00532897	0.226872	0.0234889	0.023665	conserved hypothetical protein

protein_id	Chitose_ka	Chitose_ks	Chitose_kaks	Buffeli_ka	Buffeli_ks	Buffeli_kaks	mean_kaks	function
XP_009690147.1	0.0463923	0.537074	0.0863798	0.0670377	0.47498	0.141138	0.113759	DNA-damage inducible protein
XP_009690148.1	0.045684	0.567166	0.0805479	0.0352009	0.517486	0.0680229	0.074285	conserved hypothetical protein
XP_009690149.1	0.0585452	0.564047	0.103795	0.0496065	0.575226	0.0862384	0.095017	conserved hypothetical protein
XP_009690150.1	0.0312727	0.394099	0.0793526	0.0274263	0.362815	0.075593	0.077473	conserved hypothetical protein
XP_009690151.1	0.098369	0.303437	0.324183	0.0920544	0.236154	0.389807	0.356995	conserved hypothetical protein
XP_009690152.1	0.0187905	0.572583	0.0328171	0.0194749	0.509642	0.0382128	0.035515	pyruvate kinase
XP_009690153.1	0.074422	0.522874	0.142332	0.06789	0.484879	0.140014	0.141173	conserved hypothetical protein
XP_009690154.1	0.0424426	0.481017	0.0882351	0.0330219	0.378595	0.0872224	0.087729	uncharacterized protein
XP_009690155.1	0.0879038	0.812031	0.108252	0.0933357	0.718693	0.129869	0.119061	conserved hypothetical protein
XP_009690156.1	0.0381848	0.571674	0.0667947	0.0309992	0.529999	0.0584891	0.062642	protein kinase
XP_009690157.1	0.0420729	0.715009	0.0588425	0.0456862	0.825605	0.0553367	0.05709	cysteine proteinase
XP_009690158.1	0.198827	0.134824	1.47472				1.47472	hypothetical protein
XP_009690159.1	0.00473529	0.313465	0.0151063	0.00507411	0.472156	0.0107467	0.012927	putative ADP/ATP transporter
XP_009690160.1	0.0131646	0.237886	0.0553402				0.0553402	ubiquitin
XP_009690161.1	0.0092364	0.483274	0.0191121	0.00722892	0.35363	0.020442	0.019777	uncharacterized protein
XP_009690162.1	0.0076509	0.656734	0.0116499	0.00608263	0.582251	0.0104467	0.011048	DNA repair helicase
XP_009690163.1	0.10302	0.35095	0.293546	0.088829	0.305799	0.290482	0.292014	conserved hypothetical protein
XP_009690164.1	0.0546577	0.5585	0.0978652	0.0619532	0.68206	0.0908324	0.094349	histone acethyltransferase-like protein
XP_009690166.1	0.00716564	0.960167	0.00746291	0.00475753	0.86306	0.0055124	0.006488	heat shock protein
XP_009690168.1	0.0865734	1.07679	0.0803994	0.0683362	1.77684	0.0384594	0.059429	possible RING-finger-like protein
XP_009690169.1	0.085846	0.484742	0.177096	0.0655239	0.457243	0.143302	0.160199	conserved hypothetical protein
XP_009690170.1	0.0759751	0.493639	0.153908	0.074583	0.500693	0.14896	0.151434	conserved hypothetical protein
XP_009690171.1	0.0499655	0.35179	0.142032	0.0384769	0.315264	0.122047	0.13204	conserved hypothetical protein
XP_009690172.1	NA	NA	NA				NA	conserved hypothetical protein
XP_009690173.1	0.0753434	0.435903	0.172844	0.06072	0.507359	0.119679	0.146261	DNA repair protein Rad50
XP_009690174.1	0.0073805	1.16511	0.00633458	0.00759874	1.07871	0.00704428	0.006689	DNA repair protein Rad50
XP_009690175.1	0.118887	0.336159	0.353663	0.107796	0.521952	0.206525	0.280094	uncharacterized protein
XP_009690176.1	0.00422133	0.11255	0.0375063				0.0375063	ubiquitin carrier protein
XP_009690177.1	0.0406917	0.833563	0.0488166	0.0399299	0.918286	0.0434831	0.04615	coatomer complex subunit alpha
XP_009690178.1	0.00750398	0.446324	0.0168129	0.0786095	0.378271	0.207813	0.112313	conserved hypothetical protein
XP_009690180.1	0.0379108	0.360975	0.105023	0.0346734	0.394738	0.087839	0.096431	conserved hypothetical protein
XP_009690181.1	0.0984254	0.384452	0.256015	0.0880077	0.306756	0.286898	0.271456	uncharacterized protein
XP_009690182.1	0.0516793	0.89468	0.0577628	0.0489752	0.950459	0.0515279	0.054645	5'-3' exoribonuclease 2
XP_009690183.1	0.0470011	0.313236	0.15005	0.0401685	0.406009	0.0989352	0.124493	uncharacterized protein
XP_009690184.1	0.0626992	0.494876	0.126697	0.0543971	0.459625	0.118351	0.122524	phenylalanyl-tRNA synthetase
XP_009690185.1	0.0415629	0.645814	0.0643574	0.0264721	0.593065	0.044636	0.054497	uncharacterized protein
XP_009690186.1	0.0560794	0.378552	0.148142	0.0618761	0.423013	0.146275	0.147208	dihydroorotate dehydrogenase%2C mitochondrial precursor
XP_009690187.1	0.0206302	0.520623	0.039626	0.0232961	0.547141	0.0425779	0.041102	N-ethylmaleimide-sensitive factor
XP_009690188.1	0.0876095	0.505863	0.173188	0.0711127	0.48459	0.146748	0.159968	conserved hypothetical protein
XP_009690189.1	0.0205159	0.656583	0.0312464	0.0223666	0.705339	0.0317104	0.031478	uncharacterized protein
XP_009690190.1	NA	NA	NA				NA	hypothetical protein
XP_009690191.1	0.0384682	0.893435	0.0430565	0.038027	0.965539	0.0393842	0.04122	protein kinase
XP_009690192.1	0.123345	0.5422	0.22749	0.143936	0.669024	0.215144	0.221317	uncharacterized protein
XP_009690193.1	NA	NA	NA				NA	uncharacterized protein
XP_009690194.1	0.0268759	0.726999	0.0369683	0.0210439	0.655768	0.0320905	0.034529	translation initiation factor IF-2
XP_009690195.1	0.0788827	0.437876	0.180148	0.0738551	0.375488	0.196691	0.18842	uncharacterized protein
XP_009690196.1	0.0623498	0.810582	0.0769198	0.0529614	0.865872	0.0611654	0.069043	uncharacterized protein
XP_009690197.1	0.043678	0.276102	0.158195				0.158195	uncharacterized protein
XP_009690198.1	0.164467	0.554647	0.296525	0.170051	0.600435	0.283212	0.289868	conserved hypothetical protein
XP_009690199.1	0.0580986	0.33194	0.175028	0.0700032	0.448981	0.155916	0.165472	conserved hypothetical protein
XP_009690200.1	0.052928	0.702323	0.0753613	0.0637218	0.708888	0.0898897	0.082626	uncharacterized protein
XP_009690201.1	0.0523407	0.326206	0.160453	0.0726931	0.504927	0.143968	0.152211	50S ribosomal protein L24
XP_009690202.1	0.0905062	0.570322	0.158693	0.0823795	0.508756	0.161923	0.160308	conserved hypothetical protein

protein_id	Chitose_ka	Chitose_ks	Chitose_kaks	Buffeli_ka	Buffeli_ks	Buffeli_kaks	mean_kaks	function
XP_009690203.1	0.0554365	0.459901	0.12054	0.0457183	0.593233	0.0770664	0.098803	cyclin-like protein
XP_009690204.1	0.034801	0.698752	0.0498045	0.0315303	0.62962	0.0500782	0.049941	serine/threonine protein kinase
XP_009690205.1	0.0336565	0.934675	0.0360087	0.00838173	0.87936	0.00953162	0.02277	uncharacterized protein
XP_009690206.1	0.0625563	0.738885	0.084663	0.0654204	0.837492	0.0781147	0.081389	erythrocyte membrane-associated malaria antigen-like
XP_009690207.1	0.118208	0.664355	0.177929	0.173038	0.659483	0.262384	0.220157	conserved hypothetical protein
XP_009690208.1	0.032972	0.280464	0.117562	0.0273782	0.232243	0.117886	0.117724	glutaredoxin-like protein grla
XP_009690209.1	0.0511856	0.695424	0.0736034	0.0408425	0.629794	0.0648506	0.069227	3-demethylubiquinone-9 3-methyltransferase-like protein
XP_009690210.1	0.129763	0.465677	0.278655	0.13835	0.565264	0.244753	0.261704	conserved hypothetical protein
XP_009690211.1	0.0528966	0.76419	0.0692191	0.0539627	0.576525	0.0935999	0.081409	DNA topoisomerase I
XP_009690212.1	0.0674533	0.904657	0.0745623	0.0674394	0.929815	0.07253	0.073546	uncharacterized protein
XP_009690213.1	0.0517531	0.880427	0.0587818	0.0582085	0.724189	0.0803775	0.06958	1-phosphatidylinositol-4-phosphate 5-kinase
XP_009690215.1	0.00678494	0.2841	0.0238822	0.00232521	0.34023	0.00683424	0.015358	proteasome (prosome%2C macropain) 26S subunit%2C non-ATPase%2C 14
XP_009690216.1	0.0408495	0.391166	0.10443	0.0503532	0.364232	0.138245	0.121338	conserved hypothetical protein
XP_009690217.1	0.0229342	0.879801	0.0260675	0.0183182	0.959489	0.0190917	0.02258	transcription initiation factor TFIIb
XP_009690218.1	0.0759885	0.420307	0.180793	0.0943589	0.414206	0.227807	0.2043	conserved hypothetical protein
XP_009690219.1	0.00653605	0.332195	0.0196754	0.00408109	0.425977	0.00958056	0.014628	uncharacterized protein
XP_009690221.1	0.0229299	0.474789	0.048295	0.016492	0.633925	0.0260157	0.037155	DNA-directed RNA polymerase 2 subunit
XP_009690222.1	0.0288939	0.284	0.101739	0.0264879	0.32284	0.0820465	0.091893	uncharacterized protein
XP_009690223.1	0.0141084	0.152369	0.0925936				0.0925936	conserved hypothetical protein
XP_009690224.1	0.0596032	0.295786	0.201508				0.201508	conserved hypothetical protein
XP_009690225.1	0.0756555	0.394471	0.19179	0.0388568	0.208989	0.185927	0.188858	uncharacterized protein
XP_009690226.1	0.0284242	0.32923	0.0863355	0.0489124	0.30473	0.160511	0.123423	conserved hypothetical protein
XP_009690229.1	0.0183342	0.569714	0.0321814	0.000250007	0.250007	0.001	0.016591	maternal g10 transcript
XP_009690230.1	0.0229621	0.565375	0.0406139	0.0194368	0.553593	0.0351103	0.037862	mitochondrial processing peptidase subunit alpha
XP_009690231.1	0.11525	0.459452	0.250841	0.0995151	0.426916	0.233102	0.241972	conserved hypothetical protein
XP_009690232.1	0.00691579	0.662475	0.0104393	0.000346763	0.346763	0.001	0.00572	conserved hypothetical protein
XP_009690234.1	0.0326762	0.71814	0.0455012	0.0232335	0.679417	0.0341962	0.039849	molecular chaperone DnaJ
XP_009690235.1	0.0216467	0.0855161	0.25313	0.13835	0.110843	1.24816	0.750645	ribosomal protein S29
XP_009690236.1	0.0275546	0.598069	0.0460726	0.0301487	0.705834	0.0427136	0.044393	transporter protein cg10
XP_009690237.1	0.032743	0.299923	0.109171	0.0343598	0.389016	0.088325	0.098748	conserved hypothetical protein
XP_009690238.1	0.116113	0.711181	0.163268	0.0555725	0.411644	0.135001	0.149135	conserved hypothetical protein
XP_009690239.1	0.0399536	0.377653	0.105794	0.0432778	0.452953	0.0955457	0.10067	uncharacterized protein
XP_009690240.1	0.0215331	0.462851	0.0465227	0.0190943	0.431605	0.0442401	0.045381	uncharacterized protein
XP_009690241.1	0.0479294	0.283227	0.169226	0.0378712	0.315943	0.119867	0.144546	uncharacterized protein
XP_009690242.1	0.0974286	0.621736	0.156704	0.102883	0.654381	0.157222	0.156963	uncharacterized protein
XP_009690243.1				0.310417	0.324617	0.956255	0.956255	hypothetical protein
XP_009690244.1	0.0461044	0.740567	0.0622555	0.0368289	0.660949	0.0557212	0.058988	conserved hypothetical protein
XP_009690245.1	0.0906333	0.651684	0.139076	0.0794908	0.711413	0.111736	0.125406	uncharacterized protein
XP_009690247.1	0.00375235	0.0542955	0.0691098	0.045575	0.099367	0.458653	0.263881	uncharacterized protein
XP_009690248.1	0.0159261	0.105854	0.150454	0.000357058	0.357058	0.001	0.075727	60S ribosomal protein L37
XP_009690249.1	0.0720335	0.432222	0.166659	0.0702579	0.273467	0.256916	0.211787	conserved hypothetical protein
XP_009690251.1	0.052729	0.476449	0.110671	0.0628407	0.350013	0.179538	0.145104	predicted protein
XP_009690252.1	0.101468	0.386217	0.262722	0.121093	0.386682	0.313158	0.28794	conserved hypothetical protein
XP_009690253.1	0.0600519	0.183819	0.32669	0.0414532	0.372603	0.111253	0.218971	conserved hypothetical protein
XP_009690254.1	0.0367925	0.230846	0.159381	0.00790841	0.140262	0.0563832	0.107882	conserved hypothetical protein
XP_009690255.1	0.0324641	0.404017	0.0803532	0.0245978	0.451144	0.0545232	0.067438	protein kinase
XP_009690256.1	0.0157263	0.378491	0.0415498	0.014919	0.625129	0.0238655	0.032708	uncharacterized protein
XP_009690257.1	0.0573118	0.44951	0.127499	0.0560822	0.590891	0.0949112	0.111205	heat shock protein
XP_009690258.1	0.00547826	1.17355	0.00466811	0.00437012	1.23877	0.0035278	0.004098	heat shock protein 90
XP_009690259.1	0.00271635	0.184724	0.0147049				0.0147049	ADP-ribosylation factor
XP_009690260.1	0.0781943	0.266594	0.293309	0.0659449	0.32134	0.205218	0.249263	Conserved hypothetical protein
XP_009690261.1	0.0326921	0.584816	0.0559015	0.0251286	0.564447	0.044519	0.05021	conserved hypothetical protein
XP_009690262.1	0.0229461	0.568583	0.0403566	0.0219944	0.656564	0.0334993	0.036928	uncharacterized protein

protein_id	Chitose_ka	Chitose_ks	Chitose_kaks	Buffeli_ka	Buffeli_ks	Buffeli_kaks	mean_kaks	function
XP_009690263.1	0.0688294	0.387176	0.177773	0.0690998	0.417281	0.165595	0.171684	conserved hypothetical protein
XP_009690264.1	0.0954922	0.553223	0.172611	0.079547	0.506748	0.156975	0.164793	conserved hypothetical protein
XP_009690265.1	0.0303467	0.797242	0.0380646	0.0327717	0.519921	0.0630321	0.050548	uncharacterized protein
XP_009690266.1	0.000118861	0.118861	0.001	0.000169546	0.169546	0.001	0.001	conserved hypothetical protein
XP_009690267.1	0.0310253	0.357998	0.0866634	0.0114743	0.43385	0.0264475	0.056555	ATP synthase delta chain%2C mitochondrial
XP_009690268.1	0.0408332	0.640177	0.0637842	0.0243283	0.447954	0.0543098	0.059047	conserved hypothetical protein
XP_009690269.1	0.108717	0.485142	0.224094	0.12867	0.516264	0.249232	0.236663	hypothetical protein
XP_009690270.1	NA	NA	NA	0.0481114	0.429317	0.112065	NA	hypothetical protein
XP_009690272.1	0.0776059	0.977309	0.0794078	0.0610528	0.892544	0.0684031	0.073905	uncharacterized protein
XP_009690273.1	0.0556458	0.46701	0.119153	0.0526081	0.384566	0.136799	0.127976	ion transport protein
XP_009690274.1	0.0327165	0.581237	0.0562877	0.0357509	0.686855	0.0520501	0.054169	falcilysin
XP_009690275.1	0.0672524	0.852571	0.0788819	0.0706219	0.884501	0.0798438	0.079363	uncharacterized protein
XP_009690276.1	0.00717187	0.877049	0.00817727	0.00480165	0.971456	0.00494273	0.00656	conserved hypothetical protein
XP_009690277.1	0.0579469	0.638962	0.0906891	0.0678499	0.725071	0.093577	0.092133	conserved hypothetical protein
XP_009690278.1	0.0507481	0.703708	0.0721153	0.0571157	0.586242	0.0974267	0.084771	conserved hypothetical protein
XP_009690279.1	0.0943497	0.361745	0.260818	0.115715	0.29321	0.39465	0.327734	conserved hypothetical protein
XP_009690280.1	0.0508515	0.486164	0.104597	0.0283428	0.419495	0.0675641	0.086081	anthranilate synthase component II
XP_009690281.1	0.0518836	0.834947	0.06214	0.0433612	0.66685	0.0650239	0.063582	uncharacterized protein
XP_009690282.1	0.101863	0.625015	0.162977	0.0583538	0.470278	0.124084	0.143531	50S ribosomal protein L9
XP_009690283.1	0.0829219	0.351649	0.235808	0.0526841	0.413918	0.127282	0.181545	conserved hypothetical protein
XP_009690284.1	0.0395064	0.519405	0.0760608	0.0260118	0.447355	0.0581457	0.067103	uncharacterized protein
XP_009690285.1	0.0510363	1.0219	0.0499425	0.0564914	0.900728	0.0627174	0.05633	conserved hypothetical protein
XP_009690286.1	0.108504	0.349302	0.310631	0.121661	0.446431	0.272519	0.291575	conserved hypothetical protein
XP_009690287.1	0.0569301	0.529945	0.107426	0.0435612	0.463551	0.0939729	0.100699	uncharacterized protein
XP_009690288.1	0.0627419	0.213828	0.293422	0.0583318	0.291216	0.200304	0.246863	uncharacterized protein
XP_009690289.1	0.0254529	0.327739	0.0776619	0.0258052	0.388542	0.0664155	0.072039	hypothetical protein
XP_009690290.1	NA	NA	NA				NA	ribosomal protein L39
XP_009690292.1	0.13465	0.482012	0.27935	0.118829	0.58974	0.201494	0.240422	conserved hypothetical protein
XP_009690293.1				0.013389	0.000267781	50	50	hypothetical protein
XP_009690295.1	0.00645373	0.38586	0.0167256	0.0127674	0.348622	0.0366226	0.026674	RNA binding motif protein 42
XP_009690296.1	0.0286193	0.113211	0.252796	NA	NA	NA	NA	conserved hypothetical protein
XP_009690297.1	0.119445	0.581407	0.205441	0.116949	0.539015	0.216968	0.211205	conserved hypothetical protein
XP_009690298.1	0.0554263	0.846587	0.0654703	0.0441503	0.8029	0.0549886	0.060229	uncharacterized protein
XP_009690299.1	0.0752565	0.729042	0.103227	0.0675832	0.542758	0.124518	0.113873	RNA helicase
XP_009690300.1	0.0395127	0.517746	0.0763169	0.0386955	0.547181	0.0707179	0.073517	uncharacterized protein
XP_009690301.1	0.0186932	0.360913	0.0517942	0.00825672	0.352398	0.0234301	0.037612	ubiquitin carrier protein
XP_009690302.1	0.0175319	0.511704	0.0342617	0.0147459	0.626373	0.0235416	0.028902	T-complex protein 1 epsilon subunit
XP_009690303.1	0.0176278	0.522211	0.0337561				0.0337561	vesicle trafficking protein-like 1
XP_009690304.1	0.00434026	0.193475	0.0224332				0.0224332	conserved hypothetical protein
XP_009690305.1	0.0357358	0.52401	0.0681969	0.0319919	0.447103	0.0715537	0.069875	ATP-dependent helicase
XP_009690306.1	0.0636392	0.800124	0.0795367	0.0600804	0.596123	0.100785	0.090161	major facilitator superfamily MFS-1 protein
XP_009690307.1	0.0515965	0.506339	0.101901	0.0478574	0.394833	0.121209	0.111555	conserved hypothetical protein
XP_009690308.1	0.0362727	0.518089	0.0700125	0.0487561	0.592669	0.0822653	0.076139	conserved hypothetical protein
XP_009690309.1	0.0182441	0.529591	0.0344495	0.0121737	0.722942	0.0168391	0.025644	uncharacterized protein
XP_009690310.1	0.0041428	0.201305	0.0205797	0.000120648	0.120648	0.001	0.01079	40S ribosomal protein S11
XP_009690311.1	0.0645641	0.340487	0.189623	0.0481034	0.36747	0.130904	0.160264	conserved hypothetical protein
XP_009690312.1	0.0250329	0.395861	0.0632367	0.0458267	0.37064	0.123642	0.093439	proteasome regulatory protein
XP_009690313.1	0.219758	0.00651658	33.7229				33.7229	hypothetical protein
XP_009690314.1	0.0448826	0.702356	0.0639029	0.0483269	0.508215	0.0950915	0.079497	conserved hypothetical protein
XP_009690316.1	0.0345576	0.379263	0.0911178	0.0470029	0.477716	0.0983909	0.094754	Cg8 protein
XP_009690317.1	0.0527978	0.624403	0.0845573	0.0485072	0.766211	0.0633079	0.073933	conserved hypothetical protein
XP_009690318.1	0.0604981	0.545051	0.110995	0.055749	0.673709	0.0827494	0.096872	eukaryotic translation initiation factor 3 subunit 2
XP_009690319.1	0.0240677	0.816467	0.0294778	0.0179901	0.988526	0.0181989	0.023838	beta adaptin

protein_id	Chitose_ka	Chitose_ks	Chitose_kaks	Buffeli_ka	Buffeli_ks	Buffeli_kaks	mean_kaks	function
XP_009690320.1	0.000411431	0.411431	0.001				0.001	60S ribosomal protein L30
XP_009690321.1	0.172058	0.510339	0.337144	0.150165	0.475378	0.315885	0.326515	conserved hypothetical protein
XP_009690322.1	0.0219957	0.722615	0.030439	0.0154916	0.606669	0.0255355	0.027987	eukaryotic translation initiation factor 3
XP_009690323.1	0.0138338	0.197412	0.070076				0.070076	conserved hypothetical protein
XP_009690324.1	0.0236646	0.606083	0.0390452	0.0148931	0.807041	0.018454	0.02875	isoleucyl-tRNA synthetase
XP_009690325.1	0.0688323	0.36926	0.186406	0.0752032	0.400597	0.187728	0.187067	chaperonin
XP_009690326.1	0.0476196	0.606863	0.0784685	0.0388799	0.528154	0.0736147	0.076042	topoisomerase-related nucleotidyltransferase
XP_009690327.1	0.0383404	0.648688	0.0591045	0.0370203	0.677571	0.0546367	0.056871	endoplasmic reticulum transport protein
XP_009690328.1	0.051868	0.695144	0.0746148	0.0530445	0.712206	0.0744792	0.074547	conserved hypothetical protein
XP_009690329.1	0.0508903	0.421887	0.120625	0.0354233	0.298795	0.118554	0.11959	conserved hypothetical protein
XP_009690330.1	0.0907174	0.412171	0.220096	0.0442531	0.489726	0.0903629	0.155229	uncharacterized protein
XP_009690331.1	0.0813392	0.455337	0.178635	0.0682795	0.41993	0.162597	0.170616	helicase
XP_009690332.1	0.017587	0.378924	0.0464132	0.00934934	0.232272	0.0402518	0.043332	60S ribosomal protein L27a
XP_009690334.1	0.00422824	0.012226	0.345839	0.0217028	0.512903	0.0423136	0.194076	thymidylate kinase
XP_009690335.1	0.0659274	0.450728	0.146269	0.078284	0.446457	0.175345	0.160807	cyclin-dependent protein kinase PHO85 homologue
XP_009690336.1	0.152465	0.723913	0.210612	0.0965673	0.672256	0.143647	0.177129	conserved hypothetical protein
XP_009690337.1	0.0308292	0.515221	0.0598368	0.0329341	0.480183	0.0685867	0.064212	glutaminyl-tRNA synthetase
XP_009690338.1	0.00025384	0.25384	0.001	0.000283928	0.283928	0.001	0.001	ribosomal protein L23
XP_009690339.1	0.0112741	0.346966	0.0324934	0.00904616	0.426535	0.0212085	0.026851	syntaxin 5
XP_009690340.1	0.00978273	0.0961122	0.101784				0.101784	predicted protein
XP_009690341.1	0.0498841	0.464969	0.107285	0.0472672	0.448477	0.105395	0.10634	uncharacterized protein
XP_009690342.1	0.142442	0.464078	0.306935	0.191321	0.545099	0.350985	0.32896	conserved hypothetical protein
XP_009690343.1	0.121504	0.349233	0.347917	0.142789	0.421382	0.338858	0.343387	conserved hypothetical protein
XP_009690344.1				0.112518	0.412602	0.272704	0.272704	conserved hypothetical protein
XP_009690345.1	0.0517584	0.544897	0.0949874	0.0469918	0.505338	0.0929908	0.093989	Bromodomain-containing protein 1
XP_009690346.1	0.0444573	0.537712	0.0826786	0.0402538	0.546844	0.0736111	0.078145	uncharacterized protein
XP_009690347.1	0.0225458	0.686921	0.0328216	0.0324058	1.08798	0.0297852	0.031303	cargo protein
XP_009690348.1	0.113877	0.476477	0.238998	0.094381	0.427307	0.220874	0.229936	uncharacterized protein
XP_009690349.1	0.0693236	0.474259	0.146172	0.0556924	0.399719	0.139329	0.142751	ATP-dependent RNA helicase
XP_009690350.1	0.00198998	0.246198	0.00808282	0.00032908	0.32908	0.001	0.004541	predicted protein
XP_009690351.1	0.0490807	0.390754	0.125605	0.0388207	0.382028	0.101617	0.113611	apurinic/apyrimidinic endonuclease
XP_009690352.1	0.0513779	0.540646	0.0950305	0.0526381	0.576687	0.0912766	0.093154	conserved hypothetical protein
XP_009690353.1	0.133369	0.427133	0.312243				0.312243	conserved hypothetical protein
XP_009690354.1	0.0871867	0.418183	0.208489				0.208489	conserved hypothetical protein
XP_009690357.1	0.131329	0.744095	0.176496	0.170507	0.499852	0.341115	0.258806	uncharacterized protein
XP_009690358.1	0.0358378	0.280032	0.127977	0.0113647	0.130632	0.0869979	0.107487	uncharacterized protein
XP_009690359.1	0.0786011	0.353089	0.22261	0.0616008	0.330886	0.186169	0.20439	conserved hypothetical protein
XP_009690360.1	0.0804871	0.600901	0.133944	0.104021	0.770247	0.135048	0.134496	uncharacterized protein
XP_009690362.1	0.0759544	0.309154	0.245685				0.245685	uncharacterized protein
XP_009690364.1	0.113765	0.539251	0.210968	0.111031	0.429556	0.258478	0.234723	uncharacterized protein
XP_009690366.1	0.0197771	0.157235	0.12578				0.12578	conserved hypothetical protein
XP_009690367.1	0.0436591	0.373172	0.116994				0.116994	uncharacterized protein
XP_009690368.1	0.0324235	0.636344	0.0509527	0.0353162	0.687859	0.0513422	0.051147	adhesion regulation modulator protein
XP_009690369.1	0.098874	0.467459	0.211514	0.0879396	0.50045	0.175721	0.193617	uncharacterized protein
XP_009690370.1	0.0315032	0.547346	0.0575562	0.0477457	0.219405	0.217614	0.137585	conserved hypothetical protein
XP_009690371.1	NA	NA	NA				NA	uncharacterized protein
XP_009690372.1	0.0759213	0.338685	0.224165	0.081077	0.317969	0.254984	0.239574	uncharacterized protein
XP_009690373.1	0.0665971	0.561068	0.118697	0.0584012	0.408333	0.143023	0.13086	ribosomal protein S9/S16
XP_009690374.1	0.0764271	0.458483	0.166696	0.102968	0.408339	0.252162	0.209429	conserved hypothetical protein
XP_009690375.1	0.0447147	0.704857	0.0634379	0.054379	0.76726	0.0708743	0.067156	uncharacterized protein
XP_009690376.1	0.0205125	0.170377	0.120395	0.0233675	0.224202	0.104225	0.11231	serine/threonine protein phosphatase 1%2C PP1-gamma catalytic subunit
XP_009690377.1	0.0632622	1.03548	0.0610945	0.0770785	1.17853	0.0654021	0.063248	uncharacterized protein
XP_009690378.1	0.0170675	0.499235	0.0341873	0.0210655	0.364593	0.0577782	0.045983	mRNA capping enzyme

protein_id	Chitose_ka	Chitose_ks	Chitose_kaks	Buffeli_ka	Buffeli_ks	Buffeli_kaks	mean_kaks	function
XP_009690379.1	0.032167	0.3173	0.101377	0.0283373	0.339522	0.0834623	0.09242	uncharacterized protein
XP_009690380.1	0.15062	0.783122	0.192333				0.192333	uncharacterized protein
XP_009690381.1	0.144479	0.646243	0.223567	0.17904	0.710997	0.251816	0.237692	uncharacterized protein
XP_009690382.1	0.0335939	0.421061	0.0797838	0.0437479	0.513815	0.0851434	0.082464	hydrolase
XP_009690383.1	0.0141664	0.416522	0.0340112	0.00532301	0.2641	0.0201553	0.027083	eukaryotic peptide chain release factor
XP_009690384.1	3.57164E-05	0.0357164	0.001				0.001	molecular chaperone protein
XP_009690385.1	0.0539726	0.308646	0.174869				0.174869	conserved hypothetical protein
XP_009690386.1	0.0164183	0.29409	0.0558275				0.0558275	uncharacterized protein
XP_009690387.1	0.00687861	0.531748	0.0129359	0.00403704	0.541555	0.00745454	0.010195	RNA polymerase subunit RPB4-like protein
XP_009690388.1	0.118838	0.448656	0.264876	0.121905	0.390497	0.312179	0.288527	conserved hypothetical protein
XP_009690389.1	0.0341173	0.715028	0.0477146	0.0389992	0.790174	0.0493552	0.048535	uncharacterized protein
XP_009690391.1				0.0999528	0.440786	0.22676	0.22676	conserved hypothetical protein
XP_009690392.1				0.000365568	0.365568	0.001	0.001	small nuclear ribonucleoprotein associated protein b
XP_009690393.1	0.0584685	0.2401	0.243517	0.123705	0.429755	0.28785	0.265684	conserved hypothetical protein
XP_009690394.1	0.0288151	0.408903	0.0704693	0.0241041	0.419638	0.0574402	0.063955	Diphthamide synthesis protein
XP_009690395.1	0.16175	0.374964	0.431373	0.167496	0.319763	0.523815	0.477594	uncharacterized protein
XP_009690396.1	0.0774823	0.533457	0.145246	0.0912311	0.503386	0.181235	0.163241	uncharacterized protein
XP_009690397.1	0.062037	0.403697	0.153672	0.0506985	0.374237	0.135472	0.144572	splicing factor
XP_009690398.1	0.106896	0.6422	0.166453	0.10675	0.680022	0.156981	0.161717	conserved hypothetical protein
XP_009690399.1	0.0288901	0.338335	0.0853889	0.0404785	0.375817	0.107708	0.096548	50S ribosomal protein L9
XP_009690400.1	0.0540503	0.543652	0.0994207	0.0534904	0.472628	0.113176	0.106298	Mg protoporphyrin IX chelatase
XP_009690401.1	0.0712667	0.611615	0.116522	0.058528	0.536048	0.109184	0.112853	dual-specificity phosphatase
XP_009690402.1	0.064999	0.442923	0.14675	0.0455929	0.390756	0.116679	0.131715	uncharacterized protein
XP_009690403.1	0.0090224	0.533754	0.0169037	0.0107305	0.432238	0.0248254	0.020865	60S acidic ribosomal protein P2
XP_009690404.1	0.0357773	0.456995	0.0782882				0.0782882	uncharacterized protein
XP_009690405.1	0.0298489	0.565076	0.0528227	0.0186272	0.573926	0.0324557	0.042639	cleavage and polyadenylation specificity factor subunit 4
XP_009690406.1	0.0593835	0.551446	0.107687	0.0209856	0.190252	0.110304	0.108995	uracil-DNA glycosylase
XP_009690407.1	0.00370208	0.367882	0.0100632	0.00940499	0.275817	0.0340986	0.022081	splicing factor 3b subunit 4
XP_009690408.1	0.0388749	0.503675	0.0771825	0.0441476	0.684217	0.0645229	0.070853	conserved hypothetical protein
XP_009690409.1	0.0504345	0.491909	0.102528	0.042223	0.412032	0.102475	0.102501	eukaryotic translation initiation factor 3%2C subunit 6
XP_009690410.1	0.0258944	0.43128	0.0600408	0.0273532	0.407318	0.0671544	0.063598	U1/2 small nuclear ribonucleoprotein
XP_009690411.1	0.0275207	0.44072	0.0624448	0.0189814	0.435756	0.0435596	0.053002	CAMP-dependent protein kinase catalytic subunit
XP_009690412.1	0.0901718	0.729769	0.123562	0.0790455	0.629379	0.125593	0.124578	uncharacterized protein
XP_009690413.1	0.0361358	0.442367	0.0816875	0.0303475	0.555839	0.0545977	0.068143	uncharacterized protein
XP_009690414.1	0.0423448	0.394222	0.107414	0.0111053	0.117835	0.0942441	0.100829	conserved hypothetical protein
XP_009690415.1	0.0427122	0.485167	0.088036	0.0561227	0.428937	0.130841	0.109439	uncharacterized protein
XP_009690416.1	0.104285	0.6783	0.153744	0.0980819	0.692002	0.141736	0.14774	uncharacterized protein
XP_009690417.1	0.182418	0.871974	0.209201	0.205922	0.759411	0.27116	0.240181	hypothetical protein
XP_009690418.1	0.078253	0.636856	0.122874	0.0666765	0.610184	0.109273	0.116073	uncharacterized protein
XP_009690419.1	0.0937327	0.49894	0.187864	0.103508	0.505769	0.204654	0.196259	conserved hypothetical protein
XP_009690420.1	0.0366121	0.490517	0.07464	0.0329026	0.555918	0.0591861	0.066913	ABC transporter
XP_009690421.1	0.0398461	0.581521	0.0685205	0.0305264	0.529161	0.0576883	0.063104	splicing factor 3 subunit 1
XP_009690422.1	0.0743626	0.449151	0.165562	0.073803	0.40015	0.184438	0.175	ABC transporter
XP_009690423.1	0.0438037	0.568058	0.0771114	0.0488085	0.565235	0.0863507	0.081731	uncharacterized protein
XP_009690424.1	0.0289865	0.270716	0.107074	0.0464271	0.313072	0.148296	0.127685	ribosomal protein L20
XP_009690425.1	0.0343854	0.320647	0.107237	0.0334362	0.512616	0.0652266	0.086232	translocation protein
XP_009690426.1	0.0712507	0.901765	0.0790125	0.0736577	0.566389	0.130048	0.10453	uncharacterized protein
XP_009690427.1	0.047935	0.49616	0.096612	0.0510688	0.600616	0.0850274	0.09082	uncharacterized protein
XP_009690428.1	0.0212774	0.218375	0.0974352	0.0196143	0.180443	0.108701	0.103068	cytochrome c
XP_009690429.1	0.0760966	0.353977	0.214976	0.0742716	0.426331	0.174211	0.194594	conserved hypothetical protein
XP_009690430.1	0.121345	0.590879	0.205364	0.0607193	0.603809	0.10056	0.152962	uncharacterized protein
XP_009690431.1	0.051986	0.763673	0.0680736	0.047656	0.63815	0.0746784	0.071376	calcium-dependent protein kinase
XP_009690432.1	0.0673273	0.451771	0.14903				0.14903	conserved hypothetical protein

protein_id	Chitose_ka	Chitose_ks	Chitose_kaks	Buffeli_ka	Buffeli_ks	Buffeli_kaks	mean_kaks	function
XP_009690433.1	0.0647896	0.575834	0.112514	0.0537439	0.600702	0.0894686	0.100991	uncharacterized protein
XP_009690434.1	0.049241	0.680629	0.0723463	0.0469771	0.642724	0.0730907	0.072718	conserved hypothetical protein
XP_009690435.1	0.0980584	0.714085	0.13732				0.13732	hypothetical protein
XP_009690436.1	0.153083	0.666757	0.229593	0.130353	0.651271	0.200152	0.214872	uncharacterized protein
XP_009690437.1	0.0476904	0.475675	0.100258	0.0322643	0.308408	0.104616	0.102437	uncharacterized protein
XP_009690438.1	0.057054	0.604806	0.0943344	0.0461874	0.551713	0.0837162	0.089025	uncharacterized protein
XP_009690439.1	0.0778329	0.74363	0.104666	0.0702083	0.755122	0.0929762	0.098821	uncharacterized protein
XP_009690440.1	0.0882577	0.870159	0.101427	0.0549569	0.564917	0.0972832	0.099355	uncharacterized protein
XP_009690441.1	0.0518493	0.630918	0.0821807	0.0428991	0.887431	0.0483408	0.065261	uncharacterized protein
XP_009690442.1	0.0663364	0.726173	0.0913506	0.0598565	0.76879	0.0778581	0.084604	conserved hypothetical protein
XP_009690443.1	0.0258134	0.811981	0.0317906	0.0203379	0.669909	0.0303591	0.031075	uncharacterized protein
XP_009690444.1	0.0322186	0.721177	0.0446751	0.0230353	0.662844	0.0347522	0.039714	uncharacterized protein
XP_009690445.1	0.0645729	0.969633	0.0665952	0.0674292	1.01117	0.0666846	0.06664	uncharacterized protein
XP_009690446.1	0.108404	0.624541	0.173575	0.104881	0.725258	0.144612	0.159093	conserved hypothetical protein
XP_009690447.1	0.00783577	0.895584	0.00874934	0.0124542	0.744752	0.0167226	0.012736	clathrin-coat assembly protein
XP_009690448.1	0.125737	0.507558	0.247729	0.0759198	0.172518	0.440068	0.343898	conserved hypothetical protein
XP_009690449.1	0.0806781	0.900643	0.0895783	0.0734141	1.02456	0.0716543	0.080616	uncharacterized protein
XP_009690450.1	0.126921	0.954977	0.132904	0.121524	1.21986	0.099621	0.116262	conserved hypothetical protein
XP_009690451.1	0.108777	0.459507	0.236726	0.120355	0.434989	0.276686	0.256706	uncharacterized protein
XP_009690452.1	0.0205602	0.996967	0.0206228	0.0188512	0.774715	0.0243331	0.022478	nucleoside diphosphate kinase
XP_009690453.1	0.0387653	0.66056	0.0586854	0.0310035	0.624524	0.0496434	0.054164	signal recognition particle receptor subunit alpha
XP_009690454.1	0.0451102	0.705505	0.0639403	0.0470988	0.882212	0.0533871	0.058664	conserved hypothetical protein
XP_009690455.1	0.035225	0.746017	0.0472174	0.0515735	0.989583	0.0521164	0.049667	conserved hypothetical protein
XP_009690456.1	0.0672995	0.549993	0.122364	0.0618066	0.50546	0.122278	0.122321	conserved hypothetical protein
XP_009690457.1	0.0427899	0.676733	0.0632301	0.022499	1.09347	0.0205758	0.041903	uncharacterized protein
XP_009690458.1	0.0100632	0.458228	0.0219611	0.0133131	0.590163	0.0225584	0.02226	mitochondrial respiratory chain complexes assembly protein
XP_009690459.1	0.0473344	0.531188	0.0891105	0.0371219	0.603865	0.0614739	0.075292	ATP-dependent RNA helicase
XP_009690460.1	0.0801016	0.643588	0.124461	0.0946121	0.515876	0.183401	0.153931	conserved hypothetical protein
XP_009690461.1	0.0301824	0.381922	0.0790278	0.030529	0.488103	0.0625462	0.070787	uncharacterized protein
XP_009690462.1	0.0107872	0.441225	0.0244482	0.0107498	0.457486	0.0234976	0.023973	uncharacterized protein
XP_009690463.1	0.0785733	0.363008	0.21645	0.110246	0.462245	0.238501	0.227475	uncharacterized protein
XP_009690464.1	0.0402155	0.856342	0.046962	0.0373029	0.705884	0.0528456	0.049904	predicted protein
XP_009690465.1	0.0608944	0.543986	0.111941	0.0942152	0.563843	0.167095	0.139518	purine nucleoside phosphorylase
XP_009690466.1	0.0756892	0.549923	0.137636	0.0820201	0.513873	0.159612	0.148624	purine nucleoside phosphorylase
XP_009690467.1	0.0496644	0.464579	0.106902	0.061226	0.345046	0.177443	0.142172	uncharacterized protein
XP_009690468.1	0.0256994	0.531821	0.0483235	0.0268981	0.347249	0.0774605	0.062892	uncharacterized protein
XP_009690469.1	0.01968	0.289392	0.0680046	0.0431182	0.16622	0.259405	0.163705	uncharacterized protein
XP_009690470.1	0.00704175	0.319818	0.022018				0.022018	vacuolar ATP synthase subunit F
XP_009690471.1	0.0285049	0.569149	0.0500835	0.0194994	0.730511	0.0266929	0.038388	uncharacterized protein
XP_009690472.1	0.0558797	0.318376	0.175514	0.0442351	0.33707	0.131234	0.153374	conserved hypothetical protein
XP_009690473.1	0.0479386	0.46569	0.102941	0.0487928	0.472543	0.103256	0.103099	conserved hypothetical protein
XP_009690476.1	0.0203753	0.4014	0.0507607	0.0175537	0.265921	0.0660111	0.058386	uncharacterized protein
XP_009690477.1	0.0716945	0.564617	0.126979	0.0740921	0.533552	0.138866	0.132922	conserved hypothetical protein
XP_009690480.1	0.0243726	0.585788	0.0416064	0.0258811	0.702788	0.0368264	0.039216	oxoglutarate/malate translocator protein
XP_009690481.1	0.041452	0.678323	0.0611095	0.0391828	0.638307	0.0613856	0.061248	uncharacterized protein
XP_009690482.1	0.0475984	0.398061	0.119576	0.0393729	0.358566	0.109807	0.114692	conserved hypothetical protein
XP_009690483.1	0.0428442	0.4801	0.0892401	0.0423905	0.566178	0.0748714	0.082056	conserved hypothetical protein
XP_009690484.1	0.0224809	0.304182	0.0739061	0.0167715	0.314527	0.053323	0.063615	uncharacterized protein
XP_009690485.1	0.0339608	0.629762	0.0539264	0.035624	0.451996	0.078815	0.066371	ABC transporter
XP_009690486.1	0.148609	0.118204	1.25723	0.0966025	0.115384	0.837227	1.047229	hypothetical protein
XP_009690487.1	0.0396677	0.410231	0.0966961	0.0419379	0.565348	0.0741806	0.085438	uncharacterized protein
XP_009690488.1	0.0397044	0.520113	0.076338	0.0392983	0.466273	0.0842818	0.08031	uncharacterized protein
XP_009690489.1	0.00405715	0.434466	0.00933824	0.00136401	0.44045	0.00309685	0.006218	cell differentiation protein

protein_id	Chitose_ka	Chitose_ks	Chitose_kaks	Buffeli_ka	Buffeli_ks	Buffeli_kaks	mean_kaks	function
XP_009690490.1	0.1672	0.223365	0.748549	0.156049	0.268647	0.580869	0.664709	hypothetical protein
XP_009690491.1	0.226892	0.0629716	3.60308	0.198324	0.059203	3.34989	3.476485	hypothetical protein
XP_009690492.1	0.00495292	0.604492	0.00819352	0.00484238	0.665799	0.00727303	0.007733	tubulin subunit beta
XP_009690493.1	0.0565165	0.442866	0.127615	0.0511513	0.419226	0.122014	0.124814	uncharacterized protein
XP_009690494.1	0.0381414	0.756898	0.0503917	0.0382724	0.710218	0.0538883	0.05214	DNA topoisomerase 2
XP_009690495.1	0.0788951	0.379831	0.207711	0.0631872	0.339023	0.186381	0.197046	conserved hypothetical protein
XP_009690497.1	0.0307521	0.369455	0.0832366	0.0217102	0.408131	0.0531942	0.068215	DNA polymerase delta small subunit
XP_009690498.1	0.0599154	0.628343	0.0953545	0.0534888	0.555109	0.0963573	0.095856	conserved hypothetical protein
XP_009690499.1	0.0559888	0.503387	0.111224	0.0649949	0.455281	0.142758	0.126991	uncharacterized protein
XP_009690500.1	0.0548067	0.324323	0.168988	0.0546539	0.255546	0.213871	0.19143	conserved hypothetical protein
XP_009690501.1	0.22076	0.283287	0.779279				0.779279	hypothetical protein
XP_009690502.1	0.0329106	0.259552	0.126797				0.126797	diacylglycerol kinase
XP_009690503.1	0.0164147	0.620942	0.0264352	0.0210054	0.541899	0.0387626	0.032599	RNA processing protein
XP_009690504.1	0.0123955	0.516241	0.024011	0.00394949	0.262589	0.0150406	0.019526	conserved hypothetical protein
XP_009690505.1	0.029481	0.428198	0.0688489	0.0411745	0.302863	0.135951	0.1024	conserved hypothetical protein
XP_009690506.1	0.0997881	0.430161	0.231978	0.0777749	0.348356	0.223263	0.227621	conserved hypothetical protein
XP_009690507.1	0.000114663	0.114663	0.001				0.001	splicing factor 3B subunit 5-like protein
XP_009690508.1	0.0534581	0.592684	0.0901966	0.0559453	0.51977	0.107635	0.098916	conserved hypothetical protein
XP_009690509.1	NA	NA	NA				NA	conserved hypothetical protein
XP_009690510.1	0.121326	0.474057	0.255931	0.113022	0.512837	0.220386	0.238158	conserved hypothetical protein
XP_009690511.1	0.0405605	0.5847	0.0693697	0.0539893	0.491225	0.109907	0.089638	conserved hypothetical protein
XP_009690512.1	0.117726	0.510276	0.23071	0.132338	0.349236	0.378935	0.304822	conserved hypothetical protein
XP_009690513.1	0.0177932	0.348104	0.0511145	0.01346	0.332248	0.0405119	0.045813	uncharacterized protein
XP_009690514.1	0.0101265	0.420842	0.0240624	0.0121586	0.339309	0.0358333	0.029948	40S ribosomal protein S6
XP_009690516.1	0.0855224	0.390216	0.219167	0.0837648	0.288954	0.28989	0.254528	uncharacterized protein
XP_009690517.1	0.0167242	0.452713	0.036942	0.0179963	0.403475	0.0446033	0.040773	DnaJ protein
XP_009690518.1	0.0404344	1.03273	0.0391528	0.0423137	1.16407	0.0363497	0.037751	uncharacterized protein
XP_009690519.1	0.0917042	0.467935	0.195976	0.0859819	0.415128	0.207121	0.201549	uncharacterized protein
XP_009690520.1	0.0532012	0.652856	0.0814899	0.0612698	0.660798	0.0927209	0.087105	uncharacterized protein
XP_009690521.1	0.0253943	0.483722	0.0524978	0.0174603	0.664082	0.0262924	0.039395	CAMP-dependent protein kinase regulatory subunit
XP_009690522.1	0.0931137	0.330602	0.281649	0.111787	0.253444	0.441072	0.36136	conserved hypothetical protein
XP_009690523.1	0.0853762	0.299581	0.284985	0.0120283	0.101373	0.118654	0.201819	conserved hypothetical protein
XP_009690524.1	0.0749846	0.745475	0.100586	0.0670495	0.810448	0.0827315	0.091659	conserved hypothetical protein
XP_009690525.1	0.0387465	0.810729	0.0477922	0.0376519	0.891501	0.0422342	0.045013	ATP-dependent RNA helicase
XP_009690526.1	0.046132	0.613443	0.0752018	0.044734	0.670509	0.0667164	0.070959	conserved hypothetical protein
XP_009690527.1	0.0814766	0.716406	0.11373	0.0784828	0.865909	0.0906363	0.102183	uncharacterized protein
XP_009690529.1	0.0548923	0.848396	0.0647012	0.0539228	1.15753	0.0465844	0.055643	uncharacterized protein
XP_009690530.1	0.0233887	0.257804	0.0907228	0.026532	0.19616	0.135257	0.11299	conserved hypothetical protein
XP_009690531.1	0.102059	0.533477	0.191308	0.0852788	0.558972	0.152564	0.171936	uncharacterized protein
XP_009690532.1	0.0520044	0.871148	0.0596964	0.0460866	1.0499	0.0438963	0.051796	uncharacterized protein
XP_009690533.1	0.0349841	0.353866	0.0988627	0.0343697	0.425375	0.0807987	0.089831	autophagy-related peptidase
XP_009690534.1	0.0275412	0.802338	0.0343262	0.0279421	0.99857	0.0279821	0.031154	conserved hypothetical protein
XP_009690535.1	0.0115397	0.512498	0.0225165	0.0172994	0.582271	0.0297102	0.026113	centromere/microtubule binding protein
XP_009690536.1	0.0651566	0.561556	0.116029	0.0530749	0.650739	0.0815611	0.098795	DNA helicase
XP_009690537.1	0.0531092	0.399831	0.132829	0.0394232	0.492138	0.080106	0.106467	uncharacterized protein
XP_009690538.1	0.0305747	0.636241	0.0480552	0.0394836	0.882075	0.0447622	0.046409	chromatin-binding protein
XP_009690539.1	0.145069	0.530269	0.273576	0.147309	0.502494	0.293155	0.283365	uncharacterized protein
XP_009690540.1	0.076283	0.3946	0.193317	0.0749369	0.440115	0.170267	0.181792	conserved hypothetical protein
XP_009690541.1	0.0943956	0.0910686	1.03653				1.03653	hypothetical protein
XP_009690542.1	0.103741	0.0891399	1.1638				1.1638	hypothetical protein
XP_009690543.1	0.0502974	0.699389	0.0719162	0.050627	0.654034	0.0774073	0.074662	predicted protein
XP_009690544.1	0.112399	0.947691	0.118603	0.11489	0.802754	0.14312	0.130861	uncharacterized protein
XP_009690545.1	0.234623	0.542909	0.432159	0.2665	0.408885	0.651774	0.541967	hypothetical protein

protein_id	Chitose_ka	Chitose_ks	Chitose_kaks	Buffeli_ka	Buffeli_ks	Buffeli_kaks	mean_kaks	function
XP_009690546.1	0.0375736	0.462345	0.0812674	0.0457171	0.608864	0.0750859	0.078177	uncharacterized protein
XP_009690547.1	0.0371532	0.511141	0.0726867	0.0317117	0.652653	0.0485889	0.060638	ATP-dependent RNA helicase
XP_009690548.1	0.0676527	0.59547	0.113612	0.0761042	0.564055	0.134923	0.124268	conserved hypothetical protein
XP_009690549.1	0.105657	0.316669	0.333652	0.0669715	0.310227	0.215879	0.274765	conserved hypothetical protein
XP_009690550.1	0.0181672	0.265121	0.068524	0.025795	0.370417	0.0696379	0.069081	CDK-activating kinase assembly factor
XP_009690551.1	0.0308866	0.552397	0.0559138	0.0225604	0.530955	0.0424903	0.049202	uncharacterized protein
XP_009690552.1	0.0339482	0.773478	0.0438903	0.0321936	0.735936	0.0437451	0.043818	calcium-transporting ATPase
XP_009690553.1	0.0412618	0.414813	0.0994708	0.0409898	0.375272	0.109227	0.104349	N2%2CN2-dimethylguanosine tRNA methyltransferase
XP_009690554.1	0.0302266	0.179709	0.168197	0.0211134	0.238975	0.0883501	0.128274	hypothetical protein
XP_009690555.1	0.0322912	0.251787	0.128248	0.0184279	0.372055	0.04953	0.088889	ubiquitin fusion degradation protein
XP_009690556.1	0.0294368	0.717353	0.0410353	0.00619319	0.176495	0.0350898	0.038063	uncharacterized protein
XP_009690557.1	0.0112257	0.985378	0.0113923	0.00988375	1.05957	0.00932809	0.01036	DNA replication licensing factor
XP_009690558.1	0.0576898	0.383838	0.150297	0.0488129	0.48405	0.100843	0.12557	conserved hypothetical protein
XP_009690559.1	0.0561595	0.359428	0.156247	0.0555274	0.314814	0.176382	0.166315	conserved hypothetical protein
XP_009690560.1	0.167629	0.165291	1.01414	NA	NA	NA	NA	hypothetical protein
XP_009690561.1	0.0715322	0.181887	0.393278	NA	NA	NA	NA	conserved hypothetical protein
XP_009690562.1	0.0402393	0.38308	0.105041	0.049763	0.411066	0.121058	0.113049	conserved hypothetical protein
XP_009690563.1	0.0567353	0.427621	0.132676	0.0550378	0.562992	0.0977594	0.115218	5'-3' exonuclease
XP_009690564.1	0.0347274	0.848971	0.0409052	0.0247593	0.632839	0.0391241	0.040015	uncharacterized protein
XP_009690565.1	0.0236835	0.0612315	0.386786				0.386786	pre-mRNA branch site protein p14
XP_009690566.1	0.0181783	0.424081	0.0428652	0.0254081	0.413051	0.0615133	0.052189	conserved hypothetical protein
XP_009690567.1	0.0905742	0.39952	0.226708	0.0775617	0.445389	0.174144	0.200426	conserved hypothetical protein
XP_009690568.1	0.0755087	0.700227	0.107835	0.0764165	0.661824	0.115463	0.111649	isoleucyl-tRNA synthetase
XP_009690569.1	0.0831008	0.378882	0.219332	0.0753365	0.358889	0.209915	0.214623	brain protein 44-like
XP_009690570.1	0.0293636	0.331439	0.0885942	0.0451503	0.307592	0.146786	0.11769	U1 small nuclear ribonucleoprotein
XP_009690571.1	0.0518006	0.56862	0.0910988	0.050623	0.676103	0.0748747	0.082987	NADH-cytochrome b5 reductase
XP_009690572.1	0.0184057	0.890473	0.0206696	0.0135473	0.873728	0.0155052	0.018087	small GTPase
XP_009690573.1	0.0401864	0.466007	0.0862355	0.0311655	0.540326	0.0576791	0.071957	uncharacterized protein
XP_009690574.1	0.0501637	0.437345	0.114701	0.0519538	0.445931	0.116506	0.115603	lariat debranching enzyme
XP_009690575.1	0.0742618	0.643931	0.115326	0.0615944	0.539057	0.114263	0.114794	conserved hypothetical protein
XP_009690576.1	0.010126	0.883662	0.0114591	0.0256185	0.901671	0.0284123	0.019936	nucleolar protein Nop5
XP_009690577.1	0.179437	0.327765	0.547457	0.207791	0.471775	0.440444	0.49395	hypothetical protein
XP_009690578.1	0.0910163	0.373284	0.243826	0.0780802	0.502715	0.155317	0.199572	hypothetical protein
XP_009690579.1	0.0674253	0.110261	0.611508				0.611508	hypothetical protein
XP_009690580.1	0.0444493	0.414104	0.107339	0.0319409	0.314446	0.101578	0.104459	TS-Ikeda type piroplasm surface protein precursor
XP_009690581.1	0.0362581	0.541286	0.0669852	0.0238796	0.375845	0.0635359	0.065261	conserved hypothetical protein
XP_009690582.1	0.062058	0.468103	0.132573	0.0608995	0.284233	0.214259	0.173416	conserved hypothetical protein
XP_009690583.1	0.0385993	0.759498	0.050822	0.0609817	0.605947	0.100639	0.075731	conserved hypothetical protein
XP_009690584.1	0.0516286	0.4573	0.112899	0.0275828	0.348638	0.0791158	0.096007	conserved hypothetical protein
XP_009690585.1	0.0295683	0.763572	0.0387237	0.0273841	0.595375	0.0459946	0.042359	sulfate transporter
XP_009690586.1	NA	NA	NA				NA	hypothetical protein
XP_009690587.1	0.0410776	0.454899	0.0903006	0.0427023	0.558849	0.0764111	0.083356	conserved hypothetical protein
XP_009690588.1	0.107283	0.326519	0.328567	0.0916021	0.318659	0.287461	0.308014	cyclophilin-RNA interacting protein
XP_009690589.1	0.0876864	0.498581	0.175872	0.0847352	0.526728	0.160871	0.168372	conserved hypothetical protein
XP_009690590.1	0.0214078	0.328702	0.0651282	0.018548	0.341682	0.0542844	0.059706	uncharacterized protein
XP_009690591.1	0.0157956	0.461749	0.0342081	0.038526	0.365166	0.105503	0.069856	conserved hypothetical protein
XP_009690592.1	0.0280955	0.398416	0.0705179	0.0188482	0.29301	0.064326	0.067422	nucleolar protein
XP_009690593.1	0.0310069	0.378041	0.08202	0.0342429	0.382106	0.0896162	0.085818	GTPase
XP_009690594.1	0.0158408	0.251177	0.0630664	0.0124919	0.298028	0.0419154	0.052491	proteasome subunit y
XP_009690595.1	0.0479516	0.348888	0.137441	0.0348541	0.356269	0.097831	0.117636	conserved hypothetical protein
XP_009690596.1	0.0973817	0.478046	0.203708	0.0738673	0.505992	0.145985	0.174847	uncharacterized protein
XP_009690597.1	0.11512	0.581205	0.198072	0.105021	0.511232	0.205427	0.201749	uncharacterized protein
XP_009690598.1	0.0326749	0.564781	0.0578542	0.0296261	0.509523	0.0581448	0.058	uncharacterized protein

protein_id	Chitose_ka	Chitose_ks	Chitose_kaks	Buffeli_ka	Buffeli_ks	Buffeli_kaks	mean_kaks	function
XP_009690599.1	0.0679042	0.474225	0.14319	0.0541448	0.462941	0.116959	0.130075	proline synthetase associated protein
XP_009690600.1	0.0562174	0.416824	0.134871	0.0630348	0.423223	0.14894	0.141905	uncharacterized protein
XP_009690601.1	0.0414705	0.24932	0.166335	NA	NA	NA	NA	conserved hypothetical protein
XP_009690602.1	0.0167968	0.469074	0.0358085	0.01453	0.552611	0.0262933	0.031051	60S ribosomal protein L4/L1
XP_009690603.1	0.286863	0.14949	1.91894	NA	NA	NA	NA	hypothetical protein
XP_009690604.1	0.116791	1.05857	0.110329	0.116271	0.999682	0.116308	0.113318	conserved hypothetical protein
XP_009690605.1	0.035704	0.290669	0.122834	0.0336918	0.280009	0.120324	0.121579	conserved hypothetical protein
XP_009690606.1	0.00834191	0.285998	0.0291677	0.00994662	0.493117	0.0201709	0.024669	conserved hypothetical protein
XP_009690607.1	0.085097	0.203069	0.419054	0.0815824	0.349968	0.233114	0.326084	conserved hypothetical protein
XP_009690608.1	0.0521825	0.158706	0.3288	0.0873771	0.567912	0.153857	0.241329	guanylate kinase
XP_009690609.1	0.0261065	0.728874	0.0358176	0.0175975	0.642609	0.0273844	0.031601	pyrophosphate--fructose 6-phosphate 1-phosphotransferase subunit alpha
XP_009690610.1	0.0627732	1.01872	0.0616196	0.0610759	0.817875	0.0746762	0.068148	conserved hypothetical protein
XP_009690612.1	0.0387868	0.601136	0.0645224	0.0384921	0.619836	0.0621004	0.063311	peptidyl-prolyl cis-trans isomerase
XP_009690613.1	0.0241055	0.433328	0.0556287	0.016463	0.460487	0.0357512	0.04569	conserved hypothetical protein
XP_009690614.1	0.0777997	0.707346	0.109988	0.0902358	0.725471	0.124382	0.117185	conserved hypothetical protein
XP_009690615.1	0.103254	0.450346	0.229278	0.084905	0.436669	0.194438	0.211858	conserved hypothetical protein
XP_009690616.1	0.0175301	0.340105	0.0515433	0.00718003	0.251593	0.0285383	0.040041	casein kinase II subunit beta
XP_009690617.1	0.0189348	0.351655	0.0538449	0.00742481	0.376391	0.0197264	0.036786	conserved hypothetical protein
XP_009690618.1	0.0744028	0.387514	0.192	0.0673302	0.276128	0.243837	0.217919	conserved hypothetical protein
XP_009690619.1	0.0444198	0.408909	0.10863	0.026337	0.332766	0.0791455	0.093888	conserved hypothetical protein
XP_009690620.1	0.080541	0.447562	0.179955	0.0672177	0.34252	0.196244	0.1881	conserved hypothetical protein
XP_009690621.1	0.0185496	0.510274	0.0363523	0.0152223	0.463275	0.032858	0.034605	uncharacterized protein
XP_009690622.1	0.094619	0.818805	0.115557	0.0724746	0.586887	0.12349	0.119524	conserved hypothetical protein
XP_009690623.1	0.0954341	0.630683	0.151319	0.087848	0.438414	0.200377	0.175848	conserved hypothetical protein
XP_009690624.1	0.0406956	0.56677	0.0718027	0.0312817	0.479881	0.0651864	0.068495	conserved hypothetical protein
XP_009690625.1	0.0812349	0.425827	0.19077	0.0766543	0.370884	0.20668	0.198725	conserved hypothetical protein
XP_009690626.1	0.00862614	0.474768	0.0181692	0.00489482	0.461113	0.0106152	0.014392	U4/U6 snRNP-associated protein
XP_009690627.1	0.0331846	0.438085	0.0757494	2.30978E-05	0.0230978	0.001	0.038375	ubiquitin-like protein
XP_009690628.1	0.0343288	0.332748	0.103168	0.0414021	0.229139	0.180685	0.141927	peptidyl-prolyl cis-trans isomerase
XP_009690629.1	0.0747699	0.382856	0.195295	0.0465274	0.167793	0.27729	0.236292	conserved hypothetical protein
XP_009690630.1	0.0039623	0.374376	0.0105837	0.00361038	0.333296	0.0108324	0.010708	proliferating cell nuclear antigen 2
XP_009690632.1	0.0984052	0.573356	0.17163	0.0959304	0.475262	0.201847	0.186739	conserved hypothetical protein
XP_009690633.1	0.0176204	0.240804	0.073173	0.00526207	0.299626	0.0175621	0.045368	transmembrane protein 17
XP_009690635.1	0.0900534	0.414175	0.217428	0.0683804	0.362382	0.188697	0.203063	conserved hypothetical protein
XP_009690636.1	0.0773479	0.301516	0.25653	0.0563638	0.283122	0.199079	0.227804	conserved hypothetical protein
XP_009690637.1	0.0537062	0.524181	0.102457	0.0688378	0.435032	0.158236	0.130347	mitochondrial carrier protein
XP_009690638.1	0.212982	0.241762	0.880955	0.184399	0.167891	1.09832	0.989637	hypothetical protein
XP_009690639.1	0.0561482	0.362032	0.155092	0.0907155	0.301063	0.301317	0.228205	hypothetical protein
XP_009690640.1	0.00557064	0.421622	0.0132124	0.00367869	0.393851	0.00934031	0.011276	14-3-3 protein
XP_009690642.1	0.0156898	0.719924	0.0217937	0.0119252	0.362741	0.0328752	0.027334	uncharacterized protein
XP_009690643.1	0.00020526	0.20526	0.001	0.000150902	0.150902	0.001	0.001	histone H2A variant
XP_009690644.1	0.0334516	0.25717	0.130076	0.00723385	0.115433	0.062667	0.096371	uncharacterized protein
XP_009690645.1	0.00107896	0.503658	0.00214225	0.00118801	0.378589	0.00313799	0.00264	ATP-dependent RNA helicase
XP_009690646.1	0.0327962	0.309766	0.105874	0.0149636	0.0435215	0.34382	0.224847	conserved hypothetical protein
XP_009690647.1	0.0902161	0.703794	0.128185	0.0830907	0.54469	0.152547	0.140366	uncharacterized protein
XP_009690648.1	0.076532	0.75352	0.101566	0.0585742	0.52504	0.111561	0.106564	uncharacterized protein
XP_009690649.1	0.0908075	0.238453	0.380819	0.107722	0.263777	0.408383	0.394601	conserved hypothetical protein
XP_009690650.1	0.0856937	0.37632	0.227715	0.0879326	0.325955	0.269769	0.248742	thrombospondin%2C type I repeat containing protein
XP_009690651.1	0.0209587	0.228006	0.0919218				0.0919218	conserved hypothetical protein
XP_009690652.1	0.0464003	0.289816	0.160103	0.0197686	0.279932	0.0706193	0.115361	conserved hypothetical protein
XP_009690653.1	0.0104978	0.282728	0.0371303	0.0183236	0.228602	0.080155	0.058643	molecular chaperone
XP_009690654.1	0.0136149	0.459119	0.0296544	0.00862083	0.418192	0.0206145	0.025134	uncharacterized protein
XP_009690655.1	0.0922612	0.434629	0.212276	0.0849626	0.297509	0.28558	0.248928	uncharacterized protein

protein_id	Chitose_ka	Chitose_ks	Chitose_kaks	Buffeli_ka	Buffeli_ks	Buffeli_kaks	mean_kaks	function
XP_009690656.1	0.119742	0.486234	0.246264	0.0923373	0.500274	0.184573	0.215419	conserved hypothetical protein
XP_009690657.1	0.090033	1.1464	0.0785352	0.109534	0.779837	0.140457	0.109496	uncharacterized protein
XP_009690658.1	0.0450494	0.646003	0.0697355	0.0403796	0.634837	0.0636063	0.066671	serine/threonine protein kinase
XP_009690659.1	0.0248383	0.364318	0.0681773				0.0681773	ribosomal RNA assembly protein
XP_009690660.1	0.0258292	0.616461	0.0418991	0.0180928	0.367279	0.0492616	0.04558	uncharacterized protein
XP_009690661.1	0.0340478	0.701077	0.048565	0.0337055	0.824308	0.0408895	0.044727	50S ribosomal protein L22
XP_009690662.1	0.0532251	0.451474	0.117892	0.0426937	0.503138	0.0848549	0.101373	sodium/hydrogen exchanger
XP_009690663.1	0.0232811	0.468629	0.0496792	0.0202053	0.408175	0.0495016	0.04959	splicing factor 3a subunit 2
XP_009690664.1	0.0565292	0.673064	0.0839879	0.0677276	0.77891	0.0869517	0.08547	molecular chaperone protein DnaJ
XP_009690665.1	0.0467427	0.382731	0.122129	0.0461095	0.357557	0.128957	0.125543	GDP-mannose pyrophosphorylase
XP_009690666.1	0.0144563	0.435509	0.0331941	0.0123438	0.652256	0.0189248	0.026059	ubiquitin-conjugating enzyme E2
XP_009690667.1	0.0888522	0.460003	0.193156	0.0599372	0.153309	0.390956	0.292056	uncharacterized protein
XP_009690668.1	0.0318838	0.458623	0.0695207	0.0374362	0.329138	0.11374	0.09163	clathrin-coat assembly protein
XP_009690669.1	0.072368	0.33452	0.216334	0.0540719	0.402153	0.134456	0.175395	conserved hypothetical protein
XP_009690670.1	0.0183487	0.525422	0.0349218	0.0183803	0.408364	0.0450097	0.039966	Conserved hypothetical protein
XP_009690671.1	0.0784874	0.54591	0.143774	0.0561123	0.679419	0.0825886	0.113181	Conserved hypothetical protein
XP_009690672.1	0.0778893	0.281674	0.276522	0.0935156	0.240728	0.388471	0.332496	uncharacterized protein
XP_009690673.1	0.0720103	0.497824	0.14465	0.0683449	0.484147	0.141166	0.142908	conserved hypothetical protein
XP_009690674.1	0.0385631	0.750433	0.0513879	0.036224	0.773353	0.0468402	0.049114	uncharacterized protein
XP_009690675.1	0.0356463	0.425815	0.083713	0.0283378	0.482822	0.0586919	0.071202	hydrolase
XP_009690676.1	0.0531799	0.112312	0.4735				0.4735	conserved hypothetical protein
XP_009690677.1	0.0397392	0.281547	0.141146	0.0424393	0.295689	0.143527	0.142336	conserved hypothetical protein
XP_009690685.1	NA	NA	NA				NA	hypothetical protein
XP_009690686.1	0.0888813	1.37961	0.0644247	0.101348	1.21289	0.0835595	0.073992	choline/ethanolamine kinase
XP_009690687.1	0.0826842	0.392716	0.210545				0.210545	choline/ethanolamine kinase
XP_009690689.1	0.0433542	0.492295	0.0880655	0.0351502	0.298274	0.117846	0.102956	cleavage stimulation factor subunit 1
XP_009690690.1	0.0584513	0.242099	0.241435	0.0604867	0.25782	0.234609	0.238022	transducin-like G-protein beta
XP_009690691.1	0.161517	0.360448	0.448102	0.186297	0.406937	0.457803	0.452952	hypothetical protein
XP_009690692.1	0.0715965	0.733941	0.0975507	0.0610509	0.655212	0.0931773	0.095364	miTOT_ic checkpoint protein
XP_009690693.1	0.0487852	0.46305	0.105356	0.04371	0.429063	0.101873	0.103614	serine/threonine protein kinase
XP_009690694.1	0.0239683	0.23318	0.102789	0.014015	0.135637	0.103328	0.103058	phosphopanTOT_henoylcysteine decarboxylase
XP_009690695.1	0.0390828	0.382407	0.102202	0.0197864	0.51322	0.0385534	0.070378	uncharacterized protein
XP_009690696.1	0.0523059	0.543814	0.0961834	0.0546403	0.602243	0.0907281	0.093456	citrate synthase%2C mitochondrial precursor
XP_009690697.1	0.0166908	0.234203	0.0712665				0.0712665	1-hydroxy-2-methyl-2-(e)-butenyl 4-diphosphate synthase
XP_009690698.1	0.0448626	0.366274	0.122484	0.0404977	0.230574	0.175639	0.149061	conserved hypothetical protein
XP_009690699.1	0.0818509	0.522471	0.156661	0.0795179	0.486136	0.163571	0.160116	uncharacterized protein
XP_009690700.1	0.0365967	0.305289	0.119875	0.034148	0.284521	0.120019	0.119947	ABC transporter
XP_009690701.1	0.103909	0.383046	0.271269				0.271269	uncharacterized protein
XP_009690702.1	0.0827326	1.1491	0.0719977	0.100632	0.988279	0.101826	0.086912	uncharacterized protein
XP_009690703.1	0.0957007	0.520024	0.184032	0.107412	0.62317	0.172363	0.178198	CAMP-dependent protein kinase%2C beta-catalytic subunit
XP_009690704.1	0.0221594	0.304746	0.0727143	0.0170698	0.203905	0.0837145	0.078214	conserved hypothetical protein
XP_009690705.1	0.020345	0.490612	0.0414686	0.0176763	0.706846	0.0250073	0.033238	glycerol kinase
XP_009690706.1	0.0527866	0.371599	0.142053	0.0480128	0.344159	0.139508	0.140781	membrane skeletal protein IMC1
XP_009690707.1	0.109664	0.199482	0.549743	0.0877129	0.285457	0.307271	0.428507	conserved hypothetical protein
XP_009690708.1	0.0228632	0.694851	0.0329037	0.0161316	0.740401	0.0217876	0.027346	conserved hypothetical protein
XP_009690709.1	0.137254	0.412939	0.332382	0.101166	0.409575	0.247002	0.289692	conserved hypothetical protein
XP_009690710.1	0.0142834	0.430089	0.0332104	0.00620341	0.292063	0.02124	0.027225	60S ribosomal protein L17
XP_009690711.1	0.0538971	0.691396	0.077954	0.0373133	0.814617	0.0458048	0.061879	conserved hypothetical protein
XP_009690712.1	0.108195	0.550009	0.196714	0.100846	0.5401	0.186717	0.191715	conserved hypothetical protein
XP_009690713.1	0.000330863	0.330863	0.001	0.00412865	0.211149	0.0195532	0.010277	calmodulin
XP_009690714.1	0.0631813	0.617707	0.102284	0.0858031	0.517464	0.165815	0.134049	uncharacterized protein
XP_009690715.1	0.0227256	0.443368	0.0512568	0.0330772	0.529298	0.0624926	0.056875	uncharacterized protein
XP_009690716.1	0.0221086	0.453881	0.0487101	0.0184783	0.539777	0.0342332	0.041472	uncharacterized protein

protein_id	Chitose_ka	Chitose_ks	Chitose_kaks	Buffeli_ka	Buffeli_ks	Buffeli_kaks	mean_kaks	function
XP_009690718.1	0.0282589	0.330665	0.0854606	0.0242163	0.321398	0.0753467	0.080404	uncharacterized protein
XP_009690719.1	0.0366789	0.344096	0.106595	0.0262385	0.323821	0.0810276	0.093811	uncharacterized protein
XP_009690720.1	0.00797182	0.462264	0.0172452				0.0172452	Ngp
XP_009690721.1	0.049626	0.298327	0.166348				0.166348	uncharacterized protein
XP_009690722.1	0.0474017	0.537947	0.088116	0.0423493	0.611887	0.0692109	0.078663	uncharacterized protein
XP_009690723.1	0.116258	0.752494	0.154496	0.0848857	0.638721	0.1329	0.143698	conserved hypothetical protein
XP_009690724.1	0.102109	0.326045	0.313176	0.0881199	0.295346	0.298362	0.305769	uncharacterized protein
XP_009690725.1	0.090006	0.726559	0.12388	0.0692426	0.633802	0.10925	0.116565	conserved hypothetical protein
XP_009690726.1	0.0136342	0.474714	0.0287208	0.011767	0.513334	0.0229227	0.025822	conserved hypothetical protein
XP_009690727.1	0.0474412	0.317751	0.149303	0.0421143	0.34566	0.121838	0.13557	uncharacterized protein
XP_009690728.1	0.231637	0.0901191	2.57035				2.57035	hypothetical protein
XP_009690729.1	0.00649394	0.54739	0.0118634	0.00516846	0.553212	0.00934265	0.010603	protein transport protein
XP_009690730.1	0.000326733	0.326733	0.001	0.000082669	0.082669	0.001	0.001	small nuclear ribonucleoprotein
XP_009690731.1	0.0674563	0.490558	0.137509	0.070713	0.51934	0.13616	0.136834	conserved hypothetical protein
XP_009690732.1	0.041606	0.186353	0.223265				0.223265	conserved hypothetical protein
XP_009690733.1	0.0236334	0.681255	0.034691	0.0204485	0.72162	0.028337	0.031514	calcium-dependent protein kinase
XP_009690734.1	0.0357065	0.849446	0.0420351	0.0539768	0.675694	0.0798835	0.060959	conserved hypothetical protein
XP_009690735.1	0.0445029	0.396045	0.112368				0.112368	conserved hypothetical protein
XP_009690736.1	0.0477647	0.400081	0.119388	0.0407838	0.348867	0.116903	0.118146	GTPase activator
XP_009690737.1	0.0556384	0.470799	0.118179	0.0687631	0.586914	0.11716	0.11767	UMP-CMP kinase
XP_009690738.1	0.000388797	0.388797	0.001	0.000542805	0.542805	0.001	0.001	mitochondrial distribution and morphology family 35/apoptosis protein
XP_009690739.1	0.0626177	0.244164	0.256458	0.0470029	0.356743	0.131756	0.194107	hypothetical protein
XP_009690740.1	0.0238854	0.64281	0.0371578	0.0233181	0.642843	0.0362733	0.036716	ubiquitin-activating enzyme E1
XP_009690741.1	0.0325724	0.399528	0.0815272	0.0413351	0.395792	0.104437	0.092982	HEAT repeat containing protein
XP_009690742.1	0.0975541	0.517414	0.188542	0.0950151	0.355645	0.267163	0.227852	conserved hypothetical protein
XP_009690743.1	0.0219591	0.362009	0.0606588				0.0606588	TatD-like deoxyribonuclease
XP_009690744.1	0.0558975	0.459035	0.121772	0.0488616	0.437021	0.111806	0.116789	kinesin-like protein
XP_009690745.1	0.0566674	0.506848	0.111804	0.0580814	0.384368	0.151109	0.131457	uncharacterized protein
XP_009690746.1	0.0377489	0.461542	0.0817887	0.0222054	0.232124	0.0956618	0.088725	CDC6-like ATPase
XP_009690747.1	0.017856	0.19041	0.0937765				0.0937765	conserved hypothetical protein
XP_009690748.1	0.0614231	0.320479	0.19166	0.0426387	0.368169	0.115813	0.153736	4-diphosphocytidyl-2c-methyl-D-erythritol kinase
XP_009690749.1	0.0386183	0.264751	0.145866	0.0273227	0.285863	0.0955796	0.120723	50S ribosomal protein L28
XP_009690750.1	0.00953017	0.190869	0.0499305	2.98333E-05	0.0298333	0.001	0.025465	conserved hypothetical protein
XP_009690751.1	0.013098	0.165317	0.0792294	0.0238747	0.146161	0.163345	0.121287	eukaryotic translation initiation factor 2 beta
XP_009690752.1	0.00838437	0.367694	0.0228025				0.0228025	YOR3162c protein
XP_009690753.1	0.0489375	0.707719	0.0691482	0.0542424	0.606803	0.0893905	0.079269	uncharacterized protein
XP_009690754.1	0.089353	0.544097	0.164222	0.0668625	0.473998	0.141061	0.152641	uncharacterized protein
XP_009690755.1	0.0725262	0.829482	0.0874355	0.074145	0.912356	0.0812677	0.084352	major facilitator superfamily MFS-1 protein
XP_009690756.1	0.0545447	0.849969	0.0641726	0.042525	0.817148	0.0520407	0.058107	conserved hypothetical protein
XP_009690757.1	0.108868	0.737916	0.147534	0.0973097	0.724997	0.134221	0.140877	major facilitator superfamily MFS-1 protein
XP_009690758.1	0.0352784	0.420127	0.0839709	0.0162492	0.351087	0.0462825	0.065127	uncharacterized protein
XP_009690759.1	0.043887	0.447866	0.0979915	0.0339979	0.434141	0.0783107	0.088151	uncharacterized protein
XP_009690760.1	0.0429362	0.450536	0.0953004	0.0246549	0.478668	0.0515074	0.073404	vacuolar-protein sorting-associated protein
XP_009690761.1	0.00924778	0.130391	0.0709236				0.0709236	1-acylglycerol-3-phosphate O-acyltransferase
XP_009690762.1	NA	NA	NA				NA	hypothetical protein
XP_009690763.1	0.063451	0.226352	0.280321	0.0335048	0.060857	0.55055	0.415435	uncharacterized protein
XP_009690764.1	0.0844491	0.601531	0.14039	0.112293	0.646131	0.173793	0.157091	conserved hypothetical protein
XP_009690765.1	0.0723802	0.336276	0.21524	0.07685	0.350471	0.219276	0.217258	uncharacterized protein
XP_009690768.1	0.11137	0.432464	0.257525	0.122669	0.545211	0.224993	0.241259	conserved hypothetical protein
XP_009690769.1				0.0562628	0.136379	0.412547	0.412547	conserved hypothetical protein
XP_009690771.1	0.0299663	0.819459	0.0365684	0.0361016	0.622305	0.0580127	0.047291	ATP-dependent RNA helicase
XP_009690772.1	1.69661E-05	0.0169661	0.001				0.001	small nuclear ribonucleoprotein
XP_009690773.1	0.0393864	0.564983	0.0697125	0.0377249	0.675011	0.0558879	0.0628	conserved hypothetical protein

protein_id	Chitose_ka	Chitose_ks	Chitose_kaks	Buffeli_ka	Buffeli_ks	Buffeli_kaks	mean_kaks	function
XP_009690774.1	0.104057	0.632628	0.164484	0.07103	0.763408	0.0930434	0.128764	conserved hypothetical protein
XP_009690775.1	0.0392358	0.363297	0.107999				0.107999	uncharacterized protein
XP_009690776.1	0.00791607	0.413808	0.0191298	0.00291545	0.303884	0.00959395	0.014362	initiation factor 4E
XP_009690777.1	0.0992079	0.395688	0.250723	0.0795188	0.281109	0.282875	0.266799	serine/threonine-protein kinase ripk4
XP_009690778.1	0.0313671	0.552786	0.0567438	0.0263136	0.514074	0.0511863	0.053965	ABC transporter
XP_009690779.1	0.140438	0.224515	0.625514	0.160993	0.167907	0.95882	0.792167	hypothetical protein
XP_009690780.1	0.199793	0.280978	0.711063	0.0558268	0.141131	0.395568	0.553315	hypothetical protein
XP_009690781.1	0.0640693	0.427961	0.149708	0.0678894	0.452832	0.149922	0.149815	uncharacterized protein
XP_009690783.1	0.137467	0.351105	0.391526	0.11667	0.513736	0.2271	0.309313	conserved hypothetical protein
XP_009690784.1	0.143779	0.230272	0.624387				0.624387	hypothetical protein
XP_009690788.1	0.0458049	0.107293	0.426914				0.426914	conserved hypothetical protein
XP_009690789.1	0.134261	0.508749	0.263903	0.103468	0.925072	0.111849	0.187876	conserved hypothetical protein
XP_009690790.1	0.0299911	0.473169	0.0633834	0.0361138	0.540072	0.0668684	0.065126	uncharacterized protein
XP_009690791.1	0.0570488	0.293536	0.19435	0.0571366	0.232598	0.245645	0.219998	uncharacterized protein
XP_009690792.1	0.116474	0.859667	0.135487	0.115633	0.731499	0.158077	0.146782	conserved hypothetical protein
XP_009690793.1	0.0933476	0.598992	0.155841	0.0815799	0.595526	0.136988	0.146415	farnesyl-protein transferase beta subunit
XP_009690794.1	0.0894823	0.339036	0.263931	0.09055	0.353412	0.256216	0.260074	uncharacterized protein
XP_009690795.1	0.0861012	0.725855	0.11862	0.0814496	0.682214	0.11939	0.119005	conserved hypothetical protein
XP_009690796.1	0.108902	0.347086	0.313759	0.15196	1.06048	0.143294	0.228527	uncharacterized protein
XP_009690797.1	0.0992819	0.585476	0.169575	0.0945145	0.718155	0.131607	0.150591	uncharacterized protein
XP_009690798.1	0.0276771	0.650959	0.0425175	0.0216227	0.674774	0.0320443	0.037281	conserved hypothetical protein
XP_009690799.1	0.000131521	0.131521	0.001				0.001	lipid-binding protein
XP_009690800.1	0.017605	0.292714	0.0601439	0.0187088	0.478036	0.0391369	0.04964	60S ribosomal protein L18
XP_009690801.1	0.0272951	0.48986	0.0557201	0.0189879	0.481749	0.0394145	0.047567	60S ribosomal protein L18
XP_009690803.1	0.0644242	0.365235	0.176391				0.176391	conserved hypothetical protein
XP_009690804.1	0.000248256	0.248256	0.001	0.000319974	0.319974	0.001	0.001	small GTP binding protein rab6
XP_009690805.1	0.0507547	0.348036	0.145832	0.048044	0.296919	0.161808	0.15382	uncharacterized protein
XP_009690806.1	0.007543	0.0736557	0.102409				0.102409	farnesyltransferase beta subunit
XP_009690808.1	0.086251	0.609078	0.141609	0.0674263	0.585962	0.115069	0.128339	conserved hypothetical protein
XP_009690809.1	0.0821328	1.01957	0.0805563	0.0900019	1.00877	0.0892194	0.084888	uncharacterized protein
XP_009690810.1	0.128182	0.11172	1.14734	0.114433	0.14598	0.783893	0.965616	hypothetical protein
XP_009690811.1	0.0362738	0.610192	0.0594465	0.0440206	0.816431	0.0539183	0.056682	conserved hypothetical protein
XP_009690812.1	0.0666654	0.718634	0.0927668	0.0781978	0.770975	0.101427	0.097097	uncharacterized protein
XP_009690813.1	0.0951617	0.537911	0.17691	0.0748435	0.475007	0.157563	0.167237	conserved hypothetical protein
XP_009690814.1	0.0477803	0.467904	0.102116	0.0523765	0.497993	0.105175	0.103646	conserved hypothetical protein
XP_009690815.1	0.116543	0.313753	0.371448	0.0854794	0.284393	0.300568	0.336008	hypothetical protein
XP_009690816.1	0.106086	0.804954	0.131792	0.0915008	0.748042	0.12232	0.127056	conserved hypothetical protein
XP_009690817.1	0.0491763	1.09098	0.0450755	0.0319619	1.14991	0.0277952	0.036435	conserved hypothetical protein
XP_009690818.1	0.0268952	0.425238	0.0632475				0.0632475	uncharacterized protein
XP_009690820.1	0.0579756	0.596112	0.0972561	0.0625559	0.701244	0.089207	0.093232	uncharacterized protein
XP_009690821.1	0.0665372	0.490329	0.135699	0.0517405	0.394224	0.131247	0.133473	chromatin assembly protein
XP_009690822.1	0.0348843	0.382384	0.0912284	0.024862	0.329755	0.0753954	0.083312	cytochrome C-type heme lyase
XP_009690823.1	0.010749	1.66746	0.00644637	0.00801112	1.28192	0.00624932	0.006348	uncharacterized protein
XP_009690824.1	0.0128903	0.586084	0.0219939	0.0103298	0.408978	0.0252576	0.023626	phosphatidylinositol/phosphatidylcholine transfer protein
XP_009690825.1	0.0991355	0.00199362	49.7264				49.7264	hypothetical protein
XP_009690827.1	0.0948631	0.544856	0.174107	0.0675702	0.739046	0.0914289	0.132768	conserved hypothetical protein
XP_009690828.1	0.0515176	0.27412	0.187938	0.061639	0.325196	0.189544	0.188741	uncharacterized protein
XP_009690829.1	0.151441	0.0976654	1.55061				1.55061	hypothetical protein
XP_009690830.1	0.0954312	0.297131	0.321175	0.162185	0.163345	0.992898	0.657037	hypothetical protein
XP_009690831.1	0.0596218	0.529746	0.112548	0.040892	0.31748	0.128802	0.120675	conserved hypothetical protein
XP_009690832.1	0.00708831	0.779841	0.00908944	0.0116934	0.587307	0.0199103	0.0145	ribosomal protein S2
XP_009690833.1				7.27476E-05	0.0727476	0.001	0.001	conserved hypothetical protein
XP_009690834.1				NA	NA	NA	NA	conserved hypothetical protein

protein_id	Chitose_ka	Chitose_ks	Chitose_kaks	Buffeli_ka	Buffeli_ks	Buffeli_kaks	mean_kaks	function
XP_009690835.1	0.0529233	0.382279	0.138442	0.0337535	0.212523	0.158823	0.148633	uncharacterized protein
XP_009690836.1	0.101729	0.37858	0.268712				0.268712	conserved hypothetical protein
XP_009690837.1	0.10489	0.48009	0.21848	0.120807	0.455578	0.265173	0.241826	conserved hypothetical protein
XP_009690838.1	0.0646464	0.180798	0.357561				0.357561	hypothetical protein
XP_009690839.1	0.0782082	0.371237	0.210669	0.0967934	0.379255	0.25522	0.232944	conserved hypothetical protein
XP_009690840.1	0.0498296	0.55683	0.0894881	0.042363	0.636827	0.066522	0.078005	cation transporting ATPase
XP_009690841.1	0.07208	0.427655	0.168547	0.0700636	0.414572	0.169002	0.168774	uncharacterized protein
XP_009690842.1	0.095584	0.618557	0.154527	0.100741	1.00957	0.0997858	0.127156	conserved hypothetical protein
XP_009690843.1	0.0314215	0.463842	0.067742	0.0283748	0.574825	0.0493625	0.058552	26S proteasome non-ATPase regulatory subunit 12
XP_009690844.1	0.00497871	0.605368	0.00822427	0.000353332	0.353332	0.001	0.004612	translation initiation factor eIF-1A
XP_009690845.1	0.0407892	0.312389	0.130572	0.0296795	0.413071	0.0718507	0.101211	DNA-directed RNA polymerases I and III subunit
XP_009690846.1	NA	NA	NA				NA	conserved hypothetical protein
XP_009690847.1	0.0399428	0.400305	0.0997809				0.0997809	uncharacterized protein
XP_009690848.1	0.120707	0.918047	0.131482	0.119981	0.921181	0.130247	0.130864	uncharacterized protein
XP_009690849.1	0.126869	0.132077	0.960567				0.960567	hypothetical protein
XP_009690851.1	0.0734639	0.532237	0.138029	0.10009	0.591523	0.169208	0.153618	conserved hypothetical protein
XP_009690852.1	0.111242	0.447869	0.248381	0.129173	0.569469	0.226831	0.237606	conserved hypothetical protein
XP_009690853.1	0.0494147	0.359851	0.13732	0.0228456	0.298591	0.0765114	0.106916	conserved hypothetical protein
XP_009690854.1	0.0141758	0.335272	0.0422815				0.0422815	mitochondrial ribosomal protein S14 precursor
XP_009690856.1	0.0365798	0.429805	0.0851079	0.0463854	0.40689	0.114	0.099554	conserved hypothetical protein
XP_009690857.1	0.00356949	0.096527	0.0369792				0.0369792	conserved hypothetical protein
XP_009690858.1	0.0185865	0.854008	0.0217639	0.0164498	0.604506	0.0272119	0.024488	heat shock protein 70 precursor
XP_009690859.1	0.11106	0.347818	0.319304	0.112757	0.217366	0.518744	0.419024	uncharacterized protein
XP_009690860.1	0.00768375	0.386767	0.0198666	0.0011994	0.238381	0.00503143	0.012449	eukaryotic peptide chain release factor
XP_009690861.1	0.0926532	0.516593	0.179354	0.0853078	0.48358	0.176409	0.177882	conserved hypothetical protein
XP_009690862.1	0.120308	0.624972	0.192502	0.148365	0.887949	0.167087	0.179795	conserved hypothetical protein
XP_009690863.1	0.060806	0.392053	0.155096	0.0504135	0.559699	0.0900724	0.122584	pentatricopeptide repeat containing protein
XP_009690865.1	0.086624	0.793381	0.109183	0.0919662	0.746975	0.123118	0.116151	conserved hypothetical protein
XP_009690866.1	0.19558	0.71842	0.272236	0.14908	0.656315	0.227147	0.249691	conserved hypothetical protein
XP_009690868.1	0.0649477	0.21959	0.295768				0.295768	hypothetical protein
XP_009690869.1	0.143889	0.600141	0.239759				0.239759	uncharacterized protein
XP_009690870.1	0.283179	0.597578	0.473878				0.473878	hypothetical protein
XP_009690871.1	0.132424	0.824442	0.160623				0.160623	hypothetical protein
XP_009690873.1	0.0311258	0.28829	0.107967	0.0123415	0.121633	0.101466	0.104716	conserved hypothetical protein
XP_009690874.1	0.195161	0.239248	0.815728	0.186342	0.239351	0.778531	0.79713	hypothetical protein
XP_009690875.1	0.0577753	0.56579	0.102114	0.039375	0.463192	0.085008	0.093561	conserved hypothetical protein
XP_009690876.1	0.0717845	0.469966	0.152744	0.0484153	0.472491	0.102468	0.127606	conserved hypothetical protein
XP_009690877.1	0.116482	0.226645	0.513939	0.161547	0.279466	0.578054	0.545996	hypothetical protein
XP_009690878.1	0.0538053	0.521742	0.103126				0.103126	hypothetical protein
XP_009690879.1	0.0598178	0.58261	0.102672	0.0590346	0.480515	0.122857	0.112764	ubiquitin carboxyl-terminal hydrolase
XP_009690881.1	0.0955077	0.526474	0.18141	0.0805659	0.366977	0.21954	0.200475	uncharacterized protein
XP_009690882.1	0.0717986	0.185476	0.387104	0.0763537	0.248091	0.307765	0.347434	uncharacterized protein
XP_009690884.1	0.104707	0.729637	0.143506	0.106101	0.682957	0.155356	0.149431	uncharacterized protein
XP_009690885.1	0.0646737	0.468998	0.137898	0.0838034	0.501236	0.167194	0.152546	uncharacterized protein
XP_009690886.1	0.109828	0.337348	0.325562	0.13658	0.274153	0.49819	0.411876	conserved hypothetical protein
XP_009690887.1	0.114167	0.386466	0.295413	0.103763	0.338524	0.306516	0.300964	uncharacterized protein
XP_009690888.1	0.0328556	0.32678	0.100543	0.0333508	0.482346	0.069143	0.084843	conserved hypothetical protein
XP_009690889.1	0.054037	0.480266	0.112515	0.0557971	0.34952	0.159639	0.136077	asparaginyl-tRNA synthetase
XP_009690890.1	0.14206	0.463166	0.306715				0.306715	conserved hypothetical protein
XP_009690891.1	0.148195	0.997102	0.148625				0.148625	hypothetical protein
XP_009690892.1	0.0917326	0.476611	0.192468	0.066354	0.330424	0.200815	0.196641	ankyrin repeat containing protein
XP_009690893.1				0.0341882	0.628836	0.0543673	0.0543673	uncharacterized protein
XP_009690894.1	0.0718798	0.896203	0.0802048	0.0113425	0.279111	0.0406378	0.060421	uncharacterized protein

protein_id	Chitose_ka	Chitose_ks	Chitose_kaks	Buffeli_ka	Buffeli_ks	Buffeli_kaks	mean_kaks	function
XP_009690895.1				0.0809926	0.195768	0.413718	0.413718	hypothetical protein
XP_009690897.1	0.0730581	0.97189	0.0751712	0.0427137	0.746279	0.0572356	0.066203	hypothetical protein
XP_009690898.1	0.101668	0.890293	0.114196				0.114196	conserved hypothetical protein
XP_009690899.1	0.0511493	0.274779	0.186147	0.0183576	0.468658	0.0391705	0.112659	conserved hypothetical protein
XP_009690900.1	0.0465527	0.619467	0.0751496	0.0340983	0.431144	0.079088	0.077119	dolichol phosphate mannose synthase
XP_009690903.1	0.133437	0.598607	0.222912	0.121895	0.413039	0.295119	0.259016	uncharacterized protein
XP_009690905.1	0.0598408	0.52945	0.113024	0.0355537	0.440806	0.0806562	0.09684	CTP:phosphorylcholine cytidylyltransferase
XP_009690906.1	0.0460753	0.713308	0.0645939	0.049165	0.780854	0.0629631	0.063778	formin binding protein 3
XP_009690907.1	0.0412082	0.625252	0.0659066	0.0316191	0.428485	0.0737928	0.06985	uncharacterized protein
XP_009690908.1	0.029557	0.391912	0.0754175	0.0162813	0.312602	0.0520831	0.06375	CDP-diacylglycerol-inositol 3-phosphatidyltransferase
XP_009690909.1	0.0618533	0.449897	0.137483	0.0450506	0.236881	0.190183	0.163833	uncharacterized protein
XP_009690910.1	0.0855007	0.308723	0.276949	0.0738771	0.513752	0.143799	0.210374	conserved hypothetical protein
XP_009690911.1	0.0541546	0.329665	0.164271				0.164271	cathepsin-like cysteine protease
XP_009690912.1	0.0198181	0.526386	0.0376495	0.0267915	0.382972	0.0699568	0.053803	uncharacterized protein
XP_009690913.1	0.0274615	0.159922	0.171718				0.171718	uncharacterized protein
XP_009690914.1	0.104883	0.290881	0.360569				0.360569	conserved hypothetical protein
XP_009690915.1	0.0375423	0.319014	0.117682	0.0216731	0.285348	0.0759531	0.096818	uncharacterized protein
XP_009690916.1	0.0420726	0.209515	0.200809				0.200809	uncharacterized protein
XP_009690917.1	0.031772	0.299596	0.106049				0.106049	ADP-ribosylation factor
XP_009690918.1	0.00469889	0.148559	0.0316298				0.0316298	uncharacterized protein
XP_009690919.1	0.0357145	0.380677	0.0938183	0.0385282	0.35166	0.109561	0.10169	DNA primase small subunit
XP_009690920.1	0.0258119	0.577956	0.0446607	0.0215503	0.540733	0.0398539	0.042257	CTP synthase
XP_009690921.1	NA	NA	NA				NA	hypothetical protein
XP_009690922.1	0.025904	0.359556	0.0720445				0.0720445	subunit of proteaseome activator complex
XP_009690923.1	0.0509023	0.626113	0.081299	0.0527861	0.776547	0.0679754	0.074637	conserved hypothetical protein
XP_009690924.1	0.0543464	1.55758	0.0348915	0.0682632	3.4133	0.0199992	0.027445	hypothetical protein
XP_009690926.1	0.0837475	0.418939	0.199904	0.0909765	0.272261	0.334152	0.267028	conserved hypothetical protein
XP_009690927.1	0.165434	0.485856	0.340499	0.121566	0.301234	0.40356	0.37203	hypothetical protein
XP_009690928.1	0.0694555	0.825179	0.0841703	0.0714826	0.696964	0.102563	0.093367	uncharacterized protein
XP_009690930.1	0.0485372	0.527688	0.0919808	0.0340129	0.483222	0.0703877	0.081184	conserved hypothetical protein
XP_009690931.1	0.102059	0.353171	0.288979				0.288979	hypothetical protein
XP_009690932.1	0.166404	0.512695	0.324568	0.150477	0.454032	0.331424	0.327996	conserved hypothetical protein
XP_009690933.1	0.177366	0.172833	1.02623	0.181529	0.166913	1.08757	1.0569	hypothetical protein
XP_009690934.1	0.107758	0.692105	0.155696				0.155696	conserved hypothetical protein
XP_009690935.1	0.0989347	0.491864	0.201142	0.102361	0.385846	0.265291	0.233216	uncharacterized protein
XP_009690936.1	0.100034	0.852036	0.117406	0.0932501	0.688005	0.135537	0.126471	conserved hypothetical protein
XP_009690937.1	0.0292083	0.584182	0.0499986	0.0263559	0.73339	0.0359371	0.042968	uncharacterized protein
XP_009690938.1	0.109293	0.889256	0.122903	0.0882138	0.813009	0.108503	0.115703	uncharacterized protein
XP_009690939.1	0.142813	0.34002	0.420013				0.420013	hypothetical protein
XP_009690940.1	0.0817513	0.122635	0.666624	0.0532987	0.11323	0.470713	0.568669	hypothetical protein
XP_009690941.1	0.0512576	1.04249	0.0491682	0.0514402	0.914428	0.0562539	0.052711	uncharacterized protein
XP_009690942.1	0.107316	0.179439	0.598065	0.2524	0.235258	1.07286	0.835462	hypothetical protein
XP_009690943.1	0.0526766	0.807819	0.0652085	0.0522311	0.842548	0.0619919	0.0636	protein phosphatase 2C homolog 2
XP_009690944.1	0.130572	0.295286	0.442188				0.442188	hypothetical protein
XP_009690945.1	0.133513	0.378313	0.352918	0.131673	0.35128	0.374837	0.363877	conserved hypothetical protein
XP_009690946.1	0.130123	0.443966	0.293091	0.10394	0.397588	0.261425	0.277258	conserved hypothetical protein
XP_009690948.1	0.152875	0.434569	0.351785				0.351785	conserved hypothetical protein
XP_009690949.1	0.0109523	0.501145	0.0218545	0.00158111	0.379191	0.00416971	0.013012	serine/threonine protein phosphatase pp2a catalytic subunit
XP_009690950.1	0.0788124	0.880922	0.0894658	0.0383582	0.315613	0.121536	0.105501	ubiquitin-related modifier 1 homolog
XP_009690951.1	0.0345858	0.560273	0.0617304	0.0182852	0.440537	0.0415065	0.051618	conserved hypothetical protein
XP_009690952.1				0.0958485	0.831627	0.115254	0.115254	conserved hypothetical protein
XP_009690953.1	0.0351983	0.742137	0.0474284	0.0364058	0.70904	0.0513452	0.049387	guanylyl cyclase
XP_009690954.1	0.0614363	0.411989	0.149121	0.0583297	0.355326	0.164158	0.156639	uncharacterized protein

protein_id	Chitose_ka	Chitose_ks	Chitose_kaks	Buffeli_ka	Buffeli_ks	Buffeli_kaks	mean_kaks	function
XP_009690955.1	0.102666	0.714938	0.143601				0.143601	uncharacterized protein
XP_009690956.1	0.0692112	0.183609	0.37695	0.078235	0.234893	0.333067	0.355009	conserved hypothetical protein
XP_009690958.1	0.0745227	0.476307	0.156459	0.058627	0.39995	0.146586	0.151523	conserved hypothetical protein
XP_009690959.1	0.0844432	0.431091	0.195882	0.0717836	0.313102	0.229266	0.212574	uncharacterized protein
XP_009690962.1	0.15549	0.631351	0.246281				0.246281	conserved hypothetical protein
XP_009690963.1	0.117576	0.278016	0.422909	0.0830849	0.163194	0.509116	0.466012	hypothetical protein
XP_009690964.1	0.079451	0.222165	0.357622				0.357622	conserved hypothetical protein
XP_009690965.1	0.0892865	0.876635	0.101851	0.0956386	0.854001	0.111989	0.10692	conserved hypothetical protein
XP_009690966.1	0.0607424	0.373811	0.162495	0.0423216	0.360364	0.117441	0.139968	uncharacterized protein
XP_009690967.1	0.00764254	1.11498	0.0068544	0.00748765	0.889806	0.00841493	0.007635	26S proteasome ATPase subunit
XP_009690968.1	0.0956935	0.447831	0.213682	0.103076	0.476713	0.216222	0.214952	hypothetical protein
XP_009690969.1	0.0568612	0.765387	0.0742909	0.0418479	0.583475	0.0717218	0.073006	conserved hypothetical protein
XP_009690971.1	0.14611	1.23204	0.118592	0.0530177	1.10816	0.0478429	0.083217	conserved hypothetical protein
XP_009690972.1	0.0902161	0.975917	0.0924424	0.0669647	0.955085	0.0701139	0.081278	conserved hypothetical protein
XP_009690973.1	0.0991891	0.716355	0.138464	0.0932158	0.779062	0.119651	0.129057	conserved hypothetical protein
XP_009690974.1	0.071555	0.482775	0.148216	0.0584147	0.421223	0.138679	0.143448	uncharacterized protein
XP_009690975.1	0.0259242	0.426978	0.0607154	0.0415314	0.517288	0.0802869	0.070501	FKBP-type peptidyl-prolyl cis-trans isomerase
XP_009690976.1	0.0692835	0.638296	0.108544	0.0700795	0.702809	0.0997135	0.104129	conserved hypothetical protein
XP_009690977.1	0.00111542	0.921202	0.00121083	0.0010788	0.929134	0.00116108	0.001186	actin 1
XP_009690979.1	0.0744459	0.440931	0.168838	0.0569929	0.524607	0.108639	0.138738	conserved hypothetical protein
XP_009690980.1	0.0487132	0.460985	0.105672	0.0397905	0.447104	0.0889961	0.097334	mono-oxygenase
XP_009690981.1	0.129931	0.723911	0.179485	0.124822	1.05814	0.117963	0.148724	conserved hypothetical protein
XP_009690982.1	0.0420778	0.357157	0.117813	0.0479718	0.441947	0.108546	0.11318	conserved hypothetical protein
XP_009690983.1	0.0654655	0.505454	0.129518	0.0451437	0.364842	0.123735	0.126627	uncharacterized protein
XP_009690984.1	0.139746	0.620404	0.22525				0.22525	uncharacterized protein
XP_009690985.1	0.0400657	0.776014	0.0516301	0.0757547	0.861191	0.0879651	0.069798	5'-3' exonuclease
XP_009690986.1	0.061651	0.160048	0.385202				0.385202	Ran binding protein
XP_009690987.1	0.0484987	0.556779	0.0871058	0.0429895	0.404007	0.106408	0.096757	conserved hypothetical protein
XP_009690988.1	0.0643698	0.605976	0.106225	0.0416789	0.507091	0.0821921	0.094209	26S protease subunit
XP_009690989.1	0.0676915	0.957078	0.0707273	0.0771276	0.667032	0.115628	0.093178	formate/nitrate transporter
XP_009690990.1	0.0850886	0.856053	0.0993964	0.0848316	0.71847	0.118073	0.108735	uncharacterized protein
XP_009690991.1	0.0625161	0.509917	0.122601	0.0521278	0.475688	0.109584	0.116093	uncharacterized protein
XP_009690992.1	0.118698	0.287827	0.412394	0.10793	0.246633	0.437614	0.425004	conserved hypothetical protein
XP_009690993.1	0.273342	0.838558	0.325967	0.133275	0.195506	0.681691	0.503829	hypothetical protein
XP_009690994.1	0.0139661	0.412352	0.0338694	0.0141762	0.556562	0.025471	0.02967	conserved hypothetical protein
XP_009690995.1	0.0650822	0.652538	0.0997372	0.0628292	0.557279	0.112743	0.10624	uncharacterized protein
XP_009690996.1	0.0217961	0.547444	0.0398144	0.0222515	0.350233	0.0635335	0.051674	uncharacterized protein
XP_009690997.1	0.0913462	0.690843	0.132224	0.0830297	0.459476	0.180705	0.156465	uncharacterized protein
XP_009690998.1	0.0917985	0.817419	0.112303	0.0823926	0.856664	0.0961784	0.104241	phosphoglucosamine mutase
XP_009691002.1	0.0572696	0.452538	0.126552				0.126552	conserved hypothetical protein
XP_009691005.1	0.145961	0.424405	0.343921	0.109333	0.427582	0.255701	0.299811	conserved hypothetical protein
XP_009691006.1	0.161189	0.107099	1.50506	0.202827	0.170239	1.19142	1.34824	conserved hypothetical protein
XP_009691007.1	0.0503007	0.814588	0.0617499	0.0403978	0.711009	0.0568175	0.059284	conserved hypothetical protein
XP_009691008.1	0.0418702	0.258486	0.161982				0.161982	conserved hypothetical protein
XP_009691009.1	0.0742908	0.738462	0.100602	0.0757424	0.583457	0.129817	0.115209	uncharacterized protein
XP_009691010.1	0.0436894	0.664018	0.0657955	0.0359279	0.741779	0.0484348	0.057115	uncharacterized protein
XP_009691011.1	0.0427673	1.26832	0.0337196	0.035725	1.1346	0.0314867	0.032603	glucosamine--fructose-6-phosphate aminotransferase
XP_009691012.1	0.0691034	0.458252	0.150798	0.0719966	0.45168	0.159397	0.155097	uncharacterized protein
XP_009691013.1	0.0203565	0.537211	0.037893	0.0236767	0.50919	0.0464988	0.042196	molecular chaperone protein
XP_009691014.1	0.0261366	1.05447	0.0247865	0.0238661	1.04494	0.0228396	0.023813	uncharacterized protein
XP_009691015.1	0.138836	0.469804	0.295519	0.123709	0.401292	0.308277	0.301898	conserved hypothetical protein
XP_009691016.1	0.0339754	0.346675	0.0980038	0.0402973	0.409971	0.098293	0.098148	uncharacterized protein
XP_009691017.1	0.0400043	0.432261	0.0925467	0.0364293	0.372651	0.097757	0.095152	aldo-keto reductase

protein_id	Chitose_ka	Chitose_ks	Chitose_kaks	Buffeli_ka	Buffeli_ks	Buffeli_kaks	mean_kaks	function
XP_009691018.1	0.0496784	0.602539	0.0824485	0.0548227	0.57942	0.0946164	0.088532	conserved hypothetical protein
XP_009691019.1	0.0369618	0.69605	0.0531022	0.0326883	0.691132	0.0472967	0.050199	CTD-like phosphatase
XP_009691020.1	0.0292962	0.554412	0.0528419	0.0251894	0.672113	0.0374779	0.04516	serine/threonine protein phosphatase
XP_009691022.1	0.0178532	0.238747	0.0747789				0.0747789	uncharacterized protein
XP_009691023.1	0.0311946	0.180096	0.173211	0.04348	0.311094	0.139765	0.156488	conserved hypothetical protein
XP_009691024.1	0.106597	0.463035	0.230213	0.0825789	0.431023	0.191588	0.2109	conserved hypothetical protein
XP_009691025.1	0.00501399	0.348243	0.014398	0.00264999	0.629878	0.00420716	0.009303	40S ribosomal protein S8
XP_009691026.1				0.108106	0.769038	0.140573	0.140573	conserved hypothetical protein
XP_009691027.1	0.0865761	0.797488	0.108561	0.0852322	0.913699	0.0932826	0.100922	uncharacterized protein
XP_009691030.1				0.106393	0.497729	0.213757	0.213757	conserved hypothetical protein
XP_009691031.1	0.0538029	0.615067	0.0874749				0.0874749	ABC transporter
XP_009691032.1				0.0870796	0.357273	0.243734	0.243734	conserved hypothetical protein
XP_009691051.1	0.0811435	0.7086	0.114512	0.0844729	0.979671	0.0862258	0.100369	uncharacterized protein
XP_009691052.1	0.0696696	0.757923	0.0919217				0.0919217	conserved hypothetical protein
XP_009691053.1	0.132841	0.884184	0.150242	0.114638	0.794017	0.144377	0.147309	uncharacterized protein
XP_009691055.1	0.132008	0.603674	0.218674	0.0846134	0.737085	0.114795	0.166735	uncharacterized protein
XP_009691056.1	0.118667	0.768277	0.154459	0.15239	0.991339	0.153721	0.15409	uncharacterized protein
XP_009691057.1	0.0578517	0.690208	0.0838178	0.0594354	0.656518	0.0905312	0.087175	conserved hypothetical protein
XP_009691058.1	0.293715	0.125505	2.34027	0.365665	0.294328	1.24237	1.79132	hypothetical protein
XP_009691059.1	0.109751	0.859503	0.127691	0.111342	0.828485	0.134392	0.131042	uncharacterized protein
XP_009691060.1	0.00230364	0.536468	0.00429408	0.000683116	0.683116	0.001	0.002647	40S ribosomal protein S5
XP_009691061.1	0.0358191	0.285405	0.125503	0.0219397	0.317784	0.0690398	0.097271	ADP-ribosylation factor
XP_009691062.1	0.0426593	0.478892	0.0890791	0.032766	0.513079	0.0638615	0.07647	conserved hypothetical protein
XP_009691063.1	0.00903106	0.452529	0.0199569	0.0294053	0.423932	0.0693632	0.04466	multiprotein bridging factor type 1
XP_009691064.1	0.024853	0.321804	0.07723	0.0326981	0.272084	0.120177	0.098703	farnesyl pyrophosphate synthetase
XP_009691065.1	0.0951935	0.426696	0.223094	0.0935362	0.422112	0.221591	0.222342	conserved hypothetical protein
XP_009691066.1	0.0340491	0.588859	0.0578222	0.0108464	0.116236	0.0933129	0.075568	conserved hypothetical protein
XP_009691067.1	0.0550497	0.476687	0.115484	0.0405704	0.38037	0.10666	0.111072	uncharacterised trophozoite protein
XP_009691068.1	0.0228366	0.902829	0.0252945	0.016069	0.767277	0.0209429	0.023119	vacuolar ATP synthase subunit E
XP_009691069.1	0.00271884	0.529923	0.00513062	0.00266968	0.537226	0.00496937	0.00505	uncharacterized protein
XP_009691070.1	0.0979515	0.444922	0.220154	0.0893929	0.386111	0.231522	0.225838	multicopy suppressor of ts gsp1
XP_009691071.1	0.0290878	0.262364	0.110868				0.110868	uncharacterized protein
XP_009691072.1	0.0928279	0.656568	0.141384	0.0970235	0.891816	0.108793	0.125088	conserved hypothetical protein
XP_009691074.1	0.0893215	0.690032	0.129446	0.0916157	0.60196	0.152196	0.140821	conserved hypothetical protein
XP_009691075.1	0.0823029	0.652668	0.126102	0.0826629	0.819319	0.100892	0.113497	uncharacterized protein
XP_009691076.1	0.0665788	0.318213	0.209227	0.0618045	0.368804	0.167581	0.188404	uncharacterized protein
XP_009691077.1	0.0495778	0.594602	0.0833799	0.0450367	0.571235	0.0788409	0.08111	GPI anchor transamidase
XP_009691078.1	0.0295734	0.136185	0.217156	0.0495677	0.296088	0.167408	0.192282	conserved hypothetical protein
XP_009691079.1	0.0251661	0.179843	0.139934				0.139934	nuclear transcription factor Y subunit B-8
XP_009691081.1	NA	NA	NA				NA	serine/threonine protein kinase
XP_009691082.1	0.0826726	0.863916	0.0956952	0.0651807	0.869612	0.0749538	0.085324	uncharacterized protein
XP_009691083.1	0.0003554	0.3554	0.001				0.001	uncharacterized protein
XP_009691084.1	0.0786521	0.247782	0.317424	0.061354	0.29136	0.210578	0.264001	uncharacterized protein
XP_009691085.1	0.0380955	0.447822	0.0850683	0.0378372	0.452944	0.0835363	0.084302	conserved hypothetical protein
XP_009691086.1	0.107784	0.532046	0.202584	0.0993157	0.44334	0.224017	0.213301	conserved hypothetical protein
XP_009691087.1	0.0687467	0.346517	0.198393	0.0679477	0.364109	0.186614	0.192503	integral membrane protein
XP_009691088.1	0.0899323	0.312935	0.287384	0.0869742	0.349668	0.248733	0.268058	cytochrome c oxidase copper chaperone%2C Dopuin
XP_009691089.1	0.08895	0.763427	0.116514	0.104342	1.00768	0.103547	0.110031	conserved hypothetical protein
XP_009691090.1	0.022776	0.417895	0.0545017				0.0545017	conserved hypothetical protein
XP_009691092.1	0.0674331	0.316403	0.213124	0.037569	0.215411	0.174406	0.193765	uncharacterized protein
XP_009691093.1	0.0305076	0.472751	0.0645321	0.0209067	0.392285	0.0532946	0.058913	DEAD-box RNA helicase
XP_009691094.1	0.0403494	0.963062	0.041897	0.0372816	0.884813	0.0421349	0.042016	transcription factor
XP_009691095.1	0.110374	0.431096	0.256032	0.0969385	0.345558	0.280528	0.26828	conserved hypothetical protein

protein_id	Chitose_ka	Chitose_ks	Chitose_kaks	Buffeli_ka	Buffeli_ks	Buffeli_kaks	mean_kaks	function
XP_009691096.1	0.0734399	0.852159	0.086181	0.0642648	0.781938	0.0821865	0.084184	uncharacterized protein
XP_009691097.1	0.0790306	0.727107	0.108692	0.045922	0.634048	0.0724267	0.090559	conserved hypothetical protein
XP_009691098.1	0.077783	0.646833	0.120252	0.05344	0.504458	0.105935	0.113093	histone deacetylase complex subunit Sap18-like protein
XP_009691099.1	0.0348219	0.396217	0.0878859	0.0272165	0.371537	0.0732538	0.08057	conserved hypothetical protein
XP_009691100.1	0.000516374	0.516374	0.001	0.000239025	0.239025	0.001	0.001	Tgf beta-inducible nuclear protein 1
XP_009691101.1	0.0633162	0.363756	0.174062	0.0354926	0.277509	0.127897	0.150979	uncharacterized protein
XP_009691102.1	0.0364597	0.489919	0.0744198	0.0464547	0.494586	0.0939264	0.084173	conserved hypothetical protein
XP_009691103.1	0.0274412	0.487209	0.0563233	0.0257342	0.470115	0.0547401	0.055532	elongation factor g 1%2C mitochondrial
XP_009691104.1	0.0236497	0.29269	0.0808012	0.0177335	0.38028	0.0466327	0.063717	conserved hypothetical protein
XP_009691105.1	0.00795223	0.385076	0.0206511				0.0206511	hypothetical protein
XP_009691106.1	0.102935	0.492727	0.208909	0.0959872	0.432615	0.221877	0.215393	conserved hypothetical protein
XP_009691107.1	0.0680788	0.482043	0.14123	0.0675566	0.462889	0.145946	0.143588	uncharacterized protein
XP_009691108.1	0.00188991	0.20594	0.00917696	0.0103099	0.289839	0.0355711	0.022374	proteasome subunit alpha type 2
XP_009691109.1	0.0217205	0.316453	0.0686372	0.00499652	0.398247	0.0125463	0.040592	uncharacterized protein
XP_009691110.1	NA	NA	NA	0.0253836	0.308588	0.082257	NA	conserved hypothetical protein
XP_009691111.1	0.0379387	0.330896	0.114654	0.0443796	0.366904	0.120957	0.117806	Plasmodium falciparum CPW-WPC repeat containing protein
XP_009691112.1	0.0397571	0.528422	0.0752373	0.0420881	0.465114	0.0904899	0.082864	signal peptidase
XP_009691113.1	0.0818761	0.418918	0.195447	0.056245	0.425992	0.132033	0.16374	conserved hypothetical protein
XP_009691114.1	0.0293163	0.300231	0.0976457	0.045273	0.278066	0.162814	0.13023	4-hydroxybenzoate octaprenyltransferase
XP_009691115.1	0.0420921	0.337857	0.124586	0.0232732	0.234338	0.0993146	0.11195	uncharacterized protein
XP_009691116.1	0.0461848	0.389558	0.118557	0.0459583	0.373172	0.123156	0.120857	conserved hypothetical protein
XP_009691117.1	0.0188521	0.389511	0.0483995	0.0281481	0.323238	0.0870817	0.067741	uncharacterized protein
XP_009691118.1	0.0871051	0.457709	0.190307	0.0849462	0.472186	0.1799	0.185104	conserved hypothetical protein
XP_009691119.1	0.022917	0.217785	0.105228	0.0331516	0.308683	0.107397	0.106313	uncharacterized protein
XP_009691120.1	0.0523869	0.252244	0.207683	0.0279378	0.118463	0.235836	0.221759	conserved hypothetical protein
XP_009691121.1	0.0721777	0.464211	0.155485				0.155485	uncharacterized protein
XP_009691122.1	0.0675011	0.430239	0.156892	0.0567025	0.396865	0.142876	0.149884	uncharacterized protein
XP_009691123.1	0.0685476	0.38308	0.178938	0.0724858	0.37645	0.192551	0.185745	uncharacterized protein
XP_009691124.1	0.0706146	0.531358	0.132895	0.0552727	0.51524	0.107276	0.120086	uncharacterized protein
XP_009691125.1	0.0456161	0.62748	0.0726972	0.0419905	0.54036	0.0777082	0.075203	eukaryotic translation initiation factor 3 subunit 7
XP_009691126.1	0.07689	0.720735	0.106683	0.0606545	0.49028	0.123714	0.115199	conserved hypothetical protein
XP_009691127.1	0.0724517	0.485164	0.149334	0.0594995	0.451359	0.131823	0.140578	conserved hypothetical protein
XP_009691128.1	0.0420918	0.615524	0.0683838	0.0421408	0.497605	0.0846872	0.076536	conserved hypothetical protein
XP_009691129.1	0.0398014	0.34098	0.116726	0.0513157	0.305336	0.168063	0.142394	conserved hypothetical protein
XP_009691130.1	0.061978	0.215012	0.288254	0.0574123	0.120073	0.478146	0.3832	conserved hypothetical protein
XP_009691131.1	0.147954	0.348825	0.424151	0.130299	0.323981	0.402179	0.413165	conserved hypothetical protein
XP_009691132.1	0.0207313	0.403883	0.05133	0.0159159	0.468015	0.0340074	0.042669	conserved hypothetical protein
XP_009691133.1	0.0413563	0.315688	0.131003	0.0270362	0.323829	0.0834891	0.107246	conserved hypothetical protein
XP_009691134.1	0.0488716	0.341506	0.143106	0.0392765	0.240808	0.163103	0.153105	uncharacterized protein
XP_009691135.1	0.00585951	0.355988	0.0164598	0.00389354	0.315184	0.0123532	0.014406	Ras-related protein Rab-1
XP_009691136.1	0.0357897	0.366693	0.0976012	0.0131322	0.425424	0.0308684	0.064235	ribosomal protein S9
XP_009691137.1	0.0674107	0.667823	0.100941	0.0715074	0.659056	0.1085	0.10472	adenylosuccinate lyase
XP_009691138.1	0.0419205	0.455464	0.092039	0.0479839	0.433482	0.110694	0.101366	conserved hypothetical protein
XP_009691139.1	0.0647565	0.33248	0.194768	0.0587504	0.328893	0.178631	0.1867	uncharacterized protein
XP_009691140.1	0.0125197	0.447836	0.027956	0.000390696	0.390696	0.001	0.014478	conserved hypothetical protein
XP_009691141.1	0.0587462	0.442003	0.132909	0.0361376	0.276251	0.130815	0.131862	conserved hypothetical protein
XP_009691145.1	0.00998895	0.132524	0.0753745	0.00684436	0.13965	0.049011	0.062193	nucleoside diphosphate hydrolase
XP_009691146.1	0.0311019	0.570241	0.0545417	0.034181	0.700707	0.0487806	0.051661	zinc transport protein
XP_009691147.1	0.105094	0.40067	0.262295	0.052031	0.280935	0.185207	0.223751	conserved hypothetical protein
XP_009691148.1	0.118362	0.629586	0.188	0.11449	0.603191	0.189807	0.188904	conserved hypothetical protein
XP_009691149.1	0.0533967	0.557924	0.0957061	0.0371468	0.604911	0.0614087	0.078557	conserved hypothetical protein
XP_009691150.1	0.0172959	0.527834	0.0327677	0.015007	0.529029	0.0283671	0.030567	conserved hypothetical protein
XP_009691151.1	0.0954782	0.365695	0.261087	0.0837454	0.374218	0.223788	0.242438	conserved hypothetical protein

protein_id	Chitose_ka	Chitose_ks	Chitose_kaks	Buffeli_ka	Buffeli_ks	Buffeli_kaks	mean_kaks	function
XP_009691152.1	0.0243691	0.560314	0.0434918	0.0212621	0.532216	0.0399502	0.041721	uncharacterized protein
XP_009691153.1	0.0412583	0.224635	0.183668				0.183668	uncharacterized protein
XP_009691154.1	0.00857511	0.562091	0.0152557	0.000568173	0.568173	0.001	0.008128	60S ribosomal protein L15
XP_009691155.1	0.105053	0.479086	0.219278	0.0967455	0.526217	0.183851	0.201564	conserved hypothetical protein
XP_009691156.1	0.0291618	0.594285	0.0490704	0.0322966	0.55257	0.0584481	0.053759	uncharacterized protein
XP_009691157.1	0.0579948	0.702691	0.0825324	0.0462207	0.615989	0.075035	0.078784	uncharacterized protein
XP_009691158.1	0.070086	0.857447	0.081738	0.0616969	0.90054	0.068511	0.075125	conserved hypothetical protein
XP_009691159.1	0.0337986	0.883338	0.0382624	0.0291584	0.843059	0.0345864	0.036424	U5 small nuclear ribonucleoprotein-specific helicase
XP_009691161.1	0.0195119	0.919949	0.0212098	0.0149018	0.750041	0.019868	0.020539	DNA-directed RNA polymerase III
XP_009691162.1	0.000468192	0.468192	0.001	0.000413237	0.413237	0.001	0.001	40S ribosomal protein S23
XP_009691163.1	0.0176219	0.549987	0.0320405	0.0147564	0.583186	0.0253031	0.028672	60S ribosomal protein L7
XP_009691164.1	0.0514541	0.532074	0.0967048	0.0393903	0.384762	0.102376	0.09954	uncharacterized protein
XP_009691165.1	0.0322307	0.314914	0.102347	0.0313469	0.217109	0.144383	0.123365	conserved hypothetical protein
XP_009691166.1	0.0713165	0.50596	0.140953	0.0642862	0.356342	0.180406	0.16068	conserved hypothetical protein
XP_009691167.1	0.185941	0.0234537	7.928	0.015448	0.000308959	50	28.964	hypothetical protein
XP_009691168.1	0.0711561	0.400871	0.177504	0.0669349	0.33887	0.197524	0.187514	conserved hypothetical protein
XP_009691169.1	0.0405869	0.766995	0.0529168	0.0442756	0.865768	0.0511402	0.052028	uncharacterized protein
XP_009691171.1	0.0560585	0.519673	0.107873	0.0634204	0.436517	0.145287	0.12658	uncharacterized protein
XP_009691172.1	0.000202056	0.202056	0.001	0.00005339	0.05339	0.001	0.001	uncharacterized protein
XP_009691173.1	0.0366237	0.221022	0.165701				0.165701	light induced protein like protein
XP_009691174.1	0.0552401	0.390953	0.141296	0.0646455	0.308806	0.20934	0.175318	uncharacterized protein
XP_009691175.1	0.0785191	0.421063	0.186478	0.0532525	0.312499	0.170408	0.178443	uncharacterized protein
XP_009691177.1	0.081303	0.464949	0.174864	0.0877187	0.367491	0.238696	0.20678	conserved hypothetical protein
XP_009691178.1	0.0472974	0.759828	0.0622475	0.0387222	1.66917	0.0231985	0.042723	conserved hypothetical protein
XP_009691179.1	0.0201857	0.654493	0.0308417	0.0166095	0.656832	0.0252872	0.028064	FAD-dependent glycerol-3-phosphate dehydrogenase
XP_009691181.1	0.0581283	0.392984	0.147915	0.042603	0.275477	0.154652	0.151284	conserved hypothetical protein
XP_009691182.1	0.055221	0.363186	0.152046	0.0416901	0.216209	0.192823	0.172434	conserved hypothetical protein
XP_009691183.1	0.0602054	0.450368	0.133681				0.133681	conserved hypothetical protein
XP_009691184.1	0.0499884	0.35604	0.140401	0.0523571	0.352674	0.148457	0.144429	conserved hypothetical protein
XP_009691185.1				0.00730671	0.265468	0.0275239	0.0275239	peptide chain release factor 2
XP_009691186.1	0.200811	0.12767	1.57289	0.134272	0.0539376	2.4894	2.031145	conserved hypothetical protein
XP_009691187.1	0.0209066	0.903497	0.0231397	0.0147501	0.615462	0.0239658	0.023553	T-complex protein subunit beta
XP_009691188.1	0.0742637	1.06537	0.0697068	0.0753784	0.933774	0.0807244	0.075216	conserved hypothetical protein
XP_009691189.1	0.131631	0.73452	0.179207	0.110122	0.641046	0.171784	0.175495	conserved hypothetical protein
XP_009691190.1	0.198632	0.379926	0.522817	0.174829	0.354094	0.493735	0.508276	exon junction complex protein
XP_009691191.1	0.116401	0.195687	0.594834	3.92624E-05	0.0392624	0.001	0.297917	hypothetical protein
XP_009691192.1	0.0181409	1.82924	0.00991719	0.0293172	1.59875	0.0183376	0.014127	eukaryotic translation initiation factor 5
XP_009691193.1	0.149147	0.167137	0.892364	0.133338	0.0854536	1.56036	1.226362	hypothetical protein
XP_009691194.1	0.00307604	0.514453	0.00597925	0.000426216	0.426216	0.001	0.00349	40S ribosomal protein S12
XP_009691195.1	0.00864934	0.421124	0.0205387	0.000334459	0.334459	0.001	0.010769	40S ribosomal protein S19
XP_009691196.1	0.010887	0.341111	0.0319161	0.0180707	0.37883	0.0477014	0.039809	RabGDI protein
XP_009691197.1	0.0806208	0.0875884	0.920451	NA	NA	NA	NA	conserved hypothetical protein
XP_009691198.1	0.0759003	0.43103	0.17609	0.0371596	0.340238	0.109217	0.142653	uncharacterized protein
XP_009691199.1	0.344988	0.00689975	50	0.278414	0.00556829	50	50	hypothetical protein
XP_009691200.1	0.0458242	0.212805	0.215334	0.0369225	0.190025	0.194303	0.204819	conserved hypothetical protein
XP_009691201.1	0.0789134	0.242208	0.325809	0.0720981	0.313838	0.229731	0.27777	protein farnesyltransferase subunit alpha
XP_009691202.1	0.0802234	0.511243	0.156918	0.0706603	0.421142	0.167783	0.162351	conserved hypothetical protein
XP_009691203.1	0.164049	0.229355	0.715264	0.0605494	0.0570691	1.06098	0.888122	hypothetical protein
XP_009691204.1	0.00031605	0.31605	0.001	0.000090518	0.090518	0.001	0.001	40S ribosomal protein S17
XP_009691205.1	0.0581525	0.312437	0.186125	0.0564878	0.531395	0.106301	0.146213	uncharacterized protein
XP_009691206.1	0.0516575	0.284709	0.18144				0.18144	uncharacterized protein
XP_009691207.1	0.0631918	0.370437	0.170587	0.0479994	0.361956	0.132611	0.151599	conserved hypothetical protein
XP_009691208.1	0.00950732	0.502515	0.0189195	0.0086903	0.436815	0.0198947	0.019407	uncharacterized protein

protein_id	Chitose_ka	Chitose_ks	Chitose_kaks	Buffeli_ka	Buffeli_ks	Buffeli_kaks	mean_kaks	function
XP_009691209.1	0.00717793	0.492794	0.0145658	0.00278745	0.502712	0.00554484	0.010055	uncharacterized protein
XP_009691210.1	0.126875	0.301713	0.420515				0.420515	conserved hypothetical protein
XP_009691212.1	0.0946624	0.677958	0.139629	0.0851154	0.795731	0.106965	0.123297	conserved hypothetical protein
XP_009691213.1	0.00678819	0.677111	0.0100252	0.00969549	0.575853	0.0168367	0.013431	40S ribosomal protein S3a
XP_009691215.1	0.0170208	0.487616	0.0349062	0.0076761	0.548252	0.0140011	0.024454	elongation factor tu
XP_009691216.1	0.0398309	0.276327	0.144144				0.144144	protein kinase
XP_009691217.1	0.0296571	0.332884	0.0890912				0.0890912	uncharacterized protein
XP_009691218.1				0.000029903	0.029903	0.001	0.001	uncharacterized protein
XP_009691219.1	0.108738	0.0277711	3.91549	NA	NA	NA	NA	conserved hypothetical protein
XP_009691220.1	0.0543399	0.0010868	50				50	BIR protein
XP_009691221.1	0.0527043	0.230596	0.228557	0.0405764	0.180524	0.22477	0.226664	conserved hypothetical protein
XP_009691222.1	0.0430324	0.645539	0.0666612	0.0256768	0.545135	0.0471018	0.056882	uncharacterized protein
XP_009691223.1	0.0485202	0.547873	0.0885611	0.0286253	0.472292	0.0606094	0.074585	DNA topoisomerase III
XP_009691224.1	0.0402995	0.577754	0.0697521	0.0486948	0.590855	0.0824142	0.076083	RNA splicing factor
XP_009691225.1	0.0268155	0.557273	0.0481192	0.0381193	0.518906	0.0734609	0.06079	conserved hypothetical protein
XP_009691226.1	0.0192193	0.397794	0.0483148	0.00346849	0.277463	0.0125007	0.030408	conserved hypothetical protein
XP_009691227.1	0.0782685	0.67057	0.116719	0.0615017	0.50148	0.12264	0.119679	uncharacterized protein
XP_009691228.1	0.0133832	0.403998	0.033127				0.033127	vacuolar ATP synthase subunit G
XP_009691229.1	0.0123501	0.391878	0.0315151	0.00597915	0.488485	0.0122402	0.021878	uncharacterized protein
XP_009691230.1	0.0458118	0.344451	0.133	0.0173629	0.307839	0.0564024	0.094701	60S ribosomal protein L25
XP_009691231.1	0.0684477	0.481851	0.142052	0.0950769	0.62522	0.15207	0.147061	conserved hypothetical protein
XP_009691232.1	0.0677449	0.348629	0.194318	0.0462211	0.487131	0.0948843	0.144601	conserved hypothetical protein
XP_009691233.1	0.0186254	0.481823	0.0386562	0.0247557	0.521544	0.0474662	0.043061	integral membrane protein
XP_009691235.1				0.0996997	0.501928	0.198634	0.198634	uncharacterized protein
XP_009691236.1	0.0672224	0.529426	0.126972	0.0254889	0.323781	0.0787226	0.102847	aspartyl(acid) protease
XP_009691237.1	0.0730875	0.768611	0.0950904	0.0661877	0.836471	0.0791273	0.087109	ubiquitin carboxyl-terminal hydrolase
XP_009691238.1	0.0344729	0.45993	0.0749524	0.0429038	0.610937	0.0702262	0.072589	4-hydroxy-3-methylbut-2-enyl diphosphate reductase
XP_009691239.1	0.00840065	0.157	0.0535074				0.0535074	conserved hypothetical protein
XP_009691240.1	0.0380539	0.515414	0.0738318	0.0290236	0.609566	0.0476136	0.060723	ferrodoxin reductase-like protein
XP_009691241.1				0.0485671	0.886897	0.0547607	0.0547607	conserved hypothetical protein
XP_009691242.1	0.0502486	0.960755	0.0523012	0.042901	0.891853	0.0481032	0.050202	conserved hypothetical protein
XP_009691243.1	0.0523337	1.09638	0.047733	0.0432861	0.985902	0.0439051	0.045819	DEAD/DEAH-like helicase
XP_009691244.1	0.0108899	0.568507	0.0191553	0.0062295	0.491711	0.012669	0.015912	Ras-related GTPase
XP_009691245.1	0.0923418	0.676463	0.136507	0.114258	0.671211	0.170227	0.153367	uncharacterized protein
XP_009691246.1	0.0805862	0.911032	0.088456	0.0788068	0.839145	0.0939132	0.091185	conserved hypothetical protein
XP_009691247.1	0.0725738	0.532798	0.136213	0.0454602	0.347638	0.130769	0.133491	uncharacterized protein
XP_009691248.1	0.0636797	0.900122	0.0707457	0.0596588	0.789825	0.0755342	0.07314	conserved hypothetical protein
XP_009691249.1	0.0901962	0.355431	0.253766	0.105088	0.32054	0.327847	0.290806	uncharacterized protein
XP_009691250.1	0.118246	0.656373	0.180151	0.103047	0.663768	0.155246	0.167699	conserved hypothetical protein
XP_009691251.1	0.0475227	0.95729	0.0496429	0.0246531	0.66805	0.0369031	0.043273	synaptic glycoprotein sc2
XP_009691252.1	0.0105844	0.277823	0.0380976	0.0056891	0.265739	0.0214086	0.029753	uncharacterized protein
XP_009691254.1	0.0472867	0.383326	0.123359				0.123359	regulator of nonsense transcripts-related protein
XP_009691255.1	0.0511913	0.474507	0.107883				0.107883	conserved hypothetical protein
XP_009691257.1	0.0523875	1.25727	0.0416675	0.0577744	1.31104	0.0440677	0.042868	mRNA transport factor
XP_009691258.1	0.0833926	1.06723	0.0781392	0.0692435	1.07271	0.0645503	0.071345	conserved hypothetical protein
XP_009691259.1	0.0706694	1.01807	0.0694149	0.0767983	1.10476	0.069516	0.069465	conserved hypothetical protein
XP_009691261.1	0.0586056	0.979278	0.0598457	0.0517016	0.823747	0.0627639	0.061305	conserved hypothetical protein
XP_009691262.1	0.000106163	0.106163	0.001				0.001	centrin
XP_009691263.1	0.0788277	0.831806	0.0947669	0.10509	0.820416	0.128093	0.11143	conserved hypothetical protein
XP_009691264.1	0.0572899	0.310084	0.184756				0.184756	conserved hypothetical protein
XP_009691267.1	0.0222227	1.12042	0.0198343	0.0134382	0.213185	0.0630357	0.041435	60S ribosomal protein L35a
XP_009691268.1	0.0241041	0.516099	0.0467044	0.0348916	0.526676	0.0662487	0.056477	conserved hypothetical protein
XP_009691269.1	0.0690517	0.886544	0.0778886	0.0552034	0.587732	0.0939262	0.085907	cobalamin synthesis protein

protein_id	Chitose_ka	Chitose_ks	Chitose_kaks	Buffeli_ka	Buffeli_ks	Buffeli_kaks	mean_kaks	function
XP_009691270.1	0.0403324	0.660041	0.0611059				0.0611059	uncharacterized protein
XP_009691271.1	0.0709278	0.646921	0.109639	0.0480524	0.529766	0.090705	0.100172	conserved hypothetical protein
XP_009691272.1	0.0580128	1.22321	0.0474267	0.062424	0.751531	0.0830625	0.065245	conserved hypothetical protein
XP_009691273.1	0.0579474	0.651406	0.0889574	0.0513668	0.631991	0.0812777	0.085118	conserved hypothetical protein
XP_009691274.1	0.0271308	0.483054	0.0561651	0.0227478	0.461131	0.0493305	0.052748	uncharacterized protein
XP_009691275.1	0.0189326	0.594494	0.0318466	0.0170147	0.614065	0.0277082	0.029777	methionine aminopeptidase
XP_009691276.1	0.0217327	0.465883	0.0466484	0.0195274	0.372611	0.0524068	0.049528	uncharacterized protein
XP_009691277.1	0.0997412	0.74633	0.133642	0.0945371	0.679244	0.13918	0.136411	uncharacterized protein
XP_009691278.1	0.110856	0.980177	0.113098	0.0826856	0.793235	0.104238	0.108668	conserved hypothetical protein
XP_009691279.1	0.0389969	0.500888	0.0778554	0.0104991	0.467056	0.0224794	0.050167	conserved hypothetical protein
XP_009691280.1	0.000025147	0.025147	0.001	0.0239632	0.0256963	0.932555	0.466778	hypothetical protein
XP_009691281.1	0.0251261	0.616575	0.0407511	0.0254465	0.451634	0.0563431	0.048547	uncharacterized protein
XP_009691282.1	0.0832937	0.325276	0.256071	0.0821463	0.347695	0.236259	0.246165	queuine tRNA-ribosyltransferase-like protein
XP_009691283.1	0.0266903	0.503112	0.0530504	0.03548	0.376903	0.0941358	0.073593	conserved hypothetical protein
XP_009691284.1	0.0396508	1.06828	0.0371165	0.0404813	1.02963	0.0393165	0.038217	conserved hypothetical protein
XP_009691285.1	0.0890508	0.596237	0.149355				0.149355	conserved hypothetical protein
XP_009691286.1				0.114348	0.981787	0.116469	0.116469	conserved hypothetical protein
XP_009691287.1	0.0799226	0.927682	0.086153	0.067148	0.565627	0.118714	0.102433	conserved hypothetical protein
XP_009691288.1	0.0301747	0.493974	0.0610856	0.0185332	0.483909	0.0382989	0.049692	GTP-binding protein
XP_009691289.1	0.0176022	0.880755	0.0199854	0.016488	0.67966	0.0242591	0.022122	protein CWC15 homolog
XP_009691290.1	0.0210789	0.440095	0.0478962	0.013254	0.329297	0.0402494	0.044073	uncharacterized protein
XP_009691291.1	0.0420628	0.47546	0.0884674	0.0463221	0.38229	0.12117	0.104819	hypothetical protein
XP_009691292.1	0.0311554	0.512024	0.0608475	0.0326409	0.488251	0.0668528	0.06385	uncharacterized protein
XP_009691293.1	0.0510448	0.588099	0.0867962	0.0381284	0.595928	0.0639814	0.075389	uncharacterized protein
XP_009691294.1	0.0869708	0.360021	0.241571	0.0998465	0.570998	0.174863	0.208217	hypothetical protein
XP_009691295.1	0.0797652	0.54208	0.147147	0.073913	0.575604	0.128409	0.137778	hypothetical protein
XP_009691296.1	0.037866	0.423103	0.089496	0.0228639	0.38576	0.0592697	0.074383	integral membrane protein
XP_009691297.1	0.0155973	0.399395	0.0390522	0.0387556	0.16361	0.236879	0.137966	cold shock protein
XP_009691298.1	0.016852	0.588161	0.0286521	0.0081828	0.562888	0.0145372	0.021595	ATP synthase subunit alpha
XP_009691299.1	0.00472521	0.737067	0.00641083	NA	NA	NA	NA	conserved hypothetical protein
XP_009691300.1	0.0268717	0.752633	0.0357036	0.0242444	0.574848	0.0421754	0.03894	uncharacterized protein
XP_009691301.1	0.000327265	0.327265	0.001	0.00888143	0.357974	0.0248103	0.012905	ribosomal protein S30
XP_009691302.1	0.110508	0.538192	0.205332	0.0768709	0.352545	0.218046	0.211689	conserved hypothetical protein
XP_009691303.1	0.0600925	0.918061	0.0654559	0.0511736	0.509351	0.100468	0.082962	uncharacterized protein
XP_009691304.1	0.0194234	0.183904	0.105617	0.0168993	0.17134	0.0986303	0.102124	conserved hypothetical protein
XP_009691305.1	0.0062756	0.228364	0.0274807	0.0125653	0.30146	0.0416815	0.034581	hypothetical protein
XP_009691306.1	0.0523902	0.443313	0.118179	0.050372	0.459887	0.109531	0.113855	uncharacterized protein
XP_009691307.1	0.0160424	0.474293	0.0338238	0.0202871	0.334978	0.0605626	0.047193	uncharacterized protein
XP_009691308.1	0.13113	0.644701	0.203397	0.161928	0.677101	0.23915	0.221274	hypothetical protein
XP_009691309.1	0.070152	0.73989	0.0948141	0.0727478	0.329467	0.220804	0.157809	conserved hypothetical protein
XP_009691310.1	0.0619725	0.587579	0.105471	0.0781093	0.800843	0.0975339	0.101502	ribosome biogenesis protein
XP_009691311.1	0.0669677	0.734496	0.091175	0.0482805	0.620301	0.0778339	0.084504	conserved hypothetical protein
XP_009691312.1	0.0883092	0.341908	0.258283	0.0952533	0.258811	0.368042	0.313163	SOH1-like protein
XP_009691313.1	5.26216E-05	0.0526216	0.001				0.001	ubiquitin-like protein
XP_009691314.1	0.0333933	0.684025	0.0488188	0.0480541	0.698855	0.0687613	0.05879	uncharacterized protein
XP_009691315.1	0.0559552	0.856559	0.0653255	0.039571	0.679101	0.0582698	0.061798	conserved hypothetical protein
XP_009691316.1	0.066762	0.903925	0.0738579	0.0588179	0.932343	0.0630861	0.068472	molecular chaperone DnaJ
XP_009691317.1	0.0489765	0.420348	0.116514	0.0441744	0.497975	0.0887081	0.102611	conserved hypothetical protein
XP_009691318.1	0.016608	0.341609	0.048617	0.0346641	0.314133	0.110349	0.079483	ribose 5-phosphate epimerase
XP_009691319.1	0.029154	0.530581	0.0549473				0.0549473	conserved hypothetical protein
XP_009691320.1	0.0522335	0.788124	0.0662757	0.058979	0.757091	0.0779022	0.072089	conserved hypothetical protein
XP_009691321.1	0.0648179	0.568514	0.114013				0.114013	calcyclin binding protein-like
XP_009691322.1	0.043533	0.635137	0.0685412	0.11406	0.682047	0.167232	0.117887	nicotinate-nucleotide adenylyltransferase-like protein

protein_id	Chitose_ka	Chitose_ks	Chitose_kaks	Buffeli_ka	Buffeli_ks	Buffeli_kaks	mean_kaks	function
XP_009691323.1	0.123833	0.349276	0.354541	0.146679	0.674246	0.217546	0.286044	conserved hypothetical protein
XP_009691324.1	0.159969	0.59878	0.267159	0.193856	0.514312	0.376923	0.322041	conserved hypothetical protein
XP_009691325.1	0.0839205	0.78265	0.107226	0.0808435	0.508664	0.158933	0.133079	conserved hypothetical protein
XP_009691326.1	0.110525	0.434787	0.254204				0.254204	conserved hypothetical protein
XP_009691327.1	0.199543	0.610961	0.326605	0.206514	0.590767	0.34957	0.338087	conserved hypothetical protein
XP_009691328.1	0.119156	0.63463	0.187756	0.109276	0.733799	0.148919	0.168338	conserved hypothetical protein
XP_009691329.1	0.13276	0.54508	0.24356	0.112404	0.411423	0.273208	0.258384	conserved hypothetical protein
XP_009691330.1	0.0320281	0.750965	0.0426493	0.0265665	0.579761	0.0458232	0.044236	uncharacterized protein
XP_009691331.1	0.0626555	0.517162	0.121153	0.0711056	0.446523	0.159243	0.140198	conserved hypothetical protein
XP_009691332.1	0.0737034	0.357744	0.206022	0.0330129	0.303029	0.108943	0.157482	conserved hypothetical protein
XP_009691333.1	0.030778	0.842995	0.0365103	0.0127331	0.6133	0.0207616	0.028636	uncharacterized protein
XP_009691334.1	0.0581234	0.562692	0.103295	0.0585409	0.430201	0.136078	0.119687	uncharacterized protein
XP_009691335.1	0.0506903	0.516724	0.0980995	0.0541334	0.512447	0.105637	0.101868	predicted protein
XP_009691336.1	0.0391101	0.491914	0.0795059	0.0293095	0.678773	0.0431801	0.061343	geranylgeranyltransferase subunit beta
XP_009691337.1	0.110852	0.44254	0.250491	0.0649223	0.363827	0.178443	0.214467	conserved hypothetical protein
XP_009691338.1	0.0387165	0.448347	0.0863537	0.0263259	0.432757	0.0608331	0.073593	uncharacterized protein
XP_009691339.1	0.0181467	0.224473	0.0808411	0.0156662	0.328008	0.0477617	0.064301	uncharacterized protein
XP_009691340.1	0.105316	0.26979	0.390363	0.0777404	0.297265	0.261519	0.325941	conserved hypothetical protein
XP_009691341.1	0.0383332	0.511452	0.0749496	0.0318656	0.49876	0.0638897	0.06942	uncharacterized protein
XP_009691342.1	0.0426088	0.549281	0.0775719	0.024203	0.58011	0.0417214	0.059647	tRNA nucleotidyltransferase
XP_009691343.1	0.0546498	0.541705	0.100885	0.0421846	0.591447	0.0713244	0.086105	tRNA nucleotidyltransferase
XP_009691344.1	0.020327	0.408703	0.0497353				0.0497353	conserved hypothetical protein
XP_009691345.1	0.0847719	0.435102	0.194832	0.0582497	0.709404	0.0821108	0.138471	conserved hypothetical protein
XP_009691346.1	0.114724	0.474856	0.241597	0.122377	0.476082	0.257049	0.249323	palmitoyltransferase
XP_009691347.1	0.0154727	0.589951	0.0262271	0.0103188	0.439096	0.0235001	0.024864	CGMP-dependent protein kinase
XP_009691348.1	0.0232831	0.456586	0.0509939	0.0260341	0.472699	0.0550756	0.053035	acid phosphatase
XP_009691349.1	0.0245402	0.29754	0.0824768	0.0523414	0.418052	0.125203	0.10384	zinc transport protein
XP_009691350.1	0.0174421	0.309897	0.0562836	0.0159436	0.24236	0.065785	0.061034	50S ribosomal protein L3
XP_009691351.1	0.0164738	0.201292	0.0818402				0.0818402	DAZ-associated protein 1
XP_009691353.1	0.0470847	0.527146	0.08932	0.0393754	0.346709	0.113569	0.101444	conserved hypothetical protein
XP_009691354.1	0.114668	0.522786	0.21934	0.111522	0.477035	0.233782	0.226561	conserved hypothetical protein
XP_009691355.1	0.0590501	0.602828	0.0979551	0.0493012	0.586778	0.0840202	0.090988	uncharacterized protein
XP_009691356.1	0.0420734	0.718077	0.0585917	0.0329209	0.689709	0.0477316	0.053162	conserved hypothetical protein
XP_009691357.1	0.0469212	0.242701	0.193329	0.0502163	0.214458	0.234155	0.213742	conserved hypothetical protein
XP_009691358.1	0.0465551	0.270882	0.171865	0.0363811	0.257291	0.141401	0.156633	uncharacterized protein
XP_009691359.1	0.0914449	0.251191	0.364046	0.108519	0.253881	0.427442	0.395744	conserved hypothetical protein
XP_009691361.1	0.0714075	0.829715	0.0860628	0.0581586	0.96276	0.0604082	0.073235	conserved hypothetical protein
XP_009691363.1	0.0381096	0.439711	0.0866696	0.0474901	0.280018	0.169596	0.128133	uncharacterized protein
XP_009691364.1	0.0203441	0.869161	0.0234066	0.0187093	0.894862	0.0209075	0.022157	ribonucleoside-diphosphate reductase large chain
XP_009691365.1	0.0689377	0.563809	0.122271	0.0594144	0.434681	0.136685	0.129478	ATP-dependent Clp protease proteolytic subunit
XP_009691366.1	0.01472	0.250322	0.0588041				0.0588041	bis-(5'-nucleosyl)-[tri-or tetra-] phosphatase
XP_009691367.1	0.0343772	0.640885	0.0536402	0.0263518	0.626224	0.0420804	0.04786	chromosome condensation protein
XP_009691368.1	0.0150895	0.58791	0.0256663	0.0109535	0.605861	0.0180792	0.021873	glucose-6-phosphate/phosphate translocator
XP_009691369.1	0.0309475	0.587702	0.0526585	0.00826617	0.550306	0.0150211	0.03384	conserved hypothetical protein
XP_009691370.1	0.0812386	0.871974	0.0931663	0.0731918	0.761004	0.096178	0.094672	serine/threonine-protein kinase ripk4
XP_009691371.1	0.0317993	0.704699	0.0451247	0.0289556	0.646026	0.0448212	0.044973	NADH dehydrogenase
XP_009691372.1	0.00523061	0.568472	0.00920118	0.00888604	0.324163	0.0274123	0.018307	ubiquitin carrier protein
XP_009691373.1	0.101831	0.487163	0.209028	0.0798897	0.503964	0.158523	0.183775	major facilitator superfamily MFS-1 protein
XP_009691374.1	0.0144503	0.615624	0.0234726	0.00736202	0.4958	0.0148488	0.019161	uncharacterized protein
XP_009691375.1	0.0519833	0.570524	0.091115	0.0412765	0.708022	0.0582984	0.074707	major facilitator superfamily MFS-1 protein
XP_009691376.1	0.0641064	0.778936	0.0823	0.054714	0.694419	0.0787911	0.080546	major facilitator superfamily MFS-1 protein
XP_009691377.1	0.115575	0.735736	0.157088	0.0926135	0.660327	0.140254	0.148671	conserved hypothetical protein
XP_009691378.1	0.0502356	0.287559	0.174697	0.0576923	0.48257	0.119552	0.147124	uncharacterized protein

protein_id	Chitose_ka	Chitose_ks	Chitose_kaks	Buffeli_ka	Buffeli_ks	Buffeli_kaks	mean_kaks	function
XP_009691379.1	0.0417193	0.507142	0.0822636	0.0434437	0.707106	0.0614387	0.071851	uncharacterized protein
XP_009691380.1	0.0233518	0.594163	0.039302	0.0267291	0.496742	0.0538088	0.046555	DNA ligase 1 precursor
XP_009691381.1	0.0369111	0.427434	0.086355	0.0231944	0.417603	0.0555419	0.070948	conserved hypothetical protein
XP_009691382.1	0.106686	0.320217	0.333169	0.0967278	0.256079	0.377726	0.355448	conserved hypothetical protein
XP_009691383.1	0.121659	0.487694	0.249457	0.115951	0.436329	0.265743	0.2576	conserved hypothetical protein
XP_009691384.1	0.1729	0.556018	0.310961	0.126624	0.484705	0.26124	0.2861	conserved hypothetical protein
XP_009691385.1	0.0931869	0.614828	0.151566	0.0888143	0.633175	0.140268	0.145917	conserved hypothetical protein
XP_009691386.1	0.0976201	0.478519	0.204005	0.0934046	0.390426	0.239237	0.221621	conserved hypothetical protein
XP_009691387.1	0.0227801	0.777819	0.0292871	0.0233846	1.16078	0.0201456	0.024716	glucose-6-phosphate-1-dehydrogenase
XP_009691388.1	3.17509E-05	0.0317509	0.001	0.0197353	0.311171	0.0634227	0.032211	hypothetical protein
XP_009691389.1	0.0714435	0.343212	0.208161	0.0770298	0.369401	0.208527	0.208344	uncharacterized protein
XP_009691390.1	0.0354993	0.735138	0.0482892	0.0193837	0.50769	0.0381803	0.043235	cleavage and polyadenylation specificty factor subunit
XP_009691391.1	0.0474136	0.456694	0.103819	0.0441645	0.463057	0.095376	0.099598	cleavage and polyadenylation specificty factor subunit
XP_009691392.1	0.0787022	1.0452	0.0752985	0.0741162	0.888018	0.0834625	0.07938	uncharacterized protein
XP_009691393.1	0.113639	0.340031	0.334202	0.0861106	0.286339	0.30073	0.317466	hypothetical protein
XP_009691394.1	0.0788394	0.536926	0.146835	0.0716862	0.56609	0.126634	0.136734	conserved hypothetical protein
XP_009691395.1	0.0830424	0.504225	0.164693	0.0914709	0.448071	0.204144	0.184418	nucleolar protein
XP_009691396.1	0.0943433	0.166815	0.565556	0.0634004	0.0616014	1.0292	0.797378	conserved hypothetical protein
XP_009691397.1	0.0459079	1.11085	0.0413267	0.0367588	0.964708	0.0381035	0.039715	uncharacterized protein
XP_009691398.1	0.0820073	0.374727	0.218845	0.0617853	0.329328	0.18761	0.203228	DNA mismatch repair protein
XP_009691399.1	0.0188115	0.638146	0.0294784	0.0158904	0.529339	0.0300192	0.029749	cell division cycle CDC48 homologue/transitional endoplasmic reticulum ATPase
XP_009691400.1	0.0122982	0.489099	0.0251447	0.0184619	0.533274	0.03462	0.029882	aspartyl-tRNA synthetase
XP_009691401.1	0.0247232	0.234699	0.10534	0.00313694	0.106597	0.029428	0.067384	60S ribosomal protein L34
XP_009691402.1	0.0735791	0.335743	0.219153	0.0370429	0.268655	0.137883	0.178518	conserved hypothetical protein
XP_009691403.1	0.0768411	0.342016	0.224671	0.117889	0.238434	0.494428	0.359549	hypothetical protein
XP_009691404.1	0.023879	0.703532	0.0339416	0.033834	0.483921	0.0699164	0.051929	hypothetical protein
XP_009691405.1				4.51343E-05	0.0451343	0.001	0.001	hypothetical protein
XP_009691406.1	0.0937731	0.720408	0.130167	0.0672897	0.723183	0.0930465	0.111607	conserved hypothetical protein
XP_009691407.1	0.0354919	0.46562	0.076225	0.0337455	0.347699	0.0970538	0.086639	conserved hypothetical protein
XP_009691408.1	0.0493241	0.282784	0.174423	0.0187813	0.383342	0.0489936	0.111708	uncharacterized protein
XP_009691409.1	0.0308368	0.83892	0.0367577	0.0310535	0.953489	0.0325683	0.034663	clathrin heavy chain
XP_009691410.1	0.0415978	0.707166	0.0588233	0.0354175	0.635072	0.0557693	0.057296	P-type ATPase 2
XP_009691411.1	0.0425566	0.613128	0.069409	0.033367	0.599423	0.0556652	0.062537	DEAD-box family RNA helicase
XP_009691412.1	0.125941	0.290547	0.43346	0.078753	0.326482	0.241217	0.337338	conserved hypothetical protein
XP_009691413.1	0.0295101	0.374847	0.0787256	0.0214584	0.420446	0.0510372	0.064881	uncharacterized protein
XP_009691414.1	0.108316	0.468964	0.230969	0.114388	0.516804	0.221337	0.226153	uncharacterized protein
XP_009691415.1	0.0512615	0.447204	0.114627	0.0513749	0.488196	0.105234	0.109931	uncharacterized protein
XP_009691416.1	0.0941043	0.396243	0.237491	0.0417882	0.49063	0.0851726	0.161332	hypothetical protein
XP_009691417.1	0.0353572	0.539077	0.0655885	0.0204122	0.441505	0.0462332	0.055911	origin recognition complex protein 1
XP_009691418.1	NA	NA	NA	NA	NA	NA	NA	hypothetical protein
XP_009691419.1	0.0178826	0.180347	0.0991564	0.0170529	0.161064	0.105877	0.102517	high-mobility-group protein
XP_009691420.1	0.0270467	0.218466	0.123803	0.0206424	0.510052	0.0404712	0.082137	conserved hypothetical protein
XP_009691421.1	0.0372605	0.636407	0.0585482	0.0462709	0.641333	0.0721481	0.065348	uncharacterized protein
XP_009691422.1	0.0792329	0.92335	0.0858102	0.0661846	0.920203	0.0719239	0.078867	conserved hypothetical protein
XP_009691423.1	0.0571923	1.15981	0.0493117	0.0565576	0.994802	0.0568531	0.053082	uncharacterized protein
XP_009691424.1	0.0258621	0.494864	0.052261				0.052261	conserved hypothetical protein
XP_009691426.1	0.0311779	1.08966	0.0286124	0.0233634	1.34741	0.0173395	0.022976	adenosine monophosphate deaminase 2 (isoform L) isoform 3
XP_009691427.1	0.126218	0.35999	0.350616	0.179425	0.91782	0.19549	0.273053	conserved hypothetical protein
XP_009691428.1	0.106604	0.477257	0.223368	0.0982471	0.517047	0.190016	0.206692	conserved hypothetical protein
XP_009691429.1	0.0589837	0.645783	0.0913368	0.05259	0.531843	0.0988825	0.09511	conserved hypothetical protein
XP_009691430.1	0.0455959	0.488515	0.0933357	0.0336431	0.501481	0.0670874	0.080212	methionine aminopeptidase
XP_009691431.1	0.0661999	0.702706	0.0942072	0.0646384	0.586417	0.110226	0.102217	uncharacterized protein
XP_009691432.1	0.0776233	0.220842	0.351488	0.136651	0.231075	0.591369	0.471429	hypothetical protein

protein_id	Chitose_ka	Chitose_ks	Chitose_kaks	Buffeli_ka	Buffeli_ks	Buffeli_kaks	mean_kaks	function
XP_009691433.1	0.01984	0.522544	0.037968	0.022414	0.555458	0.0403523	0.03916	conserved hypothetical protein
XP_009691434.1	0.0809474	0.362449	0.223335	0.08256	0.413901	0.199468	0.211402	conserved hypothetical protein
XP_009691435.1	0.0227749	0.406341	0.0560488	0.0256441	0.199675	0.128429	0.092239	conserved hypothetical protein
XP_009691436.1	0.0311486	0.126368	0.246491	0.0470024	0.259126	0.181388	0.21394	deaminase
XP_009691437.1	0.0522333	0.41067	0.127191	0.040599	0.403114	0.100714	0.113952	conserved hypothetical protein
XP_009691438.1				NA	NA	NA	NA	hypothetical protein
XP_009691439.1	0.0493242	0.484699	0.101763	0.0468121	0.622355	0.0752177	0.08849	cactin
XP_009691440.1	0.0380453	0.518194	0.073419	0.0419664	0.522434	0.0803285	0.076874	uncharacterized protein
XP_009691441.1	0.0550807	0.918418	0.0599734	0.0340609	0.718542	0.0474028	0.053688	homeodomain-like containing protein
XP_009691442.1	0.0587593	0.522377	0.112484	0.0657513	0.620583	0.105951	0.109217	uncharacterized protein
XP_009691443.1	0.0976049	0.376459	0.259271	0.092757	0.411444	0.225442	0.242356	conserved hypothetical protein
XP_009691444.1	0.000313136	0.313136	0.001	0.000186445	0.186445	0.001	0.001	ubiquitin carrier protein
XP_009691445.1	0.0170968	0.430543	0.0397099	0.00417589	0.607288	0.0068763	0.023293	nucleosome assembly protein
XP_009691446.1	0.018263	0.552298	0.0330674	0.0141809	0.676421	0.0209646	0.027016	proteasome subunit
XP_009691447.1	0.00557892	0.378469	0.0147407	0.0057026	0.258423	0.0220669	0.018404	proliferating cell nuclear antigen 1
XP_009691448.1	0.0263206	0.26201	0.100457	0.0435231	0.363703	0.119666	0.110062	uncharacterized protein
XP_009691449.1	0.0138716	0.393056	0.0352917	0.0113156	0.488026	0.0231864	0.029239	DNA helicase
XP_009691450.1	0.0131383	1.01265	0.0129742	0.0177756	0.91163	0.0194987	0.016236	conserved hypothetical protein
XP_009691451.1	0.0626606	0.744986	0.0841098	0.0424403	0.767034	0.0553304	0.06972	conserved hypothetical protein
XP_009691452.1	NA	NA	NA				NA	conserved hypothetical protein
XP_009691453.1	0.0971098	0.504543	0.192471	0.0999099	0.490245	0.203796	0.198134	conserved hypothetical protein
XP_009691454.1	0.0667608	0.574382	0.116231	0.0647994	0.761295	0.0851173	0.100674	conserved hypothetical protein
XP_009691457.1	0.0115958	0.598465	0.0193759	0.0127991	0.555845	0.0230264	0.021201	spliceosome-associated protein
XP_009691458.1	0.0581964	0.610446	0.0953343	0.0494985	0.537721	0.0920524	0.093693	uncharacterized protein
XP_009691459.1	0.0460988	0.529437	0.0870714	0.0419876	0.581303	0.0722302	0.079651	DEAD-box family RNA helicase
XP_009691460.1	0.036587	0.499089	0.0733075	0.00694154	0.324872	0.021367	0.047337	replication factor
XP_009691461.1	0.016037	0.121335	0.132171	0.0146838	0.122981	0.119399	0.125785	mitochondrial carrier protein
XP_009691462.1	0.0391268	0.422021	0.092713	0.0479929	0.397606	0.120705	0.106709	uncharacterized protein
XP_009691463.1	0.00516903	0.440321	0.0117392	0.0106651	0.307831	0.0346458	0.023192	U3 small nucleolar ribonucleoprotein/40S ribosomal protein S4-like
XP_009691464.1	0.000581681	0.581681	0.001	0.000382178	0.382178	0.001	0.001	40S ribosomal protein S28
XP_009691465.1	0.0605383	0.447452	0.135296	0.0553276	0.719065	0.0769439	0.10612	phosphatidylinositol-4-phosphate 5-kinase
XP_009691466.1	0.0702592	0.620399	0.113249	0.070036	0.624364	0.112172	0.11271	nucleotide binding protein 2
XP_009691467.1	0.0283929	0.35634	0.0796794	0.0199839	0.283336	0.0705306	0.075105	DNA primase large subunit
XP_009691468.1	0.0925102	0.289167	0.31992	0.0595609	0.264586	0.225109	0.272514	uncharacterized protein
XP_009691469.1	0.0435888	0.309749	0.140723	0.0293939	0.206668	0.142228	0.141475	dehydrodolichyl diphosphate synthase
XP_009691470.1	0.0297397	0.972277	0.0305877	0.0293087	0.7788	0.0376332	0.03411	protein disulphide isomerase
XP_009691471.1	0.0424175	0.501058	0.0846558	0.0481936	0.609933	0.0790145	0.081835	uncharacterized protein
XP_009691472.1	0.00514992	0.362985	0.0141877	0.000363902	0.363902	0.001	0.007594	U6 snRNA-associated sm-like protein
XP_009691473.1	0.0525059	0.61564	0.0852867	0.0527388	0.412686	0.127794	0.10654	conserved hypothetical protein
XP_009691474.1	0.106396	0.521955	0.203841	0.081905	0.372719	0.21975	0.211795	uncharacterized protein
XP_009691475.1	0.0454742	0.644145	0.0705962	0.0246973	0.489874	0.0504157	0.060506	conserved hypothetical protein
XP_009691476.1	0.150093	0.124222	1.20826				1.20826	hypothetical protein
XP_009691477.1	0.126335	0.831439	0.151947	0.110445	0.572975	0.192757	0.172352	uncharacterized protein
XP_009691478.1	0.0368581	0.572052	0.0644313	0.0499604	0.42994	0.116203	0.090317	uncharacterized protein
XP_009691479.1	0.0179721	0.478829	0.0375335	0.025629	0.402597	0.0636594	0.050596	mitochondrial import inner membrane translocase subunit
XP_009691480.1	0.0724627	0.562858	0.128741	0.0622209	0.557755	0.111556	0.120148	uncharacterized protein
XP_009691481.1	0.0516981	0.743002	0.0695801	0.0418502	0.68227	0.0613397	0.06546	neutral sphingomyelinase
XP_009691482.1	0.0273241	0.45001	0.0607189	0.0163001	0.520246	0.0313316	0.046025	uncharacterized protein
XP_009691483.1	0.00166931	0.558652	0.0029881	0.0052922	0.428086	0.0123625	0.007675	prohibitin
XP_009691484.1	0.0195845	0.389483	0.0502834	0.0133022	0.297977	0.0446415	0.047462	uncharacterized protein
XP_009691485.1	0.0984979	0.428372	0.229935	0.0858648	0.32403	0.26499	0.247463	conserved hypothetical protein
XP_009691486.1	NA	NA	NA				NA	hypothetical protein
XP_009691487.1	0.031014	0.363166	0.0853989				0.0853989	uncharacterized protein

protein_id	Chitose_ka	Chitose_ks	Chitose_kaks	Buffeli_ka	Buffeli_ks	Buffeli_kaks	mean_kaks	function
XP_009691488.1	0.0548403	0.545629	0.100508	0.0529142	0.518659	0.102021	0.101265	pentatricopeptide repeat containing protein
XP_009691489.1	0.0382596	1.39101	0.0275049	0.0270551	1.65752	0.0163226	0.021914	conserved hypothetical protein
XP_009691490.1	0.0201652	0.448925	0.0449189	0.000313823	0.313823	0.001	0.022959	RNA processing factor
XP_009691491.1	0.0421904	0.902718	0.0467371	0.0367425	0.855404	0.0429535	0.044845	RNA processing factor
XP_009691492.1	0.0418194	0.731279	0.0571867	0.0390261	0.776578	0.0502539	0.05372	uncharacterized protein
XP_009691493.1	0.0147141	0.276196	0.0532742				0.0532742	conserved hypothetical protein
XP_009691494.1	0.0568247	0.633289	0.0897296	0.0451337	0.627051	0.0719777	0.080854	uncharacterized protein
XP_009691495.1	0.0344816	0.77271	0.0446242	0.0333383	0.800369	0.0416536	0.043139	DEAD-box family helicase
XP_009691496.1	0.068339	0.564287	0.121107	0.0807974	0.594596	0.135886	0.128497	conserved hypothetical protein
XP_009691498.1	0.0588872	0.47872	0.12301	0.0369606	0.441495	0.0837169	0.103363	conserved hypothetical protein
XP_009691499.1	0.0574985	0.424426	0.135473	0.0482399	0.461605	0.104505	0.119989	conserved hypothetical protein
XP_009691500.1	0.133803	0.506863	0.263982	0.0837118	0.622647	0.134445	0.199213	conserved hypothetical protein
XP_009691501.1	0.0592519	0.428625	0.138237	0.024258	0.28094	0.0863459	0.112291	conserved hypothetical protein
XP_009691502.1	0.016722	0.396991	0.042122	0.0143418	0.485628	0.0295326	0.035827	lysine-tRNA ligase
XP_009691504.1	0.12313	0.449822	0.273731	0.146667	0.500485	0.29305	0.28339	uncharacterized protein
XP_009691505.1	0.0581593	0.697984	0.0833246	0.0435723	0.627729	0.0694126	0.076369	conserved hypothetical protein
XP_009691506.1	0.0618705	0.626046	0.0988275	0.0532443	0.549907	0.0968243	0.097826	MUS81 endonuclease homolog
XP_009691507.1	0.0226195	0.490601	0.0461057	0.00672881	0.468019	0.0143772	0.030241	syntaxin
XP_009691508.1	0.0529834	0.447925	0.118286	0.0447802	0.385447	0.116177	0.117232	conserved hypothetical protein
XP_009691509.1	0.0978433	0.515172	0.189923	0.0916358	0.4899	0.18705	0.188487	conserved hypothetical protein
XP_009691510.1	0.0587523	0.817614	0.0718582	0.0509064	0.435372	0.116926	0.094392	conserved hypothetical protein
XP_009691512.1	0.0336968	0.821423	0.0410224	0.0278131	0.71872	0.0386981	0.03986	conserved hypothetical protein
XP_009691513.1	0.0128254	1.15086	0.0111442	0.00826844	1.02653	0.00805477	0.009599	transcription factor IIIb subunit
XP_009691514.1	0.0403491	0.876818	0.0460177	0.0444041	0.811504	0.0547183	0.050368	ion-translocating ATPase
XP_009691515.1	0.0388411	0.305231	0.127251	0.0243796	0.378762	0.0643664	0.095809	membrane transporter
XP_009691516.1	0.00812774	1.19377	0.00680849	0.00555175	0.932051	0.00595649	0.006382	DEAD-box family helicase
XP_009691517.1	0.0210828	0.618447	0.0340899	0.0197669	0.490298	0.040316	0.037203	uncharacterized protein
XP_009691518.1	0.000289264	0.289264	0.001	0.0101386	0.182139	0.0556642	0.028332	60S ribosomal protein L37a
XP_009691519.1	0.0120289	0.381966	0.031492	0.000222776	0.222776	0.001	0.016246	protein translocation complex subunit gamma chain
XP_009691520.1	0.0483574	0.953142	0.0507347	0.03574	0.769939	0.0464193	0.048577	uncharacterized protein
XP_009691521.1	0.0614517	0.136599	0.449871				0.449871	conserved hypothetical protein
XP_009691523.1	0.0655498	0.561893	0.116659	0.0664565	0.477893	0.139062	0.12786	conserved hypothetical protein
XP_009691524.1	0.024514	0.616199	0.0397827	0.0152986	0.609087	0.0251172	0.03245	uncharacterized protein
XP_009691525.1	0.0886932	0.806848	0.109925	0.0818154	1.08551	0.0753705	0.092648	2-C-methyl-D-erythritol 2%2C4-cyclodiphosphate synthase
XP_009691526.1	0.0285568	0.620466	0.0460248	0.0295939	0.515151	0.057447	0.051736	conserved hypothetical protein
XP_009691527.1	0.0406697	0.296742	0.137054	0.0157981	0.153818	0.102706	0.11988	conserved hypothetical protein
XP_009691528.1	0.0670733	0.662534	0.101238	0.0716278	0.573733	0.124845	0.113041	uncharacterized protein
XP_009691529.1	0.109026	0.529103	0.206058	0.104848	0.515088	0.203554	0.204806	conserved hypothetical protein
XP_009691530.1	0.0210565	0.474289	0.0443959	0.0295738	0.40653	0.0727469	0.058571	mitochondrial carrier protein
XP_009691531.1	0.0220628	0.224543	0.0982565	0.0249537	0.184826	0.135012	0.116634	conserved hypothetical protein
XP_009691532.1	0.0612615	0.357452	0.171384	0.0546486	0.27576	0.198175	0.184779	conserved hypothetical protein
XP_009691533.1	0.0535623	0.499497	0.107232	0.0455101	0.393093	0.115774	0.111503	cysteine proteinase
XP_009691534.1	0.0501831	1.28079	0.0391814	0.0188669	0.681723	0.0276753	0.033428	conserved hypothetical protein
XP_009691535.1	0.0200992	0.472002	0.0425829				0.0425829	conserved hypothetical protein
XP_009691536.1	0.0148792	0.652845	0.0227913				0.0227913	conserved hypothetical protein
XP_009691537.1	0.021208	0.21449	0.0988764				0.0988764	conserved hypothetical protein
XP_009691538.1	0.118451	0.617729	0.191752	0.102417	0.509868	0.200869	0.19631	conserved hypothetical protein
XP_009691539.1	0.0495069	0.211778	0.233767				0.233767	conserved hypothetical protein
XP_009691540.1	0.0576813	0.603019	0.0956542	0.000243186	0.243186	0.001	0.048327	peptidyl-prolyl cis-trans isomerase
XP_009691541.1	0.038203	0.431274	0.0885818	0.0614887	0.5609	0.109625	0.099103	uncharacterized protein
XP_009691542.1	0.0870869	0.59727	0.145808	0.0807501	0.630724	0.128028	0.136918	uncharacterized protein
XP_009691543.1	0.0155051	0.663571	0.0233661	0.00910291	0.58729	0.0154999	0.019433	uncharacterized protein
XP_009691544.1	0.0844202	0.885244	0.0953637	0.081573	0.936063	0.0871448	0.091254	uncharacterized protein

protein_id	Chitose_ka	Chitose_ks	Chitose_kaks	Buffeli_ka	Buffeli_ks	Buffeli_kaks	mean_kaks	function
XP_009691545.1	0.0231233	0.444951	0.0519682	0.0206698	0.430418	0.0480227	0.049995	glucose-6-phosphate isomerase
XP_009691546.1	0.12491	0.672003	0.185877	0.116042	0.48287	0.240317	0.213097	hypothetical protein
XP_009691547.1	0.0381687	0.373362	0.10223	0.0423654	0.366068	0.115731	0.108981	conserved hypothetical protein
XP_009691548.1	0.0299034	0.339454	0.0880928	0.0149287	0.371661	0.0401676	0.06413	ER lumen protein retaining receptor 1
XP_009691549.1	0.0468975	0.549644	0.0853235	0.0224741	0.476605	0.0471545	0.066239	uncharacterized protein
XP_009691550.1	0.15475	0.526785	0.293762				0.293762	major facilitator superfamily MFS-1 protein
XP_009691551.1	0.0378041	0.900756	0.0419693	0.029492	0.986101	0.0299077	0.035938	integral membrane protein
XP_009691552.1	0.0725651	0.483952	0.149943	0.0406585	0.353166	0.115126	0.132534	integral membrane protein
XP_009691553.1	0.0425122	0.347997	0.122162	0.0335182	0.37994	0.0882198	0.105191	integral membrane protein
XP_009691555.1	0.0169507	0.202486	0.0837128				0.0837128	uncharacterized protein
XP_009691556.1	0.0738234	0.853523	0.0864926	0.0575112	1.71353	0.0335631	0.060028	conserved hypothetical protein
XP_009691557.1	0.0762081	0.381006	0.200018	0.11033	0.49452	0.223106	0.211562	peptidyl-prolyl cis-trans isomerase
XP_009691558.1	0.0261237	0.403132	0.0648019	0.0176879	0.383667	0.0461023	0.055452	ubiquitin carrier protein
XP_009691559.1	0.0826137	0.595029	0.13884	0.0842763	0.485173	0.173704	0.156272	uncharacterized protein
XP_009691560.1	0.0467993	0.872812	0.0536191	0.0424618	0.878777	0.0483192	0.050969	uncharacterized protein
XP_009691561.1	0.0413421	0.518824	0.0796843	0.0424546	0.469841	0.0903595	0.085022	uncharacterized protein
XP_009691562.1	0.0888173	0.527367	0.168416	0.0315136	0.423386	0.0744323	0.121424	conserved hypothetical protein
XP_009691563.1	0.0221314	0.291593	0.0758983	0.0194512	0.297983	0.0652763	0.070587	50S ribosomal protein L12
XP_009691564.1	0.0836136	0.425996	0.196278	0.0811701	0.445703	0.182117	0.189198	conserved hypothetical protein
XP_009691565.1				0.00948096	0.189577	0.0500111	0.0500111	conserved hypothetical protein
XP_009691567.1	0.0582408	0.415981	0.140008	0.0325107	0.187257	0.173616	0.156812	uncharacterized protein
XP_009691568.1	0.0814882	0.447286	0.182184	0.077346	0.570266	0.135631	0.158908	uncharacterized protein
XP_009691569.1	0.0426897	0.493929	0.0864288	0.0346085	0.474526	0.0729328	0.079681	uncharacterized protein
XP_009691570.1	0.0750147	0.410866	0.182577	0.0524235	0.299124	0.175257	0.178917	uncharacterized protein
XP_009691571.1	0.0355609	0.563862	0.0630666	0.0469495	0.371124	0.126506	0.094786	conserved hypothetical protein
XP_009691572.1	0.0274411	0.38315	0.0716197	0.0333832	0.344346	0.0969467	0.084283	uncharacterized protein
XP_009691573.1	0.000472521	0.472521	0.001	0.000500368	0.500368	0.001	0.001	casein kinase
XP_009691574.1	0.0721314	0.539137	0.13379	0.0504335	0.463332	0.10885	0.12132	chromosome segregation protein
XP_009691575.1	0.0870432	0.413394	0.210558	0.0963384	0.360288	0.267393	0.238976	hypothetical protein
XP_009691576.1	0.123164	0.355808	0.346154	0.0957664	0.296868	0.322589	0.334372	hypothetical protein
XP_009691577.1	0.097878	0.40725	0.240339	0.0902059	0.35433	0.254582	0.24746	conserved hypothetical protein
XP_009691578.1	0.0434591	0.196471	0.221198	0.037592	0.194474	0.193301	0.20725	uncharacterized protein
XP_009691579.1	0.0650843	0.489829	0.132871	0.0498791	0.556149	0.0896864	0.111279	uncharacterized protein
XP_009691580.1	0.0370062	0.343303	0.107795	0.0248113	0.281912	0.0880109	0.097903	pepsinogen
XP_009691581.1	0.13789	0.52699	0.261655	0.118508	0.486808	0.243439	0.252547	conserved hypothetical protein
XP_009691582.1	0.0400444	0.395569	0.101232	0.0441903	0.460878	0.0958827	0.098557	uncharacterized protein
XP_009691583.1	0.042147	0.371916	0.113324	0.0272375	0.251608	0.108254	0.110789	uncharacterized protein
XP_009691584.1	0.0477939	0.601561	0.0794497				0.0794497	uncharacterized protein
XP_009691585.1	0.0378392	0.618046	0.0612239	0.0262975	0.751537	0.0349916	0.048108	uncharacterized protein
XP_009691586.1	0.0100617	0.328873	0.0305945	NA	NA	NA	NA	conserved hypothetical protein
XP_009691588.1	0.0822992	0.629503	0.130737	0.0708856	0.614896	0.115281	0.123009	uncharacterized protein
XP_009691589.1	0.0408853	0.490484	0.0833571	0.0471714	0.45413	0.103872	0.093615	conserved hypothetical protein
XP_009691590.1	0.000124236	0.124236	0.001				0.001	U2 snRNP auxiliary factor
XP_009691591.1	0.019577	0.259413	0.0754665	0.00688893	0.265242	0.0259722	0.050719	proteasome subunit alpha type
XP_009691592.1	0.011951	0.910995	0.0131186	0.0163468	1.00169	0.0163192	0.014719	Prp8 protein
XP_009691593.1	0.0849137	0.436261	0.19464	0.0909131	0.704113	0.129117	0.161879	conserved hypothetical protein
XP_009691594.1	0.0594172	0.498378	0.119221	0.0573908	0.348637	0.164615	0.141918	conserved hypothetical protein
XP_009691595.1	0.088127	0.60086	0.146668	0.0761353	0.49732	0.153091	0.149879	uncharacterized protein
XP_009691596.1	0.0666212	0.629185	0.105885	0.0743023	0.572664	0.129749	0.117817	uncharacterized protein
XP_009691598.1	NA	NA	NA				NA	conserved hypothetical protein
XP_009691599.1	0.0424669	0.54789	0.0775098	0.0427219	0.956013	0.0446876	0.061099	cysteine protease precursor TacP
XP_009691600.1	0.0898749	0.798863	0.112504	0.119666	0.830445	0.144098	0.128301	uncharacterized protein
XP_009691601.1	0.0815361	0.981415	0.0830801	0.0963316	1.07783	0.0893756	0.086228	cysteine proteinase precursor

protein_id	Chitose_ka	Chitose_ks	Chitose_kaks	Buffeli_ka	Buffeli_ks	Buffeli_kaks	mean_kaks	function
XP_009691602.1	0.0652605	0.890767	0.0732633	0.0572037	0.884403	0.0646806	0.068972	cysteine protease precursor TacP
XP_009691603.1	0.0310372	0.554933	0.0559297	0.0291904	0.595604	0.0490098	0.05247	Tocp1 variant tocp4
XP_009691604.1				0.0216325	0.0789125	0.274133	0.274133	hypothetical protein
XP_009691605.1	0.0660642	0.843663	0.0783065	0.0628687	0.872523	0.0720539	0.07518	cysteine protease precursor TacP
XP_009691606.1	0.0252977	0.470757	0.0537383	0.0220757	0.4499	0.0490681	0.051403	uncharacterized protein
XP_009691608.1	0.0452455	0.180582	0.250554	0.0382159	0.212462	0.179872	0.215213	conserved hypothetical protein
XP_009691609.1	0.0425726	0.268109	0.158788	0.0343594	0.224461	0.153075	0.155932	eukaryotic initiation factor
XP_009691610.1	0.0643595	0.421777	0.152591	0.046496	0.483344	0.0961965	0.124394	uncharacterized protein
XP_009691611.1	0.0364702	0.509867	0.0715288	0.0273349	0.48068	0.0568671	0.064198	conserved hypothetical protein
XP_009691612.1	0.0542114	0.388789	0.139436	0.0176072	0.436772	0.0403121	0.089874	conserved hypothetical protein
XP_009691613.1	0.0421661	0.705912	0.0597329	0.0320736	0.669005	0.0479423	0.053838	uncharacterized protein
XP_009691614.1	0.0713513	0.48899	0.145916	0.0507477	0.432547	0.117323	0.13162	uncharacterized protein
XP_009691615.1	0.0150123	0.46784	0.0320885	0.00834059	0.38549	0.0216363	0.026862	50S ribosomal protein L2
XP_009691616.1	0.0842046	0.454102	0.185431	0.0980306	0.471593	0.207871	0.196651	uncharacterized protein
XP_009691617.1	0.0307372	0.461568	0.0665931	0.0218306	0.568277	0.0384154	0.052504	pre-mRNA processing factor
XP_009691618.1	0.133058	0.613115	0.21702	0.13334	0.711473	0.187414	0.202217	conserved hypothetical protein
XP_009691619.1	0.0184755	0.190392	0.0970393	0.0138106	0.122873	0.112398	0.104719	conserved hypothetical protein
XP_009691620.1	0.0219412	0.29633	0.0740432	0.0235018	0.3288	0.0714776	0.07276	bifunctional nuclease
XP_009691621.1	0.214199	0.556227	0.385092	0.185176	0.572687	0.323346	0.354219	conserved hypothetical protein
XP_009691622.1	0.0224722	0.542511	0.0414225	0.0518662	0.53376	0.0971713	0.069297	conserved hypothetical protein
XP_009691623.1	0.0230621	0.272907	0.0845053	0.0250398	0.208857	0.11989	0.102198	uncharacterized protein
XP_009691624.1	0.112895	0.523459	0.215672	0.114703	0.558427	0.205403	0.210537	uncharacterized protein
XP_009691625.1	0.0770557	0.423939	0.181761	0.0508702	0.547475	0.0929178	0.137339	conserved hypothetical protein
XP_009691626.1	0.044631	0.44218	0.100934	0.0273775	0.43271	0.0632698	0.082102	uncharacterized protein
XP_009691627.1	0.0261062	0.513864	0.0508037	0.0220676	0.34002	0.0649009	0.057852	conserved hypothetical protein
XP_009691628.1	0.0289196	0.71039	0.0407094	0.0138065	0.537301	0.025696	0.033203	uncharacterized protein
XP_009691629.1	0.0487736	0.497889	0.0979607	0.0514855	0.487842	0.105537	0.101749	conserved hypothetical protein
XP_009691630.1	0.0689226	0.556287	0.123897	0.0737952	0.447047	0.165073	0.144485	uncharacterized protein
XP_009691631.1	0.0666264	0.489755	0.13604	0.0644708	0.415443	0.155186	0.145613	uncharacterized protein
XP_009691632.1	0.0675882	0.338079	0.199918	0.05084	0.283131	0.179564	0.189741	conserved hypothetical protein
XP_009691633.1	0.0368599	0.552073	0.0667665	0.0440591	0.519909	0.0847438	0.075755	uncharacterized protein
XP_009691634.1	0.0586125	0.430295	0.136215	0.0573925	0.409525	0.140144	0.13818	Conserved hypothetical protein
XP_009691635.1	0.114768	0.561612	0.204355	0.108096	0.630977	0.171315	0.187835	Conserved hypothetical protein
XP_009691636.1	0.112996	0.533485	0.211808	0.0672751	0.332745	0.202182	0.206995	uncharacterized protein
XP_009691637.1	0.0891538	0.337318	0.264302	0.071615	0.288861	0.247922	0.256112	conserved hypothetical protein
XP_009691638.1	0.0651094	0.353247	0.184317	0.0685773	0.374451	0.183141	0.183729	conserved hypothetical protein
XP_009691639.1	0.0974674	0.331806	0.293748	0.0427491	0.258176	0.165582	0.229665	Box C/D snoRNA protein
XP_009691640.1	0.0743637	0.471832	0.157606	0.066378	0.445664	0.148942	0.153274	uncharacterized protein
XP_009691641.1	0.0673847	0.293164	0.229853				0.229853	conserved hypothetical protein
XP_009691642.1	0.0444661	0.285386	0.15581	0.061063	0.273735	0.223073	0.189441	hypothetical protein
XP_009691643.1	0.0569058	0.394852	0.144119	0.0423158	0.408388	0.103617	0.123868	uncharacterized protein
XP_009691644.1	0.00976323	0.0488629	0.199809				0.199809	uncharacterized protein
XP_009691645.1	0.0625921	0.701626	0.0892101	0.0717502	0.812792	0.0882762	0.088743	uncharacterized protein
XP_009691646.1	0.154257	0.786529	0.196124	0.156115	0.819556	0.190487	0.193305	hypothetical protein
XP_009691647.1	0.0314377	0.561887	0.0559502	0.0211752	0.353177	0.0599564	0.057953	DNA replication licensing factor
XP_009691648.1	0.014811	0.457406	0.0323805	0.0132516	0.442854	0.0299232	0.031152	uncharacterized protein
XP_009691649.1	0.0920307	0.957209	0.0961448	0.0918662	1.34208	0.0684508	0.082298	uncharacterized protein
XP_009691650.1	0.0925663	0.326675	0.283359				0.283359	conserved hypothetical protein
XP_009691651.1	0.0163485	0.324236	0.0504216	0.0120794	0.291585	0.0414266	0.045924	uncharacterized protein
XP_009691652.1	0.032223	0.693018	0.0464966	0.0262141	0.66402	0.0394779	0.042987	seryl-tRNA synthetase
XP_009691653.1	0.0452834	0.553656	0.0817898	0.0455194	0.56395	0.0807153	0.081253	RNA 3'-terminal phosphate cyclase
XP_009691654.1	0.0303828	0.519689	0.0584635	0.0296245	0.381475	0.0776577	0.068061	succinate dehydrogenase subunit
XP_009691655.1	0.00942413	0.152703	0.0617155	0.0100126	0.0718149	0.139422	0.100569	50S ribosomal protein L27

protein_id	Chitose_ka	Chitose_ks	Chitose_kaks	Buffeli_ka	Buffeli_ks	Buffeli_kaks	mean_kaks	function
XP_009691656.1	0.0268259	0.486819	0.0551044	0.0207286	0.437444	0.0473857	0.051245	dihydrolipoyl dehydrogenase
XP_009691658.1	0.00320041	0.455062	0.00703291	6.51864E-05	0.0651864	0.001	0.004016	sulfur metabolism negative regulator
XP_009691659.1	0.0642851	0.59515	0.108015	0.046354	0.549659	0.0843324	0.096174	conserved hypothetical protein
XP_009691660.1	0.0306795	0.441562	0.0694796				0.0694796	uncharacterized protein
XP_009691661.1	0.0382066	0.347607	0.109913	0.00979522	0.35661	0.0274676	0.06869	60S ribosomal protein L24
XP_009691662.1	0.0450202	0.41723	0.107903	0.0455139	0.459927	0.0989589	0.103431	uncharacterized protein
XP_009691663.1	0.0414188	0.153774	0.269348				0.269348	conserved hypothetical protein
XP_009691669.1	0.0301455	0.541566	0.0556637	0.0222305	0.398687	0.0557595	0.055712	inosine-5'-monophosphate dehydrogenase
XP_009691671.1	0.115381	0.57538	0.200531				0.200531	hypothetical protein
XP_009691672.1	0.0415112	0.381484	0.108815	0.0433448	0.429392	0.100945	0.10488	uncharacterized protein
XP_009691673.1	0.0200352	0.381127	0.0525682	0.00889239	0.314196	0.028302	0.040435	uncharacterized protein
XP_009691674.1	0.023576	0.289869	0.0813332	0.0373451	0.198249	0.188375	0.134854	conserved hypothetical protein
XP_009691676.1	0.0055134	1.0362	0.00532077	0.00515767	0.792967	0.00650426	0.005913	splicing factor subunit
XP_009691677.1	0.000216539	0.216539	0.001				0.001	conserved hypothetical protein
XP_009691678.1	0.0303309	0.353	0.0859232	0.0300573	0.331342	0.0907139	0.088319	T-complex protein 1 subunit
XP_009691679.1	0.0932895	0.384568	0.242582	0.0822606	0.377302	0.218023	0.230302	conserved hypothetical protein
XP_009691680.1	0.047603	0.411084	0.115799	0.0334212	0.40976	0.0815629	0.098681	uncharacterized protein
XP_009691681.1	0.134748	0.495992	0.271673	0.108925	0.400151	0.272209	0.271941	uncharacterized protein
XP_009691682.1	0.184143	0.447204	0.411765	0.0959919	0.439737	0.218294	0.315029	conserved hypothetical protein
XP_009691683.1	0.0598213	0.381836	0.156668	0.0515959	0.334795	0.154112	0.15539	uncharacterized protein
XP_009691684.1	0.0600078	0.340926	0.176014	0.103833	0.545853	0.190222	0.183118	conserved hypothetical protein
XP_009691685.1	0.017585	0.675997	0.0260134	0.0143809	0.504305	0.0285162	0.027265	chaperonin CPN60
XP_009691686.1	0.0230049	0.340353	0.0675913				0.0675913	uncharacterized protein
XP_009691687.1	0.0606749	0.425202	0.142697	0.0680356	0.390702	0.174137	0.158417	uncharacterized protein
XP_009691688.1	0.0650813	0.446942	0.145615	0.0478121	0.250441	0.190912	0.168264	Fasciclin-2-like protein
XP_009691689.1	0.0300638	0.594355	0.0505822	0.0156921	0.502601	0.0312217	0.040902	DNA polymerase
XP_009691690.1	0.0196164	0.652025	0.0300854	0.0152413	0.459853	0.0331438	0.031615	snRNP splicing factor U2AF
XP_009691691.1	0.0605267	0.56689	0.10677	0.055608	0.521111	0.106711	0.106741	phospholipase
XP_009691692.1	0.0406403	0.389539	0.104329	0.0241159	0.439494	0.054872	0.079601	DNA-directed RNA polymerase D subunit
XP_009691693.1	0.0544338	0.331047	0.164429				0.164429	uncharacterized protein
XP_009691695.1	0.110917	0.625728	0.177261	0.136707	0.645037	0.211936	0.194599	conserved hypothetical protein
XP_009691696.1	0.0693195	0.602259	0.115099	0.0750677	0.878186	0.0854804	0.10029	conserved hypothetical protein
XP_009691697.1	0.0328977	0.642108	0.051234	0.0316853	0.673462	0.0470483	0.049141	ATP-dependent RNA helicase
XP_009691698.1	0.0835789	0.460541	0.18148	0.0698688	0.351448	0.198803	0.190142	translation initiation factor
XP_009691699.1	0.0489439	0.538301	0.090923	0.0399385	0.680536	0.0586868	0.074805	plastid 50S ribosomal protein L21
XP_009691700.1	0.0382964	0.247113	0.154975	0.0292248	0.110836	0.263675	0.209325	conserved hypothetical protein
XP_009691701.1	0.0394744	0.332212	0.118823	0.0246719	0.40338	0.0611629	0.089993	conserved hypothetical protein
XP_009691702.1	0.111157	0.43046	0.258229	0.145577	0.510449	0.285195	0.271712	conserved hypothetical protein
XP_009691703.1	0.0275257	0.395321	0.0696286	0.0286183	0.206746	0.138422	0.104025	Band 7-related protein
XP_009691704.1	0.00598728	1.01074	0.00592368	0.00504882	0.830456	0.00607957	0.006002	DNA-directed RNA polymerase
XP_009691705.1	0.0546964	0.434001	0.126028	0.0322896	0.45783	0.0705274	0.098278	uncharacterized protein
XP_009691706.1	0.0177417	0.42075	0.0421668	0.0187138	0.283661	0.0659724	0.05407	T-complex protein 1 alpha subunit
XP_009691707.1	0.120136	0.534923	0.224586	0.0904998	0.361876	0.250085	0.237336	conserved hypothetical protein
XP_009691708.1	0.150894	0.852551	0.176991	0.126845	0.771846	0.16434	0.170666	conserved hypothetical protein
XP_009691709.1	0.0570479	0.360701	0.158159	0.0551558	0.326196	0.169088	0.163623	conserved hypothetical protein
XP_009691710.1	0.150107	0.13201	1.13709				1.13709	hypothetical protein
XP_009691711.1	0.0784953	0.588707	0.133335	0.0734446	0.681178	0.10782	0.120578	ankyrin repeat containing protein
XP_009691713.1	0.0430582	0.866979	0.0496646	0.0279694	0.614347	0.045527	0.047596	adenylyl-cyclase-associated protein
XP_009691714.1	0.0710877	0.314693	0.225896	0.0647027	0.331341	0.195275	0.210586	conserved hypothetical protein
XP_009691715.1	0.0194707	0.237014	0.0821501	0.0213136	0.237804	0.0896266	0.085888	conserved hypothetical protein
XP_009691716.1	0.0562893	0.560064	0.100505	0.045919	0.483951	0.0948836	0.097694	uncharacterized protein
XP_009691717.1	0.0281335	0.434472	0.0647532	0.0317223	0.477436	0.0664431	0.065598	uncharacterized protein
XP_009691718.1	0.0896876	0.558249	0.160659	0.0886487	0.562898	0.157486	0.159073	conserved hypothetical protein

protein_id	Chitose_ka	Chitose_ks	Chitose_kaks	Buffeli_ka	Buffeli_ks	Buffeli_kaks	mean_kaks	function
XP_009691719.1	0.0820705	0.551884	0.14871	0.0692087	0.788396	0.0877843	0.118247	conserved hypothetical protein
XP_009691720.1	0.0745166	0.615274	0.121111	0.0735074	0.785063	0.0936325	0.107372	conserved hypothetical protein
XP_009691721.1	0.0605258	0.637925	0.0948792	0.0545855	0.552899	0.0987261	0.096803	ABC transporter
XP_009691722.1	0.109342	0.440525	0.248209	0.0663831	0.299256	0.221827	0.235018	uncharacterized protein
XP_009691723.1	0.533628	0.978297	0.545466				0.545466	hypothetical protein
XP_009691724.1	0.0626784	0.376716	0.166381	0.0690522	0.474893	0.145406	0.155894	conserved hypothetical protein
XP_009691725.1	0.149804	0.148945	1.00577	0.138735	0.141911	0.977619	0.991695	hypothetical protein
XP_009691726.1	0.00237421	0.317922	0.00746791	0.00456417	0.303168	0.0150549	0.011261	60S ribosomal protein L19
XP_009691727.1	0.0431212	0.390838	0.11033	0.0347803	0.196808	0.176722	0.143526	endonuclease III
XP_009691728.1	0.0862848	0.238415	0.361909	0.0807066	0.350537	0.230237	0.296073	conserved hypothetical protein
XP_009691731.1	0.0632042	0.368365	0.171581	0.051987	0.250254	0.207737	0.189659	conserved hypothetical protein
XP_009691732.1	0.0807584	0.541258	0.149205	0.0480653	0.288344	0.166694	0.15795	conserved hypothetical protein
XP_009691733.1	0.0394449	0.313766	0.125714	0.0162526	0.12714	0.127833	0.126773	conserved hypothetical protein
XP_009691734.1	0.0592101	0.516663	0.114601	0.0624158	0.639909	0.0975385	0.10607	conserved hypothetical protein
XP_009691735.1	0.110387	0.801682	0.137694	0.14772	0.660538	0.223635	0.180665	conserved hypothetical protein
XP_009691736.1	0.114137	0.760214	0.150137	0.106367	0.541964	0.196263	0.1732	conserved hypothetical protein
XP_009691737.1	0.00350606	0.337451	0.0103898	0.00433167	0.293801	0.0147436	0.012567	histone H3
XP_009691738.1	0.170258	0.334262	0.509354	0.025756	0.0568171	0.453314	0.481334	hypothetical protein
XP_009691739.1	0.0675398	0.916564	0.073688	0.041276	0.695988	0.0593056	0.066497	conserved hypothetical protein
XP_009691740.1	0.027344	0.366678	0.0745722				0.0745722	conserved hypothetical protein
XP_009691741.1	0.10731	0.596652	0.179853	0.0991462	0.521635	0.190068	0.18496	conserved hypothetical protein
XP_009691742.1	0.120556	0.335041	0.359823	0.0818252	0.318139	0.2572	0.308511	uncharacterized protein
XP_009691743.1	0.0111089	0.860101	0.0129158	0.0121235	0.783992	0.0154638	0.01419	conserved hypothetical protein
XP_009691744.1	0.0267709	0.704834	0.0379818	0.0200197	0.631285	0.0317127	0.034847	alpha adaptin
XP_009691745.1	0.0290682	0.385719	0.0753611	0.0397036	0.302177	0.131392	0.103377	synaptobrevin
XP_009691746.1	0.0601328	0.613541	0.0980093	0.0608373	0.665599	0.0914023	0.094706	conserved hypothetical protein
XP_009691747.1	0.0901421	0.301931	0.298552	0.0713211	0.213331	0.334321	0.316437	uncharacterized protein
XP_009691748.1	0.0290068	0.387105	0.0749325	0.0205502	0.279055	0.0736423	0.074287	arsenical pump-driving ATPase
XP_009691749.1	0.0429977	0.351265	0.122408				0.122408	uncharacterized protein
XP_009691750.1	0.0671934	0.805161	0.0834533	0.0523376	0.796531	0.065707	0.07458	protein kinase
XP_009691751.1	0.06819	0.498146	0.136888	0.0621598	0.30443	0.204184	0.170536	conserved hypothetical protein
XP_009691752.1	0.043752	0.772054	0.0566697	0.0454406	0.71177	0.0638417	0.060256	methionyl-tRNA synthetase
XP_009691753.1	0.104515	0.395194	0.264465	0.116242	0.368194	0.315709	0.290087	conserved hypothetical protein
XP_009691756.1	NA	NA	NA				NA	hypothetical protein
XP_009691757.1				0.0940492	0.328395	0.286391	0.286391	uncharacterized protein
XP_009691759.1	0.034512	0.471594	0.0731818				0.0731818	conserved hypothetical protein
XP_009691760.1	0.0651649	0.387157	0.168316	0.0589552	0.395438	0.149088	0.158702	conserved hypothetical protein
XP_009691762.1	0.0993301	0.556852	0.178378	0.0906855	0.509259	0.178074	0.178226	conserved hypothetical protein
XP_009691766.1	0.047012	1.54562	0.0304163	0.0401942	1.15567	0.0347801	0.032598	uncharacterized protein
XP_009691767.1	0.116726	0.566959	0.20588	0.119052	0.570479	0.208688	0.207284	uncharacterized protein
XP_009691769.1	0.105061	0.989809	0.106142				0.106142	uncharacterized protein
XP_009691770.1	0.134548	0.419006	0.321113	0.133085	0.334572	0.397777	0.359445	conserved hypothetical protein
XP_009691771.1	0.0587479	0.412976	0.142255	0.0474347	0.341271	0.138994	0.140624	uncharacterized protein
XP_009691772.1	0.0470462	0.44044	0.106816	0.0438303	0.309119	0.141791	0.124303	thioredoxin reductase
XP_009691773.1	0.0793264	0.487256	0.162802	0.0653702	0.501837	0.130262	0.146532	conserved hypothetical protein
XP_009691774.1	0.065786	0.239145	0.275088	4.46907E-05	0.0446907	0.001	0.138044	hypothetical protein
XP_009691775.1	0.107427	0.3998	0.268702				0.268702	conserved hypothetical protein
XP_009691776.1	0.0477771	0.684129	0.0698364	0.0440643	0.479812	0.0918365	0.080836	conserved hypothetical protein
XP_009691777.1	0.0702859	0.273763	0.25674	0.0834629	0.131535	0.634532	0.445636	uncharacterized protein
XP_009691778.1	0.0104444	0.33808	0.0308932				0.0308932	ADP-ribosylation factor
XP_009691779.1	0.0844881	0.747801	0.112982	0.0488124	0.517486	0.0943261	0.103654	conserved hypothetical protein
XP_009691780.1	0.0759135	0.524033	0.144864	0.072665	0.476088	0.152629	0.148747	conserved hypothetical protein
XP_009691781.1	0.105037	0.733003	0.143296	0.116535	0.653778	0.178249	0.160772	conserved hypothetical protein

protein_id	Chitose_ka	Chitose_ks	Chitose_kaks	Buffeli_ka	Buffeli_ks	Buffeli_kaks	mean_kaks	function
XP_009691782.1	0.0549368	0.333067	0.164942	0.0457741	0.575663	0.0795153	0.122229	hypothetical protein
XP_009691783.1	0.120823	0.579551	0.208477	0.121217	0.501684	0.24162	0.225048	conserved hypothetical protein
XP_009691784.1	0.0916719	0.459757	0.199392	0.0708553	0.367167	0.192978	0.196185	conserved hypothetical protein
XP_009691786.1	0.0260796	0.593982	0.0439064	0.0244457	0.512401	0.0477082	0.045807	2-oxoglutarate dehydrogenase E1 component
XP_009691787.1	0.138034	0.365643	0.37751	0.16496	0.451969	0.364981	0.371246	hypothetical protein
XP_009691789.1	0.1354	0.443311	0.30543	0.148049	0.324625	0.45606	0.380745	hypothetical protein
XP_009691792.1	0.0772171	0.474931	0.162586	0.0240703	0.122139	0.197073	0.17983	uncharacterized protein
XP_009691794.1	0.00984309	0.583665	0.0168643	0.0109335	0.819308	0.0133449	0.015105	uncharacterized protein
XP_009691795.1	0.0516428	0.667177	0.0774049	0.0401751	0.414902	0.0968304	0.087118	uncharacterized protein
XP_009691796.1	0.0952349	0.442943	0.215005	0.109762	0.433876	0.252979	0.233992	conserved hypothetical protein
XP_009691797.1	0.0745413	0.0939489	0.793423				0.793423	hypothetical protein
XP_009691798.1	0.114662	0.560058	0.204733	0.0947318	0.438428	0.216072	0.210402	uncharacterized protein
XP_009691799.1	0.00776069	0.0991248	0.0782921				0.0782921	conserved hypothetical protein
XP_009691800.1	0.0340757	0.111188	0.30647				0.30647	hypothetical protein
XP_009691801.1	0.158515	0.465771	0.340328				0.340328	hypothetical protein
XP_009691802.1	0.0787776	0.884082	0.0891066	0.0905351	1.04822	0.0863701	0.087738	uncharacterized protein
XP_009691803.1	0.0812122	0.690812	0.11756	0.0626676	0.455473	0.137588	0.127574	uncharacterized protein
XP_009691804.1	0.0257849	0.437656	0.0589158	0.0293527	0.392418	0.0747994	0.066858	conserved hypothetical protein
XP_009691805.1	0.0217392	0.153137	0.14196	0.0167693	0.216769	0.0773599	0.10966	4-methyl-5(b-hydroxyethyl)-thiazol monophosphate biosynthesis enzyme
XP_009691807.1	0.0697614	0.857904	0.0813161	0.0119116	0.0189076	0.629994	0.355655	conserved hypothetical protein
XP_009691808.1	0.0272476	0.58547	0.0465397	0.0146374	0.644311	0.0227179	0.034629	uncharacterized protein
XP_009691809.1	0.0376606	0.344499	0.10932	0.0253261	0.283869	0.0892177	0.099269	uncharacterized protein
XP_009691810.1	0.0884741	0.869001	0.101811	0.0813483	1.00331	0.08108	0.091445	conserved hypothetical protein
XP_009691811.1	0.0530745	0.370975	0.143067	0.0286827	0.187339	0.153106	0.148087	cytochrome reductase
XP_009691812.1	0.0541936	0.551018	0.0983516	0.0426648	0.450589	0.0946869	0.096519	conserved hypothetical protein
XP_009691813.1	0.106708	0.452515	0.235812	0.102054	0.372927	0.273656	0.254734	uncharacterized protein
XP_009691814.1	0.0081525	0.199617	0.0408408				0.0408408	predicted protein
XP_009691815.1	0.0574221	0.4423	0.129826	0.0557757	0.634114	0.0879585	0.108892	uncharacterized protein
XP_009691816.1	0.032435	0.554561	0.0584877	0.0484472	0.523329	0.0925751	0.075531	uncharacterized protein
XP_009691817.1	0.0933892	0.570045	0.163828	0.112672	0.520231	0.216581	0.190204	uncharacterized protein
XP_009691818.1	0.0955837	0.389504	0.245398	0.100902	0.415532	0.242826	0.244112	uncharacterized protein
XP_009691819.1	0.0664843	0.409234	0.16246	0.0764162	0.399021	0.191509	0.176984	conserved hypothetical protein
XP_009691820.1	0.0644541	0.456725	0.141122	0.0595263	0.50111	0.118789	0.129956	conserved hypothetical protein
XP_009691821.1	0.105715	0.587786	0.179852	0.066442	0.609118	0.109079	0.144465	conserved hypothetical protein
XP_009691822.1	0.0217337	0.660693	0.0328953	0.0237536	0.395589	0.0600462	0.046471	fumarate hydratase class I
XP_009691824.1	0.162283	0.478665	0.339032				0.339032	uncharacterized protein
XP_009691825.1	0.0790489	0.629747	0.125525				0.125525	uncharacterized protein
XP_009691826.1	0.00938605	0.542904	0.0172886	0.00862876	0.414082	0.0208383	0.019063	conserved hypothetical protein
XP_009691827.1	0.17262	0.466317	0.370177	0.151888	0.475712	0.319286	0.344731	conserved hypothetical protein
XP_009691828.1	0.0510049	0.596708	0.0854772	0.0305623	0.638058	0.0478989	0.066688	conserved hypothetical protein
XP_009691829.1	0.0338072	0.507585	0.0666041	0.0334185	0.276337	0.120934	0.093769	conserved hypothetical protein
XP_009691830.1	0.0323142	0.435359	0.0742244	0.026355	0.459422	0.0573655	0.065795	uncharacterized protein
XP_009691831.1	0.0455318	0.495215	0.0919435	0.0295185	0.231626	0.127441	0.109692	conserved hypothetical protein
XP_009691832.1	0.0785304	0.54949	0.142915	0.0738653	0.434261	0.170094	0.156504	conserved hypothetical protein
XP_009691833.1	0.0380478	0.505532	0.0752629	0.00913565	0.451286	0.0202436	0.047753	CCR4-NOT transcription complex subunit 3
XP_009691834.1	0.0736882	0.0976407	0.754688				0.754688	conserved hypothetical protein
XP_009691835.1	0.0256146	0.271609	0.0943069	0.0072693	0.226631	0.0320755	0.063191	uncharacterized protein
XP_009691836.1	0.0897801	0.321511	0.279244	0.0726698	0.281375	0.258267	0.268756	uncharacterized protein
XP_009691837.1	0.0283219	0.843171	0.0335898	0.0181592	0.297336	0.0610729	0.047331	cysteinyl-tRNA synthetase
XP_009691838.1	0.0586704	0.629306	0.0932302	0.0355928	0.407853	0.0872685	0.090249	conserved hypothetical protein
XP_009691840.1	0.0449554	0.394878	0.113846	0.0250461	0.421426	0.0594318	0.086639	hexose transporter
XP_009691842.1	0.020781	0.528459	0.0393237	0.0234481	0.46153	0.0508052	0.045064	uncharacterized protein
XP_009691843.1	0.113267	0.672278	0.168483	0.100658	0.638482	0.157652	0.163067	conserved hypothetical protein

protein_id	Chitose_ka	Chitose_ks	Chitose_kaks	Buffeli_ka	Buffeli_ks	Buffeli_kaks	mean_kaks	function
XP_009691844.1	0.0982172	1.01717	0.0965592	0.102811	0.774601	0.132728	0.114644	predicted protein
XP_009691845.1	0.00513564	0.245523	0.0209172	0.00287719	0.200193	0.0143721	0.017645	centrin
XP_009691846.1	0.0662972	0.328819	0.201622	0.0530119	0.264695	0.200276	0.200949	2-C-methyl-D-erythritol 4-phosphate cytidylyltransferase
XP_009691847.1	0.0192406	0.33862	0.0568206	0.0200497	0.313327	0.0639898	0.060405	aspartyl protease precursor
XP_009691848.1	0.0471773	0.578072	0.0816115	0.0484226	0.465567	0.104008	0.09281	conserved hypothetical protein
XP_009691849.1	0.143966	0.469286	0.306776	0.0379301	0.143144	0.264979	0.285878	conserved hypothetical protein
XP_009691850.1	0.017925	0.624337	0.0287105	0.0218681	0.43889	0.0498258	0.039268	uncharacterized protein
XP_009691851.1	0.000433739	0.433739	0.001	0.000362515	0.362515	0.001	0.001	40S ribosomal protein S27
XP_009691852.1	0.072033	1.07847	0.0667917	0.0524209	0.793628	0.0660523	0.066422	conserved hypothetical protein
XP_009691855.1	0.00387536	0.322364	0.0120217	0.000286843	0.286843	0.001	0.006511	60S ribosomal protein L24
XP_009691856.1	0.0360409	0.940012	0.0383409	0.0303255	0.660022	0.0459463	0.042144	uncharacterized protein
XP_009691857.1	0.0231503	1.00218	0.0230999	0.0204804	0.722538	0.0283451	0.025723	carbamoyl phosphate synthase II
XP_009691858.1	0.139438	0.195662	0.712644	0.166373	0.113082	1.47127	1.091957	hypothetical protein
XP_009691859.1	0.171215	0.242172	0.706997	0.0566222	0.0236481	2.39437	1.550683	hypothetical protein
XP_009691860.1	0.0181256	0.85668	0.0211579	0.010075	0.604241	0.0166739	0.018916	glycogen synthase kinase
XP_009691861.1	0.0569639	0.611551	0.0931467	0.028546	0.465455	0.0613292	0.077238	26S proteasome regulatory subunit
XP_009691863.1	0.0823827	0.643838	0.127956	0.0521208	0.401932	0.129676	0.128816	conserved hypothetical protein
XP_009691864.1	0.0782761	0.493546	0.158599	0.0713874	0.418322	0.170652	0.164625	uncharacterized protein
XP_009691865.1	0.126035	0.57815	0.217997	0.0457338	0.315533	0.144941	0.181469	hypothetical protein
XP_009691866.1	0.0339146	0.42037	0.080678	0.0317442	0.370487	0.0856825	0.08318	conserved hypothetical protein
XP_009691867.1	0.114253	0.636697	0.179446	0.112267	0.357534	0.314005	0.246725	hypothetical protein
XP_009691872.1	0.0836461	0.498061	0.167944				0.167944	uncharacterized protein
XP_009691875.1	0.0590583	0.618279	0.0955205	0.075243	0.850591	0.0884597	0.09199	U1 snRNP protein
XP_009691876.1	0.0739929	1.29616	0.0570862	0.0433001	0.280663	0.154278	0.105682	uncharacterized protein
XP_009691877.1	0.0398317	0.742894	0.053617				0.053617	vacuolar ATP synthase subunit H
XP_009691881.1	0.0725293	0.566556	0.128018	0.0501298	0.498231	0.100616	0.114317	conserved hypothetical protein
XP_009691882.1	0.072616	0.355579	0.204219				0.204219	conserved hypothetical protein
XP_009691883.1	0.055349	1.25659	0.044047	0.0222325	0.195766	0.113567	0.078807	conserved hypothetical protein
XP_009691884.1	0.0486587	0.430337	0.113071				0.113071	conserved hypothetical protein
XP_009691885.1	0.10821	0.768763	0.140758				0.140758	uncharacterized protein
XP_009691887.1	0.038867	1.07356	0.0362039	0.0424203	4.39924	0.00964264	0.022923	conserved hypothetical protein
XP_009691888.1	0.0607586	0.507273	0.119775	0.0619567	0.527222	0.117515	0.118645	conserved hypothetical protein
XP_009691889.1	NA	NA	NA				NA	DNA-directed RNA polymerase II
XP_009691890.1	0.0611905	0.499513	0.1225	0.058288	0.346325	0.168304	0.145402	RNA polymerase II carboxyterminal domain phosphatase
XP_009691891.1	0.0149917	0.269352	0.0556584				0.0556584	ubiquitin-conjugating enzyme
XP_009691892.1	0.18029	0.785033	0.229659	0.0966575	0.448681	0.215426	0.222543	hypothetical protein
XP_009691893.1	0.133845	0.518309	0.258233	0.14967	0.518333	0.288752	0.273493	conserved hypothetical protein
XP_009691894.1	0.123832	0.606062	0.204322	0.12243	0.53114	0.230505	0.217413	conserved hypothetical protein
XP_009691895.1	0.000266831	0.266831	0.001				0.001	methyltransferase
XP_009691896.1	0.0749871	0.399381	0.187759				0.187759	endonuclease
XP_009691897.1	0.118884	0.368095	0.322972	0.085327	0.377793	0.225856	0.274414	uncharacterized protein
XP_009691898.1	0.182157	0.587264	0.310178	0.158926	0.383824	0.41406	0.362119	hypothetical protein
XP_009691899.1	0.0363174	0.568476	0.0638855				0.0638855	conserved hypothetical protein
XP_009691900.1	0.0380837	0.338545	0.112492	0.0397465	0.222629	0.178532	0.145512	putative RPC10 subunit of RNA polymerases I%2C II%2C and III
XP_009691901.1	0.0326123	0.565269	0.0576935	0.0166646	0.354096	0.0470624	0.052378	conserved hypothetical protein
XP_009691902.1	0.0454973	0.404071	0.112597	0.0380108	0.178549	0.212887	0.162742	uncharacterized protein
XP_009691903.1	0.0518277	0.348053	0.148907	0.0622565	0.498818	0.124808	0.136858	uncharacterized protein
XP_009691904.1	0.0461723	0.478551	0.0964836	NA	NA	NA	NA	50S ribosomal protein L17
XP_009691905.1	0.0427137	0.506104	0.0843971	0.0184512	0.30214	0.0610683	0.072733	ribosomal protein L15
XP_009691908.1	0.106929	0.857395	0.124714	0.112625	0.619253	0.181873	0.153293	uncharacterized protein
XP_009691909.1	0.101289	0.326619	0.310113	0.110462	0.306059	0.360916	0.335515	uncharacterized protein
XP_009691910.1	0.20878	0.441745	0.472625	0.178352	0.448398	0.397753	0.435189	conserved hypothetical protein
XP_009691911.1	0.133938	0.372403	0.359658	0.115957	0.342869	0.338195	0.348927	uncharacterized protein

protein_id	Chitose_ka	Chitose_ks	Chitose_kaks	Buffeli_ka	Buffeli_ks	Buffeli_kaks	mean_kaks	function
XP_009691912.1				0.0843059	0.279165	0.301993	0.301993	conserved hypothetical protein
XP_009691913.1	0.161993	0.497983	0.325298	0.0826081	0.610974	0.135207	0.230252	hypothetical protein
XP_009691914.1	0.122953	0.943074	0.130375	0.130511	0.616171	0.211809	0.171092	conserved hypothetical protein
XP_009691915.1	0.159014	0.756922	0.210079	0.1694	0.7084	0.23913	0.224604	conserved hypothetical protein
XP_009691916.1	0.0889268	0.762217	0.116669	0.0853756	0.767404	0.111253	0.113961	uncharacterized protein
XP_009691917.1	0.104094	0.715435	0.145497	0.103366	0.741697	0.139364	0.14243	conserved hypothetical protein
XP_009691918.1	0.0606161	0.186609	0.324829				0.324829	conserved hypothetical protein
XP_009691922.1	0.0925466	0.308949	0.299553	0.114	0.26608	0.428442	0.363997	conserved hypothetical protein
XP_009691923.1				0.168069	0.198266	0.847693	0.847693	hypothetical protein
XP_009691924.1				0.087143	0.107164	0.813176	0.813176	hypothetical protein
XP_009691925.1				0.0666116	0.162461	0.410015	0.410015	conserved hypothetical protein
XP_009691926.1				0.0219368	0.101557	0.216005	0.216005	ABC transporter
XP_009691941.1	0.115412	0.726311	0.158901	0.111344	0.688651	0.161684	0.160293	conserved hypothetical protein
XP_009691944.1	0.0695638	0.619743	0.112246	0.0541367	0.295499	0.183204	0.147725	conserved hypothetical protein
XP_009691945.1	0.0878897	0.304287	0.288838	0.111441	0.251436	0.443219	0.366028	hypothetical protein
XP_009691946.1	0.0463305	0.605636	0.0764988	0.0418983	0.589341	0.0710935	0.073796	uncharacterized protein
XP_009691947.1	0.0137095	0.478379	0.0286583	0.0124848	0.454743	0.0274546	0.028056	ubiquinol-cytochrome c reductase
XP_009691948.1	0.0376765	0.411714	0.0915113	0.0550185	0.472277	0.116496	0.104004	uncharacterized protein
XP_009691949.1	0.00462404	0.329109	0.0140502	0.000226997	0.226997	0.001	0.007525	DNA-directed RNA polymerase
XP_009691950.1	0.064511	0.678915	0.0950208	0.072554	0.616139	0.117756	0.106388	conserved hypothetical protein
XP_009691951.1	0.0157238	0.334842	0.0469589	0.0153268	0.288594	0.0531085	0.050034	ribosomal protein S17
XP_009691952.1	0.0304467	0.284448	0.107038	0.00993568	0.20535	0.0483842	0.077711	conserved hypothetical protein
XP_009691953.1	0.0383178	0.669863	0.0572024	0.05581	0.591408	0.094368	0.075785	importin alpha
XP_009691954.1	0.0585026	0.88353	0.0662146	0.0532817	0.782981	0.0680498	0.067132	uncharacterized protein
XP_009691955.1	0.00722304	0.665006	0.0108616	0.00757405	0.582112	0.0130113	0.011936	ubiquitin carrier protein
XP_009691956.1	0.0867061	0.415249	0.208805	0.061765	0.383625	0.161004	0.184904	uncharacterized protein
XP_009691957.1	0.0731082	0.147863	0.494432				0.494432	hypothetical protein
XP_009691958.1	0.104459	0.562726	0.18563	0.0957354	0.479885	0.199496	0.192563	hypothetical protein
XP_009691959.1	0.0824207	0.414572	0.198809	0.0719829	0.543725	0.132388	0.165599	conserved hypothetical protein
XP_009691960.1	0.0362283	0.377576	0.0959496	0.0366941	0.385854	0.0950985	0.095524	conserved hypothetical protein
XP_009691961.1	0.0530841	0.325146	0.163262	0.0476346	0.325458	0.146362	0.154812	phosphoinositide binding protein
XP_009691962.1	0.100708	0.430641	0.233856	0.112825	0.562039	0.200743	0.2173	uncharacterized protein
XP_009691963.1	0.102431	0.730962	0.140132	0.103619	0.647612	0.160001	0.150066	conserved hypothetical protein
XP_009691964.1	NA	NA	NA	NA	NA	NA	NA	hypothetical protein
XP_009691966.1	0.0209824	0.213167	0.0984321				0.0984321	uncharacterized protein
XP_009691967.1	0.0526348	0.129474	0.406527	0.062338	0.183764	0.339228	0.372877	uncharacterized protein
XP_009691968.1	0.0681985	0.523915	0.130171				0.130171	uncharacterized protein
XP_009691969.1	0.0258612	0.358819	0.072073	0.0240876	0.668099	0.0360539	0.054063	S-adenosylmethionine synthetase
XP_009691970.1	0.000051824	0.051824	0.001				0.001	high-mobility group protein
XP_009691972.1	0.016306	0.272182	0.0599085	0.0107581	0.169584	0.0634382	0.061673	uncharacterized protein
XP_009691973.1	0.0834728	0.502727	0.16604	0.0867706	0.548802	0.158109	0.162075	uncharacterized protein
XP_009691974.1	0.0416716	0.681406	0.0611553	0.043829	0.870459	0.0503516	0.055753	uncharacterized protein
XP_009691975.1	0.0508999	0.456849	0.111415	0.0508626	0.416545	0.122106	0.116761	conserved hypothetical protein
XP_009691976.1	0.0786265	0.54784	0.143521	0.0688916	0.470609	0.146388	0.144954	uncharacterized protein
XP_009691977.1	0.0275614	1.34274	0.0205263	0.0288229	1.19631	0.0240932	0.02231	glutamate dehydrogenase
XP_009691978.1	0.047118	0.524045	0.089912	0.0501347	0.476374	0.105242	0.097577	dihydroorotase
XP_009691979.1	0.0867141	0.496656	0.174596	0.0636205	0.445224	0.142896	0.158746	uncharacterized protein
XP_009691980.1	0.0365378	0.533495	0.0684876	0.0393371	0.555003	0.0708772	0.069682	serine/threonine protein kinase
XP_009691981.1	0.0546706	0.399328	0.136907	0.0572727	0.415681	0.13778	0.137344	uncharacterized protein
XP_009691982.1	0.0537259	0.313649	0.171293	0.0504695	0.477348	0.105729	0.138511	ubiquitin-like protease
XP_009691983.1	0.0602223	0.871479	0.0691036	0.0617664	0.939006	0.0657785	0.067441	likely GTP/GDP exchange factor for ARF
XP_009691984.1	0.0623504	0.280175	0.222541	0.0475445	0.174439	0.272557	0.247549	uncharacterized protein
XP_009691985.1	0.0272019	0.0210544	1.29198				1.29198	hypothetical protein

protein_id	Chitose_ka	Chitose_ks	Chitose_kaks	Buffeli_ka	Buffeli_ks	Buffeli_kaks	mean_kaks	function
XP_009691986.1	0.0762472	0.472774	0.161276	0.0648364	0.468846	0.138289	0.149782	conserved hypothetical protein
XP_009691987.1	0.042821	0.43572	0.0982762	0.0307205	0.42824	0.0717367	0.085006	phenylalanyl-tRNA synthetase
XP_009691988.1	0.0649099	0.727232	0.089256	0.0496559	0.649842	0.0764122	0.082834	conserved hypothetical protein
XP_009691989.1	0.0314282	0.24319	0.129233	0.0276562	0.244355	0.113181	0.121207	conserved hypothetical protein
XP_009691990.1	0.100164	0.583378	0.171696	0.0977019	0.539669	0.18104	0.176368	beta-tubulin cofactor D
XP_009691991.1	0.0527145	0.807099	0.0653136	0.0579876	0.473575	0.122446	0.09388	conserved hypothetical protein
XP_009691992.1	0.0610128	0.610762	0.0998963	0.0664035	0.581264	0.11424	0.107068	conserved hypothetical protein
XP_009691993.1	0.0453896	0.538913	0.0842244	0.0490762	0.53921	0.091015	0.08762	uncharacterized protein
XP_009691994.1	0.0506298	0.230217	0.219921	0.0454796	0.214117	0.212406	0.216164	conserved hypothetical protein
XP_009691995.1	0.0364487	0.474334	0.0768419	0.0210805	0.440536	0.0478519	0.062347	conserved hypothetical protein
XP_009691996.1	0.0141694	0.953147	0.0148659	0.0204587	1.09068	0.0187577	0.016812	nascent polypeptide associated complex subunit alpha
XP_009691997.1	0.050762	0.505973	0.100326	0.0383125	0.408702	0.0937421	0.097034	signal recognition particle subunit
XP_009691998.1	0.018179	0.115414	0.157511				0.157511	conserved hypothetical protein
XP_009691999.1	0.0415962	0.460563	0.090316	0.0350608	0.509555	0.0688067	0.079561	uncharacterized protein
XP_009692000.1	0.0272743	0.706819	0.0385874	0.0222725	0.580545	0.0383648	0.038476	conserved hypothetical protein
XP_009692001.1	0.0368567	0.415307	0.0887455	0.0285736	0.34708	0.0823258	0.085536	uncharacterized protein
XP_009692002.1	0.0315916	0.432028	0.0731241	0.03328	0.486138	0.0684579	0.070791	RNase L inhibitor protein
XP_009692003.1	0.049049	0.495191	0.0990507	0.0497235	0.493942	0.100667	0.099859	uncharacterized protein
XP_009692004.1	0.0384269	0.725095	0.0529957	0.0184889	0.329904	0.0560433	0.054519	conserved hypothetical protein
XP_009692005.1	0.0297363	0.463058	0.0642171	0.0355065	0.440053	0.0806869	0.072452	uncharacterized protein
XP_009692006.1	0.0569567	0.715136	0.0796446	0.0495174	0.557006	0.0888993	0.084272	conserved hypothetical protein
XP_009692007.1	0.0195632	0.475439	0.0411477	0.023447	0.440689	0.0532054	0.047177	uncharacterized protein
XP_009692008.1	0.0708589	0.302588	0.234176	0.0660082	0.252812	0.261096	0.247636	50S ribosomal protein L21
XP_009692009.1	0.0320706	0.698006	0.0459461	0.0162287	0.388899	0.04173	0.043838	uncharacterized protein
XP_009692010.1	0.053651	0.483055	0.111066	0.0477502	0.359812	0.132709	0.121887	conserved hypothetical protein
XP_009692011.1	0.000261544	0.261544	0.001	0.000290712	0.290712	0.001	0.001	40S ribosomal protein S7
XP_009692012.1	0.0448884	0.266872	0.168202				0.168202	hypothetical protein
XP_009692013.1	0.0696874	0.478125	0.145751	0.0632752	0.523719	0.120819	0.133285	uncharacterized protein
XP_009692014.1	0.082578	0.518866	0.159151	0.0602016	0.421766	0.142737	0.150944	uncharacterized protein
XP_009692015.1	0.00407675	0.575877	0.0070792	0.0039516	0.479306	0.00824441	0.007662	40S ribosomal protein S10
XP_009692016.1	0.0392208	0.498942	0.0786079	0.0408927	0.663958	0.0615893	0.070099	uncharacterized protein
XP_009692017.1	0.0190211	0.127875	0.148748				0.148748	conserved hypothetical protein
XP_009692018.1	0.035862	0.245002	0.146374				0.146374	conserved hypothetical protein
XP_009692019.1	0.0156234	0.324614	0.0481291	0.014219	0.249866	0.0569065	0.052518	vacuolar proton-translocating ATPase
XP_009692020.1	0.0414142	0.572432	0.0723478	0.0492819	0.767085	0.0642458	0.068297	FtsJ cell division protein
XP_009692021.1	0.0870998	0.48669	0.178963	0.0468153	0.525638	0.0890639	0.134013	conserved hypothetical protein
XP_009692022.1	0.0608955	0.344391	0.176821	0.0378983	0.245379	0.154448	0.165635	uncharacterized protein
XP_009692023.1	0.0040794	0.402059	0.0101463	0.00856634	0.348244	0.0245987	0.017372	proteasome subunit alpha type
XP_009692024.1	0.0486034	0.243144	0.199896	0.0655324	0.29223	0.22425	0.212073	proteasome component
XP_009692025.1	0.0611724	0.671259	0.0911308	0.0348414	0.816078	0.0426937	0.066912	conserved hypothetical protein
XP_009692026.1	0.00572344	0.300525	0.0190448	0.00206958	0.28892	0.00716317	0.013104	endoplasmic reticulum lumen protein ERD2
XP_009692027.1	0.0247038	0.621425	0.0397534	0.0112441	0.464488	0.0242075	0.03198	uncharacterized protein
XP_009692028.1	0.0668408	0.714913	0.0934951	0.0500419	0.822135	0.0608682	0.077182	asparagine-rich protein
XP_009692029.1	0.00646063	0.503741	0.0128253	0.0113926	0.60218	0.0189189	0.015872	DEAD-box family ATP-dependent helicase
XP_009692030.1	0.0181575	0.450543	0.0403014	0.0173457	0.307644	0.0563823	0.048342	serine/threonine kinase
XP_009692031.1	0.0413224	0.354437	0.116586	0.0336715	0.366487	0.0918763	0.104231	conserved hypothetical protein
XP_009692032.1	0.00707681	0.177556	0.0398567				0.0398567	Plasmodium falciparum CPW-WPC repeat containing protein
XP_009692033.1	0.0354311	0.0204186	1.73524				1.73524	hypothetical protein
XP_009692034.1	0.000410087	0.410087	0.001				0.001	conserved hypothetical protein
XP_009692035.1	0.0332777	1.06569	0.0312265	0.0317478	1.0691	0.0296959	0.030461	DNA polymerase epsilon catalytic subunit
XP_009692036.1	0.0362952	0.314025	0.11558	0.00363708	0.204042	0.0178252	0.066703	conserved hypothetical protein
XP_009692037.1	0.0413631	0.75549	0.05475	0.0335454	0.691659	0.0484999	0.051625	uncharacterized protein
XP_009692038.1	0.0595731	0.421864	0.141214	0.045319	0.41535	0.10911	0.125162	microtubule-associated protein

protein_id	Chitose_ka	Chitose_ks	Chitose_kaks	Buffeli_ka	Buffeli_ks	Buffeli_kaks	mean_kaks	function
XP_009692039.1	0.0814577	0.554261	0.146966	0.0801231	0.515146	0.155535	0.151251	conserved hypothetical protein
XP_009692040.1	0.119979	0.408648	0.2936	0.101514	0.429633	0.236282	0.264941	methylase
XP_009692041.1	0.0646802	0.399026	0.162095	0.0747573	0.475625	0.157177	0.159636	uncharacterized protein
XP_009692042.1	0.0133287	0.645555	0.0206469	0.0201566	0.893243	0.0225656	0.021606	methyltransferase
XP_009692043.1	0.0903701	0.8793	0.102775	0.0737249	0.852617	0.086469	0.094622	predicted protein
XP_009692044.1	0.0447666	0.396034	0.113037	0.0442247	0.382424	0.115643	0.11434	uncharacterized protein
XP_009692046.1	0.0646953	0.53223	0.121555	0.0642158	0.435245	0.147539	0.134547	pentatricopeptide repeat containing protein
XP_009692047.1	0.0243813	0.562705	0.0433288	0.0254565	0.581434	0.0437823	0.043556	endopeptidase ATP-binding subunit
XP_009692048.1	0.0295729	0.58973	0.0501464	0.0138588	0.691345	0.0200461	0.035096	vacuolar protein sorting
XP_009692049.1	0.00986892	0.372374	0.0265027	0.00793451	0.305555	0.0259676	0.026235	conserved hypothetical protein
XP_009692050.1	0.0426997	0.264676	0.161329	0.0248503	0.124251	0.200001	0.180665	florfenicol resistance protein
XP_009692051.1	0.061524	0.225865	0.272393				0.272393	hypothetical protein
XP_009692052.1	0.0324638	0.481402	0.067436	0.0256063	0.429196	0.0596611	0.063549	uncharacterized protein
XP_009692053.1	0.0224125	0.469363	0.0477509	0.00038366	0.38366	0.001	0.024375	fibrillarin
XP_009692054.1	0.0353204	0.578848	0.0610184	0.0428688	0.447191	0.0958624	0.07844	fibrillarin
XP_009692055.1	0.0139223	0.364096	0.038238	0.0187121	0.422871	0.0442501	0.041244	splicing component
XP_009692056.1	0.13654	0.12125	1.1261				1.1261	hypothetical protein
XP_009692057.1	0.0803054	0.641467	0.12519	0.0728189	0.548865	0.132672	0.128931	cell-cycle-related serine/threonine protein kinase
XP_009692058.1	0.010355	0.765437	0.0135282	0.00737778	0.819906	0.00899833	0.011263	conserved hypothetical protein
XP_009692059.1	0.038172	0.869063	0.0439232	0.0317569	0.445886	0.0712221	0.057573	conserved hypothetical protein
XP_009692060.1	0.0660658	0.489837	0.134873	0.0822506	0.438573	0.187541	0.161207	GTPase
XP_009692061.1	0.0820995	0.51155	0.160492	0.0838902	0.521836	0.16076	0.160626	uncharacterized protein
XP_009692062.1	0.134097	0.473758	0.283051	0.159972	0.497212	0.321739	0.302395	conserved hypothetical protein
XP_009692063.1	0.0479588	0.682036	0.0703171	0.0430042	0.731707	0.0587724	0.064545	helicase
XP_009692064.1	0.0900842	0.666477	0.135165	0.0890513	0.661407	0.134639	0.134902	conserved hypothetical protein
XP_009692065.1	0.0107742	0.465677	0.0231367	0.0118759	0.454837	0.0261103	0.024623	Hbeta58/Vps26 protein
XP_009692066.1	0.0357352	0.247807	0.144206				0.144206	uncharacterized protein
XP_009692067.1	0.0133688	0.557056	0.0239991	0.0129642	0.48092	0.026957	0.025478	conserved hypothetical protein
XP_009692068.1	0.0324791	0.513418	0.0632606	0.0615705	0.665978	0.0924512	0.077856	conserved hypothetical protein
XP_009692069.1	0.106845	0.507008	0.210737	0.0789752	0.4716	0.167462	0.1891	conserved hypothetical protein
XP_009692070.1	0.0476595	0.510447	0.0933682	0.0344727	0.473708	0.0727721	0.08307	protein kinase
XP_009692071.1	0.0360575	0.419589	0.0859353	0.0318935	0.305444	0.104417	0.095176	tryptophanyl-tRNA synthetase
XP_009692072.1	0.0909096	0.718891	0.126458	0.0823216	0.63529	0.129581	0.12802	conserved hypothetical protein
XP_009692073.1	0.0341536	0.563438	0.0606164	0.0223823	0.504754	0.044343	0.05248	DEAD-box family helicase
XP_009692074.1	0.0767335	0.623928	0.122985				0.122985	conserved hypothetical protein
XP_009692075.1	0.0521209	0.701315	0.0743189	0.0314226	0.623681	0.0503824	0.062351	coronin
XP_009692076.1	0.00315974	1.08031	0.00292485	0.00352935	0.853003	0.00413756	0.003531	60S ribosomal protein L26
XP_009692077.1	0.0334422	0.711129	0.0470269	0.0289005	1.39715	0.0206853	0.033856	uncharacterized protein
XP_009692078.1	0.0252071	0.358133	0.0703848	0.0246168	0.374158	0.0657926	0.068089	methionine aminopeptidase
XP_009692079.1	0.0221285	0.55759	0.0396859	0.0379639	0.525461	0.0722487	0.055967	uncharacterized protein
XP_009692080.1	0.0479585	0.457439	0.104841	0.0341161	0.556127	0.0613459	0.083093	ubiquitin carboxyl-terminal hydrolase
XP_009692081.1	0.0366731	0.641375	0.0571788	0.0509243	0.646521	0.0787667	0.067973	uncharacterized protein
XP_009692082.1	0.0473185	0.503371	0.0940033				0.0940033	uncharacterized protein
XP_009692083.1	0.0460226	0.560415	0.0821223	0.0381641	0.496186	0.0769148	0.079519	uncharacterized protein
XP_009692084.1	0.0163482	0.160571	0.101813	0.0314601	0.139155	0.22608	0.163946	conserved hypothetical protein
XP_009692085.1	0.0271238	0.899443	0.0301562	0.0204499	0.766463	0.0266809	0.028419	proteasome subunit
XP_009692086.1	0.0581284	0.541283	0.10739	0.063265	0.449431	0.140767	0.124079	uncharacterized protein
XP_009692087.1				0.0847127	0.0540759	1.56655	1.56655	hypothetical protein
XP_009692088.1	0.0463139	0.597234	0.0775473	0.108818	0.61048	0.178251	0.127899	conserved hypothetical protein
XP_009692089.1	0.0491513	0.623398	0.0788442	0.0321217	0.498501	0.0644366	0.07164	glycosyl transferase
XP_009692090.1	0.102517	0.390368	0.262616	0.0923925	0.51204	0.18044	0.221528	conserved hypothetical protein
XP_009692091.1	0.0701804	0.565355	0.124135	0.0591341	0.556208	0.106317	0.115226	uncharacterized protein
XP_009692094.1	0.0466051	0.486456	0.0958054	0.0339287	0.315373	0.107583	0.101694	nucleoside diphosphate hydrolase

protein_id	Chitose_ka	Chitose_ks	Chitose_kaks	Buffeli_ka	Buffeli_ks	Buffeli_kaks	mean_kaks	function
XP_009692095.1	0.20377	1.1171	0.182411				0.182411	hypothetical protein
XP_009692096.1	0.0549704	0.613891	0.0895442				0.0895442	uncharacterized protein
XP_009692097.1	0.099021	0.586923	0.168712	0.100659	0.645614	0.155912	0.162312	40S ribosomal protein S27a
XP_009692098.1	0.060231	0.780474	0.0771723	0.0521402	0.643088	0.0810778	0.079125	uncharacterized protein
XP_009692099.1	0.0142979	0.489119	0.0292319	0.0120948	0.552896	0.0218753	0.025554	conserved hypothetical protein
XP_009692100.1	0.0444685	0.26856	0.165581				0.165581	uncharacterized protein
XP_009692101.1	0.011859	0.621261	0.0190886	0.00728725	0.678439	0.0107412	0.014915	ribosomal protein S2
XP_009692102.1	0.142771	0.745813	0.19143	0.119606	0.724176	0.165161	0.178295	conserved hypothetical protein
XP_009692103.1	0.0400892	0.742536	0.0539896	0.0439256	0.460123	0.095465	0.074727	conserved hypothetical protein
XP_009692104.1	0.0378107	0.4771	0.079251	0.0409636	0.367961	0.111326	0.095288	16S rRNA processing protein
XP_009692105.1	0.0545299	0.309314	0.176293	0.0287031	0.192561	0.14906	0.162677	conserved hypothetical protein
XP_009692106.1	0.0721955	0.724465	0.0996535	0.0574777	0.683791	0.0840573	0.091855	conserved hypothetical protein
XP_009692107.1	0.0346122	1.0764	0.0321557	0.0305699	1.07165	0.0285261	0.030341	valyl-tRNA synthetase
XP_009692108.1	0.0423668	1.16995	0.0362125	0.0406917	0.801015	0.0508002	0.043506	eukaryotic translation initiation factor 3 subunit 8
XP_009692109.1	0.0492672	0.687338	0.0716783	0.034112	0.547505	0.0623043	0.066991	uncharacterized protein
XP_009692110.1	0.021338	0.572091	0.0372982	0.0232642	0.300329	0.0774625	0.05738	mitochondrial large subunit ribosomal protein
XP_009692113.1	0.0451022	0.530071	0.0850871	0.0229124	0.436341	0.0525103	0.068799	conserved hypothetical protein
XP_009692114.1	0.0276254	0.783371	0.0352647	0.0211624	0.728054	0.029067	0.032166	50S ribosomal protein L3
XP_009692115.1	0.046664	0.773326	0.060342	0.0553022	0.76616	0.072181	0.066262	GTP-binding protein/GTPase
XP_009692116.1	0.0214181	1.52943	0.0140039	0.0218944	1.0969	0.0199602	0.016982	vacuolar H+-ATPase subunit
XP_009692118.1	0.0169755	0.795203	0.0213474	0.0506996	0.523933	0.0967673	0.059057	uncharacterized protein
XP_009692119.1	0.102165	0.533512	0.191494				0.191494	uncharacterized protein
XP_009692120.1	0.156743	1.08984	0.143823				0.143823	conserved hypothetical protein
XP_009692121.1	0.0751998	0.812678	0.0925334	0.0536108	1.15653	0.0463548	0.069444	sodium transporter
XP_009692123.1				0.0864506	0.588617	0.146871	0.146871	conserved hypothetical protein
XP_009692124.1	4.10345E-05	0.0410345	0.001				0.001	conserved hypothetical protein
XP_009692125.1	0.129213	0.769815	0.167849	0.0865023	0.54513	0.158682	0.163266	conserved hypothetical protein
XP_009692126.1	0.0260225	0.810087	0.032123	0.0327591	0.71185	0.0460196	0.039071	branched-chain alpha-keto acid dehydrogenase E1
XP_009692127.1	0.112049	1.17567	0.0953058	0.103914	0.968033	0.107346	0.101326	uncharacterized protein
XP_009692129.1	0.0651258	0.830084	0.0784568	0.0126546	1.24589	0.0101571	0.044307	tRNA-splicing endonuclease
XP_009692130.1				0.11495	1.15428	0.0995865	0.0995865	uncharacterized protein
XP_009692131.1	0.00680579	0.558838	0.0121785				0.0121785	vacuolar H+-ATPase%2C a subunit
XP_009692132.1	0.011912	0.63155	0.0188615	0.0235463	0.478295	0.0492297	0.034046	conserved hypothetical protein
XP_009692136.1	0.0978149	0.712071	0.137367	0.0745279	0.821694	0.0907003	0.114034	conserved hypothetical protein
XP_009692137.1	0.0750422	0.504127	0.148856	0.0747481	0.632734	0.118135	0.133495	conserved hypothetical protein
XP_009692138.1	0.00252951	0.0624803	0.0404848	0.0471922	0.779959	0.060506	0.050495	uncharacterized protein
XP_009692139.1	0.126224	0.925364	0.136405	0.111864	0.828828	0.134966	0.135686	conserved hypothetical protein
XP_009692140.1	0.104608	0.320699	0.326188	0.114014	0.661277	0.172414	0.249301	conserved hypothetical protein
XP_009692141.1	0.0214791	0.919069	0.0233705	0.0123799	0.745749	0.0166006	0.019986	enolase
XP_009692142.1	0.0073167	0.260519	0.0280851	0.00202914	0.295258	0.00687243	0.017479	predicted protein
XP_009692143.1	0.0372582	0.612026	0.0608768	0.0242146	0.425212	0.056947	0.058912	uncharacterized protein
XP_009692144.1	0.059303	0.687736	0.0862292	0.0608036	0.586964	0.10359	0.09491	uncharacterized protein
XP_009692145.1	0.0696948	0.378297	0.184233	0.0674313	0.456678	0.147656	0.165944	conserved hypothetical protein
XP_009692146.1	0.0945669	0.591396	0.159905	0.0852292	0.68679	0.124098	0.142002	uncharacterized protein
XP_009692147.1	0.0674084	0.797105	0.0845665	0.0915418	0.495585	0.184715	0.134641	conserved hypothetical protein
XP_009692148.1	0.0826707	0.807785	0.102342	0.0708363	0.885837	0.0799654	0.091154	conserved hypothetical protein
XP_009692149.1	0.0520207	0.851961	0.06106	0.0423	0.897711	0.0471198	0.05409	conserved hypothetical protein
XP_009692150.1	0.0112615	0.0451819	0.249247	0.00832967	0.0575737	0.144678	0.196962	hypothetical protein
XP_009692151.1	0.0251597	0.57328	0.0438873				0.0438873	phosphoglycerate mutase
XP_009692152.1	0.0238582	0.276431	0.086308	0.0405978	0.335727	0.120925	0.103617	uncharacterized protein
XP_009692153.1	0.0330079	0.463277	0.0712487	0.0144535	0.215737	0.0669958	0.069122	Pbj2
XP_009692154.1	0.0599693	0.36089	0.16617	0.0571133	0.278621	0.204985	0.185578	uncharacterized protein
XP_009692155.1	0.038901	0.447413	0.0869463	0.0208097	0.270852	0.0768308	0.081889	conserved hypothetical protein

protein_id	Chitose_ka	Chitose_ks	Chitose_kaks	Buffeli_ka	Buffeli_ks	Buffeli_kaks	mean_kaks	function
XP_009692156.1	0.050284	0.306778	0.16391	0.0581922	0.272807	0.21331	0.18861	conserved hypothetical protein
XP_009692157.1	0.010998	0.730797	0.0150494	0.0110455	1.03919	0.0106289	0.012839	heat-shock protein HSP70
XP_009692158.1	0.0986242	0.709076	0.139088	0.0965609	0.657039	0.146964	0.143026	uncharacterized protein
XP_009692159.1	0.0221612	0.296413	0.0747646	0.0157852	0.273459	0.0577242	0.066244	succinyl-CoA ligase [GDP-forming] alpha-chain%2C mitochondrial
XP_009692160.1	0.00968861	0.529953	0.018282	0.0130324	0.570729	0.0228346	0.020558	uncharacterized protein
XP_009692161.1	0.0156181	0.58534	0.0266822	0.0224075	1.27414	0.0175864	0.022134	conserved hypothetical protein
XP_009692162.1	0.0156404	0.608195	0.0257161	0.00909257	0.478593	0.0189985	0.022357	RuvB-like DNA repair helicase
XP_009692163.1	0.0971279	0.610152	0.159187	0.122743	0.795359	0.154324	0.156755	uncharacterized protein
XP_009692164.1	0.077461	0.898834	0.0861794	0.0743204	1.07401	0.0691988	0.077689	uncharacterized protein
XP_009692165.1	0.0322028	0.774192	0.0415954	0.0398444	0.670063	0.0594636	0.050529	PRL1 protein
XP_009692166.1	0.137076	0.481007	0.284978	0.148966	0.496439	0.300068	0.292523	conserved hypothetical protein
XP_009692167.1	0.0816401	0.634831	0.128601				0.128601	uncharacterized protein
XP_009692168.1	0.0226745	0.390268	0.0580998	0.0165662	0.489039	0.0338751	0.045987	uncharacterized protein
XP_009692169.1	0.0662002	0.316869	0.20892	0.0285592	0.165485	0.172579	0.19075	uncharacterized protein
XP_009692170.1	3.02118E-05	0.0302118	0.001	0.000346536	0.346536	0.001	0.001	RING-box protein 1a
XP_009692171.1	0.0442153	0.569182	0.0776821	0.0474163	0.57363	0.08266	0.080171	uncharacterized protein
XP_009692172.1	0.045166	0.219186	0.206062				0.206062	conserved hypothetical protein
XP_009692173.1	0.0421684	0.514941	0.0818898	0.0406517	0.5589	0.0727352	0.077313	uncharacterized protein
XP_009692174.1	0.0357468	0.404623	0.088346	0.0399223	0.341145	0.117024	0.102685	histone H4
XP_009692176.1	0.0222105	0.731411	0.0303666	0.00895333	0.5917	0.0151315	0.022749	protein kinase
XP_009692177.1	0.0159094	0.366131	0.0434529	0.0109677	0.451035	0.0243168	0.033885	centrin
XP_009692178.1	0.00931701	0.62279	0.0149601	0.00704338	0.595734	0.011823	0.013392	40S ribosomal protein S4
XP_009692179.1	0.0245623	0.439952	0.0558295	0.0300309	0.479486	0.0626314	0.05923	uncharacterized protein
XP_009692180.1	0.0698257	0.339419	0.205721	0.0494822	0.25042	0.197597	0.201659	conserved hypothetical protein
XP_009692182.1	0.0524382	0.349342	0.150105	0.0450212	0.393923	0.114289	0.132197	conserved hypothetical protein
XP_009692183.1	0.124183	0.5476	0.226777				0.226777	conserved hypothetical protein
XP_009692184.1	0.0928665	0.5052	0.183821	0.0962752	0.391167	0.246123	0.214972	conserved hypothetical protein
XP_009692185.1	0.0701755	0.388745	0.180518	0.0772045	0.32532	0.237318	0.208918	conserved hypothetical protein
XP_009692186.1	0.0442349	0.471699	0.0937777	0.0513546	0.493205	0.104124	0.098951	conserved hypothetical protein
XP_009692187.1	0.0605246	0.813788	0.0743739	0.0376943	0.585067	0.0644274	0.069401	conserved hypothetical protein
XP_009692188.1	0.110939	0.74745	0.148424	0.100424	0.635105	0.158122	0.153273	conserved hypothetical protein
XP_009692189.1	0.0453981	0.454274	0.0999355	0.0399429	0.545776	0.0731854	0.08656	conserved hypothetical protein
XP_009692190.1	0.107388	0.602052	0.17837	0.0944068	0.535822	0.176191	0.177281	conserved hypothetical protein
XP_009692191.1	0.0656784	0.619993	0.105934	0.0590418	0.643042	0.0918164	0.098875	DEAD-box family helicase
XP_009692193.1	0.0766942	0.504259	0.152093	0.0625185	0.532815	0.117336	0.134715	conserved hypothetical protein
XP_009692196.1	0.0739218	0.384493	0.192258				0.192258	uncharacterized protein
XP_009692199.1	0.0163285	0.434428	0.0375862	0.0186397	0.480578	0.0387861	0.038186	conserved hypothetical protein
XP_009692200.1	0.0801272	0.748691	0.107023	0.0673974	0.366853	0.183718	0.14537	conserved hypothetical protein
XP_009692201.1	0.10363	0.425729	0.243419	0.078284	0.425762	0.183868	0.213643	conserved hypothetical protein
XP_009692202.1	0.0311726	0.216312	0.14411	0.0418872	0.316565	0.132318	0.138214	30S ribosomal protein S15
XP_009692203.1	0.0366873	0.903927	0.0405865	0.0434065	0.927752	0.0467867	0.043687	conserved hypothetical protein
XP_009692204.1	0.0226172	0.794617	0.028463	0.0271362	0.884407	0.0306829	0.029573	DNA-directed RNA polymerase III largest subunit RPC1
XP_009692205.1	0.0845959	0.994731	0.085044	0.0691083	1.0152	0.0680736	0.076559	uncharacterized protein
XP_009692206.1	0.0436982	0.764488	0.0571602	0.0336726	0.732678	0.0459582	0.051559	heat shock protein HSP90
XP_009692207.1	0.0869563	0.796272	0.109204	0.0702989	0.690515	0.101806	0.105505	serine/threonine protein kinase
XP_009692208.1	0.0226366	0.438385	0.0516364	0.0141885	0.400805	0.0354	0.043518	Rab GTPase
XP_009692209.1	0.0581627	0.558591	0.104124	0.0579803	0.609849	0.0950732	0.099599	uncharacterized protein
XP_009692210.1	0.0831337	0.508902	0.163359	0.0804981	0.415433	0.193769	0.178564	conserved hypothetical protein
XP_009692211.1	0.0506303	0.287871	0.175878				0.175878	uncharacterized protein
XP_009692212.1	0.048046	0.938294	0.0512057	0.0393348	0.820146	0.0479607	0.049583	vacuolar protein sorting/secretion protein
XP_009692213.1	0.078861	0.399808	0.197247				0.197247	conserved hypothetical protein
XP_009692214.1	0.0973199	0.253354	0.384126	0.0741141	0.371399	0.199554	0.29184	uncharacterized protein
XP_009692215.1	0.030675	0.390334	0.0785864	0.00498434	4.88704	0.00101991	0.039803	uncharacterized protein

protein_id	Chitose_ka	Chitose_ks	Chitose_kaks	Buffeli_ka	Buffeli_ks	Buffeli_kaks	mean_kaks	function
XP_009692216.1	0.0382067	0.338333	0.112926	0.0314173	0.298316	0.105316	0.109121	conserved hypothetical protein
XP_009692217.1	0.0328567	0.329813	0.0996222				0.0996222	conserved hypothetical protein
XP_009692218.1	0.0748147	0.560799	0.133407	0.0777905	0.500716	0.155359	0.144383	conserved hypothetical protein
XP_009692219.1	0.060393	0.557168	0.108393	0.0483912	0.434701	0.111321	0.109857	replication factor C
XP_009692220.1	0.0941843	0.583063	0.161534	0.083698	0.821352	0.101903	0.131719	uncharacterized protein
XP_009692221.1	0.0956068	0.534259	0.178952	0.0819188	0.571389	0.143368	0.16116	uncharacterized protein
XP_009692222.1	0.088202	0.760618	0.115961	0.0889945	0.692181	0.128571	0.122266	uncharacterized protein
XP_009692223.1	0.0518971	0.380191	0.136503	0.0583682	0.36126	0.161568	0.149035	uncharacterized protein
XP_009692224.1	0.0584641	0.367921	0.158904	0.0773331	0.449073	0.172206	0.165555	conserved hypothetical protein
XP_009692225.1	0.0434998	0.433121	0.100433	0.0378383	0.327727	0.115457	0.107945	uncharacterized protein
XP_009692226.1	0.039991	0.446118	0.0896422	0.0415007	0.466	0.0890573	0.08935	mRNA cleavage factor subunit
XP_009692227.1	0.000179413	0.179413	0.001				0.001	conserved hypothetical protein
XP_009692228.1	0.0146573	0.209512	0.0699594	0.034863	0.278854	0.125022	0.097491	acetyltransferase
XP_009692229.1	0.068979	0.361072	0.19104	0.076697	0.245037	0.313002	0.252021	uncharacterized protein
XP_009692230.1	0.0282593	0.366357	0.0771361	0.0445169	0.529363	0.0840952	0.080616	hypothetical protein
XP_009692231.1	0.0321312	0.51539	0.0623435	0.024243	0.499368	0.0485472	0.055445	phenylalanyl-tRNA synthetase
XP_009692232.1	0.0574139	0.546035	0.105147	0.064183	0.556428	0.115348	0.110247	uncharacterized protein
XP_009692233.1	0.0263158	0.461483	0.0570244	0.0242416	0.453758	0.053424	0.055224	isocitrate dehydrogenase
XP_009692234.1	0.0693846	0.701209	0.0989498	0.0784276	0.621606	0.126169	0.112559	conserved hypothetical protein
XP_009692235.1	0.0026503	0.359809	0.00736585	0.000317305	0.317305	0.001	0.004183	proteasome subunit beta type
XP_009692236.1	0.0238311	0.29634	0.0804182				0.0804182	conserved hypothetical protein
XP_009692237.1	0.0414891	0.436591	0.0950298				0.0950298	conserved hypothetical protein
XP_009692238.1	0.0654807	0.364292	0.179748	0.129745	0.185732	0.698561	0.439155	uncharacterized protein
XP_009692239.1	0.0361099	0.550286	0.0656202	0.0214859	0.499479	0.0430165	0.054318	SWI/SNF-related chromatin remodelling factor
XP_009692240.1	0.0589023	0.372662	0.158058	0.0493377	0.392126	0.125821	0.141939	translation elongation factor 1-alpha
XP_009692241.1	0.11316	0.645366	0.175342	0.0845606	0.671047	0.126013	0.150677	uncharacterized protein
XP_009692242.1	0.0283937	0.397072	0.0715079	0.0178229	0.310698	0.057364	0.064436	uncharacterized protein
XP_009692243.1	0.0555791	0.787808	0.0705491	0.054502	0.854118	0.0638109	0.06718	ankyrin repeat containing protein
XP_009692244.1	0.0552431	0.499268	0.110648	0.0424117	0.276285	0.153507	0.132078	uncharacterized protein
XP_009692245.1	0.0108373	0.706209	0.0153457	0.0132561	0.68908	0.0192373	0.017292	pyruvate kinase
XP_009692246.1	0.0563283	0.477495	0.117966	0.0719555	0.579919	0.124078	0.121022	uncharacterized protein
XP_009692249.1	0.0737441	0.498337	0.14798	0.0736206	0.514595	0.143065	0.145522	conserved hypothetical protein
XP_009692250.1	0.0907442	0.451755	0.20087	0.0927823	0.521827	0.177803	0.189336	conserved hypothetical protein
XP_009692251.1	4.92778E-05	0.0492778	0.001				0.001	hypothetical protein
XP_009692252.1	0.0602004	0.707012	0.0851477	0.0502578	0.502845	0.0999469	0.092547	conserved hypothetical protein
XP_009692254.1	0.0913202	0.536771	0.170129	0.10166	0.56008	0.181511	0.17582	conserved hypothetical protein
XP_009692255.1	0.0830902	0.418448	0.198567	0.101924	0.342679	0.297433	0.248	papain-family cysteine protease
XP_009692256.1	0.0151487	0.0913844	0.16577	NA	NA	NA	NA	uncharacterized protein
XP_009692257.1	0.0383123	0.0927391	0.413119				0.413119	conserved hypothetical protein
XP_009692258.1	0.0586886	0.881249	0.0665971	0.0556958	1.03121	0.0540101	0.060304	conserved hypothetical protein
XP_009692259.1	0.0634852	0.316654	0.200488	0.0370427	0.325724	0.113724	0.157106	uncharacterized protein
XP_009692260.1	0.0279081	0.793262	0.0351814	0.0372049	0.654218	0.0568692	0.046025	mitochondrial carrier protein
XP_009692261.1	0.0392938	0.690017	0.0569462	0.0312545	0.653345	0.0478376	0.052392	leucyl-tRNA synthetase
XP_009692262.1	0.102706	0.663644	0.154761	0.0908869	0.367167	0.247535	0.201148	conserved hypothetical protein
XP_009692263.1	0.0824252	0.503919	0.163568	0.0928045	0.650316	0.142707	0.153137	uncharacterized protein
XP_009692264.1	0.0831981	0.466302	0.178421	0.0856375	0.407408	0.210201	0.194311	uncharacterized protein
XP_009692267.1	0.0241361	0.395005	0.0611033	0.0126573	0.398164	0.0317892	0.046446	mRNA turnover/deadenylation component
XP_009692268.1	0.00973886	0.111145	0.0876228				0.0876228	conserved hypothetical protein
XP_009692269.1	0.0197826	1.13205	0.017475	0.0189725	0.790337	0.0240056	0.02074	conserved hypothetical protein
XP_009692270.1	0.0308302	0.252226	0.122233				0.122233	cyclin
XP_009692271.1	0.0919302	0.48551	0.189348	0.0848861	0.380765	0.222936	0.206142	conserved hypothetical protein
XP_009692272.1	0.0376555	0.537758	0.0700231	0.030423	0.431541	0.0704985	0.070261	small GTPase
XP_009692273.1	0.0566902	0.219353	0.258442				0.258442	conserved hypothetical protein

protein_id	Chitose_ka	Chitose_ks	Chitose_kaks	Buffeli_ka	Buffeli_ks	Buffeli_kaks	mean_kaks	function
XP_009692275.1	0.090291	0.958651	0.0941855	0.0956313	0.937054	0.102055	0.09812	uncharacterized protein
XP_009692276.1	0.127975	0.638391	0.200465	0.100503	0.609843	0.164802	0.182634	uncharacterized protein
XP_009692278.1	0.0316219	0.394983	0.0800587	0.031952	0.56855	0.0561991	0.068129	uncharacterized protein
XP_009692279.1	0.0278747	0.385189	0.0723662	0.0322622	0.407918	0.0790899	0.075728	conserved hypothetical protein
XP_009692280.1	0.0619456	0.355201	0.174396	0.0591713	0.452833	0.130669	0.152533	conserved hypothetical protein
XP_009692281.1	0.0463822	0.616549	0.0752288	0.0729495	0.527699	0.138241	0.106735	sybindin
XP_009692283.1	0.0573688	0.5742	0.0999108				0.0999108	uncharacterized protein
XP_009692284.1	0.0464819	0.765457	0.0607244	0.0389906	0.310684	0.125499	0.093112	nuclear RNA binding protein B
XP_009692285.1	0.0382941	0.908634	0.0421447	0.0169487	0.741994	0.0228421	0.032493	DEAD-box family RNA-dependent helicase
XP_009692286.1	0.123048	0.912749	0.13481				0.13481	conserved hypothetical protein
XP_009692287.1	0.0121434	0.203348	0.0597172				0.0597172	cytosol aminopeptidase
XP_009692288.1	0.0599231	0.439003	0.136498	0.0483221	0.506991	0.0953117	0.115905	conserved hypothetical protein
XP_009692289.1	0.0532598	0.590979	0.0901213				0.0901213	uncharacterized protein
XP_009692290.1	0.0668777	0.815705	0.0819875	0.0559138	0.727583	0.0768487	0.079418	uncharacterized protein
XP_009692291.1	0.0134322	0.555528	0.0241791	0.0121916	0.573366	0.0212631	0.022721	conserved hypothetical protein
XP_009692292.1	0.181472	0.429083	0.42293	0.118935	0.347656	0.342106	0.382518	uncharacterized protein
XP_009692293.1	0.0472003	0.631376	0.0747577	0.0680813	0.613458	0.11098	0.092869	predicted protein
XP_009692294.1	0.0335789	0.459591	0.0730627	0.0420313	0.362648	0.115901	0.094482	mitochondrial carrier protein
XP_009692295.1	0.0478978	0.115518	0.414636	0.0420474	0.244706	0.171829	0.293233	mitochondrial carrier protein
XP_009692296.1	0.019418	0.758219	0.02561	0.00622722	0.461935	0.0134807	0.019545	serine/threonine protein kinase
XP_009692297.1	0.00394962	0.439158	0.00899364				0.00899364	predicted protein
XP_009692298.1	0.0214053	1.21217	0.0176586	0.0225078	1.06626	0.0211091	0.019384	uncharacterized protein
XP_009692299.1	0.0296623	0.659619	0.0449689	0.0240983	0.658152	0.0366151	0.040792	uncharacterized protein
XP_009692301.1	0.0613722	0.494827	0.124028	0.0548755	0.451869	0.121441	0.122734	prohibitin-like protein
XP_009692302.1	0.023076	0.303571	0.0760152	0.0229091	0.399873	0.0572909	0.066653	uncharacterized protein
XP_009692303.1	0.0845336	0.514528	0.164293				0.164293	uncharacterized protein
XP_009692304.1	0.0481659	0.196166	0.245537				0.245537	uncharacterized protein
XP_009692305.1	0.0119538	0.719969	0.0166032	0.0119942	0.813712	0.0147401	0.015672	small GTPase
XP_009692306.1	0.0396499	0.827411	0.0479204	0.0425894	0.945212	0.045058	0.046489	importin-alpha
XP_009692307.1	0.0484978	0.803479	0.0603598	0.0133494	0.350346	0.0381035	0.049232	uncharacterized protein
XP_009692309.1				0.10585	0.70879	0.14934	0.14934	uncharacterized protein
XP_009692310.1	0.0817769	0.768114	0.106464	0.110383	0.717315	0.153883	0.130173	uncharacterized protein
XP_009692311.1	0.114281	0.744606	0.153479	0.0967833	0.653182	0.148172	0.150826	uncharacterized protein
XP_009692312.1	0.0133209	0.579868	0.0229722	0.0266314	0.644022	0.0413517	0.032162	conserved hypothetical protein
XP_009692314.1	0.0775744	0.82557	0.0939646	0.0514632	0.538469	0.0955732	0.094769	uncharacterized protein
XP_009692315.1	0.0856582	0.859185	0.099697	0.0915321	0.794774	0.115167	0.107432	uncharacterized protein
XP_009692316.1	0.035348	1.69111	0.0209022	NA	NA	NA	NA	RNA polymerase small subunit
XP_009692317.1	0.0539653	0.313958	0.171887	0.0753047	0.424216	0.177515	0.174701	uncharacterized protein
XP_009692318.1	0.0538439	0.80273	0.0670759	0.0504827	0.626562	0.0805708	0.073823	conserved hypothetical protein
XP_009692319.1	0.0461676	0.728934	0.0633358	0.0479951	0.713775	0.0672412	0.065288	conserved hypothetical protein
XP_009692321.1				0.0762359	1.04955	0.0726369	0.0726369	conserved hypothetical protein
XP_009692324.1	0.0726696	0.937448	0.0775186	0.0958046	0.963612	0.0994224	0.08847	uncharacterized protein
XP_009692325.1	0.0450654	1.02577	0.0439333	0.0351981	0.991445	0.0355019	0.039718	myosin light chain kinase
XP_009692326.1	0.065937	0.658113	0.100191	0.0529839	0.554772	0.0955056	0.097848	conserved hypothetical protein
XP_009692327.1	0.108352	0.883934	0.12258	0.0952798	0.736802	0.129315	0.125947	uncharacterized protein
XP_009692328.1	0.0433456	0.630097	0.068792	0.0333703	0.522969	0.0638093	0.066301	translation elongation factor EF-1 subunit alpha
XP_009692329.1	0.0312149	0.501411	0.062254	0.0249943	0.499976	0.0499909	0.056122	elongation factor subunit
XP_009692330.1	0.0698112	0.728905	0.0957755	0.0638552	1.43183	0.0445968	0.070186	uncharacterized protein
XP_009692331.1	0.0357082	0.387033	0.0922613	0.0508454	0.308826	0.164641	0.128451	uncharacterized protein
XP_009692332.1	0.0605038	0.592839	0.102058	0.0530642	0.691727	0.0767126	0.089385	DNA replication protein
XP_009692333.1	0.0561437	1.03233	0.0543856	0.0384217	0.93345	0.041161	0.047773	conserved hypothetical protein
XP_009692334.1	0.0789695	1.37773	0.0573185	0.0818255	1.1976	0.0683245	0.062822	silencing mediator for retinoid and thyroid hormone receptor
XP_009692335.1	0.0359024	0.337802	0.106282	0.0332136	0.450075	0.0737958	0.090039	uncharacterized protein

protein_id	Chitose_ka	Chitose_ks	Chitose_kaks	Buffeli_ka	Buffeli_ks	Buffeli_kaks	mean_kaks	function
XP_009692336.1				0.0169901	0.0727275	0.233613	0.233613	conserved hypothetical protein
XP_009692337.1	0.0148819	0.387254	0.0384294	0.00907383	0.329069	0.0275743	0.033002	bifunctional dihydrofolate reductase/thymidilate synthase
XP_009692338.1	0.0499512	0.454791	0.109833				0.109833	conserved hypothetical protein
XP_009692339.1	0.019108	0.589702	0.0324027	0.015398	0.573046	0.0268705	0.029637	conserved hypothetical protein
XP_009692340.1	0.0501948	0.339525	0.147839	0.0623154	0.525707	0.118536	0.133188	conserved hypothetical protein
XP_009692341.1	0.0634332	0.725833	0.0873938	0.050745	0.680979	0.0745178	0.080956	DNA helicase
XP_009692342.1	0.0552206	0.752657	0.0733676	0.0471229	0.586464	0.0803509	0.076859	chromosome maintenance protein
XP_009692343.1	0.032525	0.60653	0.0536247	0.0290513	0.346316	0.0838866	0.068756	conserved hypothetical protein
XP_009692344.1	0.027326	0.524807	0.0520686	0.0284302	0.467189	0.0608537	0.056461	conserved hypothetical protein
XP_009692345.1	0.0306915	0.76737	0.0399957	0.0406356	0.932482	0.0435779	0.041787	uncharacterized protein
XP_009692346.1	0.0173637	0.326189	0.053232	0.0287455	0.402291	0.0714547	0.062343	uncharacterized protein
XP_009692347.1	0.0155142	0.779878	0.0198931	0.00602538	0.537522	0.0112095	0.015551	metallopeptidase
XP_009692348.1	0.0370216	0.843587	0.0438859	0.043939	0.977831	0.0449352	0.044411	60S ribosomal protein L6
XP_009692349.1	0.0391929	1.03042	0.0380358	0.0259032	1.22157	0.0212049	0.02962	elongation factor 1-gamma
XP_009692350.1	0.036577	0.569715	0.0642022	0.0260512	0.570643	0.0456524	0.054927	uncharacterized protein
XP_009692351.1	0.0276055	0.254027	0.108671	0.0330807	0.308331	0.107289	0.10798	conserved hypothetical protein
XP_009692352.1	0.0551263	0.628144	0.0877606	0.0419048	0.487344	0.0859861	0.086873	conserved hypothetical protein
XP_009692353.1	0.029941	0.158033	0.18946				0.18946	uncharacterized protein
XP_009692354.1	0.0319155	0.574532	0.0555504	0.0297659	0.517757	0.0574901	0.05652	uncharacterized protein
XP_009692355.1	0.134367	0.214868	0.625346	NA	NA	NA	NA	hypothetical protein
XP_009692356.1	0.0642683	0.115417	0.556834	0.169689	0.139977	1.21226	0.884547	hypothetical protein
XP_009692357.1	0.113749	0.945368	0.120322	0.0869063	1.04552	0.0831227	0.101722	conserved hypothetical protein
XP_009692358.1	0.0858793	0.700844	0.122537	0.0916834	0.688682	0.133129	0.127833	uncharacterized protein
XP_009692359.1	0.0339168	0.742235	0.0456955	0.0354325	0.837479	0.0423085	0.044002	conserved hypothetical protein
XP_009692360.1	0.0235404	0.517946	0.0454495	0.0179236	0.688472	0.0260339	0.035742	uncharacterized protein
XP_009692361.1	0.000456793	0.456793	0.001	0.000441675	0.441675	0.001	0.001	putative 60S Ribosomal protein L44
XP_009692362.1	0.101518	0.575573	0.176377	0.0899781	0.603363	0.149128	0.162753	uncharacterized protein
XP_009692363.1	0.0709855	0.627352	0.113151	0.0644303	0.780971	0.0825002	0.097826	uncharacterized protein
XP_009692364.1	0.000518429	0.518429	0.001	0.0141156	0.798966	0.0176673	0.009334	uncharacterized protein
XP_009692365.1	0.0177217	0.760774	0.0232944	0.0107406	0.588571	0.0182486	0.020771	dimethyladenosine transferase
XP_009692366.1	0.011887	0.373415	0.0318334	0.0107895	0.41078	0.026266	0.02905	phosphorylase phosphatase
XP_009692367.1	0.0643933	0.650541	0.0989842	0.0694729	0.574398	0.120949	0.109967	uncharacterized protein
XP_009692368.1	0.0482535	0.612626	0.0787651	0.0445814	0.6192	0.0719984	0.075382	RNA polymerases I and III subunit
XP_009692369.1	0.0916612	0.583922	0.156975	0.0476451	0.381105	0.125019	0.140997	lysyl-tRNA synthetase
XP_009692371.1	0.0797384	0.285118	0.279668	0.0774765	0.363573	0.213097	0.246383	conserved hypothetical protein
XP_009692372.1	0.0542147	0.469524	0.115467				0.115467	conserved hypothetical protein
XP_009692373.1	0.0322	0.675679	0.0476558	0.0303308	0.72058	0.0420923	0.044874	pre-mRNA splicing complex subunit
XP_009692374.1	0.0921065	0.778151	0.118366	0.0754087	0.350274	0.215285	0.166826	triosephosphate isomerase
XP_009692375.1	0.0504786	0.495377	0.101899	0.0403501	0.321657	0.125444	0.113672	conserved hypothetical protein
XP_009692376.1	0.0683732	0.63327	0.107968	0.0662975	0.733867	0.0903399	0.099154	conserved hypothetical protein
XP_009692377.1	0.211855	0.205755	1.02964	0.0401281	0.118478	0.338696	0.684168	hypothetical protein
XP_009692378.1	0.0385817	0.436224	0.0884446	0.0253826	0.411555	0.0616748	0.07506	vesicle-associated membrane protein
XP_009692379.1	0.000250823	0.250823	0.001	0.000994045	0.994045	0.001	0.001	RNA polymerase common subunit
XP_009692380.1	0.0384455	0.687402	0.0559287	0.044909	0.669056	0.0671229	0.061526	uncharacterized protein
XP_009692381.1	0.0319333	0.371331	0.0859969	0.035254	0.407439	0.0865257	0.086261	uncharacterized protein
XP_009692382.1	0.106859	0.588184	0.181676	0.103029	0.352702	0.292113	0.236895	uncharacterized protein
XP_009692383.1	0.0318937	0.53924	0.0591457	0.0193776	0.433722	0.0446774	0.051912	branched-chain alpha-keto acid dihydrolipoyl acyltransferase precursor
XP_009692384.1	0.00888369	0.320223	0.0277422	0.0104977	0.167092	0.0628257	0.045284	membrane skeletal protein IMC1
XP_009692385.1	0.0885527	0.552611	0.160244	0.0795592	0.616863	0.128974	0.144609	conserved hypothetical protein
XP_009692386.1	0.0594096	0.754848	0.078704	0.0608378	0.664012	0.0916215	0.085163	uncharacterized protein
XP_009692387.1	0.0537723	0.264963	0.202942	0.0363149	0.326648	0.111174	0.157058	uncharacterized protein
XP_009692388.1	0.0535482	0.398007	0.134541	0.0447652	0.411901	0.10868	0.12161	conserved hypothetical protein
XP_009692389.1	0.0285233	0.575909	0.0495274	0.0127916	0.341515	0.0374553	0.043491	conserved hypothetical protein

protein_id	Chitose_ka	Chitose_ks	Chitose_kaks	Buffeli_ka	Buffeli_ks	Buffeli_kaks	mean_kaks	function
XP_009692390.1	0.10194	0.515109	0.197901	0.0766682	0.398617	0.192336	0.195119	uncharacterized protein
XP_009692391.1	0.0711411	0.713276	0.0997385	0.0561443	0.638649	0.087911	0.093825	conserved hypothetical protein
XP_009692392.1	0.0368351	0.566548	0.0650167	0.028128	0.607116	0.0463306	0.055674	conserved hypothetical protein
XP_009692393.1	0.0293353	0.790733	0.0370989	0.021689	1.49701	0.0144883	0.025794	uncharacterized protein
XP_009692394.1	0.0361349	0.440963	0.0819455	0.0475766	0.792151	0.0600601	0.071003	uncharacterized protein
XP_009692395.1	0.0138923	0.229375	0.0605658	0.0240791	0.370377	0.0650124	0.062789	uncharacterized protein
XP_009692396.1	0.0519541	0.67361	0.0771279	0.0232746	0.709741	0.0327931	0.05496	conserved hypothetical protein
XP_009692397.1	0.0530664	0.51386	0.10327	0.059049	0.647822	0.09115	0.09721	conserved hypothetical protein
XP_009692398.1	0.00498838	0.597096	0.00835441	0.00775825	0.751301	0.0103264	0.00934	vacuolar ATP synthase subunit beta
XP_009692399.1	0.0143134	0.328046	0.0436324	0.00466979	0.369486	0.0126386	0.028136	conserved hypothetical protein
XP_009692400.1	0.192508	0.64212	0.2998				0.2998	uncharacterized protein
XP_009692401.1	0.0190931	0.429356	0.0444692	0.026702	0.400243	0.0667146	0.055592	uncharacterized protein
XP_009692402.1	0.0381749	0.530048	0.0720217	0.0357754	0.641547	0.0557643	0.063893	conserved hypothetical protein
XP_009692403.1	0.12124	0.673973	0.179889	0.126724	0.834282	0.151896	0.165892	uncharacterized protein
XP_009692404.1	0.202852	0.197017	1.02961	0.13124	0.242068	0.542164	0.785887	hypothetical protein
XP_009692405.1	0.0102353	0.442488	0.0231312	0.00832033	0.493906	0.016846	0.019989	vacuolar ATP synthase
XP_009692406.1	0.0636483	0.920556	0.0691411	0.0609777	1.06039	0.0575052	0.063323	conserved hypothetical protein
XP_009692407.1	0.0211136	0.41255	0.0511782	0.0189992	0.444519	0.042741	0.04696	uncharacterized protein
XP_009692408.1	0.04398	0.493053	0.0891992	0.0659545	0.714088	0.0923619	0.090781	conserved hypothetical protein
XP_009692409.1	0.0723901	0.580146	0.124779	0.0705241	0.544212	0.129589	0.127184	uncharacterized protein
XP_009692410.1	0.0356728	0.386884	0.0922055	0.0380847	0.363306	0.104828	0.098517	conserved hypothetical protein
XP_009692411.1	0.0118216	0.243359	0.0485768	0.00914466	0.210679	0.0434056	0.045991	60S ribosomal subunit biogenesis protein
XP_009692412.1	0.0368959	0.225884	0.16334				0.16334	conserved hypothetical protein
XP_009692413.1	0.0184922	0.71775	0.0257641	0.0202874	0.574629	0.0353052	0.030535	conserved hypothetical protein
XP_009692414.1	0.1596	0.89298	0.178727	0.150326	0.939753	0.159963	0.169345	uncharacterized protein
XP_009692415.1	0.107373	0.920155	0.11669	0.105596	0.562476	0.187735	0.152213	uncharacterized protein
XP_009692416.1	0.139942	0.716381	0.195345	0.222643	0.913686	0.243676	0.21951	uncharacterized protein
XP_009692417.1	0.0130477	0.535516	0.0243646	0.0129051	0.572157	0.0225551	0.02346	26S proteasome regulatory subunit
XP_009692418.1	0.00545677	0.356437	0.0153092	0.00900734	0.316948	0.028419	0.021864	serine/threonine protein phosphatase
XP_009692419.1	0.0525058	0.54028	0.0971826	0.0686631	0.428894	0.160093	0.128638	conserved hypothetical protein
XP_009692420.1	0.00594127	0.678645	0.00875461	0.00205517	0.570878	0.00360002	0.006177	40S ribosomal protein S3
XP_009692421.1	0.105017	0.238925	0.439541	0.083331	0.188023	0.443196	0.441368	conserved hypothetical protein
XP_009692422.1	0.0262007	0.336176	0.0779375	0.00628098	0.230215	0.0272832	0.05261	conserved hypothetical protein
XP_009692423.1	0.020626	0.282072	0.0731232				0.0731232	conserved hypothetical protein
XP_009692424.1	0.0614002	0.354859	0.173027	0.060311	0.301002	0.200367	0.186697	conserved hypothetical protein
XP_009692425.1	0.0579527	0.449614	0.128894	0.0548417	0.429112	0.127803	0.128349	tRNA 3' processing endoribonuclease
XP_009692426.1	0.0610926	0.608092	0.100466	0.0469194	0.604707	0.0775904	0.089028	conserved hypothetical protein
XP_009692427.1	0.0705757	0.788219	0.0895382	0.0641252	0.726939	0.0882125	0.088875	conserved hypothetical protein
XP_009692428.1	0.0371506	0.423057	0.0878147	0.0449491	0.438397	0.102531	0.095173	conserved hypothetical protein
XP_009692429.1	0.0634388	0.461536	0.137452	0.057154	0.456896	0.125092	0.131272	conserved hypothetical protein
XP_009692430.1	0.0748826	0.52659	0.142203	0.0657108	0.60754	0.108159	0.125181	serine/threonine protein phosphatase 2C
XP_009692431.1	0.0510879	0.918097	0.0556454	0.0490613	1.07522	0.0456291	0.050637	chromosome segregation protein
XP_009692432.1	0.131894	0.393856	0.334879	0.098111	0.392985	0.249656	0.292268	conserved hypothetical protein
XP_009692433.1	6.93153E-05	0.0693153	0.001	3.12145E-05	0.0312145	0.001	0.001	histone H2B
XP_009692434.1	0.115159	0.326604	0.352595	0.0836194	0.34064	0.245477	0.299036	conserved hypothetical protein
XP_009692435.1	0.0354888	0.412781	0.0859749	0.0401149	0.416038	0.0964212	0.091198	DNA-binding chaperone
XP_009692436.1	0.11367	0.204574	0.555643	0.094062	0.33	0.285036	0.420339	hypothetical protein
XP_009692437.1	0.0513743	0.761934	0.0674262	0.0537639	0.822092	0.0653988	0.066413	uncharacterized protein
XP_009692438.1	0.109605	0.548821	0.19971	0.0759741	0.508495	0.14941	0.17456	conserved hypothetical protein
XP_009692439.1	0.0846763	0.713595	0.118662	0.0685362	0.664743	0.103102	0.110882	conserved hypothetical protein
XP_009692440.1	0.206522	0.173556	1.18994	0.109192	0.0612308	1.78329	1.486615	hypothetical protein
XP_009692441.1	0.0364538	0.315436	0.115566	0.0275573	0.290314	0.0949226	0.105244	uncharacterized protein
XP_009692442.1	0.0195323	0.417739	0.0467571	0.0193174	0.4306	0.0448616	0.045809	uncharacterized protein

protein_id	Chitose_ka	Chitose_ks	Chitose_kaks	Buffeli_ka	Buffeli_ks	Buffeli_kaks	mean_kaks	function
XP_009692443.1	0.0211963	0.469104	0.0451846	0.0109736	0.473892	0.0231564	0.03417	26S proteasome regulatory subunit
XP_009692445.1	0.0158261	0.256988	0.0615829	0.0246942	0.359419	0.0687057	0.065144	Ser/Arg-rich splicing factor
XP_009692446.1	0.0743948	0.592424	0.125577	0.0501096	0.598627	0.0837075	0.104642	uncharacterized protein
XP_009692447.1	0.0228349	0.431607	0.0529067	0.0281195	0.498688	0.056387	0.054647	uncharacterized protein
XP_009692448.1	7.94182E-05	0.0794182	0.001	0.000111704	0.111704	0.001	0.001	histone H3
XP_009692449.1	0.000193773	0.193773	0.001	0.00013739	0.13739	0.001	0.001	late histone H2A.2.2
XP_009692450.1	0.0574968	0.667607	0.0861237	0.060017	0.641732	0.0935234	0.089824	transcription factor
XP_009692451.1	0.0443898	0.54017	0.0821774	0.0230958	0.412335	0.0560123	0.069095	ribosomal protein L19
XP_009692452.1	0.0229688	0.509043	0.0451217				0.0451217	splicing factor
XP_009692453.1	0.0270553	0.503677	0.0537156	0.0144086	0.548592	0.0262647	0.03999	uncharacterized protein
XP_009692454.1	0.147796	0.500832	0.295101	0.14612	0.549922	0.26571	0.280405	conserved hypothetical protein
XP_009692455.1	0.031446	0.496905	0.0632836	0.0250388	0.369026	0.067851	0.065567	cardiolipin synthetase
XP_009692456.1	0.135517	0.629941	0.215127	0.11483	0.61312	0.187288	0.201208	hypothetical protein
XP_009692457.1	0.0278472	0.406183	0.0685583	0.027986	0.379765	0.0736929	0.071126	uncharacterized protein
XP_009692458.1	0.0240515	0.786374	0.0305853	0.0201285	0.611755	0.0329028	0.031744	DNA-directed RNA polymerase
XP_009692459.1	0.0359088	0.588123	0.0610566	0.0525597	0.533576	0.0985047	0.079781	MAC/perforin
XP_009692460.1	0.0455544	0.290167	0.156994	0.0563379	0.320942	0.175539	0.166266	MAC/perforin
XP_009692461.1	0.00777007	0.379821	0.0204572	0.012414	0.371218	0.0334413	0.026949	uncharacterized protein
XP_009692462.1	0.141755	0.549585	0.257931	0.110872	0.405522	0.273405	0.265668	conserved hypothetical protein
XP_009692463.1	0.0113339	0.922827	0.0122817	0.00741642	0.952651	0.00778503	0.010033	DNA-directed RNA polymerase
XP_009692464.1	0.100641	0.620286	0.162249	0.146269	0.603423	0.242399	0.202324	conserved hypothetical protein
XP_009692465.1	0.0650213	0.485448	0.133941	0.0814638	0.599448	0.135898	0.134919	uncharacterized protein
XP_009692466.1	0.0122779	0.57634	0.0213032	0.0121186	0.748351	0.0161937	0.018748	uncharacterized protein
XP_009692467.1	0.0700294	1.01428	0.0690437	0.0605442	0.962624	0.062895	0.065969	uncharacterized protein
XP_009692468.1	0.0592548	0.492154	0.120399	0.0732117	0.552399	0.132534	0.126467	uncharacterized protein
XP_009692469.1	0.179059	0.59588	0.300495	0.132585	0.4496	0.294896	0.297696	conserved hypothetical protein
XP_009692470.1	0.0526926	0.376355	0.140008	0.0826381	0.31875	0.259257	0.199632	ubiquinol-cytochrome c reductase complex
XP_009692471.1	0.0148182	0.704968	0.0210197	0.011287	0.656192	0.0172008	0.01911	U4/U6-associated splicing factor
XP_009692472.1	0.00946487	0.166987	0.0566803				0.0566803	snoRNP protein
XP_009692473.1	0.0211169	0.280525	0.0752763	0.0280795	0.295139	0.0951399	0.085208	ribosomal RNA methyltransferase
XP_009692474.1	0.0895579	0.542333	0.165135	0.0714666	0.550194	0.129893	0.147514	methylase
XP_009692475.1	0.0921448	0.65682	0.140289	0.0887054	0.764437	0.11604	0.128165	conserved hypothetical protein
XP_009692476.1	0.103267	0.612591	0.168574	0.118592	0.510532	0.232291	0.200433	conserved hypothetical protein
XP_009692477.1	0.0427909	0.614549	0.0696298	0.0288252	0.561397	0.0513455	0.060488	predicted protein
XP_009692478.1	0.0163899	0.133686	0.1226	8.65782E-05	0.0865782	0.001	0.0618	snRNP protein
XP_009692479.1	0.0968447	0.458706	0.211126	0.0868075	0.424445	0.20452	0.207823	conserved hypothetical protein
XP_009692480.1	0.033812	0.367056	0.0921168	0.0398794	0.535706	0.0744428	0.08328	microfibrillar-associated protein
XP_009692481.1	0.0969306	0.355447	0.272701	0.10379	0.394471	0.263112	0.267907	conserved hypothetical protein
XP_009692482.1	0.0290179	0.485838	0.0597276	0.0306773	0.436404	0.0702957	0.065012	uncharacterized protein
XP_009692483.1	0.0127848	0.70212	0.0182088	0.0117186	0.670852	0.0174682	0.017839	Ran-binding protein
XP_009692484.1	0.0295029	0.677098	0.0435725	0.0280888	0.600532	0.0467732	0.045173	DEAD-box family helicase
XP_009692485.1	0.0944584	0.480065	0.196762	0.0886412	0.476615	0.185981	0.191371	uncharacterized protein
XP_009692486.1	0.00504211	0.312716	0.0161236	0.0160101	0.450887	0.0355079	0.025816	proteasome subunit alpha type
XP_009692487.1	0.0321472	0.528465	0.0608314	0.0171935	0.456958	0.0376259	0.049229	tyrosyl-tRNA synthetase
XP_009692488.1	0.0133575	0.465917	0.0286692	0.0159089	0.499559	0.031846	0.030258	conserved hypothetical protein
XP_009692489.1	0.0495971	0.696674	0.0711913	0.042932	0.435673	0.0985419	0.084867	exoribonuclease
XP_009692490.1	0.0949685	0.657746	0.144385	0.103135	0.507356	0.203279	0.173832	conserved hypothetical protein
XP_009692491.1	0.0590755	0.660348	0.0894611	0.0504746	0.523501	0.0964174	0.092939	condensin subunit
XP_009692492.1	0.0520774	0.724842	0.0718467	0.0367993	0.727258	0.0506	0.061223	conserved hypothetical protein
XP_009692493.1	0.0652864	0.608445	0.1073	0.0494363	0.613987	0.0805168	0.093908	conserved hypothetical protein
XP_009692494.1	0.0557598	0.498764	0.111796	0.0462115	0.434181	0.106434	0.109115	uncharacterized protein
XP_009692495.1	0.021033	0.329088	0.063913	0.0142853	0.279904	0.0510366	0.057475	GTPase-activating protein
XP_009692496.1	0.0320595	0.429502	0.0746434	0.0191194	0.517225	0.0369653	0.055804	conserved hypothetical protein

protein_id	Chitose_ka	Chitose_ks	Chitose_kaks	Buffeli_ka	Buffeli_ks	Buffeli_kaks	mean_kaks	function
XP_009692497.1	0.0849023	0.56684	0.149782	0.0855502	0.544978	0.156979	0.153381	uncharacterized protein
XP_009692498.1	0.0786122	0.137616	0.571245				0.571245	conserved hypothetical protein
XP_009692499.1	0.0705557	0.453726	0.155503	0.0507985	0.394941	0.128623	0.142063	uncharacterized protein
XP_009692500.1	0.036958	0.494362	0.074759	0.0182483	0.534125	0.0341649	0.054462	replication factor C subunit
XP_009692501.1	0.124281	0.378884	0.328018	0.133619	0.342093	0.390593	0.359305	uncharacterized protein
XP_009692502.1	0.00557131	0.745347	0.00747479	0.00508223	1.18099	0.00430336	0.005889	glyceraldehyde-3-phosphate dehydrogenase
XP_009692503.1	0.0100851	0.723636	0.0139367	0.00693093	0.577053	0.0120109	0.012974	cytochrome C1 precursor
XP_009692504.1	0.0353545	0.494685	0.0714687	0.0308385	0.512177	0.0602105	0.06584	uncharacterized protein
XP_009692505.1	0.0450569	0.422693	0.106595	0.0284473	0.323251	0.0880036	0.097299	syntaxin
XP_009692506.1	0.0127356	0.268531	0.047427	0.0160717	0.257588	0.0623932	0.05491	60S ribosomal protein L13
XP_009692507.1	0.0730684	0.558773	0.130766	0.073013	0.499146	0.146276	0.138521	uncharacterized protein
XP_009692508.1	0.0232384	0.452003	0.0514119	0.0136802	0.386246	0.0354183	0.043415	arginyl-tRNA synthetase
XP_009692509.1	0.04805	0.331319	0.145026	0.067667	0.406939	0.166283	0.155654	conserved hypothetical protein
XP_009692510.1	0.00968244	0.604103	0.0160278	0.0152174	0.439781	0.0346021	0.025315	50S ribosomal protein L14
XP_009692511.1	0.000280176	0.280176	0.001	0.000279443	0.279443	0.001	0.001	vacuolar H+-ATPase subunit
XP_009692512.1	0.0482745	0.788309	0.061238	0.0633726	0.821951	0.0771002	0.069169	uncharacterized protein
XP_009692513.1	0.000325334	0.325334	0.001				0.001	proteasome subunit
XP_009692514.1				0.108054	0.444713	0.242975	0.242975	conserved hypothetical protein
XP_009692515.1	0.0230855	0.461415	0.050032	0.028191	0.343581	0.0820504	0.066041	splicing factor
XP_009692516.1	0.0182249	0.463736	0.0393002	0.016673	0.436753	0.0381748	0.038738	transcription modulator
XP_009692517.1	0.0424522	0.46007	0.0922735	0.0528756	0.419195	0.126136	0.109205	conserved hypothetical protein
XP_009692518.1	0.0926151	0.0290972	3.18295	0.217573	0.135223	1.609	2.395975	hypothetical protein
XP_009692519.1	0.00538756	0.433319	0.0124332	0.00321115	0.258289	0.0124324	0.012433	40S ribosomal protein S9
XP_009692520.1	0.0662879	0.523921	0.126523	0.0777974	0.480045	0.162063	0.144293	conserved hypothetical protein
XP_009692521.1	0.0593819	0.438076	0.135552	0.0492445	0.362981	0.135667	0.135609	conserved hypothetical protein
XP_009692522.1	0.0764232	0.515665	0.148203	0.113071	0.462739	0.244352	0.196277	uncharacterized protein
XP_009692523.1	0.0614091	0.385802	0.159172	0.0704161	0.331115	0.212664	0.185918	conserved hypothetical protein
XP_009692524.1	0.0847398	0.538601	0.157333	0.072855	0.482148	0.151105	0.154219	cell surface/extracellular protein
XP_009692525.1	0.00903012	0.299729	0.0301276	4.83984E-05	0.0483984	0.001	0.015564	U6 snRNP protein Lsm2
XP_009692526.1	0.0541107	0.185514	0.29168	0.0521675	0.411594	0.126745	0.209212	bis-(5'-nucleosyl)-tetraphosphatase
XP_009692527.1	0.0342977	0.557036	0.0615718	0.0160356	0.343308	0.046709	0.05414	uncharacterized protein
XP_009692528.1	NA	NA	NA	NA	NA	NA	NA	uncharacterized protein
XP_009692529.1	0.107805	0.511243	0.210868	0.104699	0.6243	0.167706	0.189287	conserved hypothetical protein
XP_009692530.1	0.0676002	0.310217	0.217913				0.217913	uncharacterized protein
XP_009692532.1	0.0858777	0.484444	0.177271	0.106423	0.513917	0.207082	0.192177	hypothetical protein
XP_009692533.1	0.0315749	0.406609	0.0776541				0.0776541	uncharacterized protein
XP_009692534.1	0.0405126	0.808794	0.0500901	0.0359551	0.831106	0.0432618	0.046676	coat protein%2C gamma subunit
XP_009692535.1	0.00890619	0.260792	0.0341505	0.000239535	0.239535	0.001	0.017575	CRIPT protein
XP_009692536.1	0.022323	0.841653	0.0265229	0.022785	0.841666	0.0270713	0.026797	dynamin
XP_009692537.1	0.0591869	0.35777	0.165433	0.049459	0.27686	0.178642	0.172038	conserved hypothetical protein
XP_009692538.1	0.0132428	0.487113	0.0271862	0.0218442	0.516671	0.0422788	0.034732	glycoprotein endopeptidase
XP_009692539.1	0.019756	0.353929	0.0558192	0.0226468	0.235566	0.0961375	0.075978	26S proteasome subunit
XP_009692540.1	0.0326411	0.304464	0.107208	0.0290795	0.257015	0.113143	0.110175	conserved hypothetical protein
XP_009692541.1	0.0365046	0.0972646	0.375312	0.202756	0.262386	0.772739	0.574025	hypothetical protein
XP_009692542.1	0.068361	0.474452	0.144084	0.0723469	0.431078	0.167828	0.155956	uncharacterized protein
XP_009692543.1	0.0223139	0.342491	0.0651517	0.0253914	0.256594	0.0989555	0.082054	protein phosphatase regulator subunit
XP_009692544.1	0.00287029	0.586836	0.00489114	0.000372321	0.372321	0.001	0.002946	G protein beta subunit-like polypeptide
XP_009692545.1	0.125132	0.58725	0.213081	0.121271	0.489705	0.24764	0.23036	uncharacterized protein
XP_009692546.1	0.0460658	0.375247	0.122762	0.0433518	0.408653	0.106085	0.114423	conserved hypothetical protein
XP_009692547.1	0.0309638	0.122714	0.252325	0.0886891	0.256924	0.345195	0.29876	uncharacterized protein
XP_009692548.1	0.0406871	0.30476	0.133505	0.0597237	0.423402	0.141057	0.137281	uncharacterized protein
XP_009692549.1	0.026798	0.34435	0.077822				0.077822	uncharacterized protein
XP_009692550.1	0.0405097	0.359601	0.112652	0.0300456	0.309798	0.0969846	0.104818	uncharacterized protein

protein_id	Chitose_ka	Chitose_ks	Chitose_kaks	Buffeli_ka	Buffeli_ks	Buffeli_kaks	mean_kaks	function
XP_009692551.1	0.0519988	0.490085	0.106102	0.0458349	0.423345	0.108268	0.107185	conserved hypothetical protein
XP_009692552.1	0.00296774	0.607784	0.00488289	0.00318603	0.517238	0.0061597	0.005521	60S ribosomal protein L12
XP_009692553.1	0.0722302	0.459276	0.15727	0.0809125	0.339581	0.238272	0.197771	uncharacterized protein
XP_009692554.1	0.055931	1.14915	0.0486714	0.0714029	1.64099	0.0435122	0.046092	uncharacterized protein
XP_009692555.1	0.0414617	0.489159	0.0847611	0.0316576	0.565974	0.0559347	0.070348	tRNA-pseudouridine synthase I
XP_009692556.1	0.000169083	0.169083	0.001	0.000077432	0.077432	0.001	0.001	40S ribosomal protein S11
XP_009692557.1	0.0315813	0.551498	0.0572646	0.0347923	0.534398	0.0651057	0.061185	conserved hypothetical protein
XP_009692558.1	2.01858E-05	0.0201858	0.001	NA	NA	NA	NA	BolA-like protein
XP_009692559.1	0.0211246	0.436014	0.0484493	0.020275	0.520312	0.0389671	0.043708	conserved hypothetical protein
XP_009692560.1	0.0222969	0.416376	0.0535499	0.0275762	0.381845	0.0722184	0.062884	conserved hypothetical protein
XP_009692561.1	0.0446375	0.321679	0.138764	0.0347676	0.320389	0.108517	0.123641	conserved hypothetical protein
XP_009692562.1	0.0538811	0.606581	0.0888276	0.0609556	0.61088	0.0997833	0.094305	conserved hypothetical protein
XP_009692564.1	0.0215909	0.654526	0.0329871	0.0175472	0.659848	0.0265928	0.02979	eukaryotic translation initiation factor
XP_009692565.1	0.0210499	0.438083	0.0480499	0.0226835	0.410549	0.0552516	0.051651	prenyltransferase
XP_009692566.1	0.00658996	0.499369	0.0131966	0.000465717	0.465717	0.001	0.007098	40S ribosomal protein S24
XP_009692567.1	0.324489	0.493106	0.658052	0.261152	0.384422	0.679337	0.668694	hypothetical protein
XP_009692568.1	0.0185855	0.831428	0.0223537	0.00641427	0.5742	0.0111708	0.016762	DEAD-box family helicase
XP_009692569.1	0.0355171	0.759443	0.0467673	0.0306064	0.77916	0.0392812	0.043024	uncharacterized protein
XP_009692570.1	0.0281731	1.08496	0.025967	0.0295807	1.00616	0.0293996	0.027683	RNA-binding protein Puf1
XP_009692571.1	0.216825	0.225599	0.961107	0.217997	0.123085	1.77111	1.366108	hypothetical protein
XP_009692572.1	0.0370497	0.299567	0.123678	0.0467875	0.352253	0.132824	0.128251	uncharacterized protein
XP_009692573.1	0.0583787	0.181919	0.320905	0.105858	0.105664	1.00184	0.661373	hypothetical protein
XP_009692574.1	0.0869953	0.692299	0.125661	0.0698052	0.598149	0.116702	0.121181	conserved hypothetical protein
XP_009692575.1	0.0878039	0.200892	0.437071	0.0944698	0.144433	0.654073	0.545572	hypothetical protein
XP_009692576.1	0.03834	1.07126	0.0357896	0.0302793	1.01187	0.029924	0.032857	uncharacterized protein
XP_009692577.1	0.024363	0.429707	0.0566967	0.0299929	0.584281	0.0513329	0.054015	uncharacterized protein
XP_009692578.1	0.0492439	0.733699	0.0671173	0.0500585	0.674626	0.0742018	0.07066	conserved hypothetical protein
XP_009692579.1	0.0592742	0.76049	0.0779421	0.0378473	0.926686	0.0408415	0.059392	uncharacterized protein
XP_009692580.1	0.0528615	0.41867	0.12626	0.0510248	0.432812	0.117891	0.122076	uncharacterized protein
XP_009692581.1	0.0190434	0.605031	0.031475	0.0172625	0.561867	0.0307234	0.031099	vesicle transport protein
XP_009692582.1	0.0368988	0.552762	0.0667534	0.0397551	0.442196	0.0899038	0.078329	uncharacterized protein
XP_009692583.1	0.0177992	0.453543	0.0392449	0.0171099	0.193908	0.0882376	0.063741	uncharacterized protein
XP_009692584.1	0.0477169	0.696809	0.0684791	0.046537	0.711689	0.0653895	0.066934	conserved hypothetical protein
XP_009692585.1	0.0630037	0.474243	0.132851	0.0666334	0.441792	0.150825	0.141838	uncharacterized protein
XP_009692586.1	0.0528098	0.396799	0.133089	0.0590701	0.413764	0.142763	0.137926	conserved hypothetical protein
XP_009692587.1	0.0327685	0.480392	0.068212	0.0487876	0.405329	0.120365	0.094288	peptide chain release factor
XP_009692588.1	0.0382621	0.577031	0.0663086	0.0565346	0.609408	0.0927696	0.079539	peptide chain release factor
XP_009692589.1	0.0881788	0.678789	0.129906	0.0867065	0.520135	0.1667	0.148303	uncharacterized protein
XP_009692590.1	0.111815	0.872298	0.128184	0.12422	0.41442	0.299745	0.213965	hypothetical protein
XP_009692591.1	0.090275	0.115867	0.779124				0.779124	hypothetical protein
XP_009692592.1	0.0208625	0.333715	0.0625158	0.0292244	0.407991	0.0716299	0.067073	uncharacterized protein
XP_009692593.1	0.00581699	1.50263	0.0038712	0.0054652	0.480691	0.0113695	0.00762	60S ribosomal protein L36
XP_009692594.1	0.0620914	0.54202	0.114556	0.0608075	0.643894	0.0944372	0.104497	conserved hypothetical protein
XP_009692595.1	0.0243406	0.349883	0.069568	0.0140241	0.350143	0.0400526	0.05481	26S proteasome regulatory subunit
XP_009692596.1	0.0169139	0.437979	0.0386181	0.0123262	0.333305	0.0369818	0.0378	uncharacterized protein
XP_009692597.1	0.0774568	0.254437	0.304424	0.0978638	0.322066	0.303862	0.304143	conserved hypothetical protein
XP_009692598.1	0.0374276	0.621113	0.0602589	0.0205352	0.505377	0.0406335	0.050446	uncharacterized protein
XP_009692599.1	0.0940889	0.379144	0.248161	0.0953369	0.331313	0.287755	0.267958	conserved hypothetical protein
XP_009692600.1	0.0583059	0.451899	0.129024	0.0527409	0.491997	0.107197	0.118111	uncharacterized protein
XP_009692601.1	0.00694215	0.607506	0.0114273	0.00571336	0.5511	0.0103672	0.010897	DNA repair protein Rad51
XP_009692602.1	0.038611	0.592116	0.0652085	0.035157	0.517295	0.0679631	0.066586	DNA damage response protein
XP_009692603.1	0.118162	0.0790895	1.49403				1.49403	hypothetical protein
XP_009692604.1	0.0288452	0.691571	0.0417097	0.0240893	0.73382	0.0328273	0.037268	uncharacterized protein

protein_id	Chitose_ka	Chitose_ks	Chitose_kaks	Buffeli_ka	Buffeli_ks	Buffeli_kaks	mean_kaks	function
XP_009692605.1	0.0565271	0.146009	0.387147				0.387147	hypothetical protein
XP_009692606.1	0.032497	0.107365	0.302677				0.302677	hypothetical protein
XP_009692607.1	0.231663	0.108403	2.13706				2.13706	hypothetical protein
XP_009692608.1	0.056291	0.658916	0.0854298	0.0470911	0.637347	0.0738862	0.079658	uncharacterized protein
XP_009692609.1	0.0828418	0.424968	0.194937	0.0672699	0.36372	0.18495	0.189943	conserved hypothetical protein
XP_009692610.1	0.0141428	0.614749	0.0230058	0.0181768	1.10435	0.0164593	0.019733	protein mago nashi homolog
XP_009692611.1	0.0706374	0.533104	0.132502	0.0666467	0.523904	0.127212	0.129857	uncharacterized protein
XP_009692612.1	0.0800652	0.481073	0.16643	0.0710894	0.565014	0.125819	0.146124	lipid-binding/transfer protein
XP_009692613.1	0.109643	0.311464	0.352024	0.0866752	0.344542	0.251566	0.301795	conserved hypothetical protein
XP_009692614.1	0.0655543	0.366879	0.178681	0.0795983	0.496458	0.160332	0.169507	uncharacterized protein
XP_009692615.1	0.0201136	0.754397	0.0266618	0.0218973	0.686386	0.0319022	0.029282	acetyl-coenzyme A synthetase
XP_009692616.1	0.0321907	0.501343	0.0642089	0.0344107	0.488026	0.07051	0.067359	importin beta/transportin
XP_009692617.1	0.030701	0.394455	0.0778315				0.0778315	thioredoxin
XP_009692618.1	0.114604	0.634774	0.180543	0.121486	0.538044	0.225793	0.203168	uncharacterized protein
XP_009692619.1	0.0448638	0.505884	0.088684	0.0425616	0.523011	0.0813779	0.085031	conserved hypothetical protein
XP_009692620.1	0.112798	0.56936	0.198113	0.119928	0.566895	0.211552	0.204833	uncharacterized protein
XP_009692621.1	0.072479	0.854904	0.0847803	0.0682666	1.02085	0.0668725	0.075826	gamma adaptin
XP_009692622.1	0.0468715	1.00248	0.0467556	0.0439048	0.973318	0.0451084	0.045932	uncharacterized protein
XP_009692623.1	0.0892904	0.478631	0.186554	0.0850156	0.545013	0.155988	0.171271	conserved hypothetical protein
XP_009692624.1	0.0372748	0.595343	0.0626108	0.0255923	0.402309	0.0636135	0.063112	uncharacterized protein
XP_009692625.1	0.0381203	0.523771	0.0727805	0.0388051	0.39236	0.0989017	0.085841	uncharacterized protein
XP_009692626.1	0.0644503	0.406286	0.158633	0.0650549	0.374844	0.173552	0.166093	conserved hypothetical protein
XP_009692627.1	0.0835635	0.591889	0.141181	0.0952955	0.474944	0.200646	0.170913	conserved hypothetical protein
XP_009692628.1	0.0448763	0.559936	0.0801455	0.0343773	0.528339	0.0650668	0.072606	conserved hypothetical protein
XP_009692629.1	0.0375097	0.482104	0.077804	0.0299429	0.450006	0.066539	0.072171	uncharacterized protein
XP_009692630.1	0.04277	0.516561	0.0827977	0.0287648	0.545345	0.0527461	0.067772	uncharacterized protein
XP_009692631.1	0.0908017	0.349611	0.259722	0.0960095	0.559009	0.17175	0.215736	uncharacterized protein
XP_009692632.1	0.141623	0.5291	0.267667	0.160626	0.531731	0.302082	0.284875	conserved hypothetical protein
XP_009692633.1	0.254127	0.336333	0.755582	0.206793	0.410353	0.50394	0.629761	conserved hypothetical protein
XP_009692634.1	0.0700768	0.537888	0.130281	0.0408508	0.408935	0.0998956	0.115088	uncharacterized protein
XP_009692635.1	0.0811574	0.470811	0.172378	0.0708452	0.380617	0.186133	0.179256	uncharacterized protein
XP_009692636.1	0.0632431	0.396538	0.159488				0.159488	uncharacterized protein
XP_009692637.1	0.0854808	0.584564	0.14623	0.0536429	0.357666	0.14998	0.148105	conserved hypothetical protein
XP_009692638.1	0.156237	0.52951	0.29506	0.141613	0.40517	0.349514	0.322287	conserved hypothetical protein
XP_009692639.1	0.0540372	0.46682	0.115756	0.0456521	0.300611	0.151865	0.13381	uncharacterized protein
XP_009692640.1	0.0689145	0.462487	0.149009	0.0359042	0.384969	0.0932652	0.121137	nucleosome/chromatin assembly factor
XP_009692642.1	0.0412916	0.53489	0.0771964	0.0254187	0.39569	0.0642389	0.070718	uncharacterized protein
XP_009692644.1	0.0633545	0.40031	0.158264	0.0564341	0.412771	0.13672	0.147492	filament assembling protein
XP_009692647.1	0.0318997	0.572638	0.0557066	0.0263627	0.563277	0.0468023	0.051254	CTP-phosphoethanolamine cytidylyltransferase
XP_009692648.1	0.0306189	0.512624	0.0597297	0.0135225	0.234873	0.0575735	0.058652	conserved hypothetical protein
XP_009692649.1	0.0851237	0.606652	0.140317	0.0860239	0.553043	0.155547	0.147932	conserved hypothetical protein
XP_009692650.1	0.0431908	0.316788	0.13634	0.0302502	0.274035	0.110388	0.123364	Plasmodium falciparum CPW-WPC repeat containing protein
XP_009692651.1	0.0207497	1.08323	0.0191554	0.0175094	0.994942	0.0175984	0.018377	splicing factor 3b subunit
XP_009692652.1	0.0281892	0.404351	0.0697146	0.0550009	0.316843	0.17359	0.121652	hypothetical protein
XP_009692653.1	0.0338903	0.481399	0.0703996	0.0119536	0.395556	0.0302198	0.05031	Ran binding protein 1
XP_009692654.1	0.0380725	0.52414	0.0726381	0.0376736	0.3904	0.0965	0.084569	50S ribosomal protein L7Ae
XP_009692656.1	0.0526563	0.957112	0.0550159	0.049515	1.1624	0.0425972	0.048807	conserved hypothetical protein
XP_009692657.1	0.00955485	0.576157	0.0165837	0.00687569	0.599793	0.0114634	0.014024	molecular chaperone ClpB
XP_009692658.1	0.125955	0.708932	0.177669	0.199878	0.777253	0.257159	0.217414	uncharacterized protein
XP_009692659.1				0.202151	0.244124	0.828066	0.828066	hypothetical protein
XP_009692660.1	0.0838125	0.664191	0.126187	0.0904266	0.682536	0.132486	0.129336	conserved hypothetical protein
XP_009692661.1	0.111356	0.512611	0.217233	0.055836	0.378347	0.147579	0.182406	conserved hypothetical protein
XP_009692662.1	0.0178383	0.375533	0.0475014	0.00523854	0.359291	0.0145802	0.031041	uncharacterized protein

protein_id	Chitose_ka	Chitose_ks	Chitose_kaks	Buffeli_ka	Buffeli_ks	Buffeli_kaks	mean_kaks	function
XP_009692663.1	0.0379665	0.454379	0.0835568	0.0366662	0.541296	0.0677377	0.075647	small heat-shock protein
XP_009692664.1	0.030975	0.479667	0.064576	0.00877651	0.422721	0.0207619	0.042669	ATP-dependent Clp protease proteolytic subunit
XP_009692665.1	0.0621684	0.365995	0.169861	0.0470344	0.337046	0.139549	0.154705	hypothetical protein
XP_009692666.1	0.0358256	0.377671	0.0948592	0.033184	0.270211	0.122807	0.108833	uncharacterized protein
XP_009692667.1	0.0255359	0.928133	0.0275132	0.0232641	0.890624	0.0261211	0.026817	predicted protein
XP_009692668.1	0.0192387	0.722342	0.0266338	0.0177025	0.718057	0.0246533	0.025644	uncharacterized protein
XP_009692669.1	0.173836	0.340004	0.511276	0.174919	0.265366	0.659162	0.585219	hypothetical protein
XP_009692670.1	0.016792	0.334956	0.0501319	0.0221954	0.363654	0.0610345	0.055583	uncharacterized protein
XP_009692671.1	0.000162005	0.162005	0.001	5.43242E-05	0.0543242	0.001	0.001	40S ribosomal protein S20
XP_009692672.1	0.0061847	0.360903	0.0171367	1.03016E-05	0.0103016	0.001	0.009068	GNAT-family N-acetyltransferase
XP_009692673.1	NA	NA	NA				NA	conserved hypothetical protein
XP_009692674.1	0.0961558	0.421081	0.228355	0.0804433	0.512369	0.157003	0.192679	uncharacterized protein
XP_009692675.1	0.0568563	0.37345	0.152246	0.0428657	0.459864	0.0932138	0.12273	uncharacterized protein
XP_009692676.1	0.273431	0.343625	0.795725	0.194931	0.297781	0.654612	0.725168	hypothetical protein
XP_009692677.1	0.100706	0.60113	0.167527	0.091331	0.412967	0.221158	0.194343	conserved hypothetical protein
XP_009692678.1	0.0612345	0.286575	0.213677	0.0546191	0.250522	0.218021	0.215849	conserved hypothetical protein
XP_009692679.1	0.0646353	0.49223	0.131311	0.0664432	0.464144	0.143152	0.137232	conserved hypothetical protein
XP_009692681.1	0.108342	0.561682	0.192888	0.0948784	0.477419	0.198732	0.19581	conserved hypothetical protein
XP_009692682.1	0.123011	0.410651	0.299551	0.0965172	0.416748	0.231596	0.265574	hypothetical protein
XP_009692683.1	0.0219743	0.432526	0.0508046	0.0293837	0.418485	0.0702145	0.06051	26S proteasome ATPase subunit
XP_009692684.1	0.0182948	0.449681	0.0406839	0.0153717	0.400991	0.0383343	0.039509	U5 snRNP-specific subunit
XP_009692685.1	0.0668594	0.277993	0.240508				0.240508	conserved hypothetical protein
XP_009692687.1	0.105517	0.472776	0.223186	0.0922855	0.615411	0.149958	0.186572	hypothetical protein
XP_009692688.1	0.0655155	0.602641	0.108714	0.0597875	0.545155	0.109671	0.109192	uncharacterized protein
XP_009692690.1	0.101674	1.17814	0.0863	0.0900221	1.05097	0.0856559	0.085978	conserved hypothetical protein
XP_009692691.1	0.169201	0.337238	0.501726	0.0916716	0.540091	0.169733	0.33573	hypothetical protein
XP_009692692.1	0.182783	0.289984	0.63032	0.0831439	0.143653	0.578784	0.604552	hypothetical protein
XP_009692693.1	0.116359	0.518427	0.224447	0.0840271	0.514263	0.163393	0.19392	conserved hypothetical protein
XP_009692694.1	0.106604	0.502452	0.212167	0.0955628	0.428207	0.223169	0.217668	hypothetical protein
XP_009692695.1	0.00960098	0.268785	0.0357199	0.0376894	0.48117	0.0783285	0.057024	endosome sorting protein
XP_009692696.1	8.22433E-05	0.0822433	0.001	6.71178E-05	0.0671178	0.001	0.001	translation initiation factor
XP_009692697.1	0.0499543	0.442364	0.112926	0.0298722	0.266988	0.111886	0.112406	glucose-inhibited division protein a-like protein
XP_009692698.1	0.0602753	0.598862	0.10065	0.0587032	0.599505	0.0979193	0.099285	serine/threonine kinase
XP_009692699.1	0.0175362	0.420559	0.0416973				0.0416973	uncharacterized protein
XP_009692700.1	0.0611582	0.514075	0.118967	0.0594469	0.562123	0.105754	0.112361	Pumilio RNA-binding region repeat containing protein
XP_009692701.1	0.0290539	0.1103	0.263408				0.263408	uncharacterized protein
XP_009692702.1	0.0304216	0.237275	0.128212	0.0298016	0.207435	0.143667	0.135939	uncharacterized protein
XP_009692703.1	0.040254	0.185954	0.216472	0.0482276	0.386338	0.124833	0.170652	leucine carboxyl methyltransferase
XP_009692704.1	0.101678	0.567777	0.179081	0.0335022	0.259966	0.128871	0.153976	uncharacterized protein
XP_009692705.1	0.076889	0.375361	0.20484	0.091513	0.39719	0.230401	0.21762	structure-specific recognition protein 1
XP_009692706.1	0.13211	0.142223	0.928893				0.928893	hypothetical protein
XP_009692707.1	0.0432012	0.487423	0.0886319	0.0392429	0.838566	0.0467976	0.067715	structure-specific recognition protein 1
XP_009692708.1	0.00616673	0.492421	0.0125233	0.00932091	0.390432	0.0238733	0.018198	60S ribosomal L1/L10a protein
XP_009692710.1	0.0732915	0.34348	0.213379	0.127139	0.58688	0.216635	0.215007	conserved hypothetical protein
XP_009692711.1	0.054355	0.674691	0.0805629	0.053673	0.587145	0.0914136	0.085988	uncharacterized protein
XP_009692712.1	0.0770488	0.466936	0.165009	0.122263	0.643546	0.189983	0.177496	uncharacterized protein
XP_009692713.1	0.0395505	0.394265	0.100315	0.0302445	0.138592	0.218227	0.159271	uncharacterized protein
XP_009692714.1	0.0605082	0.60435	0.100121	0.0463166	0.650195	0.0712349	0.085678	uncharacterized protein
XP_009692715.1	0.0710983	0.689315	0.103143	0.079111	0.863337	0.0916341	0.097389	conserved hypothetical protein
XP_009692716.1	0.130136	0.0796641	1.63355				1.63355	hypothetical protein
XP_009692717.1	0.0424637	0.457973	0.0927209	0.0410051	0.432095	0.0948983	0.09381	uncharacterized protein
XP_009692719.1	0.111207	0.409565	0.271524	0.0826549	0.32339	0.255589	0.263556	uncharacterized protein
XP_009692720.1	0.021061	0.397448	0.0529905	0.0175063	0.399421	0.0438293	0.04841	uncharacterized protein

protein_id	Chitose_ka	Chitose_ks	Chitose_kaks	Buffeli_ka	Buffeli_ks	Buffeli_kaks	mean_kaks	function
XP_009692721.1	0.00422403	0.28443	0.0148509	0.0128817	0.403576	0.031919	0.023385	uncharacterized protein
XP_009692722.1	0.0357578	0.295488	0.121013				0.121013	uncharacterized protein
XP_009692723.1	0.0445344	0.506125	0.0879909	0.0412599	0.792076	0.0520908	0.070041	signal peptidase
XP_009692725.1	0.0326462	0.265316	0.123046				0.123046	conserved hypothetical protein
XP_009692726.1	0.077432	0.592067	0.130783	0.0794042	0.569712	0.139376	0.13508	uncharacterized protein
XP_009692727.1	0.0671603	0.783991	0.0856646	0.0671599	0.699909	0.0959552	0.09081	uncharacterized protein
XP_009692728.1	0.00033422	0.33422	0.001	0.000786185	0.786185	0.001	0.001	60S ribosomal protein L32
XP_009692729.1	0.110688	0.271627	0.407501	0.0936554	0.231905	0.403852	0.405676	conserved hypothetical protein
XP_009692730.1				0.114113	0.289028	0.394818	0.394818	uncharacterized protein
XP_009692731.1	0.152373	0.56086	0.271677				0.271677	conserved hypothetical protein
XP_009692736.1	0.00305138	0.416966	0.00731806	0.00113117	0.531756	0.00212723	0.004723	tubulin subunit alpha
XP_009692737.1	0.000268011	0.268011	0.001				0.001	conserved hypothetical protein
XP_009692738.1	0.0778773	0.454412	0.17138	0.0568927	0.426527	0.133386	0.152383	conserved hypothetical protein
XP_009692739.1	0.0472766	0.561331	0.0842223	0.0605066	0.250838	0.241218	0.16272	conserved hypothetical protein
XP_009692740.1	0.0289193	0.356461	0.0811289	0.0313108	0.362892	0.0862815	0.083705	T-complex protein 1 chaperonin
XP_009692741.1	0.0932318	0.584535	0.159497	0.0801903	0.548294	0.146254	0.152875	DEAD-box family helicase
XP_009692742.1	0.0410862	0.416775	0.0985813				0.0985813	uncharacterized protein
XP_009692744.1	0.157373	0.539449	0.29173				0.29173	uncharacterized protein
XP_009692745.1	0.159083	0.388756	0.409211				0.409211	uncharacterized protein
XP_009692749.1	0.102516	0.784454	0.130684	0.10429	0.633647	0.164586	0.147635	conserved hypothetical protein
XP_009692750.1	0.118934	0.46661	0.254889	0.0936751	0.421331	0.222331	0.23861	uncharacterized protein
XP_009692751.1	0.0459169	0.626412	0.0733015	0.0446059	0.439747	0.101435	0.087368	uncharacterized protein
XP_009692753.1	0.0660168	0.779343	0.0847082	0.0957197	0.716407	0.133611	0.10916	conserved hypothetical protein
XP_009692754.1	0.085967	0.503173	0.17085	0.0513837	0.423817	0.12124	0.146045	dimethyladenosine transferase
XP_009692755.1	0.116666	0.54085	0.215709	0.121869	0.471865	0.258271	0.23699	Requim%2C req/dpf2
XP_009692756.1	0.000207748	0.207748	0.001	0.000214172	0.214172	0.001	0.001	histone H2B variant 1
XP_009692757.1	0.0366397	0.504583	0.0726139	0.00976191	0.0688186	0.14185	0.107232	conserved hypothetical protein
XP_009692758.1	0.00505785	0.475133	0.0106451	0.000350478	0.350478	0.001	0.005823	conserved hypothetical protein
XP_009692759.1	0.0534316	0.419726	0.127301				0.127301	conserved hypothetical protein
XP_009692760.1	0.14468	0.479884	0.30149	0.121113	0.362513	0.334092	0.317791	uncharacterized protein
XP_009692763.1	0.0328385	0.260049	0.126278	0.073707	0.607367	0.121355	0.123816	uncharacterized protein
XP_009692764.1	0.0214069	0.366377	0.0584288	0.0266479	0.324143	0.0822101	0.070319	uncharacterized protein
XP_009692766.1	0.0183669	0.672127	0.0273266	0.0145334	0.744312	0.019526	0.023426	uncharacterized protein
XP_009692767.1	0.0621659	0.396898	0.156629	0.0491085	0.442594	0.110956	0.133792	methionine aminopeptidase
XP_009692768.1	0.128078	0.600797	0.213181	0.140588	0.721366	0.194892	0.204037	uncharacterized protein
XP_009692770.1	0.119301	0.512021	0.233	0.108466	0.618462	0.17538	0.20419	hypothetical protein
XP_009692771.1	0.0665069	0.489028	0.135998	0.0527376	0.406061	0.129876	0.132937	uncharacterized protein
XP_009692772.1	0.00704334	0.585826	0.0120229	0.00122244	0.593567	0.00205948	0.007041	chaperonin HSP60
XP_009692773.1	0.0787415	0.459728	0.171279	0.0568887	0.537825	0.105776	0.138527	conserved hypothetical protein
XP_009692774.1	0.0450748	0.375583	0.120013	0.0449278	0.420499	0.106844	0.113428	uncharacterized protein
XP_009692775.1				0.103087	0.393537	0.261951	0.261951	conserved hypothetical protein
XP_009692777.1	0.103404	0.609123	0.169759	0.110484	0.624024	0.17705	0.173405	uncharacterized protein
XP_009692778.1	0.0455799	0.276414	0.164897	0.0326099	0.231858	0.140646	0.152772	superoxide dismutase
XP_009692779.1	0.0611927	0.355108	0.172321	0.017296	0.0690169	0.250605	0.211463	tRNA modification GTPase
XP_009692780.1	0.0924613	0.710899	0.130063				0.130063	conserved hypothetical protein
XP_009692781.1	0.0835739	1.2616	0.0662441	0.0555956	1.17725	0.047225	0.056735	uncharacterized protein
XP_009692784.1	0.11343	0.523799	0.216552				0.216552	conserved hypothetical protein
XP_009692786.1	0.31662	0.0434908	7.28017	0.324454	0.0468645	6.92323	7.1017	26S protease regulatory subunit
XP_009692787.1	0.145818	0.345084	0.422559				0.422559	uncharacterized protein
XP_009692788.1	0.135487	0.411042	0.329617	0.121847	0.360882	0.337636	0.333626	hypothetical protein
XP_009692789.1	0.113459	0.423361	0.267995	0.103122	0.373816	0.275864	0.271929	conserved hypothetical protein
XP_009692792.1	0.0591942	0.556267	0.106413	0.0717743	0.678068	0.105851	0.106132	uncharacterized protein
XP_009692793.1	0.0671617	0.197721	0.33968				0.33968	uncharacterized protein

protein_id	Chitose_ka	Chitose_ks	Chitose_kaks	Buffeli_ka	Buffeli_ks	Buffeli_kaks	mean_kaks	function
XP_009692794.1	0.011051	1.21988	0.00905909	0.0113305	1.29918	0.00872127	0.00889	splicing factor
XP_009692797.1	0.0734418	0.203001	0.361781	0.0384337	0.0674591	0.569734	0.465757	hypothetical protein
XP_009692798.1	0.00934859	0.361057	0.0258923	0.00911697	0.567136	0.0160755	0.020984	peroxiredoxin 1
XP_009692799.1	0.184577	0.860634	0.214466	0.204836	0.903447	0.226727	0.220597	conserved hypothetical protein
XP_009692800.1	0.000115721	0.115721	0.001	0.0111572	1.0659	0.0104674	0.005734	conserved hypothetical protein
XP_009692801.1	0.0725136	0.40784	0.177799	0.0672231	0.643894	0.104401	0.1411	conserved hypothetical protein
XP_009692802.1	0.050522	0.682535	0.0740211	0.0408516	0.621847	0.065694	0.069858	FG-GAP repeat containing protein
XP_009692803.1	0.0643851	0.00129133	49.8594				49.8594	hypothetical protein
XP_009692804.1	0.00545181	0.160801	0.033904				0.033904	conserved hypothetical protein
XP_009692805.1	0.0364574	0.184139	0.197988	0.0219471	0.10698	0.20515	0.201569	conserved hypothetical protein
XP_009692806.1	0.0716106	0.475666	0.150548	0.0730189	0.435366	0.167718	0.159133	conserved hypothetical protein
XP_009692807.1	0.0367075	0.392008	0.0936398				0.0936398	cell cycle regulator protein
XP_009692809.1				0.0911297	0.263344	0.346048	0.346048	conserved hypothetical protein
XP_009692810.1	0.105495	0.628575	0.167833	0.106263	0.723958	0.14678	0.157307	uncharacterized protein
XP_009692811.1	0.05103	0.465504	0.109623	0.048052	0.484405	0.099198	0.10441	50S ribosomal protein L16
XP_009692812.1	NA	NA	NA	NA	NA	NA	NA	conserved hypothetical protein
XP_009692813.1	0.0372792	0.975351	0.0382213	0.0291231	0.582822	0.049969	0.044095	phosphatidate cytidylyltransferase
XP_009692814.1	0.0239788	0.37174	0.0645043	0.0176186	0.256544	0.0686765	0.06659	ferredoxin
XP_009692815.1	0.00296554	0.291309	0.01018	0.000306428	0.306428	0.001	0.00559	40S ribosomal protein S15
XP_009692816.1	0.07939	0.495542	0.160208	NA	NA	NA	0.160208	surface protein
XP_009692817.1	0.135565	0.259622	0.522165				0.522165	conserved hypothetical protein
XP_009692818.1	0.0342099	0.921075	0.0371413	0.0176267	0.619867	0.0284363	0.032789	conserved hypothetical protein
XP_009692819.1	0.0879371	0.574334	0.153111	0.0652425	0.469417	0.138986	0.146048	conserved hypothetical protein
XP_009692820.1	0.125929	0.500592	0.251561	0.0978724	0.271953	0.359887	0.305724	conserved hypothetical protein
XP_009692821.1	0.0198102	0.69163	0.0286428				0.0286428	conserved hypothetical protein
XP_009692826.1				0.058085	0.296174	0.196118	0.196118	uncharacterized protein
XP_012965617.1	0.16241	0.0226319	7.17616	0.100293	0.0183469	5.46648	6.32132	cytochrome c oxidase subunit 1
