# Supplementary material for: Analysis of Theileria orientalis draft genome sequences reveals potential species-level divergence of the Ikeda, Chitose and Buffeli genotypes
Source: BMC Genomics. 2018 Apr 27;19:298. doi: 10.1186/s12864-018-4701-2 (PMC5921998; doi:10.1186/s12864-018-4701-2)
Supplement: Supplementary file 15 — Primers used in this study. Primers used for Sanger sequencing validation of the SNV calling pipeline. (DOC 44 kb) [file 12864_2018_4701_MOESM15_ESM.doc]

Additional file 15: Primers used for validation of SNV variant calling pipeline

| Primer name | Sequence | Chromosome | Product size (bp) |
| --- | --- | --- | --- |
| SNPvalChr1_1F | ACGTCCGAGTCTGATCCTGA | NC_025260 | 857 |
| SNPvalChr1_1R | CAGAGTCAAGGCGCTAACGA | NC_025260 |
|  |
| SNPvalChr1_2F | CGTACGTGAGGCAGCAGATA | NC_025260 | 733 |
| SNPvalChr1_2R | GCCACATTTTCCCTAAAAGTTCCA | NC_025260 |
|  |
| SNPvalChr2_1F | GGGTCAACTCAAGGAACAGGA | NC_025261 | 818 |
| SNPvalChr2_1R | ACACGTTACTCAGCGGAGAG | NC_025261 |
|  |
| SNPvalChr2_2F | GCGCCGGATAAGAAGTACCA | NC_025261 | 633 |
| SNPvalChr2_2R | TTCGACGCCTAGGTAGTCCA | NC_025261 |
|  |
| SNPvalChr2_3F | CATCCTGAAGAGTAATTTGGGGC | NC_025261 | 811 |
| SNPvalChr2_3R | CACGCAGAAACTGTTGTCGTT | NC_025261 |
|  |
| SNPvalChr3_1F | CCCTTTTAAAAATACTCAACTTGCTGAATG | NC_025262 | 749 |
| SNPvalChr3_1R | AAAGGTGTATTGGAGTAGAACAGCA | NC_025262 |
|  |
| SNPvalChr3_2F | CTCAAAAAGGGCCAAAAACGAAAAT | NC_025262 | 689 |
| SNPvalChr3_2R | TACATTTTATTTTCATCACCCTGTTACCT | NC_025262 |
|  |
| SNPvalChr3_3F | GCTCCCTCCTAGTTTTACACCTT | NC_025262 | 860 |
| SNPvalChr3_3R | TGATATGTGGAAGGATAAGAACCCC | NC_025262 |
|  |
| SNPvalChr4_1F | CCTGAGCTTGGAGAGTGAGAAAA | NC_025263 | 691 |
| SNPvalChr4_1R | TGCCCCCAATTCCGTGTC | NC_025263 |
|  |
| SNPvalChr4_2F | TTTACGCCCTTGTCCCTGTC | NC_025263 | 661 |
| SNPvalChr4_2R | TTTTAGATCCAAACAGACTGTATAATATGGAC | NC_025263 |
|  |
